# New insights into the FLPergic complements of parasitic nematodes: Informing deorphanisation approaches

**DOI:** 10.1016/j.euprot.2014.04.002

**Published:** 2014-04-19

**Authors:** Ciaran J. McCoy, Louise E. Atkinson, Mostafa Zamanian, Paul McVeigh, Tim A. Day, Michael J. Kimber, Nikki J. Marks, Aaron G. Maule, Angela Mousley

**Affiliations:** a Molecular Biosciences-Parasitology, Institute for Global Food Security, School of Biological Sciences, Queen's University Belfast, Belfast, UK; b Department of Biomedical Sciences, Iowa State University, Ames, IA, USA

**Keywords:** FMRFamide-like peptide, FLP, FLP-receptor, Neuropeptide GPCR, Nematode

## Abstract

FMRFamide-like peptide (FLP) receptors are appealing as putative anthelmintic targets. To date, 31 *flp*-encoding genes have been identified in *Caenorhabditis elegans* and thirteen FLP-activated G-protein coupled receptors (FLP-GPCRs) have been reported. The lack of knowledge on FLPs and FLP-GPCRs in parasites impedes their functional characterisation and chemotherapeutic exploitation. Using homology-based BLAST searches and phylogenetic analyses this study describes the identification of putative *flp* and *flp*-GPCR gene homologues in 17 nematode parasites providing the first pan-phylum genome-based overview of the FLPergic complement. These data will facilitate FLP-receptor deorphanisation efforts in the quest for novel control targets for nematode parasites.

## Highlights

• We report the identification of *flp* and *flp*-GPCR gene homologues in parasitic nematodes.
• We provide data to support re-evaluation of the number of *flp*-genes in nematodes.
• Post BLAST phylogenetic analysis facilitates identification of putative *flp*-GPCRs in nematode parasites.
• We expose the most highly conserved *flp* and *flp*-GPCR genes in key pathogens within phylum Nematoda.

## Graphical abstract

**Figure:**
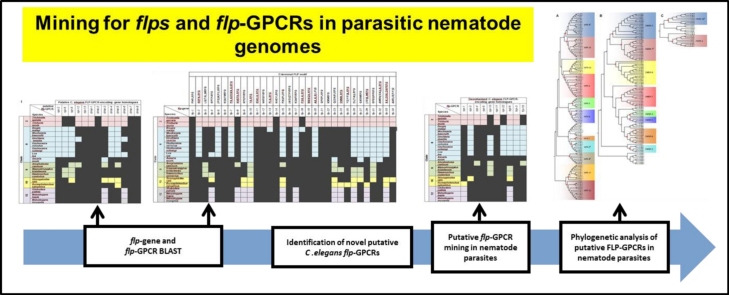

## 1 Introduction

The discovery and development of novel anti-worm control strategies has been recognised as a priority in the human and veterinary health sectors and by the horticultural industry [1–3]. Despite the recent introduction of several new drugs to the veterinary market [4,5] and mass drug administration programmes for prioritised human helminthiases [6], roundworm infections remain widespread with significant socio-economic impacts [7]. In addition, the negative impacts of plant pathogenic nematodes on global food security are underscored by the current deficiencies in chemical control options (see [8] for review).

There has been a long-standing interest in the neuropeptidergic system as a source of novel targets for anthelmintic drugs (see [9] for review), with the FMRFamide like peptide (FLP) signalling system emerging as a leading candidate [10]. The primary drivers for this interest include: (i) the importance of FLPs to parasite behaviour (and survival) and their role in modulating neuromuscular function (a proven drug target for nematode control), (ii) the lack of drugs targeting the FLPergic system such that resistance would not be a pressing concern, and (iii) the fact that most FLPs activate G-protein coupled receptors (GPCRs), proteins which are readily exploitable for drug discovery.

Whilst many facets of the FLP signalling system provide appeal as drug targets, FLP-GPCRs emerge as the most attractive. A major impediment to the exploitation of FLP GPCRs is the lack of data on the expression and function of FLP receptors in nematode parasites. Our current understanding of FLP receptor biology has been derived primarily from the model nematode *C. elegans*; 13 FLP-GPCRs, encoded on 10 genes, have been matched with their associated FLP ligands as determined by receptor activation potencies in heterologous expression systems [11–20].

Whilst focus on the identification of putative FLP-GPCRs in parasitic nematodes is of primary importance, much can be accomplished by re-mining for FLP ligands, especially in therapeutically-important species where they have not previously been reported. Indeed, better understanding of FLP complementarity across parasitic nematodes could expedite deorphanisation or, at least, influence deorphanisation approaches by providing more comprehensive species-specific peptide libraries to feed into screening platforms. The FLP-ligand data that we have for the phylum Nematoda are outdated (see [10,21]). The availability of ten parasitic nematode draft genomes and transcriptome data for over 60 nematode species [22] warrant re-interrogation of these datasets.

Here we report a pan-phylum homology-based BLAST interrogation of *flp* and *flp*-GPCR complements in 17 parasitic nematode species and perform phylogenetic analyses to identify additional putative *flp*-GPCRs. These data: (i) represent the most up to date, comprehensive insight into the *flp* and *flp*-GPCR complement of parasitic nematodes, (ii) support the re-designation of selected *flp*-encoding genes, (iii) expose the most highly conserved *flp*-GPCRs in key pathogenic species and, (iv) reveal putative novel *flp*-GPCRs in *C. elegans* and parasitic nematodes.

## 2 Methodology

### 2.1 Bioinformatics

A reciprocal BLAST (Basic Local Alignment Search Tool) based approach was implemented to identify *flp* gene sequelogues and *flp*-GPCR gene homologues within genomic and transcriptomic datasets of 17 key pathogenic nematodes representing five clades [23] and including plant, animal and human parasites. The parasite species selected for the BLAST analysis were primarily those with a published draft genome (seven species; see Supplementary Table 1). Secondary species-selection criteria were employed to include parasitic nematodes for which genomic and transcriptomic datasets were available and filtered based on their importance to human, animal or plant health or their status as a model parasite (ten species; see Supplementary Table 1). The BLAST analysis was completed between May 2012 and August 2013; the servers employed and databases queried are outlined in Supplementary Table 1. The draft genomes of two *Haemonchus contortus* strains [24,25] were published following the completion of the BLAST analysis in this study. To facilitate accuracy of the data presented, those genes that were not identified within the *H. contortus* databases outlined in Supplementary Table 1 were employed as search strings to query the whole-genome shotgun contigs (wgs) database found on the National Centre for Biotechnology Information (NCBI) BLAST server. Any datasets updated between May 2012 and August 2013 were similarly reinvestigated.

Prepropeptide and protein sequences for previously identified *flp*- [11], *flp*-GPCR [13–17,19,20,26,27], and selected orphan GPCR [28] -encoding genes in *Caenorhabditis elegans* (see Supplementary Tables 2 and 3) were retrieved from the NCBI protein database (www.ncbi.nlm.nih.gov/protein/) and used as search strings in translated nucleotide (tBLASTn) and protein (BLASTp) BLAST analysis of available datasets (see above). Only the largest of the splice variants encoded by any given *C. elegans flp*- or *flp*-GPCR gene were selected as query sequences. Additionally, prepropeptide sequences derived from *flp*-encoding genes not found in *C. elegans* (see [21]) were also used as BLAST search queries; these included FLP-29 (derived from *Ascaris suum* EST data; [21]), FLP-30 and FLP-31 (derived from *Meloidogyne incognita* EST data; [21]). The prepropeptide search string based methodology used in this study deviates from previously published methods (based on concatenated peptide search strings) employed to identify *flp* gene sequelogues within nematode genomes [29,30]. In this study, the prepropeptide approach has been shown to be as sensitive in identifying *flp* gene sequelogues as those methods previously published.

BLAST-generated alignment outputs (high scoring pairs) of the initial BLAST hits, with an expect value ≤1000 (or ≤100, where this was the maximum expect value threshold available), were manually inspected. In efforts to identify putative FLP-encoding genes in the selected nematode species, hits containing conserved FLP motifs [10,11] flanked by mono/dibasic cleavage sites were selected for further analysis. The motifs conserved within parasitic *flp* genes were used to designate initial hits as *C. elegans* gene sequelogues [21].

For *flp*-GPCR primary BLAST analysis, high-scoring return sequences (typically the hits with the smallest expect value and largest bit score) were concatenated into a single sequence to facilitate reciprocation (see [31]). Manually curated predicted protein (FLP-GPCR) sequences were used as search strings in reciprocal protein BLAST (BLASTp) queries against the *C. elegans* non-redundant protein sequence (nr) database on the NCBI BLAST server, using default settings. The top reciprocal BLAST hit was used to designate parasitic *flp*-GPCR genes as predicted *C. elegans* gene homologues.

### 2.2 Post-BLAST sequence analysis

Predicted FLP prepropeptide sequelogues and FLP-GPCR homologues were aligned using the Vector NTI Advance™11 AlignX^®^ multiple sequence alignment tool [32], using default settings. Prepropeptide cleavage sites were identified using a previously described prediction method [21]. Predicted inter-peptide regions from each prepropeptide alignment were removed to provide an unambiguous representation of FLP conservation within sequelogue alignments (see Supplementary data Figure 1). FLP-GPCR transmembrane (TM) domain prediction was performed using HMMTOP 2.1 [33]. Predicted transmembrane domains are indicated on the consensus sequence for each FLP-GPCR alignment (see Supplementary data Figure 2). Multiple sequence alignments were manually examined to identify and resolve errors (such as accidental exon duplication or exclusion) made whilst constructing predicted protein sequences.

### 2.3 Phylogenetic analysis

MEGA 5.1 software [34] was employed to generate all phylogenetic trees. GPCR multiple sequence alignments, were assembled using Clustal W [35] with default parameters. Transmembrane-only pseudosequences (TOPs) were constructed as previously described by Zamanian et al. [36] and aligned with their associated full length predicted protein sequences. TOPs were used to inform manual editing of the GPCR multiple sequence alignments. The N- and C-termini were removed and, conserved motifs and residues within the GPCR transmembrane regions were aligned. Phylogenies were constructed using the Maximum Likelihood method based on the JTT (Jones–Taylor–Thornton) matrix-based model [37]. Initial trees generated for the heuristic searches (subtree pruning and regrafting) were obtained by applying the Neighbour-Joining method [38] to a matrix of pairwise distances estimated using a JTT model. Phylogenetic analysis was limited to GPCRs with >5 transmembrane domains and trees were rooted by an out-group containing a selection of *C. elegans* secretin family GPCRs (LAT-1a, LAT-1b, LAT-2a, PDFR-1a, PDFR-1b and PDFR-1c).

## 3 Results and discussion

### 3.1 *flp*-encoding genes

In this study we identified 325 *C. elegans flp*-gene sequelogues in 17 nematode parasites (see Table 1; Supplementary Figure 1) of which only a proportion had been previously reported [21,29,30,39,40]. We believe that these data represent the most comprehensive insight into the *flp*-gene complements of parasitic nematodes to date. The genome-directed approach employed in this study enables the comparison of *flp* complementarity within and between nematode species representing different clades and lifestyles. Here we highlight the salient points emerging from this study.

#### 3.1.1 Multiple nematode parasites appear to possess a reduced complement of *C. elegans flp*-gene sequelogues

EST-database comparisons [10,21] highlighted the conservation of FLP-encoding genes across the phylum Nematoda; since most *C. elegans flp* genes were represented amongst the parasitic nematode ESTs, these data fuelled the hypothesis that all nematode species possess a *flp* complement similar to that of *C. elegans* (31 *flp*-genes; 1–28, 32–34) [21,41]. Here we show that whilst individual *flp* signatures are conserved in the nematode parasites generally, there is variability with respect to their presence and absence such that the parasitic nematodes possess variable proportions of the *C. elegans flp*-gene complement (see Table 1). Notably, *Ascaris suum* boasts the lion's share of *C. elegans flp*-genes at 84% while clade 2 species (*Trichuris muris* and *Trichinella spiralis*) display a dramatically reduced complement (13%).

Our bioinformatics-based approach is not capable of unequivocally proving the absence of an individual *flp*-gene. Whilst the disparity in *flp*-gene complement could reflect poor genomic/transcriptomic datasets or deficiencies in the ability of our BLAST-based methodology to detect *flp*-gene sequelogues, we believe that it is more likely a true reflection of the variation in *flp*-gene complement across the phylum Nematoda. Indeed, we have validated our *flp*-gene identification approach in multiple *Caenorhabditis* species (*Caenorhabditis japonica*, *Caenorhabditis brenneri*, *Caenorhabditis remanei* and *Caenorhabditis briggsae)* where the *flp*-gene complements matched *C. elegans*. In addition, it is interesting to note that *Trichinella spiralis* displayed a dramatically reduced *flp*-gene complement despite the data being derived from a high-quality (∼35 fold coverage) published genome [42]. That said, we are aware that some of the genomes employed in this study are works in progress (for example, the hookworm species) and we may expect to uncover additional *flp*-genes when their genomes are complete.

Further to this, it should be noted that the predictions made with respect to *flp*-gene complements do not necessarily reflect the FLPs expressed by any given species or lifestage. We cannot predict which of the *flps* identified from genomic data will be expressed or, similarly, in the case of transcriptomic data, whether they will be processed into bioactive peptides. The application of peptidomic-analyses tools to parasitic nematodes will shed light on this (see [39,43]).

#### 3.1.2 *flp*-gene complement appears to broadly map nematode clade division, with some exceptions

It is interesting to note that the pattern of *flp*-complementarity within nematode clades [23] is, for the most part, conserved with identical *flp*-representation in species within clade 2 and within clade 12. With respect to clade 8, *A. suum* appears to boast a larger repertoire of *flp* genes than the filarids, which are largely similar to each other. The validity of these patterns can be confirmed with the progression of clade-specific parasite genome data.

#### 3.1.3 Some *flp*-genes are more highly conserved than others

Sequelogues of *flp-1* and *flp-14* are represented within the genomes of all species examined reinforcing the pan-phylum conversation as suggested by [10]. In contrast *flp-10* sequelogues were not identified in any of species represented in this study. Other highly conserved *flp*-genes include: *flp-6*, *-11*, *-12*, *-16*, *-18*, *-19*, *-21* and *-22*.

#### 3.1.4 *flp-29* and *flp-30* are not discrete *flp*-encoding genes but rather are orthologues of *C. elegans flp-28* and *flp-2* respectively

**Fig. 1:**
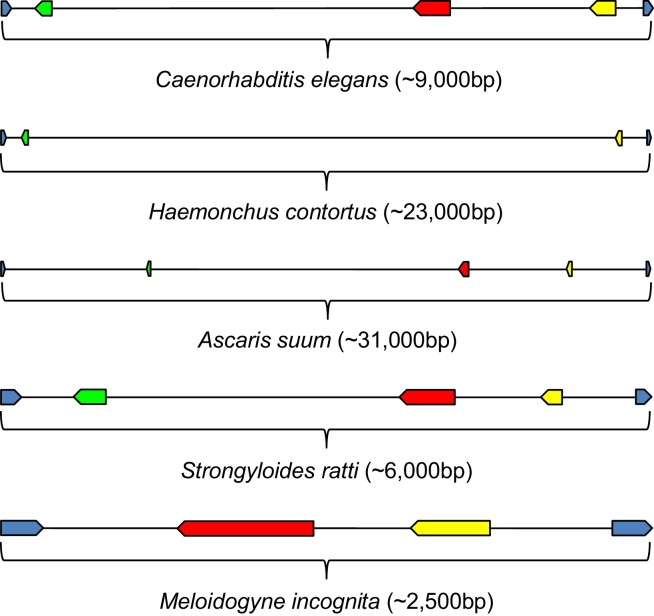
A conserved *flp-28/29*, *flp-3* and *flp-2/30* gene cluster. The location of *flp-29* and/or *flp-30* in the same genomic environment as *flp-28* and *flp-2* respectively provides evidence to support their redesignation. *flp-28/29*, *flp-3* and *flp-2/30* (green, red and yellow arrows respectively) gene sequelogue locations are shown relative to the fourth and fifth exons of a glutamate synthase gene (W07E11.1) in *C. elegans*, and in four parasitic nematode species (blue arrows). The direction of each arrow indicates gene orientation. Introns of *flp* genes are not displayed. One representative species from each clade (excluding clade 2) is included.

*flp-29* and *flp-30* have previously been reported as being parasite-specific (see [10]). During this study we noted the C-terminal motif similarity between *flp-29* (IXMRFG) and *flp-28* (VLMRFG), and *flp-30* (QMREPLRFG) and *flp-2* (EPIRFG) (see Supplementary Figure 1). We also noted that no single species with *flp-29* also had a copy of *flp-28*, and similarly those species possessing *flp-30* did not possess *flp-2*, and vice versa. This prompted further investigation, which revealed that *C. elegans*, which does not possess *flp-29* and *flp-30*, has a cluster of genes composed of *flp-2*, *flp-3* and *flp-28* within the fourth intron of a glutamate synthase gene (W07E11.1). Strikingly, where we identified *flp-29* and/or *flp-30* they were located to the same genomic environment, inhabiting the same relative position and displaying the same relative gene orientation as *flp-28* and *flp-2*, respectively (see Fig. 1). These data support the conclusion that *flp-29* and *flp-30* should be re-designated accordingly. This reduces the total number of known *flp* genes within the phylum Nematoda from 34 to 32 (see Table 1), and further reduces the number of *flp*-genes believed to be parasite specific.

#### 3.1.5 Only one *flp*-gene (*flp-31*) appears to be parasite-specific

*flp-31* is absent from the genome of *C. elegans*, but has previously been reported in the plant parasitic nematodes *Meloidogyne incognita* and *Meloidogyne hapla* [21,30]. In this study we confirm the presence of *flp-31* in another clade 12 plant parasitic nematode (*Globodera pallida*) and identify a *flp-31* sequelogue within the genome of the pine wilt nematode *Bursaphelenchus xylophilus* (clade 10), where it was previously reported as being absent [29]. These data, confirm the restriction of *flp-31* to plant parasitic nematodes and support the hypothesis that it plays a role in phytoparasitism [29,30].

Note that this study did not address the identification of novel *flp*-encoding genes. The ‘degenerate’ search string approach to novel *flp* identification, described by McVeigh et al. [44], was applied to the *T. muris* genome. However, the large number of putative *flp*-gene sequences identified are believed to be false-positives as we could not detect sequelogues in several other nematode genomes. The ‘degenerate’ search string approach, with manual annotation, that was used to identify novel peptides within transcriptomic datasets is not appropriate for trawling large genomic databases.

A BLAST based approach using *C. elegans flp* search strings to identify novel peptides within the expressed sequence tags (EST) and genome survey sequence (GSS) libraries of *A. suum* has recently been reported [39], where nine putative *flp*-encoding genes were identified that were additional to those previously described by McVeigh et al. [21]. Here we confirm the designation of three of these putative *flps* (*As-flp-3*, *As-flp-17*, and *As-flp-19*) in the *A. suum* datasets. We were unable to confirm *As-flp-4*, *As-flp-10a*, *As-flp-10b*, *As-flp-10c*, and *As-flp-25* as named by Jareki et al. [39] as *flp*-encoding genes and, in addition, we did not identify sequelogues of these putative *flps* in any of the nematode genomic/transcriptomic databases employed. Note that distinct *Ce-flp-4* and *Ce-flp-25* sequelogues were identified in *A. suum* and multiple other species in this study (see Table 1 and Supplementary Figure 1). In addition, we believe that the Jarecki et al. [39]-designated *As-flp-31* is more likely to be a sequelogue of *Ce-flp-15* for two reasons: (i) the C-terminal motif of the encoded FLP is identical to that encoded by *Ce-flp-15* (GPLRFG) and (ii) *flp*-31 has only been identified to date in PPN species (see Supplementary Figure 1).

### 3.2 FLP-receptor encoding genes

#### 3.2.1 Deorphanised *C. elegans* FLP-receptor-encoding gene homologues in parasitic nematodes

To date only one *flp*-GPCR orthologue has been reported within a parasitic nematode [40]. In a bid to address the gap in our understanding of *flp*-GPCR conservation in parasitic nematodes, the protein sequences encoded by the ten deorphanised *C. elegans* FLP receptor genes (see Supplementary Table 3) were used as search string queries to probe parasitic nematode databases.

Homologues of all of the deorphanised FLP-activated GPCRs were identified in at least three parasite species. It is interesting to note that the species-specific *flp*-GPCR complement closely maps the *flp* complement, in that those species possessing a restricted repertoire of *flp* genes seem to also exhibit fewer putative FLP-GPCR genes (e.g. the clade 2 species, *T. spiralis* and *T. muris*) and vice versa [*A. suum* possesses both the highest numbers of *flps* and *flp*-GPCRs of all parasitic species examined (see Tables 1–3)].

The most highly conserved, deorphanised FLP-GPCRs are NPR-4, NPR-5 and NPR-11 which have been matched via heterologous expression systems in *C. elegans* with *flp-18*- (NPR-4 and NPR-5) and *flp-21*-peptides (NPR-11; see Table 3). Strikingly *flp-18* and *flp-21* also emerged as two of the most highly conserved *flps* in this study (Table 1). NPR-4 and NPR-5 have been functionally characterised as *flp-18* receptors through null mutant phenotype analysis in *C. elegans* [45] confirming the heterologous expression-derived data. Whether or not this rings true for the parasites remains to be determined. In this study all species that possess NPR-4- and NPR-5-encoding genes almost always possess the gene encoding the predicted interacting ligand (Table 3), supporting pan-phylum conservation of ligand/receptor interactions like those described for a FLP-32 receptor in plant parasitic nematodes (see [40]), and as highlighted by Janssen et al. [46].

There are several issues surrounding the utility and transferability of the heterologous expression data derived from *C. elegans*. For example a number of parasite species exhibit a predicted FLP-GPCR homologue but appear to lack its most potent ligand as deduced from *C. elegans* heterologous expression data, and vice versa. More specifically, with respect to NPR-11, functional data indicate that a neuropeptide-like protein encoding gene (*nlp*-1) encodes the interacting ligands [47], as opposed to *flp-21* that was suggested by heterologous expression. These nuances are potentially the result of a number of issues including: (i) no FLP-GPCR has ever been challenged with the full *C. elegans* neuropeptide complement such that the cognate interacting ligand may have been overlooked; indeed only seven *C. elegans* peptides were screened against NPR-11 in the heterologous system; (ii) heterologous ligand matching may not mimic true *in vivo* interactions; the *flp-21*/NPR-11 *in vitro* interaction is not mirrored by functional data; (iii) the interpretation of null-mutant functional data is limited by the suitability/sensitivity of the available phenotypic assays; the *flp-21*/NPR-11 functional interaction may not have been uncovered for these reasons. Importantly, a cautionary approach must be adopted when applying data derived from *C. elegans* to parasites and highlights the need for deorphanisation efforts in both free-living and parasitic nematodes [10]. The receptor and ligand sequences identified in this study should provide a solid platform on which to base these investigations (Table 4).

**Fig. 2:**
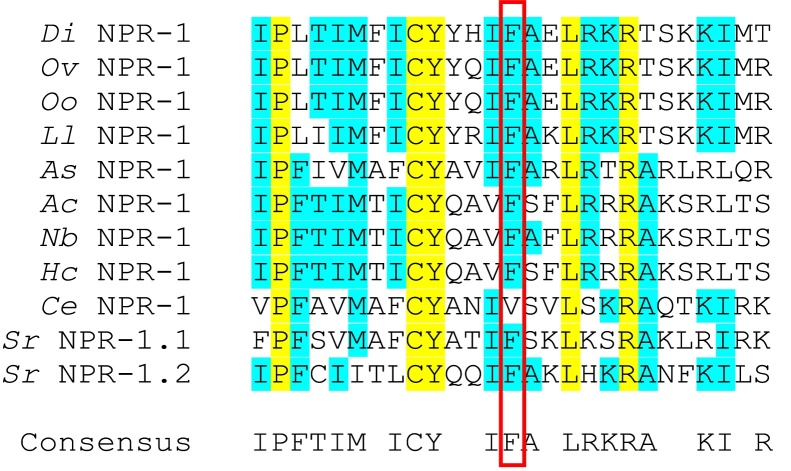
Sequence alignment of NPR-1 homologues. Parasitic nematodes display a conserved phenylalanine in NPR-1 215 in contrast to the dominant amino acid at this position (valine) in *C. elegans.* Position 215 is boxed in red. Completely and partially conserved amino acid residues are highlighted in yellow and blue, respectively. *Di*, denotes *Dirofilaria immitis*; *Ov*, denotes *Onchocerca volvulus*; *Oo*, denotes *Onchocerca ochengi*; *Ll*, denotes *Loa loa*; *As*, denotes *Ascaris suum*; *Ac*, denotes *Ancylostoma caninum*; *Nb*, denotes *Nippostrongylus brasiliensis*; *Hc*, denotes *Haemonchus contortus*; *Ce*, denotes *Caenorhabditis elegans*; *Sr*, denotes *Strongyloides ratti*. All parasitic nematode NPR-1 homologues identified in this study are detailed in Table 2 and Fig. 4.

Two isoforms of the FLP-GPCR NPR-1 exist within natural *C. elegans* populations containing either a valine (V) or phenylalanine (F) at position 215 [48]. The FLP-21 peptide activates the 215F variant, which is found exclusively in social feeding strains. By contrast FLPs encoded on both *flp-21* and *flp-18* activate the 215V strain which is associated with solitary feeding [16,27]. In contrast to *C. elegans*, all of the NPR-1 homologues identified here in parasitic nematodes exhibit the 215F isoform (see Fig. 2). Further investigation is needed to determine if this has any impact on feeding behaviour and/or peptide activation of these receptors.

#### 3.2.2 Novel putative FLP-GPCR identification in *C. elegans*

Until recently markedly fewer FLP-receptors were known that FLP-ligands. However, Frooninckx et al. [28] employed a Multiple Expectation Maximization for Motif Elicitation/Motif Alignment and Search Tool (MEME/MAST) using protein motifs derived from deorphanised neuropeptide GPCRs to identify 128 putative orphan neuropeptide receptors, some of which are likely to be FLP-receptors. These sequences were subdivided into rhodopsin- and secretin-like GPCR families, with rhodopsin-like GPCRs being further divided into six groups according to their similarity to mammalian and insect neuropeptide GPCRs [28].

In this study, we channelled the sequences from the groups delineated by Frooninckx et al. [28] which contained at least one deorphanised FLP receptor into phylogenetic analyses with the aim of identifying putative (currently orphan) FLP-GPCR-encoding genes in *C. elegans*. In addition we included the *Drosophila* Myosupressin like Receptor (DMSR)–like sequences based on their delineation as FLP-activated receptors in arthropods (see [49]).

**Fig. 3:**
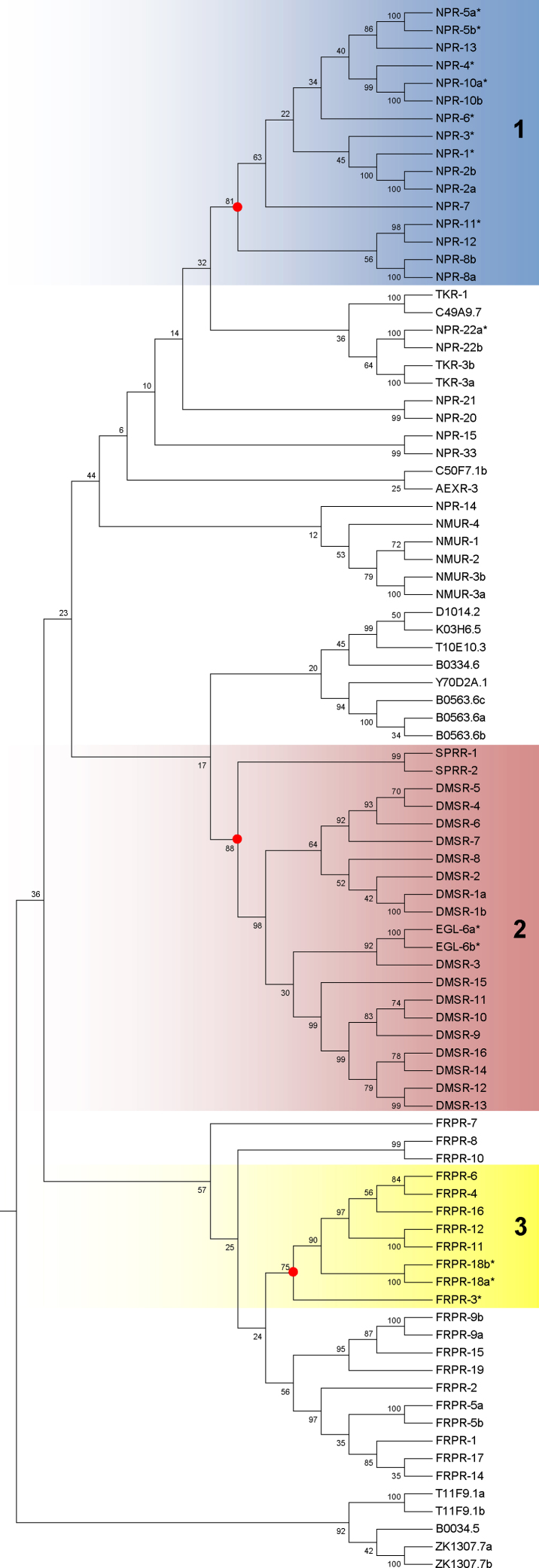
Maximum-likelihood phylogeny of orphan and deorphanised *C. elegans* neuropeptide GPCRs. The phylogeny reveals 27 putative FLP-GPCRs which clustered with deorphanised *C. elegans* FLP-receptors. *C. elegans* neuropeptide GPCR entries are identified as per previous designation [28] or NCBI accession number. Deorphanised *C. elegans* FLP-GPCRs are highlighted by an asterix. FLP-GPCR cluster nodes supported by a bootstrap analysis value of >70% are identified by red dots. Clusters are highlighted in blue (1), pink (2) or yellow (3) shading. Bootstrap values are shown as percentages.

Three clusters composed of 32 putative *Ce*-FLP-GPCRs (encoded on 29 genes) emerged from the phylogenetic analysis defined by the inclusion of at least one deorphanised FLP-GPCR and supported by a bootstrap value of >70% (see Fig. 3; Supplementary Figure 2). Whilst the clustering does not necessarily indicate that closely related receptors will be activated by highly similar ligands, it is interesting to note that NPR-4 and NPR-10 cluster with a bootstrap value of 99% and are both activated by FLP-18 peptides. Also, cluster 1 (NPY-like receptors) displayed the highest complement of deorphanised FLP-GPCRs (eight), which provides an indication that those orphan receptors in this cluster may be activated by FLPs (see [50]). In contrast, NPR-22 (activated by *flp-7* encoded peptides) did not cluster strongly enough with any orphan *C. elegans* GPCRs to permit the designation of these related orphans as putative FLP-receptors.

#### 3.2.3 Novel putative FLP-GPCR complement in parasitic nematodes

**Fig. 4:**
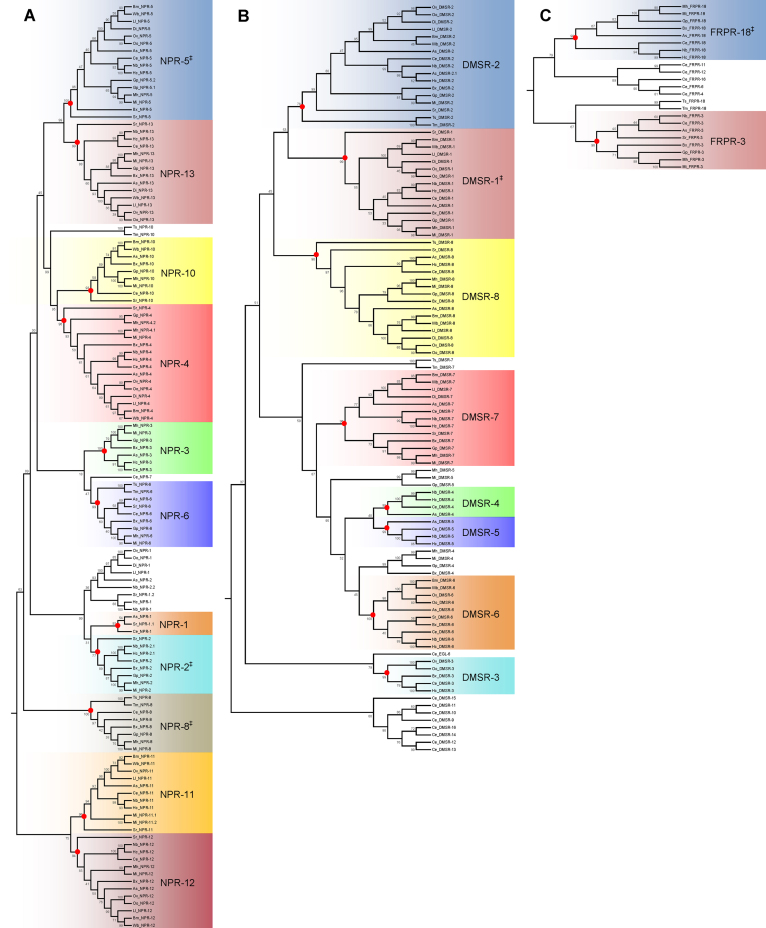
Maximum-likelihood phylogenies of putative FLP-GPCRs from parasitic nematodes. The phylogenies reveal 13 putative FLP-GPCRs that are highly conserved in nematode parasites (see Fig. 3). Entries are identified as per previous designation [28] and prefixes which indicate species: Ts, denotes *Trichinella spiralis*; Tm, denotes *Trichuris muris*; Bm, denotes *Brugia malayi*; Wb, denotes *Wuchereria bancrofti*; Di, denotes *Dirofilaria immitis*; Ov, denotes *Onchocerca volvulus*; Oo, denotes *Onchocerca ochengi*; Ll, denotes *Loa loa*; As, denotes *Ascaris suum*; Ac, denotes *Ancylostoma caninum*; Nb, denotes *Nippostrongylus brasiliensis*; Hc, denotes *Haemonchus contortus*; Ce, denotes *Caenorhabditis elegans*; Sr, denotes *Strongyloides ratti*; Bx, denotes *Bursaphelenchus xylophilus*; Gp, denotes *Globodera pallida*; Mh, denotes *Meloidogyne hapla*; Mi, denotes *Meloidogyne incognita*. Each phylogeny (A-C) represents a single *C. elegans* FLP-GPCR cluster identified in Fig. 3: (A) denotes the phylogeny of parasitic nematode FLP-GPCR homologues of the *C. elegans* predicted proteins present in Fig. 3 cluster 1; (B) denotes the phylogeny of parasitic nematode FLP-GPCR homologues of the *C. elegans* predicted proteins present in Fig. 3 cluster 2; (C) denotes the phylogeny of parasitic nematode FLP-GPCR homologues of the *C. elegans* predicted proteins present in Fig. 3 cluster 3. FLP-GPCR cluster nodes supported by a bootstrap analysis value of >70% are identified by red dots. Clusters are shaded and named according to the relevant *C. elegans* orthologue. Bootstrap values are shown as percentages. ^‡^Note that NPR-2, NPR-5, NPR-8, DMSR-1 and FRPR-18 isoforms were not delineated in the parasite species. In addition only the longest *C. elegans* FLP-/putative FLP-receptor isoforms were included in the phylogenetic analyses such that Ce*_*NPR-2 represents isoform a; Ce*_*NPR-5 represents isoform b; Ce*_*NPR-8 represents isoform b, Ce_DMSR-1 represents isoform a; Ce_EGL-6 represents isoform a; Ce_FRPR-18 represents isoform b.

Predicted orphan *C. elegans* GPCR sequences that clustered with deorphanised *C. elegans* FLP-receptors (see Fig. 3) were used as search string queries in a BLAST approach to identify putative *flp*-GPCR orthologues in parasitic nematodes. Subsequently the predicted FLP-GPCRs (*C. elegans* and parasite species; containing >5 TM domains) were subjected to phylogenetic analysis (Fig. 4). Members of the Sex Peptide related receptor gene family (SPRR-1 and SPRR-2), present within cluster 2 (see Fig. 3), were excluded from this analysis as they are known homologues of a *Drosophila* receptor which is activated by a non-FLP neuropeptide [51]. Homologues of 13 putative *C. elegans flp*-GCPRs were identified in parasitic nematodes. The bootstrap values (>70%) provided by the phylogenetic analysis supported the designation of the majority (90.3%) of these putative parasite *flp*-GPCRs as orthologues, and validated our BLAST-based approach. Approximately 50% of the putative *flp*-GPCR homologues identified in the parasites were derived from *C. elegans* NPY-like receptors (see Fig. 3, cluster 1; Fig. 4A), eight of which have been deorphanised. Therefore, this may strengthen the prediction that these orphan parasite receptors are the most likely to have FLPs as ligands (see [50]) and may provide the primary focus for future deorphanisation efforts.

With respect to broad-spectrum drug target appeal, DMSR-2, -7 and -8 are the most highly conserved putative *C. elegans* FLP-GPCRs found in parasitic nematodes. It will be interesting to see if deorphanisation studies link these to highly conserved *flp*-ligands (e.g. *flp-1* and *flp-14* encoded peptides which remain unmatched with their cognate receptors). It is likely that those putative *C. elegans flp*-GPCRs not identified in the parasites (*npr-7*, *dmsr-9*, *dmsr-10*, *dmsr-11*, *dmsr-12*, *dmsr-13*, *dmsr-14*, *dmsr-15*, *dmsr-16*, *frpr-4*, *frpr-6*, *frpr-11*, *frpr-12*, and *frpr-16*) have evolved via gene duplication following the separation of the *Caenorhabditis* spp. and the parasites from their common ancestor.

Several putative FLP-GPCRs in the parasite species did not fall into orthologous clusters [see Fig. 4; e.g. sequences previously designated as NPR-10, DMSR-7 and FRPR-18 homologues in clade 2 species (*T. spiralis* and *T. muris*), and DMSR-4, and DMSR-5, in the plant parasitic nematodes (*B. xylophilus*, *M. incognita*, *M. hapla*, and *G. pallida*)]. In addition, several putative FLP-GPCRs designated as either NPR-1 or NPR-2 (*Ov*-NPR-1, *Oo*-NPR-1, *Di*-NPR-1, *Ll*-NPR-1, *As*-NPR-2, *Nb*-NPR-2.*2*, *Sr*-NPR-1.*2*, *Hc*-NPR-1 and *Nb*-NPR-1) failed to cluster with either *Ce*-NPR-1 or *Ce*-NPR-2 (see Fig. 5A) and as such constitute two NPR-1/2-like parasite specific clusters. All sequences designated as either NPR-1 or NPR-2 homologues in this study exhibit a phenylalanine at position 215, linked to sociality in *C. elegans*.

## 4 Conclusions

This study provides the first pan-phylum genome-based overview of the FLPergic complement in parasitic nematodes. This encompasses both an update of the FLP ligand profile and reports, for the first time, the putative FLP-GPCR complement beyond *C. elegans*. Our study revises the number of *flp*-genes from 34 to 32, of which only one (*flp-31*) appears to be parasite-specific. This study also reveals that whilst the *flp*-signatures identified in nematode parasites are structurally similar to those described for *C. elegans*, the diversity in the *flp*-complement in the parasites is variably restricted. Indeed, nematode parasites only possess a proportion of the *C. elegans flp*-gene complement and exhibit inter-clade variation where clade-matched species broadly display similar *flp*-gene profiles.

With respect to FLP-GPCRs we have reported the conservation of known deorphanised *C. elegans* FLP-receptors in parasite species and our phylogenetic approach has facilitated the identification of a further 13 putative *flp*-GPCRs in nematode parasites. These data reveal that there may be many more nematode FLP-receptors than previously thought.

This dataset contributes significantly to future FLP-receptor deorphanisation efforts by providing a more comprehensive library of parasite-specific FLP ligands and a catalogue of putative parasite FLP-receptors against which to screen. These data help facilitate the functional characterisation of parasite FLP-GPCRs to reveal those most appealing for exploitation as novel chemotherapeutic targets.

## Transparency document

Transparency document

## Figures and Tables

**Table 1:** *C. elegans flp*-gene sequelogues in 17 nematode parasites. Grey boxes indicate the presence of a *flp* gene sequelogue, as identified via BLAST, in selected nematode species. Search queries employed and the *flp* genes identified are detailed in Supplementary Table 2 and Supplementary Figure 1, respectively. Sequelogues of all *C. elegans flp* genes were also identified in the genomes of *Caenorhabditis japonica*, *Caenorhabditis brenneri*, *Caenorhabditis remanei* and *Caenorhabditis briggsae*. C-terminal FLP motifs are presented in single letter notation; X, denotes variable amino acid X_o_, denotes hydrophobic amino acid; X_i_, denotes hydrophilic amino acid.

**Table 2:** Deorphanised *C. elegans* FLP-GPCR-encoding gene homologues in 17 nematode parasites. Grey boxes indicate the presence of a *flp*-GPCR homologue, as identified via BLAST, in selected nematode species. Search queries employed and *flp*-GPCR predicted proteins identified are detailed in Supplementary Table 3 and Supplementary Figure 2, respectively.

**Table 3:** *C. elegans* FLP-GPCR-encoding gene homologues and *flp*-genes encoding the most potent interacting ligand(s) in 17 nematode parasites. Grey boxes indicate the presence of a *C.elegans flp*-GPCR homologue (highlighted in red) and *flp*-gene sequelogue(s) encoding the most potent ligand in selected nematode species [10,12,17]. The total numbers of *C. elegans* FLPs screened against the *C. elegans flp*-GPCR homologue in heterologous expression systems are also indicated.

**Table 4:** **Putative*****C. elegans*****FLP-GPCR-encoding gene homologues in 17 nematode parasites.** Grey boxes indicate the presence of a *flp*-GPCR homologue, as identified via BLAST, in selected nematode species. Search queries employed and *flp*-GPCR predicted proteins identified are detailed in Supplementary Table 3 and Supplementary Figure 2, respectively. *C. elegans flp*-GPCRs not identified in the parasites included: *npr-7*, *dmsr-9*, *dmsr-10*, *dmsr-11*, *dmsr-12*, *dmsr-13*, *dmsr-14*, *dmsr-15*, *dmsr-16*, *frpr-4*, *frpr-6*, *frpr-11*, *frpr-12* and *frpr-16*.

## Appendix A Supplementary data

Supplementary Fig. 1 **Multiple sequence alignments of FLPs encoded on*****flp*****gene sequelogues identified by BLAST.** Sequences were aligned using the Vector NTI Advance™11, Align X multiple sequence alignment tool, using default settings [32]. Completely and partially conserved amino acid residues are highlighted in yellow and blue, respectively, while blocks of similar residues are highlighted in green. More divergent *flp* genes include the *flp-3* sequelogues present within filarial nematodes. Attempts to identify FLP sequences more similar to those encoded on these genes in other nematodes were unsuccessful. For this reason they have been assigned a name according to their most similar *C. elegans flp* gene sequelogue (*flp-3*). Moreover, filarial *flp-3* genes occupy a similar genomic environment to all other *flp-3* sequelogues (see Figure 2) suggesting that these genes represent *flp-3* orthologues despite encoding a divergent FLP sequence. *T. spiralis*, denotes *Trichinella spiralis*; *T. muris*, denotes *Trichuris muris*; *B. malayi*, denotes *Brugia malayi*; *W. bancrofti*, denotes *Wuchereria bancrofti*; *D. immitis*, denotes *Dirofilaria immitis*; *O. volvulus*, denotes *Onchocerca volvulus*; *O. ochengi*, denotes *Onchocerca ochengi*; *L. loa*, denotes *Loa loa*; *A. suum*, denotes *Ascaris suum*; *A. caninum*, denotes *Ancylostoma caninum*; *N. brasiliensis*, denotes *Nippostrongylus brasiliensis*; *H. contortus*, denotes *Haemonchus contortus*; *C. elegans*, denotes *Caenorhabditis elegans*; *S. ratti*, denotes *Strongyloides ratti*; *B. xylophilus*, denotes *Bursaphelenchus xylophilus*; *G. pallida*, denotes *Globodera pallida*; *M. hapla*, denotes *Meloidogyne hapla*; *M. incognita*, denotes *Meloidogyne incognita*.

Supplementary Fig. 2 **Multiple sequence alignments of the predicted FLP-GPCRs identified by BLAST.** Sequences were aligned using the Vector NTI Advance™11, Align X multiple sequence alignment tool, using default settings [32]. Completely and partially conserved amino acid residues are highlighted in yellow and blue respectively, while blocks of similar residues are highlighted in green. Predicted transmembrane domains are underlined on the consensus sequence for each FLP-GPCR alignment. Outside to inside helices have are identified by a solid line; inside to outside helices are identified by a dotted line. Note that only the longest *C. elegans* FLP-/putative FLP-receptor isoforms were included in the alignment such that *C. elegans* NPR-2 represents isoform a; *C. elegans* NPR-5 represents isoform b; *C. elegans* NPR-8 represents isoform b, *C. elegans* DMSR-1 represents isoform a; *C. elegans* EGL-6 represents isoform a; *C. elegans* FRPR-18 represents isoform b. *T. spiralis*, denotes *Trichinella spiralis*; *T. muris*, denotes *Trichuris muris*; *B. malayi*, denotes *Brugia malayi*; *W. bancrofti*, denotes *Wuchereria bancrofti*; *D. immitis*, denotes *Dirofilaria immitis*; *O. volvulus*, denotes *Onchocerca volvulus*; *O. ochengi*, denotes *Onchocerca ochengi*; *L. loa*, denotes *Loa loa*; *A. suum*, denotes *Ascaris suum*; *A. caninum*, denotes *Ancylostoma caninum*; *N. brasiliensis*, denotes *Nippostrongylus brasiliensis*; *H. contortus*, denotes *Haemonchus contortus*; *C. elegans*, denotes *Caenorhabditis elegans*; *S. ratti*, denotes *Strongyloides ratti*; *B. xylophilus*, denotes *Bursaphelenchus xylophilus*; *G. pallida*, denotes *Globodera pallida*; *M. hapla*, denotes *Meloidogyne hapla*; *M. incognita*, denotes *Meloidogyne incognita*.

Supplementary Table 1 **Nematode Genome and Transcriptome BLAST databases/servers employed.** BI, denotes Broad Institute; 959, denotes 959 Nematode Genomes; SI, denotes Sanger Institute; WB, denotes WormBase; INRA, denotes INRA Genomic Resources; EST, denotes Expressed Sequence Tags; NR/NT, denotes Nucleotide Collection; TSA, denotes Transcriptome Shotgun/RNA-seq Assembly; GSS, denotes Genome Survey Sequences; WS231-8, denotes Wormbase release WS231-8; PP, denotes Predicted Proteins; 454, denotes 454 reads; GS/C, denotes the most recent Genomic Scaffolds/Contigs available; UP, denotes unplaced reads; P, denotes published genome; UOG, denotes unpublished on-going; UIA, denotes unpublished in annotation; GOG, denotes GSS on-going; 454OG, denotes 454 on-going. NCBI: http://www.ncbi.nlm.nih.gov/; WormBase: http://www.wormbase.org/tools/blast_blat; 959 Nematode genomes: http://www.nematodes.org/nematodegenomes/index.php/959_Nematode_Genomes; The Sanger institute: http://www.sanger.ac.uk/resources/software/blast/; The Broad institute: http://www.broadinstitute.org/annotation/genome/filarial_worms/Blast.html; INRA Genomic resources: http://meloidogyne.toulouse.inra.fr/blast/blast.html.

Supplementary Table 2 *flp* gene BLAST query GenBank accession numbers.

Supplementary Table 3 *flp* / putative *flp*-GPCR BLAST query GenBank accession numbers. *denotes deorphanised FLP-GPCR-encoding gene.
