# Supplementary material for: New insights into the FLPergic complements of parasitic nematodes: Informing deorphanisation approaches
Source: EuPA Open Proteom. 2014 Apr 19;3:262–72. doi: 10.1016/j.euprot.2014.04.002 (PMC4405611; doi:10.1016/j.euprot.2014.04.002)
Supplement: Supplementary Fig. 1 — Multiple sequence alignments of FLPs encoded onflpgene sequelogues identified by BLAST. Sequences were aligned using the Vector NTI Advance™11, Align X multiple sequence alignment tool, using default settings [32]. Completely and partially conserved amino acid residues are highlighted in yellow and blue, respectively, while blocks of similar residues are highlighted in green. More divergent flp genes include the flp-3 sequelogues present within filarial nematodes. Attempts to identify FLP sequences more similar to those encoded on these genes in other nematodes were unsuccessful. For this reason they have been assigned a name according to their most similar C. elegans flp gene sequelogue (flp-3). Moreover, filarial flp-3 genes occupy a similar genomic environment to all other flp-3 sequelogues (see Figure 2) suggesting that these genes represent flp-3 orthologues despite encoding a divergent FLP sequence. T. spiralis, denotes Trichinella spiralis; T. muris, denotes Trichuris muris; B. malayi, denotes Brugia malayi; W. bancrofti, denotes Wuchereria bancrofti; D. immitis, denotes Dirofilaria immitis; O. volvulus, denotes Onchocerca volvulus; O. ochengi, denotes Onchocerca ochengi; L. loa, denotes Loa loa; A. suum, denotes Ascaris suum; A. caninum, denotes Ancylostoma caninum; N. brasiliensis, denotes Nippostrongylus brasiliensis; H. contortus, denotes Haemonchus contortus; C. elegans, denotes Caenorhabditis elegans; S. ratti, denotes Strongyloides ratti; B. xylophilus, denotes Bursaphelenchus xylophilus; G. pallida, denotes Globodera pallida; M. hapla, denotes Meloidogyne hapla; M. incognita, denotes Meloidogyne incognita. [file mmc2.pdf]

1. FLP-1 sequelogues

T. spiralis FLP-1	-----------------SVPSFLRFG	-----SVLEPNFIRFG	----------	-------------NSPNFLRFG
T. muris FLP-1	-------------------VNFLRFG	-----KMTNPNFLRFG	----------	-----------APNSPNFLRFG
B. malayi FLP-1	------------------KPNFIRFG	----------------	----------	FLQFGHSSSAFIPSGQNFLRFG
W. bancrofti FLP-1	------------------KPNFIRFG	----------------	----------	FLQFGHSSSAFTPSGQNFLRFG
D. immitis FLP-1	------------------KPNFIRFG	----------------	----------	------SSSIFTPGGQNFLRFG
O. volvulus FLP-1	------------------KPNFIRFG	----------------	----------	------SSSAFTPSGQNFLRFG
O. ochengi FLP-1	------------------KPNFIRFG	----------------	----------	------SSSAFTPSGQNFLRFG
L. loa FLP-1	------------------KPNFIRFG	----------------	----------	FLQFGHSSSAFTPGRQNFLRFG
A. suum FLP-1	------------------KPNFIRFG	------GSDPNFLRFG	TPSNNFLRFG	-----------SNQAQNFLRFG
A. caninum FLP-1	------------------KPNFMRYG	------GSDPNFLRFG	----------	-------------NQPNFLRFG
N. brasiliensis FLP-1	------------------KPNFMRFG	------GADPNFLRFG	----------	-------------NQPSFLRFG
H. contortus FLP-1	------------------KPNFMRFG	------GSDPNFLRFG	-NQPNFLRFG	-------------NQPNFLRFG
C. elegans FLP-1	------------------KPNFMRYG	-----AGSDPNFLRFG	----------	-------------SQPNFLRFG
S. ratti FLP-1	------------------KPNFIRYG	------AADPNFLRFG	SEHQNFLRFG	---------NLGGNNANFLRFG
B. xylophilus FLP-1	----------------GSEPNFLRFG	AAPAPNAAGANFLRFG	----------	-----------SGADPNFLRFG
G. pallida FLP-1	-----------------SDPNFLRFG	-------TSSNFLRFG	----------	-----SSASMSTSSEPNFLRFG
M. hapla FLP-1	-NSLLMSGPWALNSWSEADPNFLRFG	-----AAQSANFLRFG	----------	----------SAPYDPNFLRFG
M. incognita FLP-1	-NSLLMSGPWALNSWSDADPNFLRFG	----AAGQSANFLRFG	----------	----------SAPYDPNFLRFG
Consensus	KPNFIRFG	GSDKPNFlRFG	NNFLRFG	P PNFLRFG

T. spiralis FLP-1	----------	-------AAAANFLRFG	---------	---ASPNFLRFG
T. muris FLP-1	----------	-----------------	---------	------------
B. malayi FLP-1	----------	AAEPNFLHLDPNFLRFG	---------	--SAEPNFLRFG
W. bancrofti FLP-1	----------	EAEPNFLHFDPNFLRFG	---------	--SAEPNFLRFG
D. immitis FLP-1	----------	-------AVEPNFLRFG	---------	--SAEPNFLRFG
O. volvulus FLP-1	AAEPNFLRFG	-----------NFLRFG	---------	--SAEPNFLRFG
O. ochengi FLP-1	AAEPNFLRFG	-----------NFLRFG	---------	--SAEPNFLRFG
L. loa FLP-1	AAEPNFLRFG	----------PNFLRFG	---------	--SAEPNFLRFG
A. suum FLP-1	NAEPNFLRFG	-------PADPNFLRFG	---------	--SAEPNFLRFG
A. caninum FLP-1	----------	---------EPNFLRFG	---------	-ASADPNFLRFG
N. brasiliensis FLP-1	----------	------AAGDPNFLRFG	---------	-AAGDPNFLRFG
H. contortus FLP-1	----------	------AAGDPNFLRFG	---------	-GAGDPNFLRFG
C. elegans FLP-1	----------	------ASGDPNFLRFG	SDPNFLRFG	-AAADPNFLRFG
S. ratti FLP-1	SNSPDFLRFG	------AAGDPNFLRFG	---------	-----PNFLRFG
B. xylophilus FLP-1	----------	-----------------	---------	LDATEPNFLRFG
G. pallida FLP-1	----------	-----------------	---------	-GGVDPTFLRFG

M. hapla FLP-1	----------	-----------------	---------	-GLVDQSYLRFG
M. incognita FLP-1	----------	-----------------	---------	-GLVGQSYLRFG
Consensus	AAEPNFLRFG	DPNFLRFG		SAEPNFLRFG

T. spiralis FLP-1	-----ANPNFLRFG	-------	-------	--AAPNFLRFG
T. muris FLP-1	---LAGNPNFLRFG	-------	-------	--AVGNFLRFG
B. malayi FLP-1	--TEVGDPNFLRFG	-------	-------	---KPNFLRFG
W. bancrofti FLP-1	--TEVGDPNFLRFG	-------	-------	---KPNFLRFG
D. immitis FLP-1	--IEVNEPNFLRLG	-------	-------	---KPNFLRFG
O. volvulus FLP-1	--IEANEPNFLQFG	-------	-------	---KPNFLRFG
O. ochengi FLP-1	--IEANEPNFLQFG	-------	-------	---KPNFLRFG
L. loa FLP-1	--IELRDPNFLRFG	-------	QNFLRFG	-AAEPNFLRFG
A. suum FLP-1	SDIGISEPNFLRFG	NNFLRFG	-------	---KPNFLRFG
A. caninum FLP-1	----AVDPNFLRFG	-------	-------	---KPNFLRFG
N. brasiliensis FLP-1	----AVDPNFLRFG	-------	-------	---KPNFLRFG
H. contortus FLP-1	----GVDPNFLRFG	-------	-------	---KPNFLRFG
C. elegans FLP-1	----SADPNFLRFG	-------	-------	---KPNFLRFG
S. ratti FLP-1	---STTNTNFLRFG	-------	-------	--LQPDFLRFG
B. xylophilus FLP-1	PDPSAMSNNFLRFG	-------	-------	--QSNNFLRFG
G. pallida FLP-1	-----ANNNFLRFG	-------	-------	-QANPNFLRFG
M. hapla FLP-1	--SGAKANNFLRFG	-------	-------	QSNNPNFLRFG
M. incognita FLP-1	SSGGNKGNNFLRFG	-------	-------	QSNNPNFLRFG
Consensus	DPNFLRFG			KPNFLRFG

2. FLP-2 sequelogues

```
          A. suum FLP-2        -FRGEPIRFG   -AQREPIRFG
       A. caninum FLP-2        -FRGEPIRFG   -VPREPIRFG
     H. contortus FLP-2        -FRGEPIRFG   -VPREPIRFG
N. brasiliensis FLP-2          ----EPIRFG   -VPREPIRFG
       C. elegans FLP-2        -LRGEPIRFG   -SPREPIRFG
         S. ratti FLP-2        -FRGEPIRFG   SSFREPIRFG
    B. xylophilus FLP-2        -FRGEPVRFG   -AYREPIRFG
       G. pallida FLP-2/30     PYFREPLRFG   ----------
         M. hapla FLP-2/30     -QMREPLRFG   ----------
     M. incognita FLP-2/30     -QMREPLRFG   ----------

                Consensus       FRGEPIRFG    APREPIRFG
```

3. FLP-3 sequelogues

B. malayi FLP-3	---------	---------	-----------	---------------
W. bancrofti FLP-3	---------	---------	-----------	---------------
D. immitis FLP-3	---------	---------	-----------	---------------
O. volvulus FLP-3	---------	---------	-----------	---------------
O. ochengi FLP-3	---------	---------	-----------	---------------
L. loa FLP-3	---------	---------	-----------	---------------
A. suum FLP-3	---------	TPLGTMRFG	DANPLGTMRFG	---------------
C. elegans FLP-3	SPLGTMRFG	TPLGTMRFG	SAEPFGTMRFG	RNPENDTPFGTMRFG
S. ratti FLP-3	---------	---------	-----------	---------------
B. xylophilus FLP-3	---------	---------	-----------	---------------
M. incognita FLP-3	---------	---------	-----------	---------------
M. hapla FLP-3	---------	---------	-----------	---------------
G. pallida FLP-3	---------	---------	-----------	---------------
Consensus		TPLGTMRFG	A P GTMRFG	

B. malayi FLP-3	-------------	---------	-------------AIHFIRSHLGIMRFG
W. bancrofti FLP-3	-------------	---------	-------------AIHFIRSHLGIMRFG
D. immitis FLP-3	-------------	---------	-------------AIRFIRSHLGIMRFG
O. volvulus FLP-3	-------------	---------	-------------TIRFIRSHLGIMRFG
O. ochengi FLP-3	-------------	---------	-------------TIRFIRSHLGIMRFG
L. loa FLP-3	-------------	---------	-------------AIHFIRSHLGLMRFG
A. suum FLP-3	-------------	---------	------------------NGPLGTMRFG
C. elegans FLP-3	ASEDALFGTMRFG	---------	---------------EDGNAPFGTMKFG
S. ratti FLP-3	-------------	---------	-------------------SPLGTMRFG
B. xylophilus FLP-3	-------------	SPLGTMRFG	-----------------ADGPLGTMRFG
M. incognita FLP-3	-------------	---------	----NAPLLDENVAQLVGESPLGTMRFG
M. hapla FLP-3	-------------	---------	----NAPLLDENAAQIVGESPLGTMRFG
G. pallida FLP-3	-------------	---------	NAPTMLTETDGGGGQSSNGSPLGTMRFG
Consensus			G SPLGTMRFG

B. malayi FLP-3	---------	------------	--------------	---------
W. bancrofti FLP-3	---------	------------	--------------	---------
D. immitis FLP-3	---------	------------	--------------	---------
O. volvulus FLP-3	---------	------------	--------------	---------
O. ochengi FLP-3	---------	------------	--------------	---------
L. loa FLP-3	---------	------------	--------------	---------
A. suum FLP-3	---------	--GDPLGTMRFG	----GGPLGTMRFG	---------
C. elegans FLP-3	---------	EAEEPLGTMRFG	SADDSAPFGTMRFG	NPLGTMRFG
S. ratti FLP-3	---------	------------	-TAKPNPLGTMRFG	---------
B. xylophilus FLP-3	NPLGTMRFG	---NPLGTMRFG	---AEGPLGTMRFG	---------
M. incognita FLP-3	---------	RNTSPLGTMR--	--------------	---------
M. hapla FLP-3	---------	RNSSPLGTMRFG	--------------	---------
G. pallida FLP-3	---------	KFNSPLGTMRFG	-GHNNAPFGTMRFG	---------
Consensus		R SPLGTMRFG	APLGTMRFG	

4. FLP-4 sequelogues

B. malayi FLP-4	--SHGKPTFIRFG	---------	---------
W. bancrofti FLP-4	--SHGKPTFIRFG	---------	---------
D. immitis FLP-4	--SHAKPTFIRFG	---------	---------
O. ochengi FLP-4	--SHGKPTFIRFG	---------	---------
O. volvulus FLP-4	--SHGKPTFIRFG	---------	---------
A. suum FLP-4	---SGKPTFIRFG	---------	---------
A. caninum FLP-4	SSSNGKPTFIRFG	AQPSFIRFG	AQPSFIRFG
N. brasiliensis FLP-4	---SGKPTFIRFG	AQPSFIRFG	AQPSFIRFG
C. elegans FLP-4	-SSNGKPTFIRFG	ASPSFIRFG	---------
B. xylophilus FLP-4	--SNGKPTFIRFG	---------	---------
Consensus	SHGKPTFIRFG	AQPSFIRFG	AQPSFIRFG

5. FLP-5 sequelogues

A. suum FLP-5	---SPKQKFIRFG	--AGSARFIRFG	AGPRFIRFG
A. caninum FLP-5	-----APKFIRFG	--GGGAKFIRFG	-AAKFIRFG
N. brasiliensis FLP-5	-----APKFIRFG	--AGGAKFIRFG	-AAKFIRFG
H. contortus FLP-5	-----APKFIRFG	--AGGAKFIRFG	---------
C. elegans FLP-5	---APKPKFIRFG	---AGAKFIRFG	-GAKFIRFG
S. ratti FLP-5	----AGQKLIRFG	-GG--QKLIRFG	GGQKLIRFG
B. xylophilus FLP-5	---APKAKFIRFG	-AG--QKFIRFG	---------
G. pallida FLP-5	---VPKPKFIRFG	-AG--QKLIRFG	-AQKFIRFG
M. hapla FLP-5	---SPKPKFIRFG	SAGNNQKFIRFG	-AQKFIRFG
M. incognita FLP-5	---SPKPKFIRFG	SAGNNQKFIRFG	-AQKFIRFG
Consensus	SPKPKFIRFG	AG QKFIRFG	AQKFIRFG

6. FLP-6 sequelogues

B. malayi FLP-6	--------	--------	KSSYMRFG	KSAYMRFG
W. bancrofti FLP-6	--------	--------	KSSYMRFG	KSAYMRFG
D. immitis FLP-6	--------	--------	KSSYMRFG	KSAYMRFG
O. ochengi FLP-6	--------	--------	KSSYMRFG	KSAYMRFG
O. volvulus FLP-6	--------	--------	KSSYMRFG	KSAYMRFG
L. loa FLP-6	--------	--------	KSSYMRFG	KSAYMRFG
A. suum FLP-6	--------	KSAYMRFG	KSAYMRFG	KSAYMRFG
A. caninum FLP-6	--------	KSAYMRFG	KSAYMRFG	KSAYMRFG
N. brasiliensis FLP-6	--------	KSAYMRFG	KSAYMRFG	KSAYMRFG
H. contortus FLP-6	--------	KSAYMRFG	KSAYMRFG	KSAYMRFG
C. elegans FLP-6	--------	KSAYMRFG	KSAYMRFG	KSAYMRFG
S. ratti FLP-6	--------	KSAYMRFG	KSAYMRFG	KSAYMRFG
B. xylophilus FLP-6	--------	KSAYMRFG	KSAYMRFG	KSAYMRFG
G. pallida FLP-6	--------	KSAYMRFG	KSAYMRFG	KSAYMRFG
M. hapla FLP-6	KSAYMRLG	KSAYMRFG	KSAYMRFG	KSAYMRFG
M. incognita FLP-6	KSAYMRLG	KSAYMRFG	KSAYMRFG	KSAYMRFG
Consensus	KSAYMRLG	KSAYMRFG	KSAYMRFG	KSAYMRFG

B. malayi FLP-6	KSAYMRFG	--------	--------
W. bancrofti FLP-6	KSAYMR--	--------	--------
D. immitis FLP-6	KSAYMRFG	--------	--------
O. ochengi FLP-6	KSAYMRFG	--------	--------
O. volvulus FLP-6	KSAYMRFG	--------	--------
L. loa FLP-6	KSAYMR--	--------	--------
A. suum FLP-6	KSAYMRFG	KSAYMRFG	--------
A. caninum FLP-6	KSAYMRFG	KSAYMRFG	--------
N. brasiliensis FLP-6	KSAYMRFG	KSAYMRFG	--------
H. contortus FLP-6	KSAYMR--	--------	--------
C. elegans FLP-6	KSAYMRFG	KSAYMRFG	KSAYMRFG
S. ratti FLP-6	KSAYMRFG	KVAALRFG	--------
B. xylophilus FLP-6	KSAYMRFG	--------	--------
G. pallida FLP-6	KSAYMRFG	--------	--------
M. hapla FLP-6	KSAYMR--	--------	--------
M. incognita FLP-6	KSAYMR--	--------	--------
Consensus	KSAYMRFG	KSAYMRFG	

7. FLP-7 sequelogues

A. suum FLP-7	TPMDRSSMVRFG	------------	APMDRISMVRFG	MPIDRSSMVRFG
A. caninum FLP-7	APMDRSSMVRFG	APMDRSSMVRFG	APMDRSSIVRFG	APMDRSSMVRFG
N. brasiliensis FLP-7	ATMDRSSMVRFG	APMDRSTMVRFG	APMDRSTMVRFG	APMDRASMVRFG
H. contortus FLP-7	-----SSIFRFG	APMDRSAMVRFG	APMDRSAMVRFG	APMDRSSMVRFG
C. elegans FLP-7	TPMQRSSMVRFG	SPMQRSSMVRFG	SPMQRSSMVRFG	SPMERSAMVRFG
S. ratti FLP-7	APMDRSSMIKFG	AQLDR-AMVRFG	APLDRSSMVRFG	APLDR-AMVRFG
G. pallida FLP-7	------------	------------	APLDRSAMARFG	APLDRSAIARFG
M. hapla FLP-7	APFDRSALVRFG	APFDRSALVRFG	APLDRAAMVRFG	APFDRSSMVRFG
M. incognita FLP-7	APLDRSALVRFG	APLDRSALVRFG	APLDRAAMVRFG	APFDRSSMVRFG
B. xylophilus FLP-7	APMDRASMVRFG	APMDRASMVRFG	APMDRASMVRFG	APMDRASMVRFG
Consensus	APMDRSSMVRFG	APMDRSAMVRFG	APMDRSSMVRFG	APMDRSSMVRFG

A. suum FLP-7	------------	------------	------------
A. caninum FLP-7	------------	------------	------------
N. brasiliensis FLP-7	------------	------------	------------
H. contortus FLP-7	RAPEAHPLIRFG	------------	------------
C. elegans FLP-7	SPMDRSKMVRFG	SSIDRASMVRLG	TPMQRSSMVRFG
S. ratti FLP-7	-APLDRAMVRFG	-APLDRAMVRFG	APLDR-AMVRFG
G. pallida FLP-7	------------	------------	------------
M. hapla FLP-7	------------	------------	------------
M. incognita FLP-7	------------	------------	------------
B. xylophilus FLP-7	------------	------------	------------
Consensus	APD MVRFG	A AMVR G	PL R AMVRFG

8. FLP-8 sequelogues

T. spiralis FLP-8	ATDKGGKALFVRFG	--------	--------	--------	KNEFVRFG
T. muris FLP-8	AHVLGGKALFVRFG	--------	--------	--------	KNEFVRFG
B. malayi FLP-8	------KNEFIRFG	--------	--------	--------	KNEFIRFG
W. bancrofti FLP-8	------KNEFIRFG	--------	--------	--------	KNEFIRFG
D. immitis FLP-8	------KNEFIRFG	--------	--------	--------	--------
O. volvulus FLP-8	------KNEFIRFG	--------	--------	--------	KNEFIRFG
O. ochengi FLP-8	------KNEFIRFG	--------	--------	--------	KNEFIRFG
L. loa FLP-8	------KNEFIRFG	--------	--------	--------	KNEFIRFG
A. suum FLP-8	------KNEFIRFG	KNEFIRFG	KNEFIRFG	KNEFIRFG	KNEFIRFG
N. brasiliensis FLP-8	------KNEFIRFG	--------	KNEFIRFG	KNEFIRFG	KNEFIRFG
H. contortus FLP-8	------KNEFIRFG	--------	--------	--------	KNEFIRFG
C. elegans FLP-8	------KNEFIRFG	--------	KNEFIRFG	KNEFIRFG	KNEFIRFG
S. ratti FLP-8	------KNEFIRFG	KNEFIRFK	--------	--------	KNEFIRFG
B. xylophilus FLP-8	------KNEFIRFG	--------	--------	--------	KNEFIRFG
Consensus	KNEFIRFG	KNEFIRF	KNEFIRFG	KNEFIRFG	KNEFIRFG

9. FLP-9 sequelogues

N. brasiliensis FLP-9	KPSFVRFG	KPSFVRFG
A. caninum FLP-9	KPSFVRFG	KPSFVRFG
H. contortus FLP-9	KPSFVRFG	KPSFVRFG
C. elegans FLP-9	KPSFVRFG	KPSFVRFG
Consensus	KPSFVRFG	KPSFVRFG

11. FLP-11 sequelogues

B. malayi FLP-11	AIRNALVRFD	--SGIRNALVRFG	------------
W. bancrofti FLP-11	AIRNALVRFD	--SGIRNALVRFG	------------
D. immitis FLP-11	AMRNALIRFG	--SGIRNALIRFG	------------
O. ochengi FLP-11	AMRNALIRFG	--SGIRNALVRFG	------------
O. volvulus FLP-11	AMRNALIRFG	--SGIRNALVRFG	------------
L. loa FLP-11	AIRNALVRFG	--SGIRNALVRFG	------------
A. suum FLP-11	AMRNALVRFG	--SGMRNALVRFG	-NGAPQPFVRFG
A. caninum FLP-11	AMRNALVRFG	AGGGMRNALVRFG	-NGAPQPFVRFG
N. brasiliensis FLP-11	AMRNALVRFG	AGGGMRNALVRFG	-NGAPQPFVRFG
H. contortus FLP-11	AMRNALVRFG	AGGSMRNALVRFG	-NGAPQPFVRFG
C. elegans FLP-11	AMRNALVRFG	ASGGMRNALVRFG	-NGAPQPFVRFG
S. ratti FLP-11	AMRNALVRFG	--AGMRNALVRFG	-NAAPQPFVRFG
B. xylophilus FLP-11	SMRNALVRFG	--SGMRNALVRFG	AQSAPEPFVRFG
G. pallida FLP-11	----------	--STMRNALVRFG	--NPPQPFVRFG
M. hapla FLP-11	TMRNALIRFG	-SATLRSAMVRFG	-TSAPQPFVRFG
M. incognita FLP-11	TMRNALIRFG	-SATLRSAMVRFG	-NSAPQPFVRFG
Consensus	AMRNALVRFG	SGMRNALVRFG	NGAPQPFVRFG

12. FLP-12 sequelogues

W. bancrofti	FLP-12	-RNKFEFIRFG
D. immitis	FLP-12	-RNKFEFIRFG
O. volvulus	FLP-12	-RNKFEFIRFG
O. ochengi	FLP-12	-RNKFEFIRFG
L. loa	FLP-12	-RNKFEFIRFG
A. suum	FLP-12	-RNKFEFIRFG
A. caninum	FLP-12	-RNKFEFIRFG
N. brasiliensis	FLP-12	-RNKFEFIRFG
H. contortus	FLP-12	-RNKFEFIRFG
C. elegans	FLP-12	-RNKFEFIRFG
S. ratti	FLP-12	-RNKFEFIRFG
B. xylophilus	FLP-12	-RNKFEFIRFG
G. pallida	FLP-12	-KNKFEFIRFG
M. hapla	FLP-12	KNNKFEFIRFG
M. incognita	FLP-12	KNNKFEFIRFG
	Consensus	RNKFEFIRFG

13. FLP-13 sequelogues

W. bancrofti FLP-13	--------------	-----------	NDAFQTSPLIRFG	-----------
D. immitis FLP-13	--------------	-----------	NEAFQTSPLIRFG	-----------
O. volvulus FLP-13	--------------	-----------	TEAFQTSPLIRFG	-----------
O. ochengi FLP-13	--------------	-----------	TEAFQTSPLIRFG	-----------
L. loa FLP-13	--------------	-----------	--AFQTSPLIRFG	-----------
A. suum FLP-13	--DSKLMDPLIRFG	-----------	-AEGLSSPLIRFG	-----------
A. caninum FLP-13	--------------	-----------	-------------	-----------
N. brasiliensis FLP-13	DSFDESGSPLMGFG	-DPGAPLIRFG	--APEAQPLIRFG	TPEGAPLIRFG
H. contortus FLP-13	-SFEENASPLIRFG	DLSGAPLIRFG	--APEAHPLIRFG	APDSAPLIRFG
C. elegans FLP-13	----AMDSPLIRFG	AADGAPLIRFG	--APEASPFIRFG	AADGAPLIRFG
S. ratti FLP-13	-----SSSPLVRFG	-----------	-ASMIDSPLVRFG	-SPSGPLVRFG
B. xylophilus FLP-13	----AYAGPLIRFG	-----------	-NSPITDPLIRFG	-----------
B. xylophilus FLP-13.2	--------------	-----------	-------------	-----------
G. pallida FLP-13	--------------	TPSVEPIIRFG	-------------	-----------
M. hapla FLP-13	--------------	-APTAPIIRFG	-------------	-----------
M. incognita FLP-13	--------------	-APTAPIIRFG	-------------	-----------
Consensus	ASPLIRFG	APGAPIIRFG	A TSPLIRFG	APDSAPLIRFG

W. bancrofti FLP-13	-------------	------------	-----------	-----------	-----------
D. immitis FLP-13	-------------	------------	-----------	-----------	-----------
O. volvulus FLP-13	-------------	------------	-----------	-----------	-----------
O. ochengi FLP-13	-------------	------------	-----------	-----------	-----------
L. loa FLP-13	-------------	------------	-----------	-----------	-----------
A. suum FLP-13	-------------	------------	IVTDETVLRFG	-----------	-----------
A. caninum FLP-13	SYDETAGPLIRFG	-DMEGAPLIRFG	-----------	-----------	-----------
N. brasiliensis FLP-13	--DAEASPLIRFG	----SAPLIRFG	--SAAPLVRFG	SPEAAPLLRFG	SPEASPLIRFG
H. contortus FLP-13	--DPEASPLIRFG	--SPAAPLIRFG	SPNASPLIRFG	-----------	-----------
C. elegans FLP-13	--APEASPFIRFG	-ASPSAPLIRFG	SPSAVPLIRFG	-SAAAPLIRFG	-ASSAPLIRFG
S. ratti FLP-13	---SPAVPLVRFG	--SLSGPLVRFG	-SGMSPLIRFG	-ASAGPLVRFG	-----------
B. xylophilus FLP-13	---PDHSPLIRFG	AAFRSAPHIRFG	-----------	-----------	-----------
B. xylophilus FLP-13.2	-------------	------------	---SNPLIRFG	----SPLVRFG	-----------
G. pallida FLP-13	---STVVPLIRFG	PAERAAPLIRFG	TANAVPLIRFG	-----------	-----------
M. hapla FLP-13	-------------	-SFNSAPLIRFG	-LNNAPLIRFG	-----------	-----------
M. incognita FLP-13	-------------	-SPNSAPLIRFG	-LNNAPLIRFG	-----------	-----------
Consensus	EASPLIRFG	A SAPLIRFG	AAPLIRFG	AAAAPLVRFG	AAPLIRFG

14. FLP-14 sequelogues

T. spirallis FLP-14	--------	KHEYLRFG	KHDYLRFG---	--------
T. muris FLP-14	KHEYLRFG	KHEYLRFG	KHEYLRFG---	--------
B. malayi FLP-14	KHEYLRFG	KHEYLRFG	KHEYLRFG---	--------
W. bancrofti FLP-14	KHEYLRFG	KHEYLRFG	KHEYLRFG---	--------
D. immitis FLP-14	KHEYLRFG	KHEYLRFG	KHEYLRFG---	--------
O. volvulus FLP-14	KHEYLRFG	KHEYLRFG	KHEYIRFG---	--------
O. ochengi FLP-14	KHEYLRFG	KHEYLRFG	KHEYIRFG---	--------
L. loa FLP-14	KHEYLRFG	KHEYLRFG	KHEYLRFG---	--------
A. suum FLP-14	KHEYLRFG	KHEYLRFG	KHEYLRFG---	--------
A. caninum FLP-14	KHEYLRFG	KHEYLRFG	KHEYLRFG---	KHEYLRFG
N. brasiliensis FLP-14	KHEYLRFG	KHEYLRFG	KHEYLRFG---	KHEYLRFG
H. contortus FLP-14	KHEYLRFG	KHEYLRFG	KHEYLRFSRFG	KHEYLRFG
C. elegans FLP-14	KHEYLRFG	KHEYLRFG	KHEYLRFG---	KHEYLRFG
S. ratti FLP-14	KHEYLRFG	KHEYLRFG	KHEYLRFG---	--------
B. xylophilUs FLP-14	--------	KHEYLRFG	KHEYLRFG---	--------
G. pallida FLP-14	--------	KHEYLRFG	KHEYLRFG---	--------
M. hapla FLP-14	--------	KHEYLRFG	KHEFVRFG---	--------
M. incognita FLP-14	--------	KHEYLRFG	KHEFVRFG---	--------
Consensus	KHEYLRFG	KHEYLRFG	KHEYLRFG	KHEYLRFG

15. FLP-15 sequelogues

A. suum FLP-15	GRPRGPLRFG	-----------
N. brasiliensis FLP-15	AGPQGPLRFG	RGGPSGPLRFG
H. contortus FLP-15	AGPQGPLRFG	-RGPSGPLRFG
C. elegans FLP-15	GGPQGPLRFG	-RGPSGPLRFG
Consensus	AGPQGPLRFG	RGPSGPLRFG

16. FLP-16 sequelogues

B. malayi FLP-16	--------	GQTFVRFG	--------
W. bancrofti FLP-16	--------	GQTFVRFG	--------
D. immitis FLP-16	--------	GQTFVRFG	--------
O. volvulus FLP-16	--------	GQTFVRFG	--------
O. ochengi FLP-16	--------	GQTFVRFG	--------
L. loa FLP-16	--------	GQTFVRFG	--------
A. suum FLP-16	--------	AQTFVRFG	AQTFVRFG
A. caninum FLP-16	AQTFVRFG	AQTFVRFG	AQTFVRFG
N. brasiliensis FLP-16	AQTFVRFG	AQTFVRFG	AQTFVRFG
H. contortus FLP-16	AQTFVRFG	AQTFVRFG	GQTFVRFG
C. elegans FLP-16	AQTFVRFG	AQTFVRFG	AQTFVRFG
S. ratti FLP-16	AQTFVRFG	AQTFVRFG	AQTFVRFG
B. xylophilus FLP-16	AQTFVRFG	AQTFVRFG	AQTFVRLG
G. pallida FLP-16	AQTFVRFG	AQTFVRFG	GQTFVRFG
M. hapla FLP-16	AQTFVRFG	AQTFVRFG	GQTFVRFG
M. incognita FLP-16	AQTFVRFG	AQTFVRFG	AQTFVRFG
Consensus	AQTFVRFG	AQTFVRFG	AQTFVRFG

17. FLP-17 sequelogues

A. suum FLP-17	KSAFVRFG	KSAFVRFG	KSSYIRFG
A. caninum FLP-17	KSAFVRFG	KSAFVRFG	KSQYIRFG
H. contortus FLP-17	KSAFVRFG	KSAFVRFG	KSQYIRFG
N. brasiliensis FLP-17	KSAFVRFG	KSAFVRFG	KSQYIRFG
C. elegans FLP-17	KSAFVRFG	KSAFVRFG	KSQYIRFG
S. ratti FLP-17	KSAFVRFG	KSAFVRFG	KSSYVRFG
B. xylophilus FLP-17	KSAFVRFG	--------	KSSYIRFG
Consensus	KSAFVRFG	KSAFVRFG	KSQYIRFG

18. FLP-18 sequelogues

T. spiralis FLP-18	----------------	---------	-------------	-YDAPGLMRFG
T. muris FLP-18	----------------	---------	-------------	----PGLMRFG
W. bancrofti FLP-18	SQLIDYGDIPGVLRFG	---------	---DVPG-VLRFG	EGDIPGVLRFG
D. immitis FLP-18	-----------LFRLG	---------	---DVPG-VLRFG	EGDIPGVLRFG
O. volvulus FLP-18	----------DLFRFG	---------	---AVPG-VLRFG	--EIPGVLRFG
O. ochengi FLP-18	----------DLFRFG	---------	---AVPGGVLRFG	--EIPGVLRFG
L. loa FLP-18	----DYGDIPGVLRFG	---------	---DIPG-VLRFG	EGDIPGVLRFG
A. suum FLP-18	-GFGDEMSMPGVLRFG	GMPGVLRFG	---AVPG-VLRFG	-GDVPGVLRFG
A. caninum FLP-18	---DFDNGMPGVLRFG	EVPGVFRFG	---SVPG-VLRFG	--SVPGVLRFG
N. brasiliensis FLP-18	---DPEGGMPGVLRFG	EVPGVLRFG	---SMPG-VLRFG	--GVPGVLRFG
H. contortus FLP-18	---XLVGGMPGVLRFG	EVPGVLRFG	---CMPG-VLRFG	--SVPGVLRFG
C. elegans FLP-18	-------EMPGVLRFG	SVPGVLRFG	---SVPG-VLRFG	--EIPGVLRFG
S. ratti FLP-18	-------EMPGVLRFG	AVPGVLRFG	---GVPG-LLRFG	-DDMPGLLRFG
B. xylophilus FLP-18	----DDQAMPGVLRFG	EMPGVLRFG	---AVPG-VLRFG	-SDMPGVLRFG
G. pallida FLP-18	----------------	---------	DEFVAPG-VLRFG	---MPGVLRFG
M. hapla FLP-18	----------------	---------	DDFIAPG-VLRFG	---MPGVLRFG
M. incognita FLP-18	----------------	---------	DDFIAPG-VLRFG	---MPGVLRFG
Consensus	MPGVLRFG	EVPGVLRFG	VPG VLRFG	DIPGVLRFG

T. spiralis FLP-18	------------	---YDDSAPGLMRFG	----------	----------
T. muris FLP-18	------------	---------------	----------	----------
W. bancrofti FLP-18	-NDDIPGVLRFG	---------------	----------	----------
D. immitis FLP-18	-NDDIPGVLRFG	------SVPGVLRFG	----------	----------
O. volvulus FLP-18	-SEDVPGVLRFG	------SEPGVLRFG	----------	----------
O. ochengi FLP-18	-SEDVPGVLRFG	------SEPGVLRFG	----------	----------
L. loa FLP-18	-NDDIPGVLRFG	------SVPGVLRFG	----------	----------
A. suum FLP-18	--SDMPGVLRFG	------SMPGVLRFG	----------	----------
A. caninum FLP-18	---EMPGVLRFG	------STPGVLRFG	HDIPGVMRFG	-NVPGVLRFG
N. brasiliensis FLP-18	---EMPGVLRFG	------AVPGVLRFG	HEIPGVMRFG	-NVPGVLRFG
H. contortus FLP-18	---EMPGVLRFG	------AMPGVLRFG	TEIPGLMRFG	-NVPGVLRF-
C. elegans FLP-18	------------	-----SEVPGVLRFG	-DVPGVLRFG	-SVPGVLRFG
S. ratti FLP-18	--DQIPGLLRFG	-----GDMPGVLRFG	EQIPGLLRFG	-DMPGLLRFG
B. xylophilus FLP-18	DGAEMPGVLRFG	-----SEMPGVLRFG	SDMPGVLRFG	GDMPGVLRFG
G. pallida FLP-18	---AVPGVLRFG	-----AEVPGVLRFG	--MPQVLRFG	----------
M. hapla FLP-18	---AVPGVLRFG	QAQESGAVPGVLRFG	--MPQVLRFG	----------
M. incognita FLP-18	---AVPGVLRFG	QSQESGAVPGVLRFG	--MPQVLRFG	----------
Consensus	DMPGVLRFG	SVPGVLRFG	DIPGVLRFG	NVPGVLRFG

19. FLP-19 sequelogues

B. malayi	FLP-19	---WASQLRFG	-ANWASKVRFG
W. bancrofti	FLP-19	---WASQLRFG	-TNWASKVRFG
D. imitis	FLP-19	---WASQLRFG	-ANWASKVRFG
O. volvulus	FLP-19	---WASQLRFG	-VNWASKVRFG
O. ochengi	FLP-19	---WASQLRFG	-VNWASKVRFG
L. loa	FLP-19	---WASQLRLG	-ANWASKVRFG
A. suum	FLP-19	---WASQLRLG	-ASWASKVRFG
A. caninum	FLP-19	AETMGGQVRFG	-ASWASSVRFG
H. contortus	FLP-19	---WANQVRFG	ASSWASSIRFG
N. brasiliensis	FLP-19	---WANQVRFG	ASSWASSVRFG
C. elegans	FLP-19	---WANQVRFG	-ASWASSVRFG
S. ratti	FLP-19	---WASQLRYG	-SSWASQLRYG
B. xylophilus	FLP-19	---WASSLRFG	GPGWASQVRFG
G. pallida	FLP-19	---WATQLRFG	--QWASQVRFG
M. hapla	FLP-19	---WSTQLRYG	-SPWSSQVR--
M. incognita	FLP-19	---WSTQLRYG	-SPWSSQVRFG
	Consensus	WASQLRFG	A WAS VRFG

20. FLP-20 sequelogues

A. suum FLP-20	AVLRLG	SLMRLG	GIMRLG	APMRLG
A. caninum FLP-20	AMMRLG	------	AMMRLG	------
N. brasiliensis FLP-20	AMMRLG	------	AIMRLG	------
H. contortus FLP-20	AMMRLG	------	AIMRLG	------
C. elegans FLP-20	------	AMMRFG	AMMRFG	SVFRLG
S. ratti FLP-20	------	ALVRLG	AYVRLG	------
B. xylophilus FLP-20	------	AYMRLG	AYMRLG	------
G. pallida FLP-20	------	------	ALMRLG	------
M. hapla FLP-20	------	AIMRLG	ALMRLG	------
M. incognita FLP-20	------	AIMRLG	ALMRLG	------
Consensus	AMMRLG	AIMRLG	AIMRLG	A RLG

21. FLP-21 sequelogues

B. malayi	FLP-21	-ALGPRPLRFG
W. bancrofti	FLP-21	-ALGPRPLRFG
D. immitis	FLP-21	-ARGPRPLRFG
O. volvulus	FLP-21	-APGPRPLRFG
O. ochengi	FLP-21	-APGPRPLRFG
L. loa	FLP-21	-ALGPRPLRFG
A. suum	FLP-21	-GLGPRPLRFG
A. caninum	FLP-21	-GLGPRPLRFG
N. brasiliensis	FLP-21	-GLGPRPLRFG
H. contortus	FLP-21	-GLGPRPLRFG
C. elegans	FLP-21	-GLGPRPLRFG
S. ratti	FLP-21	-AVGPRPLRFG
B. xylophilus	FLP-21	-GMGPRPLRFG
G. pallida	FLP-21	GSLGPRPLRFG
M. hapla	FLP-21	GSLGPRPLRFG
M. incognita	FLP-21	GSLGPRPLRFG
	Consensus	ALGPRPLRFG

22. FLP-22 sequelogues

B. malayi FLP-22	---VPNAKWMRFG	-LPNAKWMRFG	-AQTAKWMRFG
W. bancrofti FLP-22	TLNVPNAKWMRFG	-LPNAKWMRFG	-AQTAKWMRFG
D. immitis FLP-22	---TPNIKWMRFG	-LPNTKWMRFG	-VPTAKWMRFG
O. volvulus FLP-22	---TVNTKWMRFG	-LPNTKWMRFG	-AQTTKWMRFG
O. ochengi FLP-22	---TVNTKWMRFG	-LPNTKWMRFG	-AQTTKWMRFG
L. loa FLP-22	---TGNVKWMRLG	-----------	-AQNVKWMKFG
A. suum FLP-22	---ASNMKWMRFG	-SPNVKWMRFG	-APNMKWMRFG
A. caninum FLP-22	---TPSAKWMRFG	-SPNAKWMRFG	-SPEAKWMRFG
N. brasiliensis FLP-22	---TPSAKWMRFG	-SPNAKWMRFG	-TPDAKWMRFG
H. contortus FLP-22	---TPSAKWMRFG	-SPNAKWMRFG	-TPDAKWMRFG
C. elegans FLP-22	---SPSAKWMRFG	-SPSAKWMRFG	-SPSAKWMRFG
S. ratti FLP-22	---APNVKWMRFG	-----------	-SPQVKWMRFG
B. xylophilus FLP-22	---APAMKWMRFG	-SPQGKWMRFG	APQ-GKWMRFG
G. pallida FLP-22	-QPAGGVKWMRFG	-----------	TPQ-GKWMRFG
M. hapla FLP-22	---ENGVKWMRFG	VPQQSKWMRFG	APSSGKWMRFG
M. incognita FLP-22	---ENGVKWMRFG	VPQQSKWMRFG	APSSGKWMRFG
Consensus	TPNVKWMRFG	PNAKWMRFG	A TAKWMRFG

23. FLP-23 sequelogues

W. bancrofti	FLP-23	------QDFLRFG
D. immitis	FLP-23	-----VQNFLRFG
O. volvulus	FLP-23	-----VQNFLRFG
O. ochengi	FLP-23	-----KQDFLRFG
C. elegans	FLP-23	--VVGQQDFLRFG
H. contortus	FLP-23	-----LQDFLRFG
	Consensus	MQDFLRFG

24. FLP-24 sequelogues

B. malayi FLP-24	VPNPADMMIRFG
W. bancrofti FLP-24	VPNPADMMIRFG
D. immitis FLP-24	VPSPADMMIRFG
O. volvulus FLP-24	VPSAADMMIRFG
O. ochengi FLP-24	VPSAADMMIRFG
L. loa FLP-24	VPSAADMMIRFG
A. suum FLP-24	VPSAADMMIRFG
A. caninum FLP-24	VPSAGDMMVRFG
H. contortus FLP-24	VPSAGDMMVRFG
C. elegans FLP-24	VPSAGDMMVRFG
S. ratti FLP-24	APNKADMMIRFG
Consensus	VPSAADMMIRFG

25. FLP-25 sequelogues

B. malayi FLP-25	---------TNYDFIRFG	---KDGPGTYDYIRFG
W. bancrofti FLP-25	---------TNYDFIRFG	----DGPETYDYIRFG
D. immitis FLP-25	--------VPNYDFVRFG	-----NDPAYDFIRFG
O. volvulus FLP-25	--------TRDYDFIRFG	---SGQDNKYDYIRFG
O. ochengi FLP-25	--------TRDYDFIRFG	---SGQDNEYDYIRFG
L. loa FLP-25	---------TNYDFIRFG	------PATYDYIRFG
A. suum FLP-25	---------ADYDFIRFG	----KGDNSYDYIRFG
A. caninum FLP-25	----------NYDFVRFG	-------ASYDYIRFG
N. brasiliensis FLP-25	----------HYDFVRFG	------AASYDYIRFG
H. contortus FLP-25	----------HYDFVRFG	-------ASYDYIRFG
C. elegans FLP-25	----------DYDFVRFG	-------ASYDYIRFG
S. ratti FLP-25	SESQIPEGFSYDFVRFG	--GVTESNNYDFVRFG
G. pallida FLP-25	----------AYDYIRFG	NSEHSAGSTYDYIRFG
M. incognita FLP-25	-----SS-SSYDFVRFG	-SNGNNGNTYDYIRFG
M. hapla FLP-25	-----SSPSSYDFVRFG	-SNGNNGNTYDYIRFG
Consensus	YDFVRFG	TYDYIRFG

26. FLP-26 sequelogues

```
   A. suum FLP-26   RIDINDLALRFG   SSYSFDPSNLNLRFG   ------TFVIPTDLALRFG
A. caninum FLP-26   ------------   ---EFNADDLTLRFG   ---GGEIAFHPNDLALRFG
C. elegans FLP-26   ------------   ---EFNADDLTLRFG   GGAGEPLAFSPDMLSLRFG

        Consensus                     EFNADDLTLRFG      G  IAF P DLALRFG
```

27. FLP-27 sequelogues

A. caninum FLP-27	TMGGRMRFG
C. elegans FLP-27	GLGGRMRFG
B. xylophilus FLP-27	FKGGRMRFG
G. pallida FLP-27	SKGSRMRFG
M. hapla FLP-27	AKGSRMRFG
M. incognita FLP-27	AKGSRMRFG
Consensus	AKGSRMRFG

28. FLP-28 sequelogues

A. suum FLP-28/29	--------ILMRFG
A. caninum FLP-28/29	--------ILMRFG
N. brasiliensis FLP-28/29	--------ILMRFG
H. contortus FLP-28/29	--------IFMRFG
C. elegans FLP-28/29	--------VLMRFG
S. ratti FLP-28/29	--------VMMRFG
Consensus	ILMRFG

31. FLP-31 sequelogues

B. xylophilus FLP-31	-GRPRGPPRFG
G. pallida FLP-31	LYRPRGPPRFG
M. hapla FLP-31	LYRPRGPPRFG
M. incognita FLP-31	LYRPRGPPRFG
Consensus	LYRPRGPPRFG

32. FLP-32 sequelogues

A. caninum FLP-32	AMRNSLVRFG
N. brasiliensis FLP-32	AMRNSLVRFG
C. elegans FLP-32	AMRNSLVRFG
S. ratti FLP-32	AMRNSLIRFG
B. xylophilus FLP-32	AMRNSLVRFG
G. pallida FLP-32	AMRNALVRFG
M. hapla FLP-32	AMRNSLVRFG
M. incognita FLP-32	AMRNSLVRFG
Consensus	AMRNSLVRFG

33. FLP-33 sequelogues

A. suum FLP-33	SLDGLQKPRFG
A. caninum FLP-33	SIDGIQKPRFG
N. brasiliensis FLP-33	SIDGIQKPRFG
H. contortus FLP-33	SIDEIQKPR--
C. elegans FLP-33	TIDGIQKPRFG
Consensus	SIDGIQKPRFG

34. FLP-34 sequelogues

```
       B. malayi FLP-34   -------VASAINGALRLRYG   --------------------------
    W. bancrofti FLP-34   ---DLEQFASAINGALRLRYG   --------------------------
      D. immitis FLP-34   ---DLGQFTSAINGALRLRYG   ------QAVIDALVKAINEAEPLRYG
     O. volvulus FLP-34   ---DLEQFTSAINGALRLRYG   -----QLPVADRVIASLNGAERLRFG
      O. ochengi FLP-34   ---DLEQFTSAINGALRLRYG   -----QLPLADRVIASLNGAERLR--
          L. loa FLP-34   ---ALEQIASAINGALRLRYG   --------------------------
         A. suum FLP-34   SAHDLEGFASALNSASRLRYG   --------------------------
      A. caninum FLP-34   --SDMSEFTSAINGASRLRYG   ------APLTNKLIQSLNGAERLRFG
 N. brasiliensis FLP-34   --SDMSEFASAINGASRLRYG   ------APLTNKLIESLNGAERLRFG
    H. contortus FLP-34   --SDLSDFASAINSAGRLRYG   ------APITSKLIQSLNEAERLRFG
      C. elegans FLP-34   --ADISTFASAINNAGRLRYG   ------ALNRDSLVASLNNAERLRFG
   B. xylophilus FLP-34   --SNLNEFMSAMKGAPRLRYG   ----MAAQNLPELIDTLNGAERLR--
      G. pallida FLP-34   -SDGLSDFVGSLNGAARLRYG   -ASNYAEALPAGLLDQLNGAERLRFG
        M. hapla FLP-34   --ADVRDFIGSLNGASRLRYG   ASNNYAQALPAGLLDQLNGAERLRFG
    M. incognita FLP-34   --ADVRDFIGSINSASRLRYG   APSSYAEALPAGLLDQLNGAERLRFG

           Consensus         DL  F SAINGA RLRYG           L   LI SLNGAERLRFG
```
