# Supplementary material for: New insights into the FLPergic complements of parasitic nematodes: Informing deorphanisation approaches
Source: EuPA Open Proteom. 2014 Apr 19;3:262–72. doi: 10.1016/j.euprot.2014.04.002 (PMC4405611; doi:10.1016/j.euprot.2014.04.002)
Supplement: Supplementary Fig. 2 — Multiple sequence alignments of the predicted FLP-GPCRs identified by BLAST. Sequences were aligned using the Vector NTI Advance™11, Align X multiple sequence alignment tool, using default settings [32]. Completely and partially conserved amino acid residues are highlighted in yellow and blue respectively, while blocks of similar residues are highlighted in green. Predicted transmembrane domains are underlined on the consensus sequence for each FLP-GPCR alignment. Outside to inside helices have are identified by a solid line; inside to outside helices are identified by a dotted line. Note that only the longest C. elegans FLP-/putative FLP-receptor isoforms were included in the alignment such that C. elegans NPR-2 represents isoform a; C. elegans NPR-5 represents isoform b; C. elegans NPR-8 represents isoform b, C. elegans DMSR-1 represents isoform a; C. elegans EGL-6 represents isoform a; C. elegans FRPR-18 represents isoform b. T. spiralis, denotes Trichinella spiralis; T. muris, denotes Trichuris muris; B. malayi, denotes Brugia malayi; W. bancrofti, denotes Wuchereria bancrofti; D. immitis, denotes Dirofilaria immitis; O. volvulus, denotes Onchocerca volvulus; O. ochengi, denotes Onchocerca ochengi; L. loa, denotes Loa loa; A. suum, denotes Ascaris suum; A. caninum, denotes Ancylostoma caninum; N. brasiliensis, denotes Nippostrongylus brasiliensis; H. contortus, denotes Haemonchus contortus; C. elegans, denotes Caenorhabditis elegans; S. ratti, denotes Strongyloides ratti; B. xylophilus, denotes Bursaphelenchus xylophilus; G. pallida, denotes Globodera pallida; M. hapla, denotes Meloidogyne hapla; M. incognita, denotes Meloidogyne incognita. [file mmc3.pdf]

1. NPR-1 homologues

```
      B. malayi NPR-1     ----------------------------------------------------------------------------------------------------
   W. bancrofti NPR-1     ----------------------------------------------------------------------------------------------------
     D. immitis NPR-1     ----------------------------------------------------------------------------------------------------
    O. volvulus NPR-1     ----------------------------------------------------------------------------------------------------
     O. ochengi NPR-1     ----------------------------------------------------------------------------------------------------
         L. loa NPR-1     ----------------------------------------------------------------------------------------------------
        A. suum NPR-1     SLRLQPEVSGVGITLFSGYKTIATRLRYHHSHRFVNPKDISSGASLMKRMLNYGTTKRMKTIVDITFASICKRVIKRRISHDRSLALASVTRKYRCAPLT
     A. caninum NPR-1     ----------------------------------------------------------------------------------------------------
    H. contortus NPR-1    ----------------------------------------------------------------------------------------------------
     C. elegans NPR-1     ---------------------------------------------------------------------------------------------------M
N. brasiliensis NPR-1     ----------------------------------------------------------------------------------------------------
     S. ratti NPR-1.1     ----------------------------------------------------------------------------------------------------
     S. ratti NPR-1.2     ------------------------------------------------------------------------------------IIKYFKKYLYNMDVIV

            Consensus

      B. malayi NPR-1     -----MKCIVYTDKFRDPSMDIRVTVPFAIAYLLIFILGVIGNVLVVCLTLSHRKLQTVQNMFILNLASADLVVCIFSLPITPVANVYKNWYFGSAMCHG
   W. bancrofti NPR-1     ------------DKFRDPSMDIRVIVPFAIAYLLIFILGVIGNILVICLTLSDRKLQTVQ----------------------------------------
     D. immitis NPR-1     ------------DKFRDPSMDIHAIIPFGIAYLLIFILGVIGNVLVICLTLSHRKLQTVQNMFILNLASADLVVCIFSLPITPITNVYKNWYFGAAMCHG
    O. volvulus NPR-1     --------------FRDPSMDIRAIIPFGIAYLLIFILGVIGNVFVICLTLSNRKLQTVQNMFILNLASADLVICIFSLPITPVTNVYKNWYFGAAMCHG
     O. ochengi NPR-1     --------------FRDPSMDIRAIIPFGIAYLLIFILGVIGNVFVICLTLSNRKLQTVQNMFILNLASADLVICIFSLPITPVTNVYKNWYFGAAMCHG
         L. loa NPR-1     ---MVVMIMITMDKFHDPSMDIRAIIPFAIAYLLIFILGVIGNVLVICLTLSHRKLQTVQNMFILNLASADLVVCIFSLPITPVANVYKNWYFGSTMCHG
        A. suum NPR-1     IMSSHNGCDVYSQLYPDPSSSPLAIGTFTILYALIFLMGIIGNSALIYVTLQNRTLQTVQNMFILNLAASDIVMCILSVPVTPVTNIYKNWFFGAILCRL
     A. caninum NPR-1     ----------------------------------------------------------------------------RSVPLTPVTHIAFLWYFGSMLCRV
   H. contortus NPR-1     -------CRSYTEKFPDPSNHPVTVALFAVLYSFVFVISFAGNLGVIYATVRHRSLQTVQNIFIVNLAVSDVILCVLSIPLTPVTHIAKQWYFGSMLCRV
     C. elegans NPR-1     EVENFTDCQVYWKVYPDPSQSIYAIVPFLTVYLFLFFLGLFGNVTLIYVTCSHKALLSVQNIFILNLAASDCMMCILSLPITPITNVYKNWYFGNLLCHL
N. brasiliensis NPR-1     -------CRTYTEKFPDPSNHPITIALFAVLYTVVFVISFAGNLSVIYATVRHRSLQTVQNIFIVNLAVSDVILCVLSIPLTPVTHIAKQWYFGSMLCRV
     S. ratti NPR-1.1     -----DKCEVYYQMYPDPSSNPYIFIPFAIFYMVIFTLGIIGNVSIIYVTVRHKVLQSVQNMFILNLAASDIIVCLLSLPITPVTNVFKNWYFGEVLCRL
     S. ratti NPR-1.2     LLNKSDNCIGYSEMYPDPSNHLLAIIIIAIYYTIIFILGLIGNLYIIILTLKHKSLQSVQNIFILNLAISDIFLCLMSIPITPFVNIYKEWIFGSPACRI

            Consensus             C  Y D F DPS  I AIIPFAI YLLIFILGVIGNV VI LTLSHR LQTVQNMFILNLASSDLVVCI SLPITPVTNVYKNWYFGS LC L

      B. malayi NPR-1     LPWMQGIAVFIGTFSLCAISVDRYIMVILPTRRIINKTRSKYITIILWTLSIISTLPYAMYMDLNGGDG---ICGQFCTENGKLRLRRDIRYWF------
   W. bancrofti NPR-1     ----------------------------------------------------------------------------------------------------
     D. immitis NPR-1     LPWMQGIAVFIGTFSLCAISVDRYIMVILPTRRIINKTRSKYITIVLWTLSIISTLPYAMYMDLVVVDG---ICGQFCTENWPNSRLRSIYTIFVFVVQF
    O. volvulus NPR-1     LPWMQGIAVFIGTFSLCAISVDRYIMVILPTRRIINKTRSKYITIILWSLSIISTLPYAMYMDLVVVDG---ICGQFCTENWPNARFRTMYTVIVFVVQF
     O. ochengi NPR-1     LPWMQGIAVFIGTFSLCAISVDRYIMVILPTRRIINKTRSKYITIILWSLSIISTLPYAMYMDLVVVDG---ICGQFCTENWPNARFRTMYTVIVFVVQF
         L. loa NPR-1     LPWMQGIAVFIGSFSLCAISVDRYIMVILPTRKIINKTRSKYITIVLWTLSIISTLPYAMYMDLIVVDG---ICGQFCTENWPSSRLRTIYTIFVFVVQF
        A. suum NPR-1     IPYVQGVSVFISTFSLGAIALDRYVLVVHPHTRSLSKHGAMTVTTILWTLSIFVTLPYAFFMTIESYPG---VCGEFCTEKWPNASSRRAYTMVVLVAQF
```

```
        A. caninum NPR-1     VGGIQAIGLFIGTFSLCAIAIDRYFRLVIAPGSPLRKVNAIRITILLWIISILATLPYVYHMKMKKYPAIN-VCGEFCTEKWPNVHSKRIYTLFVLAIQF
       H. contortus NPR-1    VGGIQAIGLFIGTFSLCAIAIDRYFRLVIAPGSPLRKVNAIRITILLWIISIIVTLPYVYHMKMKKYPTISDVCGEFCTEKWPNVHSKRIYTLFVLAIQF
         C. elegans NPR-1    IPCIQGISIFVCTFSLGAIALDRYILVVRPHSTPLSQRGAFLTTVLLWILSFVVTLPYAFNMQMIEYTEE-RICGYFCTEKWESAKSRRAYTMIVMLAQF
    N. brasiliensis NPR-1   VGGIQAIGLFIGTFSLCAIAVDRYFRLVIAPGSPLRKVNAIRITILLWVISILATLPYVYHMKMRKYPSIN-VCGEFCTEKWPNVHSKRIYTLFVLAIQF
         S. ratti NPR-1.1    IALIQGVSIYICTFSLGAIAVDRYILVVKPHSKALSRKGALVVTGILWTLSLIFNIPYVLFMYIQSYFKG--VCGVFCTEKWPNPESRRAYTLMVMVAQF
         S. ratti NPR-1.2    SGSIQGIGIFISTYSLCAIAIDRYYRLVESPKTSLTKIQAIFATLTIWIISFIITFPYIYNMKLVTYNN---ICGYFCTEDWDNDKHRQIYTIILFFVQG

               Consensus     LP IQGIAVFIGTFSLCAIAVDRYIMVVLP  R L K  A  ITIILWTLSIISTLPYA YM L  Y G   ICG FCTE WPN   R IYTL V VVQF


          B. malayi NPR-1    ----------------------------------------------------------------------------------------------------
       W. bancrofti NPR-1    ---------------------------RLKERSMAMASAGLVENPNNNVLDCKR-------------------------NFGSN---------AFKENHT
         D. immitis NPR-1    IIPLTIMFICYYHIFAELRKRTSKKIMT------------------------------------------------------------------------
        O. volvulus NPR-1    IIPLTIMFICYYQIFAELRKRTSKKIMRLKERSVVMTSGGLSVENADNLLDCNR--------------------ISDNDAFQENQ---------------
         O. ochengi NPR-1    IIPLTIMFICYYQIFAELRKRTSKKIMRLKERSVVMASGGLSVENADNLLDCNR--------------------ISDNDAFQENQ---RNELVANARRNT
             L. loa NPR-1    VIPLIIMFICYYRIFAKLRKRTSKKIMRLKERSMAMASAGLLENPNNNMLDCKR--------------------NFDGNDFKENQDRLRNELLANARRNT
            A. suum NPR-1    VIPFIVMAFCYAVIFARLRTRARLRLQRINERCSFVLEKTNSPSSMRHPRFKAV--------------------SSEISDICMTQEKVRHHLLKQTRRTT
         A. caninum NPR-1    VIPFTIMTICYQAVFSFLRRRAKSRLTSIAQQANLLYVVAATAGGDTQQHKEQLT----------------------------HLIEQKRRVLQQRRRVT
       H. contortus NPR-1    VIPFTIMTICYQAVVFSFLRRRAKSRLTSIAQQANLLYVVAATAGGDTQQHKEQ---------------------------LTHLIEQKRRVMQQRRRVT
         C. elegans NPR-1    VVPFAVMAFCYANIVSVLSKRAQTKIRKMVERTSALESSCAFPSHGLEQYENEL------------------------NEFLDKQEKEKQRVVLQNRRTT
    N. brasiliensis NPR-1   IIPFTIMTICYQAVFAFLRRRAKSRLTSIAQQANLLYVVAATAGGDTQQHKEQLT----------------------------HLFEQKRRVTQQRRRVT
         S. ratti NPR-1.1    VFPFSVMAFCYATIFSKLKSRAKLRIRKIDERFVALGRSSNYTNISEHLAMETDNKTCVVLSTTLTVTNNIEISKGNVPTLALQKEKDKQRLLNQTRRNT
         S. ratti NPR-1.2    IIPFCIITLCYQQIFAKLHKRANFKILSISQQVNLLSVLTNTVCIEKKKRIEIQ-----------------------------------------KKKIT

               Consensus     VIPFTIM ICY  IFA LRKR   KI RI ER   L                                                    K  LL Q RR T


          B. malayi NPR-1    ----------------------------------------------------------------------------------------------------
       W. bancrofti NPR-1    ILLVSMVVLFAVSWLPHNIVSLLMEFDES----LFERHDGVNSIYLINLFTHWVAMANNVWNPILYALLNPLFIELMAKTLYRLRRLICCCFCGERSTTI
         D. immitis NPR-1    VLLVSMVVLFAISWLPHNIVSLLMEFDES----LFERSDGVNSIYLINLFTHWYVHA--------------------------------------------
        O. volvulus NPR-1    -----------------------------------------------------IAMANNVWNPILYALLNPLFIELMAKTLYRLR---------------
         O. ochengi NPR-1    ILLVSMVVLFAISWLPHNIVSLLMEFDET----IFERHDGVNNIYLINLFTH-IAMANNVWNPILYALLNPLFIELMAKTLYRLR---------------
             L. loa NPR-1    ILLVSMVVLFAVSWLPHNIVSLLMEFDES----LFERYDGVNSIYLINLFTHWVAMANNVWNPILYALLNPLFIELMAETLYRLRAILCCCFYREPSTIR
            A. suum NPR-1    MILVSMVLFFALTWLPHNIVSLIIEYDESNRFFRTFGIDNMSLSYLINLFTHSVAMTNNIANPVLYAWLNPTFRHLVIQTCCRRYQPIQRN-NVKCDANT
         A. caninum NPR-1    LILVSMVVIFGITSLPHNIVSTLMEFTDS---QDLLAYNGNDYTYLLNLITHFLAMLSCVANPLLYAFLNPEFRELILNGLAGIKWAPRFVSRTWQPTQT
       H. contortus NPR-1    LILVSMVVIFGITSLPHNIVSTLMEFTDSR---DLLAYNGNDYTYLLNLITHSLAMLSCVANPMLYAFLNPEFRELILNGLAGFKWTPRFVSRT------
         C. elegans NPR-1    SILVTMVVWFGITWLPHNVISLIIEYDDTQSFFRLYGRDDYDISYLLNLFTHSIAMSNNVLNPVLYAWLNPSFRQLVIKTYFGDRRKSDRIINQTSVYKT
    N. brasiliensis NPR-1   LILVSMVVIFGITSLPHNIVSTLMEFTDSR---DLLAYNGNDYTYLLNLITHFLAMLSCVANPLLYAFLNPEFRELILNGLAGIKWAPRFVSRT------
         S. ratti NPR-1.1    IILVSMVVIFGLTWLPHNVVSLVLEYDEDQTIFKIDGDDDLDLSYLVNLFTHSIAMTNIVFNPVLYAWLNPQFRDLCIQTFYKNRKAKSTNKNLSKCITS
         S. ratti NPR-1.2    IILILMVMIFACASLPLNIVSIITELDSSG---LFILNNGTDITYITNICAHGIAMIICITNPILYALLNPEFRILVMKSL-------------------

               Consensus     IILVSMVVIFAITWLPHNIVSLLMEFDES         DG   TYLINLFTH IAM N V NPILYA LNP FRELMI TL   R
```

```
        B. malayi NPR-1      ----------------------------------------------------------------------------------------------------
    W. bancrofti NPR-1       REETFEV---------------------------------------------------------------------------------------------
        D. immitis NPR-1     ----------------------------------------------------------------------------------------------------
      O. volvulus NPR-1      ----------------------------------------------------------------------------------------------------
       O. ochengi NPR-1      ----------------------------------------------------------------------------------------------------
            L. loa NPR-1     EEPLEVE---------------------------------------------------------------------------------------------
           A. suum NPR-1     SKISNNAAINCIGEISHDSPQEIGRIPQLKTATHEDKIHSHYRQKYDDEDVSCADESNEAKHPNVLHRYRVPTSHQNDGNTENPNENATALFTNNMMLIS
        A. caninum NPR-1    TCCATKAPHSVLVAI-------------------------------------------------------------------------------------
      H. contortus NPR-1     ----------------------------------------------------------------------------------------------------
        C. elegans NPR-1    KIVHDTKHLNGRAKIGGGGSHEALKERELNSCSENLSYHVNGHTRTPTPEVQLNEVSSPEISKLVAEPEELIEFSVNDTLV-------------------
 N. brasiliensis NPR-1      ----------------------------------------------------------------------------------------------------
        S. ratti NPR-1.1     ETKLNHNDSFIPLRDEGKKIITSYQTSKTMEQQQ------------------------------------------------------------------
        S. ratti NPR-1.2     ----------------------------------------------------------------------------------------------------

              Consensus
```

2. NPR-2 homologues

```
           A. suum NPR-2     --------------------------------------------------DCIVYIEEFQDPSMKLEVIASFALIYSVIFVLGVIGNTFVIFLTLRHRKL
        A. caninum NPR-2     ----------------------------------------------------------------------------------------------------
N. brasiliensis NPR-2.1      ---------------------------------------------------CHRYVDDHPDLTNEPIIMLVFAILYVHIFVLGIVGNVAVLYLTLKNRHL
N. brasiliensis NPR-2.2      --------------------------------------------------ECAVFTDQVKDPSLETPVVVVFSILYATEFFLGIIGNIGVIYLTFGNKKL
   H. contortus NPR-2.1      ---------------------------------------------------CHRYVDDHPDLTNELAVMIIFAVLYVHIFVLGIVGNVAVLYLTLKNRHL
   H. contortus NPR-2.2      ----------------------------------------------------------------------------------------------------
        C. elegans NPR-2     MSMVNTRVEAPDLLALKNMLRQSDGTRMLQEMRRKLIQLHSSQMINETEETCDRYIDKHPDMTNEPTVLVTFSLLYLHIFLLGILGNSAVLYLTMKHRQL
          S. ratti NPR-2     --------------LSQVNNTKGLQIMNNIHEYPQTCEYYQSLNYADSRYNCTVFHEKCPDLTNEIPVMISFLLYYTIIFVFGIIGNFIIAWLTLKNKSL
      B. xylophilus NPR-2   --------------------------------------------------------------------MTLFAIVYTHIFVLGLIGNLSIMFLTLRYHHL
        G. pallida NPR-2     --------MEMLFTSGDAPSSSSSTAATIQTARLHSAVVQLEQPHEQQQQQCLHYVDQQPDPSNSAWAITLFLALYSNIFVLGLLGNLAIVLVTIRCQHL
          M. hapla NPR-2     ------------------------------------------------------YIIQSPDPTNSLLAIIIFGIIYINIFIFGLLGNLAIVLLTLRCRHL
      M. incognita NPR-2     ------------------------------------------------------YIIQSPDPTNSLLAIIIFGIIYINIFIFGLLGNLAIVLLTLRCSHL

               Consensus                                                        C  YID  PD TN   VIIIFAILY  IFVLGIIGNLAILYLTLR RHL


           A. suum NPR-2     QTVQNMFILNLAGADLVVCIFSLPITPITNVYKNWYFGSTMCHGLPWMQG-IAVFIGTFSLCAISIDRYLMVTLPTKKIIDKTRSIWITAILWTLSIIFT
        A. caninum NPR-2     ------------------------------------------------------------------SRGSTTSASIEIPRSFRGSVVAVDNIRSS----L
N. brasiliensis NPR-2.1      QSVQNIFILNLAASDVLMCLTSLPITPITNVYKTWFFASPVCKLIPLVQG-ASVFVSTFSLSAIALDRYNLVVRPHRHPLRSRGASVVALLLWIISALVC
N. brasiliensis NPR-2.2      QTVQNMFILNLALADLIVCIFSLPLTPITNIYKNWYFGDKMCHSLPWIQG-ASVFVATFSLALIAINRYQMVVSPHHKRMTPTQARFVMIGLWISSVLIT
   H. contortus NPR-2.1      QSVQNIFILNLAASDVLMCLTSLPITPITNVYKTWFFASPVCKLIPLVQG-ASVFVSTFSLSAIALDRYNLVVRPHRHPLRSRGASVVALLLWIISALVC
   H. contortus NPR-2.2      ---QNMFILNLALADLIVCMFSLPLTPVTSIYKNWYFGELMCHSLPWIQG-ASVFVATFSLAAIAIDRYMMVVTPHQRRMT--HARLVMVCLWVISGLIT
        C. elegans NPR-2     QTVQNIFILNLCASNVLMCLTSLPITFITNVYKQWFFSSPVCKLIPLVQG-ASIFVSTFSLSAIALDRYNLVVRPHKQKLSSRSAMMIALLIWVISVVVC
          S. ratti NPR-2     QTVQNIFILNLVISDIVICIFSVPVTPITLIFKNWYFGSLMCHLMPLAQVNILFFVSTLSLSAIAIDRYILVVKPHTTPITIKGARNIAIALWLFSILIS
      B. xylophilus NPR-2   RTVQNIFILNLAISDVIVCLLSLPFTPVTSIYKVWMFGQPICHLLPLVQA-VSIFVSTFSLSAIAIDRYNLVVRPHARPLTIHGARNVAVVLWVLSVVVS
        G. pallida NPR-2     RSVQNIFILNLAISDIIVCLLSLPFTPVSYIYKEWLFGSAICHLLPMVQA-ASVFVSTFSLSAIAIDRYWLVLYPHSGSISPAKATVVAAVLWLLSVLIS
          M. hapla NPR-2     RSVQNIFILNLALSDVIVCLLSLPFTLVTNIFKEWLFGSAICHLLPMVQA-VSVLVSTFSLSAIAIDRYWLVK---------------------------
      M. incognita NPR-2     RSVQNIFILNLALSDVIVCLLSLPFTLVTNIFKEWLFGSAICHLLPMVQA-VSVLVSTFSLSAIAIDRYWLVHRPHGHSLSPSQATIVACILWAFSLFIS

               Consensus     QSVQNIFILNLALSDVIVCL SLPITPITNIYK WYFGS ICHLLPLVQG ASVFVSTFSLSAIAIDRY LVVRPH   LS   A VVALLLWIISVLIS


           A. suum NPR-2     LPYAMYMDVIVVDGICGQFCTEKWPDSGLRRTYTIFVFIVQFIIPLIIMFICYYKIFDELKKR-TTKKIKQIRERSVVLASALACTPNQVASHALAEKDT
        A. caninum NPR-2     YAYGWYMDVEQLPGICGEFCTERWPSSEVRKGYALMVLVMQFILPFATMAVCYYAIFARLRER-TESKLKKLTERTQLLES--TAGSSSPSGKNEAGTHP
N. brasiliensis NPR-2.1      MPYGWYMDVVRIGGLCGEFCTEQWPLPEVRKGYALMVLVMQFILPFATMAVCYYTIFARLRER-TESKLKKLTERSQLLES--AAGNQSPSAKAENGAHP
N. brasiliensis NPR-2.2      LPYSWYMALVEHDGLCGKFCTEIWPSPQLRQAYTVFVMIAQFFVPFWIMFYCYSRIFKHLKRR-TQAKIRKMNERSLILTASMPVLSTVKRKMGTKVDVL
   H. contortus NPR-2.1      MPYGWYMDVVRIGGLCGE-CTEKWPLPEVRKGYALMVLVMQFILPFATMAVCYYTIFARLRERRTESKLKKLSERSQLLES--AVGNSSPSAKTENGAHT
   H. contortus NPR-2.2      LPYSWFMTLEEHEGLCGKFCTEAWPNARLRQAYTVFVLITQFFLPFVIMFYCYSKIFKHLRRR-TQARIRKMNERSLILTASMPVLTTVKRKMDANVDIL
        C. elegans NPR-2     MPYGWYMDVEKLNGLCGEYCSEHWPLAEVRKGYTFLVLITQFLFPFATMAFCYYNIFSRLRQR-VETKLKKLSERSQLLENSTTCGTTNHIVSINAEAVQ
          S. ratti NPR-2     SPYGYVMDIEKYPGFCGDYCDEQWSDG-VKPIFSLAVFTAQFLIPCLTMAFCYICIFAKLRAR-TNVKLKKLTER-------------------SLLLDQ
      B. xylophilus NPR-2   LPYAYYMRLETYEELCGQFCTEIWPSKSMQRSYALVVLIMQFLLPFVTMSFCYSTIFSKLRER-ANSKLKKLDERSQLLNI---H----QALDKKDSVKL
```

```
        G. pallida NPR-2     VPYSVHMTLVEYDGYCGKFCTEQWPSTQIRRGYALSVLVLQFLIPFVTMSYCYASIFALLRRR-ANSKLQKLNER------------------QTMLAKL
          M. hapla NPR-2     ------------------FCSEQWPNIQLRRGYALAVLALQFLLPFLTMSFCYASIFSRLRRR-NNTKLKKLNER------------------QNMLARQ
      M. incognita NPR-2     LPYAYYMGLEEYEGYCGKFCSEQWPNTQIRRGYALAVLALQFLLPFLTMSFCYASIFSRLRRR-NNTKLKKLNER------------------QIMLARQ

              Consensus      LPYGWYMDLE YDGLCG FCTE WP   VRRGYAL VLIMQFLLPFLTMAFCYASIFARLRRR TNSKLKKL ERS LL S               LA


           A. suum NPR-2     KEFQDRLRCELVANARRNTILLVSMVVMFAIAWLPHNIVSLLMEFDERIFERDDGVN----IIYLFNLFTHS--VAMTNNVSNPILYALLNPLFIELIAE
        A. caninum NPR-2     CSFEEQQRLSVLASQRRTTTILASMVLLFGFTWLPHNVYTLVYEYDEGIFLNGKT-----DNTYIVSMVAHLI--SMTTNVANPILYAWL----------
 N. brasiliensis NPR-2.1     VTFEEQQRLSVLASQRRTTTILASMVLLFGFTWLPHNVYTLIIEYDDGFFHNGKS-----DNTYIVSMIAHLI--SMTTNVANPILYAWLNPSFKEMLLT
 N. brasiliensis NPR-2.2     EQCRR--RCLLLRQTRRNTVVLALMVIFFFISWCPHNVVSMALEFSDDGVFFINDTN----YSYLASLISHS--TAMISIMANPVLYGFLNRGFVSHIRQ
     H. contortus NPR-2.1     VTLEEQQRLSVLASQRRTTTILASMVLLFGFTWLPHNVYTLIIEYDEAFFHNGES-----DNTYIVSMIAHLI--SMTTNVANPILYAWLNPSFKEMLLT
     H. contortus NPR-2.2     EN----------------TVVLVLMVSFFFIAWCPHNVVSVALEFTDGDAFLIHETD----YSYLASLMSHRYVTNKFSIVVNGV---------------
        C. elegans NPR-2     NGLENKQRLAVLAQQRRTTTILSCMVLLFAFTWLPHNVVTLMIEYDGFFFHSDETSATSTDHTYIVSMTAHLI--SMLTNVTNPFLYAWLNPMFKEMLIK
          S. ratti NPR-2     ILTECKQIVYLLQQQRRTTSVLTSMVLIFVFAWLPYSISTILMDFNFDIYRYEDKSHRYIELSYFVQLLTHFLRIAMIMNIANPILYAWLNPNFKNIFMA
     B. xylophilus NPR-2     KNGDDTQRANLLAQQRRTTTILASMVLIFGLTWLPQNIVTLMLEYDDEIFSHGAKN-----YTYLVSMIAH-----------------------------
        G. pallida NPR-2     SSDRAHRVHAVSKQQRRTTLILVTIVLFFFFAWLPHNIREMVIEYDEQLLHWNSIN-----YTYFLSMVTHSV--AMTTNVANPILYAWLNPTFKEVFLR
          M. hapla NPR-2     SNR----LNALNKQQRRTTTILATVVLFFGLAWLPQNVLTMIIEYDINLLNWGNTN-----YLYLFSLISHSL--AMTTNVANPILYAWLNPTFKELFMK
      M. incognita NPR-2     S--------TLNKQQRKTTTILATVVLFFGLAWLPQNVLTMIIEYDIHLLNWGNVN-----YLYLLSLISHSL--AMTTNVANPILYAWLNPTFKELFMR

              Consensus         E  QR ALLAQQRRTTTILASMVLLFGFAWLPHNVVTLIIEYDD IF  G        YTYLVSLIAH I  AMTTNVANPILYAWLNP FKEL L


           A. suum NPR-2     SITHLRTTFIHTFFHRNDISNIL-----------------------------------------------------
        A. caninum NPR-2     ----------------------------------------------------------------------------
 N. brasiliensis NPR-2.1     SIRGKVRAQDK-----------------------------------------------------------------
 N. brasiliensis NPR-2.2     EWTNSMKKSKR-----------------------------------------------------------------
     H. contortus NPR-2.1     SIRGKTRKQDK-----------------------------------------------------------------
     H. contortus NPR-2.2     ----------------------------------------------------------------------------
        C. elegans NPR-2     TLRGGSKSPKPADIKQTSFIRMPNSGAPSQSSYL------------------------------------------
          S. ratti NPR-2     AIKRKKNSENINRNNI------------------------------------------------------------
     B. xylophilus NPR-2     ----------------------------------------------------------------------------
        G. pallida NPR-2     TANSLLLRRKMHNGNSGGSVRSEHGTSCEQKKQLLQQRESNRDTQQTTCVVSLGNGQTLVQANGAGALERPKNEYI
          M. hapla NPR-2     T----KILKKRKENN-------------------------------------------------------------
      M. incognita NPR-2     TTINPLIFRKRKENN-----LIQTSTKQLNTTSIRSSIQNNNSLNEKRILVHLNN---------------------

              Consensus      T
```

3. NPR-3 homologues

```
 A. suum NPR-3           ---------------------------------------------------------IQQSLPDKVDLLQVQIIFSVLYFLVWIVAVSGNSLVLYIVTVK
 A. caninum NPR-3        ----------------------------------------------------------------------------------------------------
 N. brasiliensis NPR-3   ------------------------------------------------ISGMSNCTNIRDSMFETIDLTAIRLVFGMLYTLVWVAAIVGNTLVLYVVSFN
 H. contortus NPR-3      -------------------------------------------------EMSTNCTRIQDTMFETIDLTAVRLVFCVLYCLVWVAAIVGNTLVLYVVSFN
 C. elegans NPR-3        -----------------------------------------------MEGGRNCVMTVQQWQPEYNDMNQIRAIFSLLYLLVWVGAIVGNTLVLYVLTFN
 B. xylophilus NPR-3     -----------------------------MSNVDVYDGVSPVWENFLWRHHQQNCTPIQESIPEKVDSLPIQLLFSVLYLTVWLCAITGNLCVIYVVTLK
 G. pallida NPR-3        MDYYSSPDNNQQQNNNNGLSTALAPDQWPQNVVEPNVWPHFLWRSHPPGDRHNICLPIQASFPELVDLLPIQLLFSVLYLLIWLASIVGNASVLYVVSLK
 M. hapla NPR-3          ----------------------------------------------------------------MVDLLPVQLIFSFLYLLIWLAAILGNVSVLYVVSLK
 M. incognita NPR-3      --------------------------------------------------------------PEMVDLLPVQLIFSFLYLIIWLAAILGNVSVLYVVSLK

 Consensus                                                                        IQ S PE VDLL IQLIFSVLYLLVWLAAIVGN  VLYVVSLK


 A. suum NPR-3           QVSLS-VRTIFIGSLAVSDILMSLTSLPVTAVTIFTRDWVFPSIFCKLIGVFQGGSIFVSSFTLTVIAVDRMLLILYPSRAIITFERALSIVLGIWAIGY
 A. caninum NPR-3        ---------------------------------------------------------------------------------------------AIWVLGY
 N. brasiliensis NPR-3   QVS---------------DLLMSLTSLPWTAVTIFTREWIFPAPICKLIGVFQ-----------------------------------------------
 H. contortus NPR-3      QVSLS-VRSVFIGCLAVSDMLMSLTSLPWTAVSIFTREWIFPAPICKLIGVFQGGSIFVSSFTLTVIALDRCLLILKPNKEIIGYNRAVAIVVAIWVTGY
 C. elegans NPR-3        QVSLS-VRTVFVGCLAGSDLLMCLFSLPITAISIFSRVWVFPAIFCKLIGVFQGGTIFVSSFTLTVIALDRCVLILRPNQEIVNFPRAVFIVFCIWLLGY
 B. xylophilus NPR-3     QVSLSAVRSVFICSLAVSDIVMSLTSLPITAVSIFTRDWVFPGICCKLMGLFQGGSVFVSSFTLTAIAVDRYILIKHPASSKVNFNKALYSVILIWIMGY
 G. pallida NPR-3        QVQLSSVRSVFICSLAASDILMSLTSLPITAISIFTRDWVFPSPFCKLMGIFQGGTVFVSSFTLTAIAVDRYILIRHPTKQKIKFRTAVSIVVGVWALGY
 M. hapla NPR-3          QVQLSSVRSVFICSLAASDILMSMTSLPITAVSIFTRDWVFPTFFCKLMGIFQGGAVFVSSFTLTAIAVDRYILIKHPTKQKIKFRTALSIIASLWVLGY
 M. incognita NPR-3      QVLSS-VRSVFICSLAASDILMSMTSLPITAVSIFTRDWVFPTFFCKLMGIFQGGAVFVSSFTLTAIAVDRYILIKHPTKQVIKFRTALSIVASLWMLGY

 Consensus               QVSLS VRSVFI SLA SDILMSLTSLPITAVSIFTRDWVFPA FCKLIGVFQGGSVFVSSFTLT IAVDR ILI HP K  I F  ALSIV  IWVLGY


 A. suum NPR-3           SLSLPNGIFSRTVHYFPYCGTFCEEAWPDSNLEDG----SKLGRIYGTAVMTLQFGVPALISSFCYWRISRVITQQIE-KRREQQIMLKESEKKLTSRRT
 A. caninum NPR-3        SLALPTGIFSKAVSFEKICGVFCEEIWPDTSESGLS---SPSRRLYGLSVLLSQFGIPALISSVCYWLISRVMSSQLE-RRRGH-TLLKESEEKLVNRKT
 N. brasiliensis NPR-3   ----------------------------------------------------------------------------------------------------
 H. contortus NPR-3      ALALPVGIFSRAISYGEICGVFCEEIW--FRPDAMETGHSAGRRLYGLSVLLSQFGVPALISSVCYWLISRVMSSQLE--RRRGHTLLKESEEKLVNRKA
 C. elegans NPR-3        SLALPVGIYSDIAVYDEICGTFCEENWPDFNPDTGR---SGIRRAYGLSVLVLQFGIPALISSICYWMISRVMSDQLA-RRRGH-NIRPESETKLVNRKT
 B. xylophilus NPR-3     LFALPVGIFSTTESYYPFCGSFCEETWPNFRPDTNNRGDSQMRKTYGLAVLIIQFGLPLVISSLCYYSIGRIISRQIK-KRHQQQILLQDNQHRLENRKH
 G. pallida NPR-3        ACALPIGIFSSTQVYPPFCGTFCEENWPDTDEQSGR---SRIRKLYGLLVLIVQFGLPTVISSLCYWSIGLNFELARE----RQVVLLQESKDRMKSQKN
 M. hapla NPR-3          TCTLPVGIFSSTVEYSPFCGQFCEENWPDTDTQSGR---SRLRKFYGLLVLIVQFGLPVLISTLCYWKIGQLIRLQMRRRSERQMVILQESKDRMKSQKS
 M. incognita NPR-3      ACAMPVGIFSSTVEYSPFCGQFCEENWPDTDTQSGR---SRLRKFYGILVLIVQFGLPVLISTLCYWQIGKLR--R---RSERQMVILQESKDRMKSQKS

 Consensus               A ALPVGIFS TV Y PFCG FCEE WPDT   SG    S LRK YGLSVLIVQFGLP LISSLCYW I RVIS QL  RR  Q VLL ES DKL  RKT
```

```
           A. suum NPR-3   RSNRMMVCMVLGLVLAWLPLNLINLSRDFN----GVTAWFSTIFAFCHVIAMTSAAWNPVIYSWFNPQLRMALKSI------------------------
        A. caninum NPR-3   RANRMMIAMVLGFIFAWMPKNAINLYRDLG----SSVNSNKVINGICAV---------------------------------------------------
   N. brasiliensis NPR-3   ---RMMIAMVLGLILAWMPLNAINLYRD----------------------AMTSAALNPVIYSWFNPQFR------------------------------
      H. contortus NPR-3   RANRMMITMVLGFIFAWMPLNAINLYRDFGSF--SNTTWFSTVFALCHVSAMTSAALNPVIYSWFNPQFRGAIQSL------------------------
        C. elegans NPR-3   RANRMMIVMVVGFVLAWMPFNAVNLYRDLF----GISKWYSTVFALCHVCAMCSAVLNPIIYSWFNPQFRQSITTLFKGTDEARLIKKKPQSTSKMVSYP
     B. xylophilus NPR-3   RSNRMMVAMVGGLVLAWLPMNLINLMRDFDASPDRFSDWYSLVFAGCHVVAMTSAVWNFVIYSWFNLQFKQTLLQACNQHTKQSGYNVVTRAKSVATEGP
        G. pallida NPR-3   RTNRMMVTMVIGLVLAWLPMNLINLWRDFNSE--NSTEWYSLIFAACHSVAMTSAVWNPLIYSFFNPQFKETIRTSLERRRQSQVPKFFGIFFVLLLKAL
          M. hapla NPR-3   KTNLMMATMVAGLVLAWLPMNLINLWRDLN----NNTAWFSLIFAASHSIAMTSAVWNPIIYSFFNPQFRETIRKVLERNNNNHLTTSSPNFNSNGYCSV
      M. incognita NPR-3   KTNLMMATMVAGLVLAWLPMNLINLWRDLN----NENSWFSLIFAASHSIAMTSAVWNPIIYSFFNPQFRETI---------------------------

             Consensus     RTNRMMI MVLGLVLAWLPMNLINLYRD        T WFSLIFA CHVIAMTSAVWNPVIYSWFNPQFR TI


           A. suum NPR-3   --------------------------------------------------
        A. caninum NPR-3   --------------------------------------------------
   N. brasiliensis NPR-3   --------------------------------------------------
      H. contortus NPR-3   --------------------------------------------------
        C. elegans NPR-3   TNFSEIRKETEIASTKTKITIAENDYRAGDQLL-----------------
     B. xylophilus NPR-3   EF------------------------------------------------
        G. pallida NPR-3   KKRIIFNNENAKFRLPPLC-------------------------------
          M. hapla NPR-3   NNNDYIRRSSARSLLSALGTAIGVKDDKREGLKTMRNSVVFLPPKRLKNK
      M. incognita NPR-3   --------------------------------------------------

             Consensus
```

4. NPR-4 homologues

```
      B. malayi NPR-4     ----------------------MYNNNNTCLDMNAELWRNRRDWSTQSFTIFIFVFFYAAIILIGIVGNLCVILAISRTRTLQTVPNMFIFSLSCSDLVV
   W. bancrofti NPR-4     ----------------------MYNNNNTCLDMNAELWRNRRDWSTQSFTIFIFVFFYAVIILIGIVGNLCVILAISRTRTLQTVPNIFIFSLSCSDLVV
     D. immitis NPR-4     -------------------------DNNTCLDMNAELWRNKHDWSTHSSTIFIFVFFYTAIILVGIVGNLCVILAISRTRTLQTVPNMFIFSLSCSDLVV
     O. vovulus NPR-4     -------------------------NNNTCLDMNAELWRNSRDWSTQSSTVFIFVFFYGAIILIGIVGNLCVILAISRTRTLQTIPNMFIFSLSCSDVVV
     O. ochengi NPR-4     -------------------------NNNTCLDMNAELWRNSRDWSTQSSTVFIFVFFYGAIILIGIVGNLCVILAISRTRTLQTIPNMFIFSLSCSDVVV
         L. loa NPR-4     ----------------------MYNNN-TCLDMNAELWRNRRDWSTQSSTIFIFVFFYAAIILIGIIGNLCVILAISRTRTLQTVPNMFIFSLSCSDLVV
        A. suum NPR-4     -NALWCNKSRCAVLFLILPKKSLRMSNESCLDMNDELWRFRNDLTTQPAVMAIFALLYATIIVLGIFGNLCVILAIARTRSLQTVPNLFIFSLSCSDVVV
N. brasiliensis NPR-4     -----------------------MRNSTTCLNLNDELWEYRNDYTTRAFTMVIFAAFYSVIILCGFVGNICVILAITRNKVLQTVPNLFILSLSCSDIAV
   H. contortus NPR-4     --------------------------TTPVLSRTSKFYSKRSEFCFQVSTMVIFAILYSVIILCGFIGNVCVIVAITRNKVLQTVPNLFILSLSFSDIAV
     C. elegans NPR-4     ------------------------MNGSDCLNLNSELWLYREDLSSRWYIMLVFAFLYLIIIAAGIIGNSCVILAITRNKSLQTVPNLFILSLSCSDIVV
       S. ratti NPR-4     ----------------------------NCTTLNEYVRQTSNDWSTYYSVWFIFAVIYSIIISLGIVGNLCVVLAVIRTKSLQTVPNYFIMSLSCSDMVV
  B. xylophilus NPR-4     -----------------------------CSSVNEQLWVLRRDPTTQLWVWALFAFFYSLIIICGCLGNLCVILAISRTKSLQTVPNMFIFSLSCSDFMV
     G. pallida NPR-4     -----------------------MAFPNDCVDMNHFLWEFQRDPTSRPLTAVIFAVVYSLIVLIGVSGNVCVLLSIGRTRSLQTVSNLFIFSLSCSDIVV
     M. hapla NPR-4.1   ---------MELHSPSTLLFNASSTPSVICEDMNAYLWNARRDLTTRPFVMLAFASFYSSIIILGLIGNSCVIIAIARIRSLQTVPNMFIFSLSCSDMLV
     M. hapla NPR-4.2   MQQEIHLIYNNNQQNNVNNYSSEFISLIPCVDMNKILWALRRDPTARLPIAALFASLYTIKNIFLFKGNGCVLLAISRTKSLQTVSNLFIFALSCGDTGV
   M. incognita NPR-4     ----MEASTMELLPPPTILFNVSSAPSVICEDMNAYLWNTRRDLTTCPFVMAVFASLYSSIILLGIIGNSCVIMAIARIKSLQTVPNMFIFSLSCSDILV

            Consensus                                 TCLDMNAELW  RRDWSTQ  TM IFAFFYSVIILIGIVGNLCVILAISRTRSLQTVPNMFIFSLSCSDIVV


      B. malayi NPR-4     CFTSATITPIAAFKKEWIFGAVLCSVAPFIAGASLCFSTFTLSAISVDRFLLIYFPTRKALSRLQALIVIMVICMLSTSLSAPVIFKQRLKRFGNYCGHF
   W. bancrofti NPR-4     CFTSATITPIAAFKKEWIFGPVLCSIAPFIAGASLCFSTFTLSAISVDRFLLIYFPTRKALSRLQALTVIMVICMLSTSLSAPVIFKQRLKRFGNYCGHF
     D. immitis NPR-4     CFTSATITPITAFKKEWIFGAVLCSIAPFIAGTSLCFSTFTLSAISVDRFLLIYFPTRKALSRLQALIVIMVICILSTSLSAPVIFKQRLKTFGNYCGQF
     O. vovulus NPR-4     CFTSATITPITAFKKEWIFGAVLCSIAPFIAGTSLCFSTFTLSAISVDRFLLIYFPTRKALSRLQALIVIMVICILSTSLSAPVIFKQKLKTYANYCGQF
     O. ochengi NPR-4     CFTSATITPITAFKKEWIFGAVLCSIAPFIAGTSLCFSTFTLSAISVDRFLLIYFPTRKALSRLQALIVIMVICILSTSLSAPVIFKQKLKTYANYCGQF
         L. loa NPR-4     CFTSATITPIAAFKKEWIFGAVLCSIAPFIAGASLCFSTFTLSAISVDRFLLIYFPTRKTLSRLQALIVIMVICILSTSLSAPVIFKQRLKRFGNYCGQF
        A. suum NPR-4     CCISATITPITAFKKEWLFGVTLCSIAPFIAGISLCFSTFTLTAISVDRFLLICFPMKNALTKSHALILIAGICAMAACLSAPIMFKQRLQTFENFCGKF
N. brasiliensis NPR-4     CSISATITPITAFKKEWIFGAALCRFAPFIAGISLCFSTFTLTAISIDRYLLIRFPMKKPLSHLHAILVIGLTCVLAACISSPIIVRQKLGKFENFCGQY
   H. contortus NPR-4     CSISATITPITAFKKEWIFGEILCRVAPFIAGISLCFSTFTLTAISIDRYLLIRFPMKKPLTHFHAVLVIGLTCVLAACISSPIIFRQKLGSFENFCGQY
     C. elegans NPR-4     CCTSATITPITAFKKEWIFGEALCRIAPFIAGISLCFSTFTLTAISIDRYILIRFPMRKPITHYQAVGVIAIICAFAATITSPIMFKQKLGEFENFCGQY
       S. ratti NPR-4     STISATVTPFTAFAKNWYFGRHGCMIFNTLG-MSIIFSTLTLTAISIDRLILIRYPMKTPINQSQAFIWIFCNVAAGIIFSLPSAYHSEIQVISNICGEF
  B. xylophilus NPR-4     CLTSATITPITAFAKVWLFGSVLCSVAPFLAGTSLSFSTFTLAAISIDRFMLIRFPMKKPFSHTQAFMII---CTMAMGLSMPVALMHTLTKINNYCGMY
     G. pallida NPR-4     CCVSATFTPYTAFTKVWVFGVALCSLVNFTAGTSLCFSIFILAAISVDRFLLIRFPLNNQLRHNHALLIIPILCLLAMFVSFPMTFTQKLLRMEGYCGEF
     M. hapla NPR-4.1   CFISATITPIAAFRKDWIFGQFLCSFAPFLAGVSLCFSTFTLAAISVDRFLLILFPTRKAFSHTQALFIILATCLLASGFSLPMLFMQKLKPVAHYCGRF
     M. hapla NPR-4.2   WFVSATFTRLLLFK-----------------GTSLCFSAFTLAAISVDRFLLIRFPLNNQLRHKHAFFTILVISLLSMILSLPMLFTQKLVKMEGYCGEF
   M. incognita NPR-4     CFISATITPIAAFKKDWIFGQFLCSFAPFVAGGSLCFSTFTLAAISVDRFLLILFPTRKAFSHTQALFIILVAFLLASGFSLPMLFMQKLKPVTHYCGRF

            Consensus     CFTSATITPITAFKKEWIFG VLCSIAPFIAG SLCFSTFTLSAISVDRFLLI FPTRKALSH QALIVIMVICILAT LSAPVIFKQKLK F NYCG F
```

```
     B. malayi NPR-4     CTEEWGIDQ-SGRRVYGSIMLSVQFIVPLVIITFCYTAISFKLGK----------------------QRAAIKRRQRTNRMLIGMVVAFSASWFFSVLFN
  W. bancrofti NPR-4     CTEEWGIDQ-SGRRVYGSIMLSVQFIVPLVIITFCYTAISFKLGK----------------------QRAAIKRRQRTNRMLIGMVVAFSASWFFSVLFN
    D. immitis NPR-4     CTEEWGIDQ-SGRRIYGSIMLSVQFIVPLIIITFCYTAISFKLGK----------------------QRAAVKRRQRTNRMLIGMVVAFSASWFFSVLFN
    O. vovulus NPR-4     CTEEWGMDQ-SGRRIYGSIMLSVQFVVPLMIITFCYTAISFKLGKGILQRKLRQ-QNEWQLGMSDQAR-AAIKRRQRTNRMLIGMVVAFSASWFFSVLFN
    O. ochengi NPR-4     CTEEWGMDQ-SGRRIYGSIMLSVQFVVPLMIITFCYTAISFKLGKGILQRKLRQ-QNEWQLGMSDQARRAAIKRRQRTNRMLIGMVVAFSASWFFSVLFN
        L. loa NPR-4     CTEEWGIDQ-SGRRVYGSIMLSVQFIVPLVIITFCYTAISFKLGKGILQRNRKH-HN-EWQLGMTDQQRAAIKRRQRTNRMLIGMVVAFSASWFFSVLFN
       A. suum NPR-4     CTEDWGMDQ-SGRRIYGAIMVGVQFVVPLTIIVICYTAIAVKLGQGMLIKGKN--SNYDWQVQMTDQQRAAVRRRQRTNRMLICMVVAFSASWLWSVLFN
N. brasiliensis NPR-4    CTEDWSDNE-SQRKVYGAALLCVQLVVPLTIIVVSYTAISLRIGQSMILRNTKRYATTNWNVQLTDQQRMALKRKQRTNRMLIAMVVAFSASWMWSVTFN
   H. contortus NPR-4    CTEDWSDNE-SQRKVYGAALLCVQLVVPLTIIVVSYTAISLRIGQSVILRNTKRDSSSNWNVQLTDQQRMALKRKQRTNRMLIAMVVAFSASWMWSVTFN
    C. elegans NPR-4     CTENWGANE-SQRKIYGAALMFLQLVIPLTIIIISYTAISLKIGQSMILKGAKKQKTDNWEMELSDQQRIAVKRRQRTNRMLIGMVVAFACSWIWSVTFN
      S. ratti NPR-4     CDENWGDDKDSFRMYYSVLIYAVQFVIPLTIIIICYTLISLRLNQSLLLKKAK--NKNGWET---DQERVAVKRKARTNRMFIGMVVAFILSYLPNVVFN
  B. xylophilus NPR-4    CFEDWGPYS-SQRRAYGTIVLSVQFIIPLSIIIICYTAISVRLGQSMILKGKK--RDYQWQLQMNDQHRAATKRRQRTNRMFIAMVVAFSLSWVWSVVFN
     G. pallida NPR-4    CVEDWGSNE-LYREIYGTLLLIVQFFVPLLIISICYIGISVRLSRNILI---R--RKKMGNSWLHHRQSMADRRRQRTNRMFIAMVIAFSLSWVWSVLYN
     M. hapla NPR-4.1    CFEDWGEMH-GIRRIYGTLLLTVQFIIPLNFIILCYTAISFRLGKGVN--LRTK-KKCEWKIPISAQRNAATKRRQRTNRMFIAMVIAFSFSWGWSVLYN
     M. hapla NPR-4.2    CFEHWSPYE-RLREVYGTILLIVQFVIPLIIITFCYTGISVRLNKSVLL------RKKMGNTWQIYHQKMANQRRQRTNRMFVLMVFVFILSWIWTVLFN
   M. incognita NPR-4    CFEDWGEMI-GIRRIYGTLLLTVQFIIPLALITFCYTAISFRLGKSVN--LRTR-KKCEWQMPISAQRNAATKRRQRTNRMFIAMVIAFSVSWIWSVLYN

          Consensus      CTEDWG D  S RRIYGSILLSVQFVVPLVIITFCYTAISFKLGK ILLR  K      WNV L  QQRAAIKRRQRTNRMLIGMVVAFSASWIWSVLFN

     B. malayi NPR-4     VLRDYDCLPKWCQNQEYFFGIATHCIAMSSTVW-NPLLYAALNLQLRAAFFRLLPECVRQPEMQKSATYNCKPL----LQSGSALRNDLTK-NKILNCDH
  W. bancrofti NPR-4     VLRDYDCLPKWCQNQEYFFGIATHCIAMSSTVW-NPLLYAALNLQLRAAFLRLLPECVRQRKVQKSATYNCKPL----LQSGSALRNDFTK-NKILNCDY
    D. immitis NPR-4     VLRDYDYLPEWCQDQEYFFGIATHCIAMSSTVW-NPLLYAALNLQLRAAFLRLLPECVTQLQARKSTAHNCKPL----LQSRSVLRNDFTK-NKVLNCD-
    O. vovulus NPR-4     ILRDYDCLPQWCQHQQYFFGIATHCIAMSSTVW-NPLLYAALNLQLRAAFLQLLPKCVRQRQTQESTTVHSR----------KPLRDDFSK-NKLLNTDR
    O. ochengi NPR-4     ILRDYDCLPQWCQHQQYFFGIATHCIAMSSTVW-NPLLYAALNLQLRAAFLQLLPKCVRQRQTQESTTVHSR----------KPLRDDFSK-NKLLNYDR
        L. loa NPR-4     VLRDYDCLPEWCQDQEYFFGIATHCIAMSSTVW-NPLLYAALNLQLRAAFLRLLPECVRQREERKSATYNCKPL----LQSRNVLRDDFTK-SKVLNCDH
       A. suum NPR-4     VLRDYDRLPDWMKIQEYLYGIATHCIAMTSTVW-NPLLYAVLNPQLRAAFIRLMPSCLQSTTTFERCDQLTRNGTLLETGDRRDSTKMQQNVRQAISSAP
N. brasiliensis NPR-4    VLRDYDYLPQVIKDQEYLFGIATHCIAMTSTVW-NPLLYAMLNLQLRAAFIELLPRVIRAKLRIGNDNASRLLNG--AAQPTLASSPSLKYGSS------
   H. contortus NPR-4    VLRDYDYLPQMIKDQEYLFGIATHCIAMTSTVW-NPLLYAMLNLQLRAAFIELLPQVIRIRLRLRNDHSIRLING---SHHSIAAIPTLKYGSSVRFSFS
    C. elegans NPR-4     ILRDYEYLPELIKTQEYIFGIATHCIAMTSTVW-NPLLYAVLNLQLRAAFIDLMPHWLRRHLNLEGDNSSPLLN-----HPTMTITN--KYGSTATKTVK
      S. ratti NPR-4     ILRDFNQVPKFISEQGYFFGIVMHCIAMTSTIW-NPLLYAALNYQLRAAFIDLMPACIHRKL--------------------------------------
  B. xylophilus NPR-4    VFKDYELLPSYVMEKEYIVGISLHCIAMTSTVW-NPLLYAMMNLQLRAAFIQLMPVCIKEYLYKEEEPHPMAQSCKPRPSTLLTPNGNVDQSSLPSGCTT
     G. pallida NPR-4    LLRDYECLPPLVTRQEFFFGILTHCIAMSSTVWWNPILYALLNLQLRSAFLRLVPESTRKFMAKRKFDRIGSKSKLTAKDAKLMAPTLLMATFEQRKTVK
     M. hapla NPR-4.1    LLRDYDGLPNLVRDQEFFFGILTHCIAMSSTVW-NPILYALLNLQLRAAFLQLVPQCIKHSLTCTCSKSNGR------LSGRSVARCIYLEESQLLGSSV
     M. hapla NPR-4.2    ILRDFGFLPEFISEQEFLYGVITHAIAMTSTVW-NPILYALLNMQLRNAFLNLIPIKLRKWLSKERSLNKLLAPAPSALYISSNKKEIINNLPPRASSCL
   M. incognita NPR-4    LLRDYDGLPKFVHDQEFFFGILTHCIAMSSTVW-NPILYALLNLQLRAAFLQLVPQCIRHSLTCTCSPNKGR------LSGRSVARCAYLEESRLLGSSV

          Consensus      VLRDYD LP WI DQEYFFGIATHCIAMSSTVW NPLLYALLNLQLRAAFL LLP CIR  L         R             R        LL S

     B. malayi NPR-4     RKSCDLLAVYKAVNRYESFEVDDI----------------------------------------------------------------------------
  W. bancrofti NPR-4     RKR-------------------------------------------------------------------------------------------------
    D. immitis NPR-4     ----------------------------------------------------------------------------------------------------
    O. vovulus NPR-4     RRR-------------------------------------------------------------------------------------------------
```

```
     O. ochengi NPR-4      RR--------------------------------------------------------------------------------------------------
         L. loa NPR-4      RRR-------------------------------------------------------------------------------------------------
        A. suum NPR-4      GQYGATESSKGSPNVIIHLTTIGRDRYSRKFERCSFRSLVQRLSTKRENTSLVNNAHEFRRYSQQYQILSNSTSSGSPITASQLINDCQQIQHSFIKRSI
N. brasiliensis NPR-4      ----------------------------------------------------------------------------------------------------
   H. contortus NPR-4      YAFPQECSLLYHVNFELHYSNNVNFMWSTSNYSQSLY---------------------------------------------------------------
     C. elegans NPR-4      ATYINTSNGQPYVSTSLVGKVQPEAPSFKFNGSGRKKSAMMRILVQKRNAEEEEQLITKESPSPPEIQMDTLCAASIIPRRKSAQPRSTNEKVVLPRKAS
       S. ratti NPR-4      ----------------------------------------------------------------------------------------------------
  B. xylophilus NPR-4      PRNGWLAPGFLKTGPRNSISSMASAFSSQPNGGLDDGTRLVQSTLTSTKYGAVESSENTRWSDEGFVDHGTVRLTTF-----------------------
     G. pallida NPR-4      ERPNEPRQLKTDFLPKPRKFESRTNKSLCSSEGR------------------------------------------------------------------
      M. hapla NPR-4.1    LRGASSHLQKRETGYGNKNCFNCLIGSSGSIESSSNETQKQMIAVRSAEKKRDDQIPVQKNFFQMC----------------------------------
      M. hapla NPR-4.2    LPLAHNKNEENYLKSSGDELFSSTTKSGTNLVSGSASVRSQSINLKSTRSVRGTGATISA----------------------------------------
   M. incognita NPR-4      LRGTTGQVQRRETNYENKNCFNCLIGSSRSVESSSNETQKQTFSVKCEGNKGDAKILSHNHHRHQLERITADVGFDEQTWTEQLRKDSEDDALDDCNDDL

          Consensus         R


      B. malayi NPR-4      -------------------
    W. bancrofti NPR-4     -------------------
      D. immitis NPR-4     -------------------
      O. vovulus NPR-4     -------------------
     O. ochengi NPR-4      -------------------
         L. loa NPR-4      -------------------
        A. suum NPR-4      SHLHNESANCPFIIRSYSY
N. brasiliensis NPR-4      -------------------
   H. contortus NPR-4      -------------------
     C. elegans NPR-4      F------------------
       S. ratti NPR-4      -------------------
  B. xylophilus NPR-4      -------------------
     G. pallida NPR-4      -------------------
      M. hapla NPR-4.1     -------------------
      M. hapla NPR-4.2     -------------------
   M. incognita NPR-4      RERDTDERI----------

          Consensus
```

5. NPR-5 homologues

```
    B. malayi NPR-5    -------------------------------------------------------------------MLIGITGNGSVLIAFTKNRRLRSARNIFLLNLI
  W. bancrofti NPR-5   -------------------------------------------------------------------MLIGITGNGSVLIAFIKNRRLRSARNIFLLNLI
    D. immitis NPR-5   -------------------------------------------------------------------MFIGIIGNGSVLIAFTKNRRLRSARNIFLLNLI
    O. vovulus NPR-5   -------------------------------------------------------IKVIFWLLYGLLMIIGITGNGSVLIAFTKNRRLRSARNIFLLNLI
    O. ochengi NPR-5   -------------------------------------------------------IKVIFWLLYGLLMIIGITGNGSVLIAFTKNRRLRSARNIFLLNLI
        L. loa NPR-5   -------------------------------------------------------------------MFIGITGNGGVLIAFTKNRRLRSARNIFLLNLI
       A. suum NPR-5   -------------------------------------------------------VQVAFSLVYGALMIVGLVGNGGVLFAVSKNKRLRSARNIFLLNLI
    A. caninum NPR-5   ----------------------------------------------------QLFVKSAFTLVYGFLFILGLVGNGGVVFAMANNRRLRSARNIFLLNLI
N. brasiliensis NPR-5  -----------------------------TENATCILVVDAIRSEGLDDVTTRLFVKSAFTLVYGLLFVLGLVGNGGVVFAMANNRRLRSARNIFLLNLI
   H. contortus NPR-5   ----------------------------------------------------QLFVKSAFTLVYGLLFILGLVGNGGVVFAMANNRRLRSARNIFLLNLI
     C. elegans NPR-5   --MVSSAATISTISTTTTPSTISNVITSHSNNGSCIQIAEAIAAQGIDDITVDFYIRSIFTFLYGFLFVLGIFGNGGVLWAVARNKRLQSARNVFLLNLI
      S. ratti NPR-5    -------------------------------------------------------IRFLITFTYAALMIIGIFGNGGVITIVLKNKRLRNARNIFLMNLM
  B. xylophilus NPR-5  -------------------------------------------------------VQALFGLVYLVLFVLGLVGNGGVFLAVLQNKRLRSARNIFLLNLI
  G. pallida NPR-5.1   ---------------------MDNETNVQTAKKECIEVAEAIRAQGINDLASWPSFQSGFTIVYGCLFLLGLLGNGSVLLAFAQNKRLRSARNLFLLNLI
  G. pallida NPR-5.2   ------------MNYSPQQKKIEQIPISTDSKGNGSEQQIVVSGRMASQWQPPAEDCVHLSEVITAHGIDDWTNNGSVLMAFGRANRIRSTRNFFLLNLI
      M. hapla NPR-5    MALIEEKQQHNLTPQTSSYISP---SSNNQQQPVCTEVSEAILSQGIDDLTSWAAVQCAIAAAYGCLFLLGLLGNGSVLLAFAQNKRLRSTRNLFLLNLI
   M. incognita NPR-5   MTLIEEKHQNHLTPQTSAYHQNENISPNNQQQIVCTEVSEAILAQGIDDLTSWTAVQCAIAAAYGCLFLLGLLGNGSVLLAFAQNKRLRSTRNLFLLNLI

            Consensus                                                         V   F  VYG L ILGIVGNGSVLIAF KNRRLRSARNIFLLNLI

    B. malayi NPR-5    ITDLLLCVTAIPVTPWYALTKNWTFGALMCRLMPLSNSCSVFVTSWSLTAIAIDKFVHIM-GPIRAPVSISQAGLVTAFIWVVSTMINIPYLMSYELVDG
  W. bancrofti NPR-5   ITDLLLCVTAIPVTPWYALTKNWTFGALMCRLMPLSNSCSVFVTSWSLTAIAIDKFVHIM-GPIRAPVSISQAGLVTAFIWVVSTMINIPYLMSYELVDG
    D. immitis NPR-5   ITDLLLCVTAIPVTPWYALTKNWTFGALMCRLMPLSNSCSVFVTSWSLTAIAIDKFVHIM-GPIRAPVSISQASLVTTFIWVVSTMINIPYLMSYELVDG
    O. vovulus NPR-5   ITDLLLCVTAIPVTPWYALTKNWTFGALMCRLMPLSNSCSVFVTSWSLTAIAIDKFVHIM-GPIRAPVSISQAGLVTAFIWVISTVINIPYLLSYELVDG
    O. ochengi NPR-5   ITDLLLCVTAIPVTPWYALTKNWTFGALMCRLMPLSNSCSVFVTSWSLTAIAIDKFVHIM-GPIRAPVSISQAGLVTAFIWVISTVINIPYLLSYELVDG
        L. loa NPR-5   ITDLLLCVTAIPVTPWYALTKNWTFGALMCRLMPLSNSCSVFVTSWSLTAIAIDKFVHIM-GPIRAPVSIGQASLVTAFIWVVSTMINIPYLMSYELVDG
       A. suum NPR-5   LTDILLCVTAIPVTPWYALTKNWLFGALMCRLMPLSNSCSVFVTSWSLTAIALDKFVHII-DPTRAPVRVRQAALITLFIWLVCSLINIPYLMSYDLVDG
    A. caninum NPR-5   LTDLLLVLTAVPVTPW------------------------------------------------------------------------------------
N. brasiliensis NPR-5  LTDLLLVLTAVPVTPWYALTKNWQFGSAMCHIMPLSNSCTVFVTSWSLTAISIDKYLHII-DPTKAPVTKRQALAITLLIWLLSTLINVPYLLSYELVDG
   H. contortus NPR-5   LTDLLLVLTAVPVTPWYALTKNWQFGSAMCHIMPLSNSCSVFVTSWSLTAISIDKYLHII-DPTKAPVTVRQALIITLLIWLLSTLINVPYLLSYELVDG
     C. elegans NPR-5   FTDLILVFTAIPVTPWYAMTKDWAFGSVMCHLVPLSNSCSVFVTSWSLTAISLDKFLHIN-DPTKQPVSIRQALAITFLIWIVSTLINLPYLMSFEHVDG
      S. ratti NPR-5    VTDLILCCFGIPLTYIYAREKKWTLGLIMCRLTPLGSNCAVFVS-WSLCAIALDKFIHIV-NPIRQPVSIKQATVITVLIWILSILANIPPIVNSDLKSG
  B. xylophilus NPR-5  LTDILLCLTGIPVTPWYALSKEWVFGSLMCRLMPLSNSCSVFVTSWSLTAIAIDKFMHII-DPTREQFSMKLAGMVTVLIWAVSTCANIPYLMSSELVDG
  G. pallida NPR-5.1   LTDILLCLTAVPVTPWYALTKDWSFGSLMCRVVPLSNSCAVFVTSWSLTAIAVDKFMHII-DLTREQFSLRTAGFVTFIIWTLSSLLNIPYLLSYELVNG
  G. pallida NPR-5.2   LTDVLLCFTAIPITPCYAVQKNWVFGPVLCRFVPFCNSCAVFVTR----AIAFDKHMHIVVDPTRRRFSRRLIGFVTLAIWLMSLVVNIPYLLSFELVQG
      M. hapla NPR-5    LTDILLCLTAVPVTPWYALTKDWAFGPLMCRLVPLSNSCAVFVTSWSLCAIALDKFLHII-DPTRGQFSLRTAATITFVIWILSTLLNIPYLLSYKLVNG
   M. incognita NPR-5   LTDILLCLTAVPVTPWYALTKDWAFGPLMCRLVPLSNSCAVFVTSWSLCAIALDKFLHII-DPTRGQFSLRTAATITFVIWIISTLLNIPYLLSYKLVNG

            Consensus  LTDLLLCLTAIPVTPWYALTKNW FGALMCRLMPLSNSCSVFVTSWSLTAIAIDKFVHII DPTRAPVSIRQAGLVT  IWVVSTLINIPYLLSYELVDG
```

```
      B. malayi NPR-5     AYYVP--NNTAPFCGLFCDEINWNGEMPRRLYGSSVVLLQ-FVIPLTIITYCYARILAKVASDMIIQNVQFSKSLSIAQREEATNRRKRVNYILIGMVIA
    W. bancrofti NPR-5    AYYVP--NNTAPFCGLFCDEINWNGEMPRRLYGSSVVLLQ-FVIPLTIITYCYARILVKVASDMIIQNVQFSKSLSIAQREEATNRRKRVNYILIGMVIA
      D. immitis NPR-5    AYYVP--NNTTPFCGLFCDEINWNGETPRRLYGSAVVLLQ-FVIPLIIITYCYTRILAKVSSDMIIQNVQFSKSLSNAQREEAINRRKRVNYILIGMVIA
      O. vovulus NPR-5    AYYVP--NNTKPFCGFFCDEINWNGETPRRLYGSTVVLLQ-FVIPLIIITYCYARILAKVASDMIIQNVQFSKSLSTAQREKATNRRKQVNYILIGIVIA
      O. ochengi NPR-5    AYYVP--NNTKPFCGFFCDEINWNGETPRRLYGSTVVLLQ-FVIPLIIITYCYARILAKVASDMIIQNVQFSKSLSTAQREKATNRRKQVNYILIGIVIA
          L. loa NPR-5    AYYVP--NNTAPFCGLFCDEINWNGETPRRLYGSAVVLLQ-FVIPLTIITYCYTRILAKVASDMIIQNVQFRKSLSTAQREEATNRRKRVNYILIGMVIA
         A. suum NPR-5    AYYVP--ENATPFCGHFCDELNWQGETPRQLYGSTVMLLQ-FVVPLAIITYCYSRILEKVAKDMIIQNAQFSESLSTAQRVEATQRKKRVNYILIGMVVA
      A. caninum NPR-5    ----------TPYCGKFCDETNWQSENSRRLYGTMVMLLQ-FVIPMAIITYCYFRILRKVSKDMIIQNVQFSASLSQKQRVDAISRKKKVNYILIGMVAT
 N. brasiliensis NPR-5    SYYVP--ENSTPYCGKFCDETNWQSENSRRLYGTMVMLLQ-FVIPMAIITYCYFRILRKVSQDMIIQNVQFSASLSQKQRIDAISRKKKVNYILIGMVAT
     H. contortus NPR-5    SYYVP------PYCGKFCDETNWQSENSRRLYGTMVMLLQQFVIPMSIITYCYFRILRKVSQDMIIQNVQFSASLSQKQRVDAISRKKKVNYILIGMVAT
      C. elegans NPR-5    SFYVQ--PGETPYCGHFCDEANWQSENSRKIYGTTVMLLQ-FVVPMAVITYCYFKILQKVSKDMIIQNAQFCQSLTQKQRSDATSRKKKVNYILIAMVVT
        S. ratti NPR-5    EAFTSSFEDNKPLCGVFCEET-WQDESTRQTYGLIALIFQ-FIVPMGIITYCYWRILHKVAKDSIIHNVQFSNSLTASQRSAAVNRKRRVNYILIAMVLA
    B. xylophilus NPR-5   AHFVP--VNSTPFCGHFCEETNWKG-EYRLFYGTTVMVFQ-FVIPLSIIMYCYSRIFAKVHKDMIIQNVQFCQSLTNSQRVDAEKRKKRVNYILIAMVAT
    G. pallida NPR-5.1    DYYVP--KNSTPFCGQFCDELNWSGDTQRRLYGTGVMLFQ-FVVPTAIITYCYWRILRKVHKDMIVQNVQFSKSLSNSQRTDALNRKKRVNYILIGMVIA
    G. pallida NPR-5.2    VYMASQDDAITPFCDTFCDEFNWESESRRQFYGAVVLLFQ-FVVPLTVITFCHLRILRKVQKDMIVQNVQFRRTLTNSQRMDAINRKRRVNYTLLGMVVA
        M. hapla NPR-5    DYYVP--RNSTPFCGQFCDELNWPDDTQRRLYGTGVMLFQ-FVLPMAIITYCYWRILRKVHKDMIVQNVQFSQSLSNTQRTDALNRKKRVNYILIGMVIA
    M. incognita NPR-5    DYYVP--RNSTPFCGQFCDELNWPDDTQRRLYGTGVMLFQ-FVLPMAIITYCYWRILRKVHKDMIVQNAQFSQSLSNTQRTDALNRKKRVNYILIGMVIA

            Consensus     AYYVP   NTTPFCG FCDEINWNGET RRLYGT VMLLQ FVIPLAIITYCY RIL KVAKDMIIQNVQFS SLSNAQR DAINRKKRVNYILIGMVIA

       B. malayi NPR-5    FIGCWLPLTVVNMLKDFKLEPLFLLEQPFFWPLLAAHVIAMSTVIWNPLLFFWLTKRKK-AHTQMPRFSVSF-NAFGTTISRISSMHRKSNAFRSTAWII
    W. bancrofti NPR-5    FIGCWLPLTVVNMLKDFKLEPLFLLEQPFFWPLL-AHVIAMSTVIWNPLLFFWLTKRKK-AQIQMPRFSVSF-NAFGTTISRISSMHRKSNAFRSTAYII
      D. immitis NPR-5    FIGCWLPLTVVNILKDFS-----------K----AHVIAMSTVIWNPLLFFWLTKRK--KKSQMPRFSISF-NAFSTKISGINSMRRKS----------
      O. vovulus NPR-5    FIGCWFPLTVVNILKDFSKEPKFLLKQPFFWPLV-AHVIAMSTVIWNPLLFFWLTKKK--TKSQMPRFSVSF-NAFGTTISRISSMHRKSYTFR------
      O. ochengi NPR-5    FIGCWFPLTVVNMLKDFSKEPKFLLKQPFFWPLV-AHVIAMSTVIWNPLLFFWLTKKK--TKSQMPRFSVSF-NAFGTTISRISSMHRKSYTFR------
          L. loa NPR-5    FIGCWLPLTVVNVLKDFRLEPSFLLEQPFFWPLV-AHVIAMSTVIWNPLLFFWLTKRKK-AQTQTPRFSVSL-NVFGTTISRINSMQRKS----------
         A. suum NPR-5    FIGCWLPLTAVNLVKDFSKEPEFLENQPFLWPLV-AHVIAMSTVIWNPLLFFWLTRKQ--KRPILGRIIASS-DALTSFVSRINSIRRTS----------
      A. caninum NPR-5    FICCWLPLTAVNLVKDYKIEPYFLRAQPFLVPLL-AHVVAMSLVVWNPVLFFWLTRKQ--KRSGLSKIIN------------------------------
 N. brasiliensis NPR-5    FICCWLPLTAVNLVKDYKIEPSFLRAQPFLWPLM-AHVVAMSLVVWNPVLFFWLTRKQ--KRSGLSKIINSS-EVVASFASRLGNSLRRMSSRNDS---K
     H. contortus NPR-5    FICCWLPLTAVNLVKDYSKEPAFLRAQPFLWPLM-AHVIAMSLVVWNPVLFFWLTRKQ--KRSGLSKIIN------------------------------
      C. elegans NPR-5    FIGCWLPLTLLNLVKDFKKEPEWLKRQPFFWAIN-AHVIAMSLVVWNPLLFFWLTRKQ--KRSGLSKILNST-EIVSSFASRVSNSIRRSTFRRN----N
        S. ratti NPR-5    FILCWFPLTLYNITKDFSK----LHSQPYFYGVV-FHSIAMSTVVLNPILFWLTLKQR----------------------------------------
    B. xylophilus NPR-5   FILCWFPITIVNLVKDFQVEPAFMTAQPYLWPLI-AHSVAMSIVVWNPLLFFWLTRKQKR--SHLGGILHTS-EIITSLTSRMQSIRSTA--GDSVRLGS
    G. pallida NPR-5.1    FIGCNTPITAVNLVKDFKQEPSWLKKQPYLWPLV-AHVIAMSTIVFNPFLFFWLTAKDKGQKGNLTGIATTTSEIITSLMSRMHSLRST---GGNEEARL
    G. pallida NPR-5.2    FIGCNAPLTAVNLAKDFGWEPEFLLRQPYLCPLV-AHLVAMSTVCWNPILFAWLGAKDKDRGSNSNAGMLSVGPSAEIIANRTPCSLAVVVG-------A
        M. hapla NPR-5    FIGCNTPITAVNLVKDFKQEPAWLKQQPFLWPLV-AHVIAMSAVIWNPFLFFWLTAKDNQKGANLSGIATAT-------ISRMHSLRSN---GQPKDEDI
    M. incognita NPR-5    FIGCNTPITAVNLVKDFKQEPAWLKQQPFLWPLI-AHVIAMSAVIWNPFLFFWLTAKDKQKGANLSGIATATSEIITSLVSRMHSLRSNN–GQSKDEDI

            Consensus     FIGCWLPLTVVNLVKDFK EP FL  QPFLWPLV AHVIAMSTVIWNPLLFFWLTKK   KRSQL RIS S      S ISRI SM R S
```

```
      B. malayi NPR-5     WHRCIPESFDGTYCSMLNREPSSRNDMQKSHLRNGLTNSKNYLTIEAERLI-----------------------------------------
   W. bancrofti NPR-5     WHRCIPESFDGTYCSTLNREPSSRNDMPKSHLRNGLTNSKNYLTVEAERLI-----------------------------------------
     D. immitis NPR-5     ----YIFRYDDIYCSSLNRRSSNRSNSTKLHLRNGSANSKNYLTVEMERLI-----------------------------------------
     O. vovulus NPR-5     --------------------------------------------------------------------------------------------
     O. ochengi NPR-5     --------------------------------------------------------------------------------------------
         L. loa NPR-5     ----YNSTYN--SAGTLNRESPNRNETPKSHLRNGLANTKNYLSVKTERLI-----------------------------------------
        A. suum NPR-5     ---TFRSQSS--ATTKVIKRQEAVDETPGSRTRPLLVTDGDRTSIARSASLSRTIL------------------------------------
     A. caninum NPR-5     --------------------------------------------------------------------------------------------
N. brasiliensis NPR-5     TEKTKKRNGAVDSEVIVGCSATSRPLIIRAELMTASS-------------------------------------------------------
    H. contortus NPR-5   --------------------------------------------------------------------------------------------
     C. elegans NPR-5     IDRVRKKQVVLDCEGSS-YTTSSRPLLIRTDVQATLSNGSTSTTREML--------------------------------------------
       S. ratti NPR-5     --------------------------------------------------------------------------------------------
   B. xylophilus NPR-5    TRIRRRTPSEKCCSRN---NSVQSRPLIVSQTDNGTTPTALLTKASSMENNML---------------------------------------
   G. pallida NPR-5.1     KRMNKRALQLQRCESAGSRRTANGTSIRLGQLSSALPLSQPLLLRENGHTATVHSRTDSSRNSPSGMVEGRRSPLAKAYSLRANSVL-----
   G. pallida NPR-5.2     IRRLSRGELTAAEPNLGQQRERKNIPMVSAHFRNKLLENGKMSSKDSVDSLPVANSKMFDGYERKLKPMHAGENPSLVVPNRRRMSTNSNTF
       M. hapla NPR-5     RYKRRFGRKAARHLYRAESETTTS---RRPVLANGLTFSDSLRLNGYGGDTQWVRKNKLFKNNNYPLKG---SNASINYIPKWTSKI-----
   M. incognita NPR-5     RYKRRFGRKTARHLYRAESETTTSTSHRRQILSNGLTFSDSL--------------------------------------------------

            Consensus                                    L  GL
```

6. NPR-6 homologues

```
  T. spiralis NPR-6    --------------------------------------------------------------------------MVVGIIVFNRRLQSVTN-FFICNLAV
     T. muris NPR-6    --------------------------------------------------------FFVILYAFVLLMAFCGNLLVIYIIVMRRRLQNVTN-FFICNLAV
      A. suum NPR-6    ------------------------------------------------------RLVFGIAYMLIFTLAVSGNSLVVYVVINNKTMQTVTN-IFITNLAI
   A. caninum NPR-6    ----------------------------------------------------------------------------------------------------
   C. elegans NPR-6    ------------------MSNDLVPSVSSILNETTPSYQSTCKIKNNPMEMEYFRPFFISMYCAVFLVASSGNFLVVYVVMTNKRMQTITN-IFITNLAV
     S. ratti NPR-6    ------------------------------------------------------RVIYLFIYLLIFIVAVTGNSLVVYVVMTKKSMQTVTN-IFITNLAI
B. xylophilus NPR-6    -------------------------------------------------------------YTTVFLISFIGNATVVYVMVIRAKTTNVMN-LLITNLAV
   G. pallida NPR-6    ----------------------------------------------AITDLVLTRSLFLIAYLSLFFVGLIGNFLVVWVVATC---------VFIANLAV
     M. hapla NPR-6    PYRRYYYRITDPPTCFQVAIAAMTIFYIFHHKFQALLSVAQARRLVALTDLLLTKVLFMIAYPSIFVVGTVGNLLVVWVVVSCKKMQTVTNTVFIANLAV
 M. incognita NPR-6    -----------PPTCS--PTLAIPIFYISHHKFQALLLVAQAKRCCVLTDLLLTKVLFMIAYPSIFVVGTVGNLLVVWVVVNCKKMQTVTNTVFIANLAV

          Consensus                                                          R LFLI Y  IFLVA  GN LVVYVVV  KKMQTVTN IFI NLAV


  T. spiralis NPR-6    SDIFVTLTSLWLTPMYGYLGKWIWGAVMCHSLPVIQGASIFINSFTLTAIALDRYIVILNPFKKR------MKMKVCFGIILAIWILSLLLVAPYGFHMD
     T. muris NPR-6    SDLFVSLTSLWLTPMYGYLGRWIWGSVMCHSVPMIQGAGIFISSLTLTAIAIDRYIVILNPFKKR------MTVKVCLTVIISIWLFSLMLVAPYGLHMD
      A. suum NPR-6    SDLLVNFTSLWLTPVYTYVGHWIWGGWLCYGLPLFQGTSIFISTLTLMAIAIDRYFVIVHHSSS-PNVNDHMSMQVCIAIITLIWTVSLLLVMPYAVHMR
   A. caninum NPR-6    ---------------------------------------------------------------------------------------VAAAGMPYAYHMR
   C. elegans NPR-6    SDIMVNFTSLWLTPTYTSIGHWIFGGGLCHGLPLFQGTSIFISTWTLTAIAIDRYIVIVHNSSN-ININDRMSMRSCLSFIVLIWLCSLLLVTPYAINMK
     S. ratti NPR-6    SDLLVNFTSLWLTPLYTFLGRWIWGGWLCYGLPLFQGTSIFISTLTLMAIALDRYFVICRHGAVKINANDHISMHVSI-IITVIWAFSLLLVMPYALHMR
B. xylophilus NPR-6    SDFLVCITSLWLTPLYWYTGQWMWGTTMCHFFPLFQGLSICNSSMTLLVIAVDRYIVVCKNQDSRYKAFNLTTKQNCKFVIAFIWSIAITLVMPYALNMK
   G. pallida NPR-6    SDLIVCCTSIWLTPTYTYIGHWIWGAWMCYALPLFQGASIFISSLTLTAIALDRHWVITSWANS---------PQVCAAVICLIWCLSVLLVLPYAVHMR
     M. hapla NPR-6    SDLLVCFSSIWLTPSYTYIGHWIWGGWLCYSLPLFQGASIFISSLSLTAIALDRYWVICLAPPNRSATQMHTSKQVCIAVICLIWCVSILLALPYAVHMR
 M. incognita NPR-6    SDLLVCFSSIWLTPSYTYIGHWIWGGWLCYSLPLFQGASIFISSLSLTAIALDRYWVICLAPPNRSATQMHTSKQVCIAVIVLIWCVSILLALPYAVHMR

          Consensus    SDLLV FTSLWLTPLYTYIGHWIWGGWLCYSLPLFQGASIFISSLTLTAIALDRYWVI      R   N  MSMQVCIAVI LIW VSLLLVMPYAVHMR


  T. spiralis NPR-6    LYHLA-ECDIYICRELWE-FHEFQAAYGFSVMALQFGIPFLIITVCYLRIWFHIFKRKMVLQLK-SDQALQRRKRLLKMLTLMVAIFGLCWLPFNVLNIL
     T. muris NPR-6    LHYSP-QCDFHICRELWE-FGELQAAYGFTVMVLQFGIPFFVIAVCYIRIWHHLCTRKSLVHLK-SDSALKRKKRLLKMLILMVSIFGLCWLPFNMLNLW
      A. suum NPR-6    MTYIPRPCEFWLCIEDWA-MEDLKSMYGVIVMALQ-LVPFVIIGFSYIQIWNFLNNRNSMVSQRESEYENARKKRLLRMLIIMVVVFGICWFPFNLLNIL
   A. caninum NPR-6    LTFLTEPCNFWVCGEDWS-MKRIKSIYGVIVMALQFIVPFVIIGISYHSIWSFLNGRRRLTRER--AVETNRKKRLLRMLVIMVVIFAVCWFPFNFLNIL
   C. elegans NPR-6    LNYIHEPCDFLICSEDWS-NAEFRSIFGIVVMILQFILPFVLIAISYIKIWLFLNSRQSMTERK---SDIKRKKRLLRMLIVMVVIFAICWFPFNLLNCL
     S. ratti NPR-6    LTKFKKPCEFWVCLEAWA-MEDLKSAYGLIVMILQFIVPFIIIGQSYIKIWKFLNNRKSLVTERESEIETQRKKHLLRMLIIMVVVFAICWFPFNLLNII
B. xylophilus NPR-6    IKKSSNLCGHYKCLESWS-TGSTRAVYGLVVTMFQFIIPMIFIGFSYMKIFMFL-QRQCQESNNRLRVVSNKKNALFRMLFVMVVAFAVCWLPVNILNVM
   G. pallida NPR-6    IAEVMWPCRFSLCVEDWGGKEDMRSLYGMTTVVLQFVVPFTVIGHSYRRIWRFMEARRLRRQSE-SDVQLMRKRRLLSMLVRMVLLFGICWLPFNFLNLL
     M. hapla NPR-6    IAQVQWPCHFSLCVEDWVDLEDLRSVYGVITAILQFVLPFAIIGRSYRLIWRFIETRRAQLLRPISEAQMNRKQRLLRMLVRMVLVFGCCWLPFNILNLL
 M. incognita NPR-6    IAQVQWPCHFSLCVEDWVDLEDLRSVYGVITAILQFVLPFAIIGRSYRLIWRFIESRRAQLLRPISEAQMNRKQRLLRMLVRMVLVFGCCWLPFNILNLL

          Consensus    L  V  PCDFWLCVEDWS MEDLRSVYGVIVMILQFIVPFVIIG SYIKIW FL  RRSLL  K SE  LNRKKRLLRMLIIMVVIFGICWLPFNILNIL
```

```
       T. spiralis NPR-6     RDVMPSHFDGKSYFVFLFLVSHLIAMSSSSWNTVLYAWMNYHFNREFRPWIHPGSWQMSQNKMTNCIKWLKRVTEAITTFLTLVETQILIDCMLQKHIIF
          T. muris NPR-6     RDLSPGAIDAKSYFVFLFLLSHLIAMSASGWNPVLYAWMNDNFSREYRYIFYFLCGRK------------------------------------------
           A. suum NPR-6     RDLHLDSSIKP-YFSFLFLSAHVISMTATCWNPIVYAWMNDAFRERFVAAVPFL----------------------------------------------
        A. caninum NPR-6     RDLHWDDFIRP-HFSFIFLIVHLISMTATCWNPILYAWMNDSFR--------------------------------------------------------
        C. elegans NPR-6     RDLKLDNFMRG-YFSFVFLSVHLMSMTATAWNPILYAFMNETFREEFAKVVPCLFARRPGTGPIRVITERTAMITNPFRRANRKKKVEEQPVTVISESPL
          S. ratti NPR-6     RDLRLDDGIKQ-YFPPLFLTSHVLSMTATCWNPIVYAWMNESFRKEFIHAIPFLQKHFQLNAKNNHRARVITDCQPALIRVNCKDNNKQK----------
     B. xylophilus NPR-6     RDIGLDQLILGRQFGIWFMRAHMLSMTGVMVNPLIYAFMNHNFRDEFCRVFP------------------------------------------------
        G. pallida NPR-6     RDLHADAFLKV---SFLFLFAHLLSMVVPAANPLLYAWMNASFRDGFLRVLP------------------------------------------------
          M. hapla NPR-6     RDLRMDAWLKP---SFLFLLAHLLSMVVPAANPLLYAWMNSAFRESFLRALP------------------------------------------------
      M. incognita NPR-6     RDLRMDGWLK----SFLFLLSHLLSMVVPAANPLLYAWMNSAFRESFLRALP------------------------------------------------

               Consensus     RDL LD FIK  YFSFLFLLAHLLSMTATAWNPILYAWMNDAFREEFLR LP

       T. spiralis NPR-6     KKYCCKVLRELNAQLLNGENAVNQWICQKPFYKMSKKFSSVKLKQLII
          T. muris NPR-6     ------------------------------------------------
           A. suum NPR-6     ------------------------------------------------
        A. caninum NPR-6     ------------------------------------------------
        C. elegans NPR-6     QTAVEPQRSIVYLDEPENGSSCQTLLL---------------------
          S. ratti NPR-6     ------------------------------------------------
     B. xylophilus NPR-6     ------------------------------------------------
        G. pallida NPR-6     ------------------------------------------------
          M. hapla NPR-6     ------------------------------------------------
      M. incognita NPR-6     ------------------------------------------------

               Consensus
```

7. NPR-8 homologues

```
T. spirallis NPR-8      --------------------------AEQCFL-----QSNGSEVGEN-FWKECLRD------QARFQLFTTTEAAFFTAAYSIICGVGLLSNGLLIYVLF
    T. muris NPR-8      --------------------------AEKCFE-----ERGFPEHDDD-FWKACLRN------LAITRVFTDVEATLFTVAYSILCGTGLLSNGLLIFVLF
     A. suum NPR-8      ------------------MFMSIDDIDAQCPRGIDKGVVTANEWRKAPFNDLCLQ----------LRRYSEWEEVIYTAIYMVISVLAVIGNGVVILAVI
  C. elegans NPR-8      MDFINELFFTDTDIEDLGMIMEVKDIDNYCDRG---ISPNASNYLTYPFDGLCLQK-FFYQLQTSLRRFTPYEEIIYTTVYIIISVAAVIGNGLVIMAVV
B. xylophilus NPR-8     ------------------MFMSIDDIDRRCPRGINASLVSDTSWQLAPFDDACLFQTFFQRMNAHLRKYNEWEEMLYTFLYFLISFFALVGNGVVILAVI
  G. pallida NPR-8      -------------------FLTVDELNRKCPRD-GLPAEDQQRWLSHPFDDSCIRS-FFLGLSAQLRRYEHWEEWFYTAMYALISALALIGNGMVILSVI
    M. hapla NPR-8      ------------------MFLSVEDIDRQCPR--AVNPNDTNNWQTFPFDDDCLKS-FFHKLTAHLRRYYIWETALYTSFYVLISICAIIGNGLVILAVV
M. incognita NPR-8      -----------------EMFLSVEDINRQCPR--AVNPNDTNNWETFPFDDNCLKS-FFHKLTAHLRRYYIWETAVYTSFYVLISICAIIGNGLVILAVV

       Consensus                          MFLSVDDIDRQCPR         N W T PFDD CLR   FF  L A LRRYT WEE LYTAIYVLISI ALIGNGLVILAVI


T. spirallis NPR-8      ASLKKRNPGDVFIINLALSNFLLAALYIPFLWLPVNEGDFTHGLFFCKFANALPGSNIFCSTLTIAVMAVDRYYAVVNMNKSQ--ISTRNNYFAILVAGL
    T. muris NPR-8      SDLKLRRPSDVFVLNLALSNFLLAALYIPFLWLPVNQGDFTHGLFFCRFANALPGSNIYCSTLTISVMAVDRYYQVVNMRRHS--VSAQRHLISVLVAGL
     A. suum NPR-8      RKREMRTNRNVLIVNLALSNLMLALTNIPFLWLPSIDFEFLYSRFFCKFANALPGSNIYCSTLTISVMAIDRYYTVKKMYAS---SHRRQCLRAVCISIL
  C. elegans NPR-8      RKKTMRTNRNVLILNLALSNLILAITNIPFLWLPSIDFEFPYSRFFCKFANVLPGSNIYCSTLTISVMAIDRYYSVKKLKIAS---NRKQCFHAVLVSLA
B. xylophilus NPR-8     RKKEMRTNRNVLIVNLALSNLMLALTTIPFLWLPSIDFEFPYSKFFCKFANALPGSNIYCSTLTISVMAIDRYYSVKQMNVG---TVNQTCVKGIILSVM
  G. pallida NPR-8      RKRSMRTARNVFIVNLALSNLMLALTTVPFLWLPSIDFDFPYSNFFCKFANALPGSNIYCSTLTISVMAIDRFYSVRSLNISSSSARPGQCTRALFISFL
    M. hapla NPR-8      RKPAMRTARNVLIVNLALSNLMLALTTVPFLWLPSIDFDFPYSNFFCKFANALPGSNIYCSTLTISVMAIDRYYSVRSIKVAGPSSRSGNCVRALLISVL
M. incognita NPR-8      RKPAMRTARNVLIVNLALSNLMLALTTVPFLWLPSIDFDFPYSNFFCKFANALPGSNIYCSTLTISVMAIDRYYSVRSIKVAGPSSRSGNCVRALLISVL

       Consensus        RKK MRT RNVLIVNLALSNLMLALTTIPFLWLPSIDFDFPYS FFCKFANALPGSNIYCSTLTISVMAIDRYYSVK MKVA   S   NCVRALLISVL


T. spirallis NPR-8      IWVVALALSLPYLLHYNLLSFRFPLEYALRFNKTEE-----IILFTTCTLRPQSCVDSGLNAED-------E-------KRCTEQVELVISILQAVFLYL
    T. muris NPR-8      IWLLAFALSLPYLLNYDLKVVHFPLEYAQRFNKSEE-----IVLFTSCALRPSSCIDSGLNPEE-------E-------KSCIQQVELVVSVLQAVFLYA
     A. suum NPR-8      IWIISFLLSFPLLVYYDTTMLYVFKDVTVFSSG------------NATKLSSYGWRQCRLAPTQRKGMEDPN--EAAKRESDAQKVQLFMSVMQALFLYV
  C. elegans NPR-8      IWIVSFILSLPLLLYYETSMLYVMREIRVVDQS------------GQEVIRSYGWRQCRLVSAG-------------RLPDITQSIQLLMSILQVAFLYI
B. xylophilus NPR-8     IWVFSFLLSFPLLIYYDTTMLYVFKDVMVYDSER-----------NTTQLRSYGWRQCRLSPGGSAADDDLNSIDADAQARMIQHFQLVMSLMQVLFLYV
  G. pallida NPR-8      IWFFSFLLSFPLLVYYRVHVLYVIKDIYVYKDTSANGHSTSANFSSQIVLQSYGWSQCRLSTG----------------------VQLGMAAMQAVMLYA
    M. hapla NPR-8      IW------------YYQINMLYVFKDVYVLKENTQ-----SEGNSSSVELRSYGWRQCRL---------------------------LGMSLMQALMLYA
M. incognita NPR-8      IWVLSLLLSFPLLIYYQINMLYVFKDVYVLKEESSK----SEGNSSSVELKSYGWRQCRLQ--------------------------LGMSLMQALMLYA

       Consensus        IWVLSFLLSFPLLLYYDI MLYVFKDV V    S           SS  LRSYGWRQCRL                        Q VQLVMSLMQALFLYA


T. spirallis NPR-8      VPLLILVVYCVKLTHFLHKADRRRQLRLLGRRKNHP------PPP-----PPITASSGG------------------IIEAGPVKTPARSTR--------
    T. muris NPR-8      IPLIVLVVYCLKLTHFLQQNEQR----IKQQRGLTK------DKL-----LGCAKADKD------------------GKLPSSAGAHSRNTR--------
     A. suum NPR-8      VPLIVLLIFNAKLTRFLQENSRQMHRSLSDGRSDDA----GTLCIVKSSDLGQTAAVR------------------------NLPARRMNVRKR------
  C. elegans NPR-8      VPLFVLSIFNVKLTRFLKTNANKMSKTRAPPKRFDRSDSHHNSLKNNNNHTSSLRSPS------------------MPSIRSSITERNKTNQRT------
B. xylophilus NPR-8     VPLVVLSIFNVKLTRFLKTNAKHMNRNRNGTLRRES----VMIQCNKSNTYAVKESPKKTRNALSLETEISTSTPPTPRVESATPAERASDRRR------
```

```
 G. pallida NPR-8    VPLVVLSIFNMKLTRFLKMNAKQLKQNSERECDSVG----VAMERRRQRGGSKASSMGGVTQRSSIATSELMLPRQVSIVTIGASSRAAEKRS-------
   M. hapla NPR-8    VPLVVLLIFNLKLTRFLEKNSKNMNNRLPKVSISATQQNRVEKVDRRTSDNESLSCAEKLMENSDGQQKGANSTKVSSLVSGGAVSAVTTNNLRKSSGGA
M. incognita NPR-8   VPLVVLLIFNLKLTRFLEKNSKNMNNRLPKISVSATQQNRVEKMDRRTSDNESLSCAEKLIENSDGQFKGANSTKSGSLVSGVAVASSTTNNLRKSSGGA

      Consensus      VPLVVLLIFNLKLTRFL NAKNMN   L   R        V     RS      SS                      IV   A A A T R R

T. spiralis NPR-8   -----------AIKLLFAMAASYALVWMPFEVLSLYIHLRSSHWTEDTVTKVMRVDQAFKLITVLSICINPIIYGFLNKNLNKR----------------
   T. muris NPR-8   -----------AIKLLFAMASSYALVWMPFQGLSLFISLRSSDWPEDTVAFIRRIDEAFKLISMLSICINPVIYGFMNKNLSKR----------------
    A. suum NPR-8   ---NR------TTALLIAMAASYAVLWLPFTVVSMLLDLRILYMEGNGAAVIERIDQSCKMISIMSICVNPFLYGFLNTNFRHEFTDIFNQWICYKLRTP
 C. elegans NPR-8   ---NR------TTSLLIAMAGSYAALWFPFTLITFLIDFELIINQDYVN-LVERIDQTCKMVSMLSICVNPFLYGFLNTNFRHEFSDIYYRYIRCETKSQ
B. xylophilus NPR-8 ---NR------TTMLLIAMAGSYAVLWFPFTLVSMLIDMDILYLEN-GAAIIERIDQSCKLISILSIGVNPFLYGFLNTNFRHEFTDIFNSLIRCSASQR
 G. pallida NPR-8   ---NR------TTALLIAMAGSYAVLWLPFTLVSILIDLDWLGVES-GSPIVERIDQTCKLLSITSICVNPFLYGFLNTNFRHEFNEIFSYCLAWMPTLT
   M. hapla NPR-8   LINNQQKRRNKTTSLLIAMAGSYAFLWFPFTLISVLIDLDLMVNI------MERVDQACKLISILSICINPFLYGFLNTNFRSEFNEIFIKCIHC-----
M. incognita NPR-8   LINN---------SLLIAMAGSYAFLWFPFTLISVLIDLDLMVN-------MERVDQACKLISILSICVNPFLYGFLNTNFRSEFNEIFIKCIHC-----

      Consensus         NR      TTSLLIAMAGSYAVLWLWFPFTLISLLIDLDLL        IIERIDQACKLISILSICVNPFLYGFLNTNFRHEF DIF   I C

T. spiralis NPR-8   ----------------------------------------------------------------------------
   T. muris NPR-8   ----------------------------------------------------------------------------
    A. suum NPR-8   TNVQQEYSTIASPRRQSSSHSPQVSTSNRFLHLLQNPYVRRSSAPCSSFIRTSIARLT------------------
 C. elegans NPR-8   PAGRFHHDVSSIAHHRQDSVYNDEATLLTTGRQSNGKDGSSSPIGFRSSVRVCSGQTKMIGDRIVLDDDIEKDSFV
B. xylophilus NPR-8 P---------------------------------------------------------------------------
 G. pallida NPR-8   MREGRQRETSSSLGADRK----------------------------------------------------------
   M. hapla NPR-8   ----------------------------------------------------------------------------
M. incognita NPR-8   ----------------------------------------------------------------------------

      Consensus
```

8. NPR-10 homologues

```
 T. spiralis NPR-10    MTRAEVESTTLLIKAPLVVNADSRQLLPAVSQNSCVEYSDYVYEIHKDLTREGWIRGIFLICYLVIFIAGITGNVCVIISVARKASLQSVRNLFIVSLSC
    T. muris NPR-10    ----------------------------------CTDYGEFAYETYGDLTREAWIRGIFIFIYVIIFVAGITGNSCVIISVARKAFLQSVRNLFIVSLSC
   B. malayi NPR-10    ------------------------------TQTPCVDINEYLWMNHSDLTSLLPTMIFFFAIYGLIILS-------LIR----HKSLQSVRNLFIISLSV
W. bancrofti NPR-10    ----------------------------------CVDINEYLWLNHSDLTSLLPTMIVFFAIYGLIIIQ-------------------SVRNLFIISLSV
     A. suum NPR-10    -----------------------------NTTPECIDIKEYLWKNQSDLTSLPSTMSVFAVIYSFIITLGVLGNTLVVLSVIRHKSLQSVRNMFIVSLSV
  C. elegans NPR-10    ----------------------------MSSSNHCIDIRAYLWQTKHDLTLHPIPIAILATIYTIIVVVGVTGNLLVVMSVMRFKVLQSVRNMFIVSLSV
    S. ratti NPR-10    --------------------------QMINESKVCKSVNDDLRENFNDVTTQPLIMIIFSIFYLIIIIVGVVGNSLVIISVLQNKILQSVRNFFIVALSC
B. xylophilus NPR-10   ----------------------------LSKSANCVDIKAFLWTSQGDLTSLPSTMLIFLILYGVLGNG--LVIVLIILSVIRHKSLQSVRNLFIVSLSC
  G. pallida NPR-10    --------------------------------------KAYLWAQHSDVTSLVPVMLLFGLIYTTIIVTSILGNLLVIFSVCQHRSLQSVR---------
    M. hapla NPR-10    ----------MEQQPETAIDSIYSTQFVNTNDDKCIDIKEFLWTNSSDLTSLPVVMGFFAFLYSAIVLTSLLGNLLVILSVCQYRSLQSVRNLFIVSLSV
M. incognita NPR-10    ----------------------MEQQFINTEEDQCIDIKEFLWTNSSDLTSLPIVMGFFAFLYSAIVLTSILGNILVILSVCQYRSLQSVRNLFIVSLSV

           Consensus                                     CIDIKEYLW N SDLTSLP IM IF  IY IIII  ILGNLLVILSV  HKSLQSVRNLFIVSLSV

 T. spiralis NPR-10    SDIVVCLTSLPITPITIIRKNWLFGLPLCYIFPLLQCMSTIISTFTLMAIAIDRFILIIYPTKPPLNRCHATTMIVAIWTLAISISIPMLLHYNLYKIPY
    T. muris NPR-10    SDIVVCLTSVPITPITIMKKNWIFGLPLCYIFPLLQCMSTIISTFTLMAIAIDRFILIVYPTKQPLQKRHAVAMIVSIWTLAGGISIPMLLHYNLYKLPY
   B. malayi NPR-10    TDIIISIVSGTVTPITAFSKIWIFGELLCYFIPLFQGASLCFSSLTLTAIAIDRYILIIFPTKRPIQKRQAIKIIGLDFALATAISLP---------MFI
W. bancrofti NPR-10    TDIIISIVSGTITPITAFSKIWIFGELLCYFIPLFQGASLCFSSLTLTAIAIDRYILIIFHTKRPIQKPQAVKIIGLNFALATAISLPI--------MFI
     A. suum NPR-10    SDIVISIVSGSITPITAFTKIWLFGEFLCYLVPLFQGASLCFSTLTLMAIAIDRFILIVYPMKRPIQKVHALWMIALNITLATAISMP---------MFL
  C. elegans NPR-10    SDIFVAIVSGSVTPITAFSKVWLFGGPLCHLLPLLQGTALSFSTLTLTAIAIDRYILICHPTKEPIRKDQALKMISFNSAISVGLSVP---------LFM
    S. ratti NPR-10    SDIIVCVVSGSFTPISAFSKTWLFGEFLCKLVPFIQGVSLFFSTFILASIAVDRFILIIFPTTQPISKKLAIVMIISYIFIGSILTKP---------IYD
B. xylophilus NPR-10   SDIVASLVSGSVTPISAFTKIWLFGETLCKFVPLIQGSSLCFSTLTLTAISIDRFILIIYPTKRSIQTKHALRMIALNCLIATSISLP---------MVF
  G. pallida NPR-10    -N------------MFAFSKIWLFGELLCKLVPVFQGVSLCFSTLTLSAISIDRFRLIVTPLRQSIQKRCACRIVALNSAIALIISLP---------MLF
    M. hapla NPR-10    SDIVICVVSGTITPIVAFTKIWPFGELLCKWVPVIQGVSLCFSTLTLTSISVDRFLLIVTPLRPSIQKCCACRIVALNCAIALAVSFP---------ILF
M. incognita NPR-10    SDIVISVVSGTITPIVAFTKIWPFGQLLCKWVPVIQGVSLCFSTLTLTSISVDRFLLIVTPLKPSIQKCCACRIVALNCAIALAISIP---------ILF

           Consensus   SDIVISIVSGSITPITAFSKIWLFGE LCYLVPLIQGVSLCFSTLTLTAIAIDRFILIIYPTK PIQK  ALRMIALN AIA AISLP         M F

 T. spiralis NPR-10    HQDGNVTVWYCGYFCDETWPNLE---ARQAYGSIVLVLQFIIPLIVITFCYASISLRVGKGVALRQG---------------------------------
    T. muris NPR-10    ERRANHTIWYCGYFCDETWPNVE---ARQIYGGAVLLLQFIIPLIVITVCYGGIFLQVIKGVAFRQTT--------------------------------
   B. malayi NPR-10    KQRFIKYENFCGQFCTEDWSSDN--MGRSIYGTVVFIFQFVAPLTIIFFCYTMISLKLTKTTLFKKQT--------------------------------
W. bancrofti NPR-10    KQRFIKYENFCGQFCTEDWDSDN-VGRSTYGGTVVFIFQFVAPLTVIFFCYTMISYR-------------------------------------------
     A. suum NPR-10    MQKLVDYGNFCGQFCTEDWGNNE--FGRSTYGTVVFILQFVVPLIVITFCYTMISLKLNKGLLVKQAS--------------------------------
  C. elegans NPR-10    KQELMQFRNYCGEYCSENWGPDA--YLRSVYGTVVFIIQFVFPLITITFCYASISIKLRRGVFVRGSQ--------------------------------
    S. ratti NPR-10    ENTLESYKDYCGKICYEKWNGNE--DGRKRYGTIVFIVQMVIPFIIISACYIMISLKLKKVSILRRTN--------------------------------
B. xylophilus NPR-10   KQKLVDYGDFCGQFCTEDWKEDT--FARSAYGTLVFILQFVVPFVIITFCYMMISIRLGQGILVKDRN--------------------------------
  G. pallida NPR-10    TQKLDTYDRFCGQFCSEYWDGELGAVARSAYGTFVFFAQFVIPFLIISSCYVMISIKINSGVLIKRAS-TEITKFNDMSTSGSKITNLSDPRSAGRHSSV
    M. hapla NPR-10    NQTLVDWPPFCGQFCTEDWSNSN--IQRSLYGTFVLFTQFVFPFLIITSCYVMISIKINSGMHAKRCSSTELAKFSDYGSS-------NTRCDKFLSSSC
M. incognita NPR-10    NQAMVDWPPFCGQFCTEDWSNSS--IQRSLYGTFVLFTQFVFPFLIITSCYVMISIKINSGMRTKRCSSAEVAKFSDYCSS-------NVRCDKFLSSSF
```

```
            Consensus    QKLV Y  FCGQFCTEDW       ARS YGTVVFILQFVIPLIIITFCY MISIKL KGV VK  S

   T. spiralis NPR-10   -------------------GKMLLSNGAGAPDALLTRHKVALKRRQRTNRMLISMVAVFVACWFFQVLLNVLRDFNATPLAIASQPYLSSLIVHCIAMSS
      T. muris NPR-10   -------------------DHRLLPMNGVSSEVILLRQKMAIRRRQRTNWMLISMVAVFVACWFFQVLLNVLRDFSAIPSPIAAQPYLFSLIVHCIAMTS
     B. malayi NPR-10   -----------------------------------FYRQQVLKRRLRTNRMLIAMVGVFVCCWMPAVVFNFLRDYHWLPNFISQQEYLIGIITHCISMSS
  W. bancrofti NPR-10   -----------------------------------QQ---VLKRRLRTNRMLIAMVGVFVCCWMPAVVFNFLRDYHWLPNFVSQ-EYLIGITTHCISMSS
       A. suum NPR-10   -------------K-----------HASGLG---TEQRRLALQRRLRTNRMLMAMVGVFLCCWMPTVAFNFLRDYRWLPSFVAQQEYLFGIITHCISMSS
    C. elegans NPR-10   ----------K--------------E------LMSEARRQLTQRRLRTNRMLIIMTVTFALSWLPSVGFNFLRDYSALPGIIDSQDYLFGIIFHCISMTS
      S. ratti NPR-10   ----------------------------------------KNRR-QRTNRMLMAMVLVFVFCWTPTVAFNFLRDYQKLPIFIQKQEYLFGILTHFISISS
 B. xylophilus NPR-10   -------------T-----------DQQQLTQQQSEQRKTALKRRLRTNRMLIAMVAVFLLCWTPAVLFNFLRDYQWLPNVVARQEYLFGVITHCISMSS
    G. pallida NPR-10   GTEECTLLLTTNGTNDAHSGTPGTNHANDVLQMAMEQKKAILNRRLRTNKMLIAMVALFFCCWAPTVLFNFLRDNQWLPKFVLSQEYLFGIITHCISMSS
      M. hapla NPR-10   GTDEGNSLYIN----EQKQQNGMLMAGDGAMKVAMDQRKMVLSRRIRTNRMLIAMVAVFFCCWAPSVIFNFLRDYQWLPKWVVAQEYLFGTSTHCLSMSS
  M. incognita NPR-10   GTDEGNSLYIN----EQKLQNGMLMAGDGAMKVAMDQRKMVLSRRIRTNRMLIAMVAVFFCCWAPSVIFNFLRDYQWLPNFVIAQEYLFGTTTHCLSISS

            Consensus                                      EQRK VLKRRLRTNRMLIAMVAVFVCCW PSVLFNFLRDYQWLP FVAAQEYLFGIITHCISMSS

   T. spiralis NPR-10   TLWNPILYAWLNDTFRTAFYEILPFMSVLCCISKPAAMEG-RFRSDSIID------------------------------------
      T. muris NPR-10   TLWNPVLYAWLNESFRTAFFEILPCVRVLCCAKRPHINDFSLYRAASVAE------------------------------------
     B. malayi NPR-10   TIWNPCLYTLLNDQFRMAFLDLLHSFRIVP--------------------------------------------------------
  W. bancrofti NPR-10   TIWNPCLYTLLNDQFRMAFLDLLQSFRIV---------------------------------------------------------
       A. suum NPR-10   TVWNPCLYALLNEQFRLAYVDLLQYCRSGQKRCGTRYTDARCSRLVSNAFLANNSITEENIKLPNSV-------------------
    C. elegans NPR-10   VIVNPFLYGYCNEHFRAAFAALLDTVKAACGMRRVSPGNPACSQLLSTHFESTTRRSVTTTIPSSI--------------------
      S. ratti NPR-10   TVWNPCLYALLNVQFRTAFSQILGHTTSVTLNERKNLEMK----------------------------------------------
 B. xylophilus NPR-10   TVWNPILYTMLNEQFRLAFAEFFKLCRPRKPR-GLRYKSANGSNESRTINTKLKSARFSNTPTI----------------------
    G. pallida NPR-10   TIWNPVLYALLNEQFRAAFSEQFKQLRHRFCLPCRDSTNSGRNRLGTMDNNGSTSAAQQQQSCRGSIRLVSIPTPSLIKFEHSVVR
      M. hapla NPR-10   TVWNPVLYALLNEQFRLAFSEIFKQIGNYFGIKQEMKVPQMQGRLRRIKVKSDVKRGS----------------------------
  M. incognita NPR-10   TVWNPILYALLNEQFRLAFSEIFKQISNYLGIKKERKMLHMQGQLKRIKVRSDVQRGSEAI-------------------------

            Consensus   TVWNPILYALLNEQFRLAFSEIL  IR
```

9. NPR-11 homologues

```
     B. malayi NPR-11  ---------------------------------------------------------NKVDCTVAKWKY---LILQLG-------------WCITAAYVV
  W. bancrofti NPR-11  ---------------------------------------------------------------MNIWEY---LILQLG-------------WCITAAYVV
    D. immitis NPR-11  ---------------------------------------------------------------------------QLG-------------WCITIAYII
    O. vovulus NPR-11  ---------------------------------------MKDDCDEWREIFTRIDSYFRDDIVLEGTEYSKTCIFQLG-------------WCITVAYII
    O. ochengi NPR-11  ---------------------------------FIEVSLKITYFRSLKRRNFWDMIMNLTSNMEKKNEYSKTCIFQLG-------------WCITVAYII
        L. loa NPR-11  ----------------------------------MPSIRMKDNCDEWRQIFTRIDSYFRDDIVLEGTNH---ASAQLG-------------WCITVAYVV
       A. suum NPR-11  ---------------------------------MPNSTLAEKDCNQWQHMFSEINSYFRNEIVLEGTDH---ASVELG-------------WCITFAYCF
    A. caninum NPR-11  ----------------------------------------------------------------------------------------------------
N. brasiliensis NPR-11  ----------------------------------------NETCEHYQNLFSRINHYFRDDIVLDGAEHA---SKELG-------------YAITAAYLV
  H. contortus NPR-11  ----------------------------------------NETCEHWQSLFGRINHYFRDDIVLNGTEHASKKVG----------------YAITAAYLV
    C. elegans NPR-11  ------------------------------------MGSVNESCDNYVEIFNKINYFFRDDQVINGTEYS---PKEFG-------------YFITFAYML
      S. ratti NPR-11  ------------------------------------------SCAQAIEVFEKLNYYFVDEPIIANHKRAPG-------------------VIIAFAYSM
  B. xylophilus NPR-11  --------------------------MADPNRSNSSEPSEARTCAQWERLFSAINHYFRDDDILQGSEHSSVESG----------------YVIVAAYVL
    G. pallida NPR-11  ----------------------------MSGDGMTPNRTGQQNCGELETLFDGMNEFFREETIIQGSEHSSILASPRESEELMNFGIFQLGNLILSAYIL
      M. hapla NPR-11  MSDVLDINAYNNSENDASYKVASSSILILSEQPQLQEQNQVKSCHEMETLFSGLNNFFRDEDILKESEHSSELVG----------------IIIFTAYLL
M. incognita NPR-11.1  ----------------------------------------------------------------------------------------------------
M. incognita NPR-11.2  MSNVLDINTYNNSK-----NGNTSSIIILSEHSQQQEQ-KLEKCHEMEALFSGLNNFFRDDDILKESEHSSELLG----------------NLIFTAYLL

            Consensus                                             C     LF  IN YFRDD VL  TEHS       G             W IT AYIL

     B. malayi NPR-11  VILFGAFGNMLTIIAVLYNPQIRTTRNFFILNLALSDFFICTIAAPITFYTVLYIFWPFGLAMCKITGSLLGFGIFLSTFSIAAIALDRCVVIIFPTKRK
  W. bancrofti NPR-11  VILFGAFGNMLTIFAVLYNPQIRTTRNFFILNLALSDFFICTIAAPITFYTVLYTFWPFGLAMCKITGSLLGFGIFLSTFSIAAIALDRCVVIIFPTKRK
    D. immitis NPR-11  VILFGTFGNMLTIIAVLYNPQIRTTRNFFILNLALSDFFICTIAAPITFYTVLYTFWPFGLTMCKITGSLLGFGIFLSTFSIAAIALDRCVVIIFPTKRK
    O. vovulus NPR-11  VILFGTFGNMLTIIAVAYNPQIRTTRNFFILNLALSDFFICTIAAPITSYTVLYTFWPFGLTMCKITGSLLGFGIFLSTFSIAAIALDR-VVIIFPTKRK
    O. ochengi NPR-11  VILFGTFGNMLTIIAVVYNPQIRTTRNFFILNLALSDFFICTIAAPITSYTVLYTFWPFGLTMCKITGSLLGFGIFLSTFSIAAIALDR-VVIIFPTKRK
        L. loa NPR-11  VILFGTFGNMLTIIAVLYNPQIRTTRNFFILNLALSDFFICTITAPITSYTVLYTFWPFGLAMCKITGSLLGFGIFLSTFSIAAIALDRYVVIIFPTKRK
       A. suum NPR-11  VIVFGALGNMLTVLAVIRNPQMRTTRNFFIVNLALSDFFICTVTAPITLYTVLYTFWPFGTATCKIAGSLQGFGIFLSTFSIAAIALDRYVLVIFPTKRK
    A. caninum NPR-11  ----------------------------------------------------LYMFWPFGTALCKIAGSLQGFNIFLSTFSIAAIALDRYVLVIFPTKRE
N. brasiliensis NPR-11  VIIFGACGNFLTMLVVALNPTMRTTRNFFIFNLALSDFFVCTITAPMTLYTVLYMFWPFGTALCKIAGSLQGFNIFLSTFSIAAIALDRYVLVIFPTKRE
  H. contortus NPR-11  VIIFGACGNFLAMLVVVLNPTMRTARNFFIFNLALSDFFVCTVTAPMTLYTVLYMFWPFGTALCKIAGSLQGFNIFLSTFSIAAIALDRYVLVIFPTKRE
    C. elegans NPR-11  IILFGAIGNFLTIIVVILNPAMRTTRNFFILNLALSDFFVCIVTAPTTLYTVLYMFWPFSRTLCKIAGSLQGFNIFLSTFSIASIAVDRYVLIIFPTKRE
      S. ratti NPR-11  VIIFGAFGNILTIFIVLRNKSMRTVQNFFIINLALGDFMICTITAPITFFNISFTFWKYGNALCKFVSSIGGFNIFLSTFSITAIALDRYFLVIFPTQQQ
  B. xylophilus NPR-11  VISLGIFGNALTIVAILRNKQMRNVRNFFILNLALSDFFICTVTAPITLYTVLYMFWPFGTALCKIAGSLQGFNIFLSTFSITAIALDRYVLVIFPTKRE
    G. pallida NPR-11  VIVFGTVGNILTILAVLRNKQMRTVRNFFIVNLALSDFFVCTVTAPTTLYTVLYVFWPFGTTLCKIAGSLQGFNIFLSTFSITAIALDRYVLVIFPTKRQ
      M. hapla NPR-11  VIAFGSFGNLLTIMAVLRNKQMRTVRNFFILNLALSDFFVCTVTAPTTLYTVLYVFWPFTRPLCKIAGSLQGFNIFLSTFSITAIALDRYVLVIFPTKQQ
M. incognita NPR-11.1  -------------------------RNFFILNLALSDFFVCTVTAPTTVYTVLYVFWPFSRPLCKIAGSLQGFNIFLSTFSITAIALDRYVLVIFPTKRQ
M. incognita NPR-11.2  VIAFGSIGNLLTIMAVLRNKQMRTVRNFFILNLALSDFFVCTVTAPTTVYTVLYVFWPFSRPLCKIAGSLQGFNIFLSTFSITAIALDRYVLVIFPTKRQ

            Consensus  VILFGAFGNMLTIIAVL NPQMRTTRNFFILNLALSDFFICTITAPITLYTVLYMFWPFG ALCKIAGSLQGFNIFLSTFSIAAIALDRYVLVIFPTKR
```

```
        B. malayi NPR-11     RQHNLALLLFFLIWVISITLALPLLITSDLNTVFDDDKCGISLKICHEKNEIWNLMPISKRAYTLAVLLTQYALPLISIVFAYSTIAMKMQIRMNSRLA-
     W. bancrofti NPR-11     RQHNLALLLFFLIWVISITLALPLLITSDLNTVFDDDKCGISLKICHEKNEIWNLMPISKRAYTLAVLLTQYALPLISIVFAYSTIAMKMQIRMNSRLA-
       D. immitis NPR-11     RQHNLALLLFILIWVISITLALPLLITSDLNTVYEDDKCGIYLEICHEKNEIWNLMPISKRAYTLGVLLTQYALPLISIVFAYSTIAMRMQIRMNSRLA-
       O. vovulus NPR-11     RQHNLALLLFFLIWVISITLALPLLITSDLNTVFEDDKCGISLKICHEKNEIW--MPISKRAYTLAVLLTQYAIPLISIVFAYSTIAMRMQIR-------
       O. ochengi NPR-11     RQHNLALLLFFLIWVISITLALPLLITSDLNTVFEDDKCGISLKICHEKNEIWN-MPISKRAYTLAVLLTQYAIPLISIVFAYSTIAMRMQIRM------
           L. loa NPR-11     RQHNLALLLFSLIWIISITLALPLLITSDLNTVFDDDKCGISLKICHEKNEIWNLMPISKRAYTLAVLLTQYALPLISIVFAYSTIAMRMQIRMNSRLA-
          A. suum NPR-11     RQHNLSLLFFSLIWLISIALALPLLIASDLNTIFEDHNCDISLQICHEKNEIWQQMPIGKEAYTLAVLVTQYALPLISIVFAYTRIAHRMQGQFAIRMS-
       A. caninum NPR-11     RQQNLSLMFFVFIWVLSLILAIPLLIASDLNTIYEDPVCEISLRICHEQNDIWQTMMISKQTYTLSVLVTQYAFPLFSLVFAYTRIAHRMKLRFANR---
  N. brasiliensis NPR-11     RQQKLSLMFFVFIWVLSLILAVPLLIASDLNTIFVEPSCGISLRICHEQNDIWQTMMISKQTYTLSVLVTQYAFPLFSLVFAYTRIAHRMKLRFANR---
      H. contortus NPR-11     RQQNLSLMFFVLIWVVSLVLAVPLLITSDLNTIYEDSSCDISLRICHEQNEIWQ-MMISKRTYTLSVLATQYAFPLFSLVFAYTRIAHRMKLRFANR---
       C. elegans NPR-11     RQQNLSFCFFIMIWVISLILAVPLLQASDLTPVFVEPSCDLALYICHEQNEIWEKMIISKGTYTLAVLITQYAFPLFSLVFAYSRIAHRMKLRFANR---
         S. ratti NPR-11     RQKFFSIILFALIWIISLLLSIPIVIVSSSTMFYEVEFCNLTLYLCNENELAWDKMPFSKLSYTISIIVIHYAFPIVSMVFAYTKIASRMHSRFVHRN--
    B. xylophilus NPR-11     RQHNLSLLFFSLIWLISILLALPLFDASDLNIIFQDKNCGISLTICHEQNEKWQKMAITKEVYTVGVLFVQYAFPLTSIAFAYSRIARRMGARFAARN--
       G. pallida NPR-11     RQQRLSLMFFSMIWLASILLAAPLFLAADLRPVFRDNRCQIELDLCSEQNERWKELSLSKESYTLGVIILQYAFPLAAIAFAYSRIARRMGARRARGGSG
         M. hapla NPR-11     RQQNLSIIFFSLIWLASILLAAPLFLAADISPIFHDVQCGIELKLCNEQNERWRELLLSKEAYTGGVIILQYLLPLASIAFAYSRIARRMGARKSCRCGS
  M. incognita NPR-11.1     RQQNLSLIFFSLIWLASILLAAPLFLAADISPIFHDSQCGIELNLCNEQNERWKELLISKEAYTVGVIILQYLLPLASIAFA-SRIARRMGTSKSFRCAS
  M. incognita NPR-11.2     RQQNLSLIFFSLIWLASILLAAPLFLAADITPIFHDSQCGIELNLCNEQNERWKELMINKEAYTVGVIILQYLLPLASIAFAYSRIARRMGTRRSCRYAN

             Consensus     RQ NLSLLFF LIWVISILLALPLLIASDLNTIFED  CGISLKICHEQNEIWN M ISK AYTLAVLLTQYALPL SIVFAYSRIA RM IR A R

        B. malayi NPR-11     ----------------------------------------------------------------------------SSTFTTNAINNERRKSVAGRQRRT
     W. bancrofti NPR-11     ----------------------------------------------------------------------------SSTFTANVINNERRKSVAGRQRRT
       D. immitis NPR-11     ----------------------------------------------------------------------------TSTFTANAINNERRRSVAGRQRRT
       O. vovulus NPR-11     ----------------------------------------------------------------------------------------------------
       O. ochengi NPR-11     ----------------------------------------------------------------------------------------------------
           L. loa NPR-11     ----------------------------------------------------------------------------SSTFTANANNNERRKSVAGRQRRT
          A. suum NPR-11     ----------------------------------------------------------------------------SCANSLATMSHRRR-SVVERQRRT
       A. caninum NPR-11     ----------------------------------------------------------------------------NISLP-------------------
  N. brasiliensis NPR-11     ----------------------------------------------------------------------------NISFPNSLSQNQRRRSVVERQRRT
      H. contortus NPR-11     -------------------------------------------------------------------------------------------NIPFSSSLT
       C. elegans NPR-11     ----------------------------------------------------------------------------NQNVTTNTNTSQRRRSVVERQRRT
         S. ratti NPR-11     ----------------------------------------------------------------------------NIKLIGEDANN-RRKHVAERQRRT
    B. xylophilus NPR-11     ---------------------------------------------------RHSR------------------------------------SIADRHRRT
       G. pallida NPR-11     GGGTAGNQPNTFRKSSGTVGFQTLTTNCFNAPEVTKQSNGQTVTLTTNLSTAASQKATKTSISSNGE----------VKNAAEALISQRRKSVADRHRRT
         M. hapla NPR-11     GGGVDS------RKNSVYLNTPVFS-NRSSLCRQIAAGGATSPFSASDNGHQNSK----LVIFTNDETNCN--LLQNVNPSAEILLNQRRKSIAVRHRRT
  M. incognita NPR-11.1     GG-RDS------RKNSVFLNTPVLGSNRSSFSRQIASGGATSPFIASDNGHKHSSNKQPLVISTNGEYSQTNGNLHNVNPSAEILLKQRRKSIADRHRRT
  M. incognita NPR-11.2     GG-RDS------RKNSVFLNTPVFGSNRSSFCRQIASGGATSPFIASDNGHKHSSNKQPLVISTNGEYSQTNGNLQNVNPSAEILLKQRRKSIADRHRRT

             Consensus                                                                                     T       RRKSVA R RRT

        B. malayi NPR-11     HLLLICVVIVFAVAWLPLNVFH----VLNTFGIVE-FSVPLFAICHLIAMGSACLNPVSYAFFNQNFRQQFIMFYEAAIRKNLQIFGWITAVRNMR-DGV
     W. bancrofti NPR-11     HLLLICVVIVFAVAWLPLNVFH----VLNTFGIVE-FSVPLFAICHLIAMGSACLNPVSYAFFNQNFRQQFVMFYGAAIRKILQIFGWITPARNMR-DRV
```

```
    D. immitis NPR-11     HLLLICVVTIFAVAWLPLNVFH----VLNTFGIVE-FSVPLFAICHLIAMGSSCLNPVSYAFFNQNFRQQFVMFYDAAIRKILQISDLVTPTRYKR-DRM
    O. vovulus NPR-11     --------------WLPLNVFH----VLNTFGIVE-FSVPLFAICHLIAMGSACLNPVSYAFFNQNFRQQFVMFYD------------------------
    O. ochengi NPR-11     --------------WLPLNVFH----VLNTFGIVE-FSVPLFAICHLIAMGSACLNPVSYAFFNQNFRQQFVMFYD------------------------
        L. loa NPR-11     HLLLICVVIVFAVAWLPLNVFH----VLNTFGIVE-FSVPLFAICHLIAMGSACLNPVSYAFFNQNFRQQFIMFYEAAIRKILQTFDWVTPARNKH-DRV
       A. suum NPR-11     HLLLMCVVIMFAVAWLPLNVFH----VLNTFGIVKGFSVPLFALCHVIAMGSACLNPVSYAFFNHNFRQQFVAMFEQAGLSRLRGILLHLRKKCMLGGRR
    A. caninum NPR-11     ----------------------------------------------------------------------------------------------------
N. brasiliensis NPR-11    HLLLMCVVAVFAIAWLPLNVFH----MVNTFEWVSLFSVSTFAVCHVAAMCSACLNPLIYAFFNHNFRTEFIALFERLGFRKLRDVVFSWDEERERKKGM
   H. contortus NPR-11    HLLLMCVVAVFAIAWLPLNVFH------NTFEWVSLFSVSTFAICHVAAMCSACLNPLIYAFFNHNFRTEFIRLFEKLGLR-------------------
     C. elegans NPR-11    HLLLVCVVAVFAVAWLPLNVFH----IFNTFELVNSFSVTTFSICHCLAMCSACLNPLIYAFFNHNFRIEFMHLFDRVGLRSLRVVIFGEQESLKKSMRT
       S. ratti NPR-11    NFLLASLVLLFAIAWLPFNIFH-----MLLSLKVITYSGTLFALCHLMGMMSACLNPVCYAFFNQNFRNELFIILHKLKLRKNSNINGINRTEYSATVKR
  B. xylophilus NPR-11    HFLMVTLVVVFASAWLPLNIFH---LIN-TFNIVQRFSVPTFAFCHVTAICSACLNPLSYAFFNQNFRTEFVAMFESLGL--------------------
     G. pallida NPR-11    NLLLVTLVVVFAFAWLPLNIFH---LVN-TFSSSASFSVPVFALCHLTAMCSACLNPCCYAFFNQNFRQEFLAIYHKLGLIWLYRRFSLFGKSIGLNGDS
       M. hapla NPR-11    NMLLVTLVVVFACAWLPLNIFHAYTLLFNKFSKTASFSVPIFALCHLTAMCSACLNPCCYAFFNNNFRQEFIAMYRKLGLM-------------------
 M. incognita NPR-11.1    NMLLVTLVVVFACAWLPLNIFHAYTLLFNKFSKTASFSVPIFAICHLTAMCSACLNPCCYAFFNNNFRQEFLAMYRKLGLITLYRCFARFTKRLCPSTEI
 M. incognita NPR-11.2    NMLLVTLVVVFACAWLPLNIFHAYTLLFNKLSKTASFSVPIFALCHLTAMCSACLNPCCYAFFNNNFRQEFLAMYRKLGLTTLYRCFVRFTKRLCPSTEI

             Consensus    HLLLV VVVVFAVAWLPLNVFH    VLNTF IV  FSVPLFAICHLIAM SACLNPV YAFFNQNFRQEFV MYE LGL  L


     B. malayi NPR-11     KYSRSAKSVIT--------TSAINAQLVGRGDNDEKSEEAKIDLNDH-----------------------------------
   W. bancrofti NPR-11    KYSRSAKPVITTQ-----HTSAINAQFVGRGDNDEKSEEAKIDLNDH-----------------------------------
    D. immitis NPR-11     KYNQSPRNAIMQ------HTSTFNVKYMGKGDKDVKLDKAEIHLNN------------------------------------
    O. vovulus NPR-11     ----------------------------------------------------------------------------------
    O. ochengi NPR-11     ----------------------------------------------------------------------------------
        L. loa NPR-11     KYNRSANAVITTQ-----HASTINAKFMGKCDDNEKPDEAKIDLNDH-----------------------------------
       A. suum NPR-11     KYAATSCAVASSKSPRMLPTTVHNEEFNEEHDAAPKESEFILRINMVNTV--------------------------------
    A. caninum NPR-11     ----------------------------------------------------------------------------------
N. brasiliensis NPR-11    EREVKSSCPTAVHTVRTSVAERLDESVALHLDTDEQL---------------------------------------------
   H. contortus NPR-11    ----------------------------------------------------------------------------------
     C. elegans NPR-11    EFRSRGGCKTVTTAEPATFQRMNESMILSAMEQDEQL---------------------------------------------
       S. ratti NPR-11    STC-------------------------------------------------------------------------------
  B. xylophilus NPR-11    ----------------------------------------------------------------------------------
     G. pallida NPR-11    SDKKTKWRPNR-----------------------------------------------------------------------
       M. hapla NPR-11    ----------------------------------------------------------------------------------
 M. incognita NPR-11.1    NKEKSVDATQSNENKELGTSPSENEANQHLQQTIQGVLKNSKEKSCEVLNVKMENQTKNPLELCETLIEKPPNLAKTIQMDL
 M. incognita NPR-11.2    SK--------------------------------------------------------------------------------

             Consensus
```

10. NPR-12 homologues

```
     B. malayi NPR-12   -----------------------------------NNFLWQHRGGS-YVLLLYGLVCIFGTLANLFVLIAFLRTSHIRNLRNYFIANLAMSDFLLCVVTA
  W. bancrofti NPR-12   -----------------------------------NNFLWQHRGGS-YVLLLYGLVCIFGTLANLFVLIAFLRTSHIRNLRNYFIANLAMSDFLLCVVTA
   O. volvulus NPR-12   ------------------------------LHKFQNSFLWQHRGEN-YVLLLYGLLCSFGTLANLFVLAAFLRTSHIRNLRNYFIANLAASDLLLCVVTA
    O. ochengi NPR-12   ------------------------------LHKFQNSFLWQHRGEN-YVLLLYGLLCIFGTLANLFVLAAFLRTSHIRNLRNYFIANLAVSDLLLCVVTA
        L. loa NPR-12   -----MPALIFAESGSRSNWSDINCDEFDEIYNIQNSFLWQHRGES-YVLLLYGLVCVFGTLANLFVLIAFLRTSHIRNLRNYFIANLAVSDLLLCVVTA
       A. suum NPR-12   ----------------------INCVWFDEIYYIQNNFYWRHRGAG-YMLLLYGLVCTFGALANLLVLVAFLRTPHLRNLRNYFIANLAVSDLLMCIVTA
N. brasiliensis NPR-12  -------------PNASGEWVVVECDWFDEIRSMQNNYFWVLHLKGSSIVFLYGLVCTFGALANLVVLFAFIRTSNLRNLRNSFIVNLACSDLLLCVVTA
   H. contortus NPR-12   -------------PNASGEWVVVECDWFDQIRAMQNNYFWVLHLKGSSIVFLYGIVCTFGALANLIVLFAFIRTSNLRNLRNSFIVNLACSDLLLCVVTA
    C. elegans NPR-12   ----------MQPPNSTGQWTEIHCDWFAEMQNYQN-SLYKINFDGSPIVPMYGLVCSFGALANFIVLLAFVRTANLRNLRNSFIVNLAFSDLILCVVTA
      S. ratti NPR-12   -----------------------------------NTMMWETRGIK-FLPYLYTVTCIIGFLANSIIIIVFLKTKKLRNLRNFYIINLAISDLTICTVSA
  B. xylophilus NPR-12  MNTMDLTPGPAAITVGSRNWSFFSCDALDEVMGIQNGFYWRNKGAG-TIIMLYAIVCSFGALANLLVIVSFLKTPHLHNLRNYFIVNLAVSDLIMCVLTA
    G. pallida NPR-12   ---------------------------------LQNHLFWRQNSPV-LLLSLYAIVCSFGTVINLFVLLSFAKVSQLRNLHNYFIANLAVSDLLLCTVTA
      M. hapla NPR-12   ----------LQFNEQQSNWTEMDCGWLDEIREIQNHFYWRHTNPA-LLLCLYAAVCFFGAIANLFVLLSFARVAQLRNLRNYFIANLAFSDLLMCTITA
   M. incognita NPR-12  -----------QFNEQHLNGTEMDCGWLDEIREIQNHFYWRHTNPA-LLLCLYAAVCFFGAIANFFVLLSFARVAQLRNLRNYFIANLAFSDLLMCTITA

             Consensus                        I C   DEI  IQN FYW HRG G YVLLLYGLVCSFGALANLFVLIAFLRTSHLRNLRNYFIANLAVSDLLLCVVTA

     B. malayi NPR-12   PVTLYLTRNLFWPFGSLACQTLASVQAVNMFVSCMTLVLIAMDRVLLTFCPIKWQVKLAAVAPVMCYCTVWLASIVIAAPYFFAVNAEDV-MLEPWNQPY
  W. bancrofti NPR-12   PVTLYLTRNLFWPFGSFACQTLASVQAVNMFVSCMTLVLIAMDRVLLTLCPIKWQ--LAAVAPIICYCTVWLTSIVIAAPYFFAVNAEDV-MLEPWNQPY
   O. volvulus NPR-12   PVTLYLTRNLFWPFGSFACQ-LASVQAVNMFVSCMTLVLIAMDRFVLTSCPFKWK--FAATAPVMCYCIVWLTSLVIAAPYFFAVTTEDF-TLEPWNQPY
    O. ochengi NPR-12   PVTLYLTRNLFWPFGSFACQ-LASVQAVNMFVSCMTLVLIAMDRFVLTSCPFKWK--FAATAPVMCYCIVWLTSLVIAAPYFFAVTTEDF-TLEPWNQPY
        L. loa NPR-12   PVTVYLTKNLFWPFGSFACQTLASVQAINMFVSCMTLVLIAMDRFLLTLCPVKWK--LAATAPIMCYFIVWLTSILISTPYFFAVTAENV-MLEPWNQPY
       A. suum NPR-12   PVTLYLTLNLFWPFGNFACQTLASVQAVNMFVSCLTLVLIAMDRVLLTLCPVKWR--LAATAPVICYCIVWLLSLFVAAPYFFAVSAEDVHMFEPWNQPH
N. brasiliensis NPR-12  PVTLYTSANAFWPFGDIACKVLASVQAVNTFVSSLTLALIAMDRVLLTKYPVKWR--LAATASIIFYCIVWVVSIVIALPYSLTVKSLKFDTVEPWNDVH
   H. contortus NPR-12   PVTLYTSANAFWPFGDIACKVLASVQAVNTFVSSLTLALIAMDRVLLTKYPVKWR--LAATASIICYCVVWVVSIVIALPYSLTVKSQKFDKVEPWNDVH
    C. elegans NPR-12   PVTLYTSLNLFWPFGDWSCKFLAGVQAVNTFVSSLTLAFIAMDRVLLTLCPVRWR--LAATAPLLCYGVVWIISIIVALPYALAVSS-KLAPFDPWSDRA
      S. ratti NPR-12   PFTIYQSMNFYFPFNESACKFVASFQAINMFVSCFTLVLIAIDRFFLTHSPISWY--VSITAPLLLYIVLWIFAFIITTPYISMIKLYTDEAFFPSSRAF
  B. xylophilus NPR-12  PFTLYLTLNLFWPFGNLACQSVASAQAVNLFVSCLTLVLIAMDRFLLTLCPVKWR--LAAKAPLAFYVVVWLASLIIALPYFFAVQAETVDYFDPWNQPS
    G. pallida NPR-12   PMTLYLSMNFFWPFGTPACQ-------------------------------------------I-------VCSVVVAAPYGAAVTAGDALMLDPWNTPH
      M. hapla NPR-12   PVTLYLSLNFFWPFGKFACQLVASIQAVNMFVSCLTLVLIAMDRFLLTLCPVKWR--LAAKAPLICYLFVWICSFIVAAPYGAAVSAEYARMLDPWNSAN
   M. incognita NPR-12  PVTLYLSLNFFWPFGKFACQLVASIQAVNMFVSCLTLVLIALDRFLLTLCPVKWR--LAAKAPLICYLFVWICSFIVAAPYGAAVSAEYARMLDPWNSAN

             Consensus  PVTLYLSLNLFWPFG FACQ LASVQAVNMFVSCLTLVLIAMDRFLLTLCPVKWR  LAATAPIICYCIVWL SIVIAAPYFFAVSAE   MLEPWNQPY

     B. malayi NPR-12   IDVTL-HICGRRRPQTCVE--KAWHRLPFSRRTYTLSMLAIQYLLPLAALAFSYSQIGSAIYKRVKASTTIGQKRRQVMMQRNRKALLLLSLLVLIYATA
  W. bancrofti NPR-12   IDVTL-HICGRKRPQTCVE--KAWHRLPFSRRTYTLSMLAIQYLLPLTALAFSYSQIGSAIYKRVKASTTIGQKRRQVMMQRNRKALLLLSLLVLIYAAA
   O. volvulus NPR-12   IDL---YICGRKRPQTCIE--KAWHRLPFSRRTYTLSVLAIQYLLPLTALALSYSQIGSAINKRIKTSTTVDQKRRQVMLQRNRKALLLLLLLVLIYAVT
    O. ochengi NPR-12   ID------CGRKRPQTCIE--KAWHRLPFSRRTYTLSVLAIQYLLPLTALALSYSQIGSAINKRIKTSTTVDQKRRQVMLQRNRKALLLLLLLVLIYAVA
        L. loa NPR-12   TDVML-HICRRKRPQTCIE--KAWHRLPFSRRTYTLSVLAIQYLLPLTALVFSYSQIGSAIRKRVKASTTIDQQRRQLMIQRNRKALLLLLLLVLVYAIA
```

```
      A. suum NPR-12     IGTMV-RYCGRHRPQICLE--KTWHRLPFSRRTYTLTVLAIQYLLPLAALAFGYSQIGSTIRRRVKASTTVDQQRRHIMMQRNRKALLLLLLLVLIYAIA
N. brasiliensis NPR-12   TPAML-KICDRSYPEICMEMQDTWERAYISKTTFTVTVLAIQYLLPLFALAYAYMQIGSTIRRRSKVSRTINQARRMSMQSRNRRALLLLMILVLTYAIC
   H. contortus NPR-12   TPKML-SICGRSYPEICMEIQDTWERAYISKTTFTITVLAIQYLLPLFALAYAYVQIGSTIRRRSKVSRTINQARRMSMQSRNRRALLLLMILVLTYAIC
     C. elegans NPR-12   TPKML-TYCNRQVPEICAEMQEAWDNAIVSKTTYTFVVLGIQYILPLAALAYAYFQIGSTIQKRSKVSRTVDTTRRMQMQNRNRRALLLLFLLVLTYAVC
       S. ratti NPR-12   TNLSLELCPNALPPNVCGE---DRWFYMYKQQIYTLTILAIQVLLPLIILIYSYTQIGFVIKHRASIPMTMDANRRHERQKKLTRSILLLTALVLVYALI
   B. xylophilus NPR-12  IDEMVGLFEPKLPPKMCLER--TWHRLPVSRRTYTLTVLAIQYVLPLVSLGFAYSQIGFTIRKRVKYNTTVDQHRKQILAQRNRKALLLLTLVLVYGIC
     G. pallida NPR-12   VDHLL-SICDRSRPQICMENPDTWNRLPFSRRNYTLFVLGIQYILPLSALAIAYSQIGSTIRKRVKMSTTVDGDRKQSMAKRNRKALLLLSCLVIVYAIA
       M. hapla NPR-12   VELLL-PICDREQPQICMEDVKRWDRLPFLEER---IIFK-RYLLPLSALAVAYSQIGSTIRRRGKASTTVHGERKQQLQRRNRKAMFLLLGLVFVYAIA
   M. incognita NPR-12   VELLL-PICDREQPQICMEDVKRWDRLPFSRRTYTLTVLAIQYLLPLSALAIAYSQIGSTIRRRGKASTTVHGERKQQIQRRNRKAMFLLLGLVLVYAIA

           Consensus     IDLML  IC R RPQICME  K WHRLPFSRRTYTLTVLAIQYLLPLTALAFAYSQIGSTIRKRVK STTVDQ RRQVM QRNRKALLLLLLLVLVYAIA

      B. malayi NPR-12   WFPMNAYNVLNVLEVIEFSQYRYLFCHLVGMTSACINPILYALINDSFRAAFISILRPVIRPCARYTTVSTNHQSTFTFSFNERELVNHNKAQISI----
   W. bancrofti NPR-12   WLPMNAYNVLSVLEVIEFSQYRYLFCHLVGMTSACINPILYALINDSFRAAFISVLRPVIRSCTRYTTVSTNQQSTFTFSFNEREPVKHQEAQISI----
    O. volvulus NPR-12   WFPMNAYNVLNVLEVIEFSQYRYIFCHLTGMTSACINPILYALINDSFRTAFISIL--------------------------------------------
     O. ochengi NPR-12   WFPMNAYNVLNVLEVIEFSQYRYIFCHLTGMTSACINPILYALINDSFRTAFISIL--------------------------------------------
         L. loa NPR-12   WFPMNAYNVLNVLEVIEFSQYRYLFCHLVGMTSACINPVLYALINDNFRAAFISVLRPVIRPCTTYSSVPINQQNTYTFSFNEHQLFKHQEVLAKVSCIK
        A. suum NPR-12   WFPMNAYNVLNVLDVIEFSQY--IFCHLVGMTSACMNPIMYGLLNDSFRSAFVSILRPFLRPCTKYTTVAVPPSHTYTFS--------------------
N. brasiliensis NPR-12   WAPMNIHNVLSGFEVISYSQYRYLFCHLVGISSACVNPITYALVNESFRNALHHMFLSFRPCYVTSGDSASS----YITNSKPAKATNREGYSTRFELPT
   H. contortus NPR-12   WAPMNIHNVLSGFEVISYSQY--LFCHLVGISSACVNPITYALVNESFRNALHHMFLSFRPCYVTSGDSASSYITGSKAAKVAV----------------
     C. elegans NPR-12   WAPMNIYHVLNGLEIINYSQNMYIFCHLVGISSTCVNPIVYALVNESFRNALQSMILQFRPCYVTTTGTAATNVYAYSATSKAENVTLMRDPFSTTPRPN
       S. ratti NPR-12   WFPMNLYHILTVLGLIKYSQY--ITLHFIGVTSVIINPLLYGIINDQFKNA-------------------------------------------------
  B. xylophilus NPR-12   WLPMNAYNVLNVFEVIEFSQY--IYCHLIGMTSASINPVMYGLINDSFRNAFFNMLRPFFGPC-------------------------------------
     G. pallida NPR-12   WAPMNAYNVLHVFEAINFSQYQYLFCHLIGMTSACLNPLLYALINDSFRSAFLQLI--------------------------------------------
       M. hapla NPR-12   WAPMNIYNVLHVLELIAFSQYNYLFCHLIGMVSACLNPLLYALINESFRAAFLQLI--------------------------------------------
   M. incognita NPR-12   WAPMNIYNVLHVLEFIAFSQYNYLFCHLIGMVSASINPLLYALIN-------------------------------------------------------

           Consensus     W PMNAYNVLNVLEVIEFSQY YLFCHLVGMTSACINPILYALINDSFR AFISIL

      B. malayi NPR-12   ------------------
   W. bancrofti NPR-12   ------------------
    O. volvulus NPR-12   ------------------
     O. ochengi NPR-12   ------------------
         L. loa NPR-12   QYESLGRHGQSLNQISYR
        A. suum NPR-12   ------------------
N. brasiliensis NPR-12   KNEEIETL----------
   H. contortus NPR-12   ------------------
     C. elegans NPR-12   ERPDSV------------
       S. ratti NPR-12   ------------------
  B. xylophilus NPR-12   ------------------
     G. pallida NPR-12   ------------------
       M. hapla NPR-12   ------------------
```

M. incognita NPR-12 ------------------

Consensus

11. NPR-13 homologues

```
       B. malayi NPR-13   ---------------------------------------------------------------------------LLCITALPITPVLAFVKRWLFGSLL
     W. bancrofti NPR-13   -----------------------------VFYSALYATIFVVGLIGNGLLIGSLVKRKRISVPNIFLINLAISDQLLCITALPITPVLAFVKRWLFGSLL
      D. immitis NPR-13   ---------------------KHYNIYFQLFYSALYATIFVIGLIGNGLLIGSLVKRKRISVPNIFLINLAISD-LLCITALPITPVLAFVKRWLFGSLL
      O. volvulus NPR-13   --------CVDVNDIMSAFGDWTLRLDVQLFYSALYATIFVAGLIGNGLLIGNLVKRKRISVPNIFLINLAISDLLLCITALPITPVLAFVKRWLFGALL
       O. ochengi NPR-13   ---------------------------FQLFYSALYATIFVAGLIGNGLLIGNLVKRKRISVPNIFLINLAISD-LLCITALPITPVLAFVKRWLFGALL
           L. loa NPR-13   ----------------------NYPSFFQLFYSALYATIFVVGLIGNGLLIGSLAKRKRISVPNIFLINLAISD-LVSVTALPITPVLAFVKRWLFGSLL
          A. suum NPR-13   ----------------------------QLIYSSLYAIIFLIGLIGNGLLISSMVRRKHISVPNIFLINLAVSDLLLCITALPITPVLAFVKQWLFGSLM
       A. caninum NPR-13   ----------------------------------------------------------------------------------------------------
 N. brasiliensis NPR-13   --------CVDVNYILQMYGDWTSLLEIRVTYSVVYAFIFVVGVVGNGLLISSVVLRKRSTVANVFLVNLAVSDLLLCITSVPITPVLAFMKRWMFGLLL
     H. contortus NRP-13   ---------------------------FQVVYSMVYAIIFIVGVVGNGLLIASVLLRKRSSVANVFLLNLAVSDLLLCITAVPITPVLAFMKRWMFGLLL
       C. elegans NPR-13   MGDAESHHCIDVNAILQQFNDWTVLFEVRLGYSVLYFLILIIGLVGNGLLITSILMRKKLSVANIFLINLAVSDLLLCITAVPITPVLAFMKRWIFGIIM
         S. ratti NPR-13   -------------------------IFLKIISYIIYAILFLLGLLCNGLLIGSMVIRKRKSVANIFLINLAIADILLCFTTLAITPLVSHKKVWLLGAFI
    B. xylophilus NPR-13   -------------------------------------------------------IRRQMTVANVFLINLAISDLLLCITALPITPVLAFMKRWIFGDIM
       G. pallida NPR-13   --------KQCVDLNTFMNSFGDWTLRTEVQYSSFYALIFLVGLIGNGLLIR--TIRRRMSVANVFLLNLAISDLLLCITALPITPVLAFVKQWIFGSVL
         M. hapla NPR-13   ---EIENCIELNSVLDQFGDWTLRWEVK-FFYSLFYAAIFIVGLIGNGFLVG--TIRRRMTVANVFLMNLAISDLLLCITALPITPVLAFVKRWIFGLAL
     M. incognita NPR-13   ---EIENCIELNSVLDQFGDWTLRLDVKVFFYSLFYAAIFIVGLIGNGFLVG--TIRRRMTVANVFLMNLAISDLLLCITALPITPVLAFVKRWIFGLAL

               Consensus                                L YS LYA IFVVGLIGNGLLIGSLVIRKRISVANIFLINLAISDLLLCITALPITPVLAFVKRWLFGSLL


       B. malayi NPR-13   CKLVPLCQGISVLISSYCLCFIAVDRYRSIVMPTREPWTTYHAYSLMAISWLTSATVSSPLFVTQRLQPLALENTTLCGY---FCGEYNWPAD-NRIKLF
     W. bancrofti NPR-13   CKLVPLCQGISVLISSYCLCFIAVDRYRSIVMPTREPWTTYHAYSLMAISWLTSATVSSPLFVTQRLQPLALENTTLCGY---FCGEYNWPAD-NRIKLF
      D. immitis NPR-13   CKLVPLCQGISVLISSYCLCFIAVDRYRSIVMPTREPWTTYHAYSLMAISWLTSATVSSPLFVTQRLQPLALENTTLCGY---FCGEYNWPED-NRVKLF
      O. volvulus NPR-13   CKLVPLCQKISVLISSYCLCFIAVDRYRSIVMPTREPWTTYHAYLLMAISWLTSATVSSPLFVTQQLQPLALENITLCGQ---FCGEYNWPED-NRIKLF
       O. ochengi NPR-13   CKLVPLCQKISVLISSYCLCFIAVDRYRSIVMPTREPWTTYHAYLLMAISWLTSATVSSPLFVTQQLQPLALENITLCG----FCGEYNWPED-NRIKLF
           L. loa NPR-13   CKLVPLCQGISVLISSYCLCFIAVDRYRSIVMPTREPWTTYHAYSLMAISWLISATVSLPLFVTQQLQPLALENTTLCGY---FCGEYNWPED-NRIKLF
          A. suum NPR-13   CKLVPLCQGISVLISSYCLCFIAVDRYRNIVTPMKEPWTVRHARILVAISWLASVTVSSPLFITQRLQPLVLENTTLCGH---FCGEYDWPGD-NRVKLL
       A. caninum NPR-13   -------------------------------------------QWILMCTWLVAMFASSPLYFSQSLKTLSMANITLCGEVCQFCGEFNWDQE-DHTKLV
 N. brasiliensis NPR-13   CKLVPSCQAISVLISSWCLCYIALDRYRSIVTPLKEPWKLKHAQRVLIATWLVAIFASSPLYFSQSLKTLTMANITLCG---EFCGEYNWAQE-DHTKLV
     H. contortus NRP-13   CKLVPPCQGFSVLISSWCLCYIAIDRYRSIVTPMKEPWKLKHAQ-VLIATWLVAIFTASPLYFSQSLKTLTMANITLCGE---FCGEFNWSQE-DHTKLV
       C. elegans NPR-13   CKLVPTCQAFSVLISSWSLCYIAIDRYRSIVTPLREPWSDRHARWLLMFTWVVAFLASYPLYYSQNLKTMVIENVTLCG---DFCGEFNWQSD-EISKLT
         S. ratti NPR-13   CKIISPCQSICVLVTSYSLCFIALDRYRSIVTPMKEPYSIFQAQIFMIFMWLSSIIISIPSFVTSGLKVISSDAATLCGE---FCGELYWPS--PQVQTG
    B. xylophilus NPR-13   CKLVPFCQGISVLISTYCLCLIAVDRYRSIVTPLKVPWTTRQAQCFMLVCWTAAVVISLPLFVAQQLKHIAFANITLCGQ---FCGEYNWPTD-TRVKLT
       G. pallida NPR-13   CKLVPLCQGISVLISSYCLCFIALDRFRSIVTPLKMPWGIGYAQALMILCWVCCIAIASPLFMAQRLQLIEFNNMTFCGL---FCGEYNWPEGHMQLKLA
         M. hapla NPR-13   CKLVPLCQGISVLISSYCLCLIAVDRYRSIVTPLKVPWNIQHAQWLMTLCWTFCIIISSPLFIVQGLQQIVYKNMTFCGE---FCTELNWPTD-FRIKLF
     M. incognita NPR-13   CKLVPLCQGISVLISSYCLCLIAVDRYRSIVTPLKVPWNIQHAQWLMTLCWTFCIIISSPLFIVQGLQQIVYKNMTFCGE---FCTELNWPTD-FRIKLF

               Consensus   CKLVPLCQGISVLISSYCLCFIAVDRYRSIVTPLKEPWT  HAQ LM ISWL SI VSSPLFVTQ LQ LALENITLCG    FCGEYNWP D  RIKLF7


       B. malayi NPR-13   YGSMLLICQYLIPVTIMTFCYWKILQKVRADWIVAQHS--MLTAAQQAQTAVRKRRVMYVLILMVVVFMASWLPLTIVNIFRDFGI---------EAFLD
```

```
W. bancrofti NPR-13      YGSMLLVCQYLIPVTIMTFCYWKILQKVRADWIVAQHS--MLTAAQQAQTAVRKRRVMYVLILMVVVFMASWL----------------T-----EAFLD
D. immitis NPR-13        YGSMLLVCQYLIPATIMTFCYWKILQKVRADWIVAQHS--MLTVAQQAQTAVRKRRVMYVLILMVVVFMASWLPLTIVNIFRDFG----I-----EAFLD
O. volvulus NPR-13       YGSMLLVCQYLIPAIIMTFCYWKILQKVQADWIVAQHS--MLTAAQQAQTAVRKRRVMYVLILMVVVFMASWLPLTIVNIFRDFGIFAHVIV--AEAFLD
O. ochengi NPR-13        YGSMLLVCQYLIPAIIMTFCYWKILQKVQADWIVAQHS--MLTAAQQAQTAVRKRRVMYVLILMVVVFMASWLPLTIVNIFRDFGIFAHVIV--AEAFLD
L. loa NPR-13            YGSMLLICQYLIPVTIMTFCYWNILQKVRADWIVAQHS--LLTAAQQAQTAVRKRRVMYVLILMVVVFMASWLPLTIVNIFRDFG---------IEAFLD
A. suum NPR-13           YGTMLLICQYLIPVSIMTFCYWKILQKVRTDWIVTEGS--MLTQAQQAQTSVRKRRVMYVLILMVVVFMASWFPLTIVNILRDFG---------IGTFLD
A. caninum NPR-13        Y-AFLLVVQFVIPAIIMSFCYWKILQKVRADWIVEEGS--MLTAAQQAQTAVRKKRVMYVLILMVVVFMTSWLPLSAVNLIKDLEIP------------
N. brasiliensis NPR-13   YGILLLVVQFMIPAIIMSFCYWKILQKVRADWIVEEGS--MLTAAQQAQTAVRKRRVMYVLILMVVVFMTSWFPLSAVNLIKDLEIPLV---------L
H. contortus NRP-13      YGISLLAVQFMIPAVIMSFCYWKILQKVRTDWIVEEGS--MLTAAQQAQTAVRKRRVMYVLILMVVVFMTSWLPLSAVNLIKDLEIP----L---VCCIL
C. elegans NPR-13        YTTSLLIIQLIIPAIIMSFCYLMILQKVQTDWLVDEGS--MLTAAQQAQTAVRKRRVMYVLILMVIVFMACWFPLSAVNLFRDLGMRFEFCQTVYKVLMM
S. ratti NPR-13          YGVILVFVQFVIPSVVMGFCYWHILQKVREDWIVNKGS--MLTQAQQAQTAIRKKRVMYVLIMMVFVFLGSWAPLTLCNMARDFG------------VQL
B. xylophilus NPR-13     YGSALLILTYLIPVSIMAFCYWKILQKVRQDWIVNTGS--MLTEAQQAHTSVRKRRVMYVLILMVAVFMGSWMPLTIVNILRDIG----------VSALE
G. pallida NPR-13        YGISLLVIQFLVPTAIMTYCYWKILQKVRQDWLVPANSSSTMTVEQQAATVLRKRRVMYVLILMVLIFMGSWMPLTIVNLLRDMD----------ILRDA
M. hapla NPR-13          YGISLLSIQFLIPTLIMTYCYWKILQKVRQDWLVPTNNS-IMSLEQQAQTAIRKR----RVMLKVLIFMGSWMPLTFVNLLRDIG----------ISFLE
M. incognita NPR-13      YGISLLSIQFLIPTLIMTYCYWKILQKVRQDWLVSTNNS-IMSLEQQAQTAIRKRRVMYVLILMVLIFMGSWMPLTFVNLLRDIG----------ISFLE

Consensus                YGSMLLVIQYLIPAIIMTFCYWKILQKVRADWIV   S  MLTAAQQAQTAVRKRRVMYVLILMVVVFMASWLPLTIVNILRDLG           AFLD

B. malayi NPR-13         IQMYFKLLNVHAIAMTSIVWNPLLYFWMSKRHRQAFKEDMIGAGNNYRR--NNDVRLTKRFVVTK--TPQPGNLLYNQN---------------------
W. bancrofti NPR-13      IQMYFKLLNVHAIAMTSIVWNPLLYFWMSK----------------------------------------------------------NFRRGTLTDPTC
D. immitis NPR-13        IQMYFKLLNVHAIAMTSIVWNPLLYFWMSKRHRKAFKEDMIGTGNNYRR--NNELRLVKRVCMSR--SPRPGNLLYDQNDT-------NYRRSTLTDPTC
O. volvulus NPR-13       IQMYFKLLNVHAIAMTSIVWNPLLYFWMSKRHRKTFKEDMIGAGN-YRR--NNELKLMKRLCEPK--SPQPGNHLYDQSDRH------NFRRGTLTDPTC
O. ochengi NPR-13        IQMYFKLLNVHAIAMTSIVWNPLLYFWMSKRHRKTFKEDMIGAGN-YRR--NNELKLMKRLCEPR--SPQPGNHLYDQSDRH------NFRRGTLTDPTC
L. loa NPR-13            MQMYFKLLNVHAIAMTSIVWNPLLYFWMSKRHRKAFKEDMIGTGNNYRR--NNEMRLMKRFFISK--SPQPNNLVYKQNHR-------NFRRGTLTDPTC
A. suum NPR-13           VQMYFKLLNVHALAMTSIVWNPLLYFWMSKRHRRALKYDMFSLTN-PKR--HNQLGVLGRFTPSPSFNVIYRRTVERHIMRN------NFRRGTLADPTC
A. caninum NPR-13        ----------------------------LQRHRRALKDDMTWLTNVRR---HTNVGVLSRFAPSPSVSLVYRRTLERHLSVG------NFRRGTLADPTC
N. brasiliensis NPR-13   EQMYFKLMNAHAIAMTSVVWNPLLYFWMSKRHRRALKDDMTWLTNARR---HTNVGVLSRFTPSPSVSLVYRRTLERHLGVN------NFRRGTLADPTC
H. contortus NRP-13      EQMYFKLLNVHAIAMTSVVWNPLLYFWMSKRHRRALKDDMTWLTNVRR---HTNVGVLSRFAPSPSVSLIYRRTLERHLGVT------NFRRGTLADPTC
C. elegans NPR-13        DQMYFKLLNVHVIAMTSIVWNPVLYFWMSKRHRRALKDDMTWLTNARR---HTNVGVLSRFTPSPSVSVVYRRTLERHLGVN------HFRRGTLADPTC
S. ratti NPR-13          TERYFITLCANIIAMTSIITNPMLYFWLSKRHRRALKDDMFWLTNVRRN--QQNIGILDQFEPS---SLLYKKSYDPLTISSF---HQNNRRGTLADPTC
B. xylophilus NPR-13     VQMYFKLLNAHAVAMSSIVSNPLLYFWMSKRHRRALKDDMFWLTNVRR---QHQMGLLEKFAPSPSVGIMYRKSLERHLN--------LFRRGTLADPTM
G. pallida NPR-13        NNMYFKLLNVHAVAMTSIVSNPLLYFWMSKRHRRALRDDINWFTNARRQQQQNVGGLLNKFTPSPSIGLLYRKNVERHLLQNAAAKYNQQRRGTLGDPTC
M. hapla NPR-13          TQMYFKLLNVHAVAMTSVVSNPLLYFYMSKRHRRALRDDMYWLTNARRQQNQHVGGLLAKFTPSPSIGLLYKKSLERHILQNATAKYNPYRRGTLADPTT
M. incognita NPR-13      TQMYFKLLNVHAVAMTSVVSNPLLYFYMSKRHRRALRDDMYWLTNARRQQNQHVGGLLAKFTPSPSIGLLYKKSLERHILQNATAKYNPYRRGTLADPTT

Consensus                IQMYFKLLNVHAIAMTSIVWNPLLYFWMSKRHRRALKDDM WLTN RR   NN VGLL RF PSPSVSLLYRKTLERHL         NFRRGTLADPTC

B. malayi NPR-13         -----------------------------------------------------------------
W. bancrofti NPR-13      ITAER-AFAHIHANCFLLVPLLPLYQA--------------------------------------
D. immitis NPR-13        ITAET-AFAHMHASCFLLVPLLPLYQA--------------------------------------
O. volvulus NPR-13       ITAET-AFAHMHANCFLLVPLLPLYQS--------------------------------------
O. ochengi NPR-13        ITAET-AFAHMHANCFLLVPLLPLYQS--------------------------------------
```

```
          L. loa NPR-13     ITAER-AFAHMHANCFLLVPLLPLYQA--------------------------------------
         A. suum NPR-13     MQSEK-ALADMHANCFLLVPLMPLCRSVNVSDSRIGLRSNDRQIVIHPSQ---------------
      A. caninum NPR-13     MSRER-AQADLHSDCFLLVPLMPLCQSV-------------------------------------
N. brasiliensis NPR-13     MSRER-AQADLHSNCFLLVPLMPLCQSLNRRNSHLTIEDHRSGHV--------------------
    H. contortus NRP-13     TMRER-AQADLHSNCFLLVPLMPLCQSVSRRDSRLTID---------------------------
      C. elegans NPR-13     TSRERSLPRELQSNCFLLVPLMPLCQSVTRKNSHLAINRDGIVIPQANGSSRRPSSVNTNSTRDW
        S. ratti NPR-13     ATRDD-QLEDVKAACFLLVPIMPFCKE--------------------------------------
   B. xylophilus NPR-13     MSRER-SLQEMHANCFLLVPLMPLC----------------------------------------
      G. pallida NPR-13     ISRER-ALQEMHANCFLLVPLMPLCVANATTLQRHH-----------------------------
        M. hapla NPR-13     LGREK-ALQEMHANCFLLVPLMPLCVANQQHLTTNRREI--------------------------
    M. incognita NPR-13     LGREK-VLQEMHANCFLLVPLMPLCVANQQRLATNQREISNNNNINLNFKRQKHPKFVCEA----

           Consensus     ITRER A ADMHANCFLLVPLMPLCQS
```

12. NPR-22 homologues

```
     T. spiralis NPR-22   --------------------------------------------LTADESAVAVVVYTLITVVAVIGNATVIFIIGHFPRLRTPTNALIANLALADLLMA
        T. muris NPR-22   ------------------------------------------------------------------GNVVVIFIVVRFPRMRTPTNALLANLALADTLVA
         A. suum NPR-22   ---------------------------------------------------LAIFLYSGLSIVAICGNSLIVLVILYFRRLRTATNILILNLAIADLMIS
      A. caninum NPR-22   ----------------------------------------------------------------------------------------------------
 N. brasiliensis NPR-22   ----------------------------------------------------------------------------------------------ADLLIA
    H. contortus NPR-22   -----------------------------------------PSQKNFYFQVLCICFYNILCVCSVYGNFLVILVILYFRRLRTATNILIFNLAIADLLIA
      C. elegans NPR-22   MDEGGGIGSSLLSRITTTASEIMMRNEPTTTENPAVQEMNHIYHLTPSMKMLCILFYSILCVCCVYGNVLVILVIVYFKRLRTATNILILNLAVADLLIS
        S. ratti NPR-22   -----------------------------------------------------VILYTLLSCIAFIGNLMIIVIILYFRKLKTGTNMLIMNLAIADIMIA
    B. xylophilus NPR-22  --------------------------------MNDSAEVLELQYLCPTCKVTAVVLYTTLSIMAFIGNSLIVAVIIYFRRLHTATNMLIMNLAIADLMIS
      G. pallida NPR-22   ------------------MIEQTLEYSNQPTQSGSENVPIAVQQLCLTCKLIAILSYSTLSLIAFVGNALILAVFFRFKRLRTPTNMLIANLAIGDLMVS
        M. hapla NPR-22   ----------------LKWIFNSKEFN--KLKNFKINLINLFILIKLKNKYIFILFISIYMLHIIVGNALILAVFLRFKRLRTPTNMLIANLALGDLMIS
     M. incognita NPR-22  -----------------------------------------------------------------VGNALILAVFLRFKRLRTPTNMLIANLALGDLMIS

               Consensus                                                     L IL YS L V A IGNALII VILYFKRLRT TNMLI NLAIADLMIS

     T. spiralis NPR-22   LLCIPFSFVSTLILQY--WPFGWFLCKVINFSQAVTVMASAYTLIAISLDRFFAILLPMSPNVKMTNRKATLCVLVVWTVAIVIAFPLLMVLTLEPS-ET
        T. muris NPR-22   LLCIPFSLASTLLLEY--WPFGWFLCKVIHFSQAVTVMASAYTLIAISVDRFCGILFPMSTRAKLTNRKAALVIAIVWTVAIAIAFPLLMVLSLAASPDD
         A. suum NPR-22   VLCMPFSYWHVIIFSDQRWIFGAIFCKLFAFLQATAVFLSSWTLVAISFDRFTAIMFLMSPWMRLTRRRALYLVAITWIFSLIMAAPLFYVNQIQR---R
      A. caninum NPR-22   -------------------VFGSAMCSLLSFPFG-AVFLSSWTLVVISFDRWMAIMFVLSPSMRLTRKRAVYLVLATWAFSITMALPLFLVAQITT---T
 N. brasiliensis NPR-22   VFCIPFSYWQVLIFDDQRWVFGSAMCSLLSFFK-------------------------------------------------------------------
    H. contortus NPR-22   VFCIPFSYWQVLIFDDQRWVFGSAMCSLLSFFQGIAVFLSSWTLVVISFDRWMAIMFVLSPSMRLTRRRAVYLVLATWAFSITMALPLFLVSKIHT---T
      C. elegans NPR-22   VFCIPFSYWQVLIYDDQRWLFGSMMCSLLAFLQAMAVFLSAWTLVVISFDRWMAIMFLLTPNIRITRRRALYLVAATWIFSILMALPLLFTTRFFED--Q
        S. ratti NPR-22   LICMPFSYWHVIIFEDSKWIFGSLFCKLLNFFQATAVFSSSWTLVAIRFDRCLAILFVMSRWNRLNKKRALYVIFIIWFFSLLMACPLYFANEVWSHEGN
    B. xylophilus NPR-22  IFCMPLSYWHVIIFEDQRWIFGAFLCKIFNFLQASAVFLSSWTLVVISFDRFMAIMFVMSPWLRLTRKRAFFLILGTWVFSLAMALPLLIVNQTEAS--A
      G. pallida NPR-22   VFCIPLSYWHVLVFEDQRWVFGAFLCKCFNYLQATAVFLSSWTLVAISFDRFMAIQFAMSPWLKMTRRRALYAILGTWLFSLGMALPLLVVNKLSHS--S
        M. hapla NPR-22   IFCIPLSYWHVLVFEDQRWIFGAFLCKTFNYLQATAVFVSSWTLVAISFDRFMAIQFSMSPWLKMNRKRALYATLITWGFSLIMALPLLVVNQLESS--T
     M. incognita NPR-22  IFCIPLSYWHVLVFEDQRWIFGAFLCKTFNYLQATAVFVSSWTLVAISFDRFMAIQFSMSPWLKMNRKRALYATLITWGFSLIMALPLLVVNQLESS--S

               Consensus  VFCIPFSYWHVLIFEDQRWIFGAFLCKLLNFLQATAVFLSSWTLVAISFDRFMAIMFVMSPWLRLTRRRALYLVLITW FSLIMALPLLVVN L  S  T

     T. spiralis NPR-22   GDVTHCIENWTWT----ANETTGEYEYSLAAMLLQYFVPLLVLTLTYAGIGMKIWFTHVPGEGGYAGQCN---------RERKTLVKKVIPTMFIVTAVY
        T. muris NPR-22   DTILYCAENWTW-----ANETTGEYEYSLAAMCLQYFVPLLVLTLTYAGIGLKLWFS----EGGHAGRFS---------RQRKSMVKKVLPTMFIVTTVY
         A. suum NPR-22   DGVDTCEEYGWQYLES-KFGDGITHTYGTVVFSLQYCLPLTVLIISYASIGVKMWNS--RVPGVQP-------STRCVVNERHESVKKMIRMVLLVSALY
      A. caninum NPR-22   GGIKRCDEDWTAVEAF---IPGSRRLYSYLVLVLQYVVPLTVLIITYTFIGMKMWNS--RIPGPSS-------STKKVVMERHESVKKLIPMVILISAMF
 N. brasiliensis NPR-22   ----------------------------------------------------------------------------------------------------
    H. contortus NPR-22   DGIKRCDEDWS----------QSRRLYSYLILVLQYVVPLTVLIITYTFIGMKMWNS--RIPGPTS-------STKKVVMERQESVKKLIPMVILISAMF
      C. elegans NPR-22   DGLPNCGENWTYFGDS---GEQVRKVYSSMVLILQYVVPQAVLIITYTHIGIKMWNS--RVPGMQNG------ATKKMIVDRHESVKKLVPMVILISALF
        S. ratti NPR-22   DNTDTCVEKWNLLKVFGSDETKIVSLYNKSLFVLQYCMPLIVLIVTYTIIGFKMWFS--EIPGADSCSQASKRTNKTLLQERHESVKKLIPMVILISALY
    B. xylophilus NPR-22  LDVETCQEKWELLSPL-GDVDQIVHSYTSALFALQYCLPLFVLVITYTFIGLRMWNS--KVPGADRT-----QSTRYVVQGRHDSVKKLIPMVLLVSALY
```

```
       G. pallida NPR-22   DGVETCLEQWEHFFS-----ANFVSMYTSSLFGLQYCLPLAVLLITYTAIGCKMWNSG-KVPGGES------YLPPRASNKLKESVKKLIPMVLLVSALY
         M. hapla NPR-22   EGVDTCLEKWNDFFS-----DSFVSLYTSSLFALQYCLPLSVLLITYTAIGCKMWNS--KVPGR------------------KESVKKLIPMVLLVSALY
     M. incognita NPR-22   EGVDTCLEKWNDFFS-----DSFVSLYTSSLFALQYCLPLSVLLITYTAIGCKMWNS--KVPG------------------------KLIPMVLLVSALY

           Consensus       DGVDTC E W               LYSSSLF LQYCLPL VLIITYT IGMKMWNS  KVPG  S         K VV ER ESVKKLIPMVLLVSALY


      T. spiralis NPR-22   TFCWLPIN-VFNVVRALHPSVTDSTYVLHIWWACHTIAMVHCLVNPIIYICRNRRFREGFAF--------------------------------------
         T. muris NPR-22   SFCWLPLN-VFNILRALYPLINEYTYVLHVWWACHTVAMVHCMVNPIIYVSRNRRFREGFAY--------------------------------------
          A. suum NPR-22   ACCWLPQNLLINILLPLNPNIVSHPYILYFWWMANTIAMLHSIVNPFVYYTRNARFQEGICYFL-RFLPCVK----------------------------
       A. caninum NPR-22   AFCWLPLLLLINVIVDLWPSVASWRYILYIWWFSHGLAMMHSMVNPIVYFLRNARFREGFCYFS-RFLPCVHFQEFKLLSEQAKSGRSR-----------
  N. brasiliensis NPR-22   ----------------------------------------------------------------------------------------------------
      H. contortus NPR-22   AFCWLPLLLLINVVIDLWPSVASWKFILYMWWFSHGLAMMHSMVNPIVYFLRNARFREGFCYFS-RFLPFVHFSGFKLLTDQN-----------------
       C. elegans NPR-22   ALCWLPLLILINVIPEFYPDINSWGYILYLWWFAHGLAMSHSMVNPIIYFIRNARFREGFCFFSSKLLPCISFKELRLLTDNTSRSFRNRSRFSGVINPT
         S. ratti NPR-22   AGCWLPQNLLMNVLVN-DTDILTHPYILFIWWIAHTIAMFHSVVNPFIYYCQNKRMREGLRYLL-RWLPCVTFTEFKLLA--------------------
    B. xylophilus NPR-22   AGCWLPQNLLMNIWVTYDPTITSHPYILYIWWAAHTIAMFHSIVNPFIYYWQNRRINEGVRYLL-RFLPFIHFDNFHYLEEKPGCRAVKFVNMVAYMPVA
       G. pallida NPR-22   AVCWLPQNLLMNIFVTYDPAILSHPYILYIWWGSHSLAMCHSAVNPFVYYVTNRRIHAALKHLL-RWMPCLRNARNMALAEFDARKR---LASSFRYVSA
         M. hapla NPR-22   AVCWLPQNLLMNIWVTYDSSALNYPYILYIWWGAHTLAMFHSIVNPFVYYTTNRRIHAALKHLL-RWLPCLQNSRNVALAEFDQTARQKAVTNSFKYPIG
     M. incognita NPR-22   AVCWLPQNLLMNIWVTYDSSALNYPYILYIW-GAHTLAMFHSIVNPFVYYATNRRIHAALKHLL-RWLPCLQNARNVALAEFDQTARQKAVTNSFKYPVG

           Consensus       A CWLPQNLLMNILV   PSI SHPYILYIWWAAHTLAM HSIVNPFVYY RNRRFREGL Y L RFLPCV       L E


      T. spiralis NPR-22   -----------------------------------------------
         T. muris NPR-22   -----------------------------------------------
          A. suum NPR-22   -----------------------------------------------
       A. caninum NPR-22   -----------------------------------------------
  N. brasiliensis NPR-22   -----------------------------------------------
      H. contortus NPR-22   -----------------------------------------------
       C. elegans NPR-22   SSDEKPATSLTRYSRSGVLDRQTCRSARFFEARPLVVVRNNSANSLA
         S. ratti NPR-22   -----------------------------------------------
    B. xylophilus NPR-22   GKKVLHSTVSDSG----------------------------------
       G. pallida NPR-22   DQHHRRTTLQKTGSGSWHDATVPQ-----------------------
         M. hapla NPR-22   DNRRLTLHTQKTISLTMD-----------------------------
     M. incognita NPR-22   DSRRLTLHTQKTISLSMD-----------------------------

           Consensus
```

13. DMSR-1 homologues

```
     B. malayi DMSR-1      MNNLERCQTV-IIELPENSIVYDWFYAIHTFYSPIHTYLSIALCILGAMCNFCNIVVLTR-KRMRTPVNMTLTAMACCDTVVLFSNLIYTTHYTLAAFAN
  W. bancrofti DMSR-1      MNNLERCQTV-IIELPESSIVYDWLYAVHNFYSPIHTYLSIVLCILGAICNFCNIVVLTR-KRMRTPVNMTLTAMACCDTVVLFSNLIYTTHYTLAAFAN
   D. immitis DMSR-1       MNDLERCQTV-IIELPERSIVYDWFYAIHNFYNPIHTYLSIVICILGTMCNFCNIVVLTR-KRMRTPVNMILTAMACCDTVVLFSNLIYTTHYTFAAFVN
   O. volvulus DMSR-1      ------CQTV-IIELPERSIVYDWFYAIHTFYRPIHAYLSIIICILGAMCNFCNIVVLTR-KRMRTPVNMILTAMACCDTVVLFSNLVYTTHYTFT---N
    O. ochengi DMSR-1      MSNLKRCQTV-IIELPERSIVYDWFYAIHTFYRPIHAYLSIIICILGAMCNFCNIVVLTRRKRMRTPVNMILTAMACCDTVVLFSNLVYTTHYTFT---N
       L. loa DMSR-1      MNTLERCQTV-IIELPENSIVYDWFYAIHTFYSPIHTYLSIVLCILGAMCNFCNIVVLTR-KQMRTPVNMTLTAMACCDTVVLFSNLIYTTHHTFATFAN
      A. suum DMSR-1      MNALDQCQTV-IVQLPAESIVYEWLYGIHTFYRPIHTYLSIFMCAVGTICNFCNIVVLTR-KQMRTPVNMILTAMACCDTVVLFSNLIYTTHYTFVAFAN
    A. caninum DMSR-1     ------------------------------------------------------------R-RQMRTPVNMILTAMACCDTVVLFSNLIYTTHYSFVAFKH
N. brasiliensis DMSR-1    -MDTDQCQTV-LVHLPNHSVVYDFFREVHLFYKPIHTYLSIIMCIFGTVANFCNIVVLTR-RQMRTPVNMILTAMACCDTVVLFSNLIYTTHYSFVAFKH
   H. contortus DMSR-1    -MDADQCQTV-LVHLPNHSVVYDFFREVHLFYKPIHTYLSIIMCIFGTVANFCNIVVLTR-RQMRTPVNMILTAMACCDTVVLFSNLIYTTHYSFVAFKH
    C. elegans DMSR-1     -MEFTECKTT-FIHLPDKSFLYDVFVSVYNFYHPIHAYLSIFLCVLGTIANFCNIVVLTR-RTMRTPVNMILTAMASCDTVVLFSNLIYTTHYSFVAFKF
      S. ratti DMSR-1     ------CSTV-IIELPTESIVYDILKTVSDYYKPIHAYLSIFICVIGAACNFCNIVVLTR-RSMRTPVNMVLTAMAFCDTVVLFSNLIFTYHYTFDAYDS
  B. xylophilus DMSR-1    ------CQTV-VFQLPNESVIYDRLADLHAFYKPIHTYLSIVLCALGAVCNFCNIVVLTR-RQMRTPVNMILTAMACCDTVVLFSNLIYTTHYTFVAFAN
    G. pallida DMSR-1     MDPLHQCSTD-VFQLPDQSPFYELLKTIKDFYQPIHTYLSIVLCAVGTLCNVCNIVVLTR-KKMRSPVNLILCAMACCDTVVLSSNMVYTTHYTFVAFNN
      M. hapla DMSR-1     MDLLQQCQTQSFFQLPTESWLYIWLLTIKDFYKPIHTYLSIVLCALGAICNFCNIVVLTR-KKMRTPVNMILTAMACCDTVVLFSNLTYTTHYTFVAFEH
   M. incognita DMSR-1    MDLLQQCQTQSFFQLPTESWVYVWLLAIKDFYKPIHTYLSIVLCALGAICNFCNIVVLTR-RKMRTPVNMILTAMACCDTVVLFSNLTYTTHYTFVAFEH

             Consensus     M  L  CQTV II LPE SIVYDWF AIH FYKPIHTYLSIVLCILGAICNFCNIVVLTR KRMRTPVNMILTAMACCDTVVLFSNLIYTTHYTFVAF N

     B. malayi DMSR-1      CHPKQWSYGWAVFLVCHAHLSLIGHSSSIWLSVMLALIRYMTLRSRRKVNVLQIHLRHSYMAIAFVVFFVTLMNTPNFLAYKIIEMKLSETCNITDTGNY
  W. bancrofti DMSR-1      CHPKQWSYGWAVFLVCHAHLSLIGHSSSIWLSVMLALIRYMTLRSRRKVDVLRIHLRHSYMAIAFVVLFVTLMNTPNFLAYKIIEMKLGETCNITDTSNY
   D. immitis DMSR-1       CHPEQWSYGWAVFLVCHAHLSLIGHSSSIWLSVMLALIRYMTLRSRRKVNVLQIHLRHSYIAIAFVVLFVTSMNTPNFLAYKIFEMRLSETCNTTDVGNL
   O. volvulus DMSR-1      CHPEQWSYGWAIFLVCHAHLSLIGHSSSIWLSVMLALIRYMTLRSRKKVNVLQIHLRHSYMAIAIVVLFVTLMNTPNFLAYKIFEMRLSETCNTTDIGNL
    O. ochengi DMSR-1      CHPEQWSYGWAIFLVCHAHLSLIGHSSSIWLSVMLALIRYMTLRSRKKVNVLQIHLRHSYMAIAIVVLFVTLMNTPNFLAYKIFEMRLSETCNTTDIGNL
       L. loa DMSR-1      CHPKQWSYGWAIFLVCHAHLSLIGHSSSIWLSVMLALIRYMTLRSRRKVNVMQIHLRHSYMAIAFVVLFVTLMNTPNFLAYKIIEMRLSETCNITDIGNR
      A. suum DMSR-1      CHPRHWSYGWAMFLIAHAHLSLVGHSSSVWLSVMLALIRYMTLRSRGNMNGIQIGLRHSYMAIGFVVLFVAVMNAPNFLTYKIIEMRLNETCEIKDESVR
    A. caninum DMSR-1     CHPRHWSYGWALFLIF------Q-----------------------------IGLKHSYFAIGATICFVAIVNAPNFLTYKIHEL--NDTCIIRDPALL
N. brasiliensis DMSR-1    CHPRHWSYGWALFLIAHAHLSLVGHSSSVWLSVMLALLRYMTLRGRGNAGGSQIGLKHSYLAIGATICFVAIVNAPNFLTYKIHEIRLNDTCVVRDPALL
   H. contortus DMSR-1    CHPRHWSYGWALFLIAHAHLSLVGHSSSVWLS-MLALLRYMTLRSRGNSAGTQVGLKHSYLAIGGTICFVAVVNAPNFLTYKIHEFRLNDTCVVRDPALL
    C. elegans DMSR-1     CHPKHWSYSWALFLIAHAHLSLVAHSSSVWLSVMLALVRYVTLRSRGNMGGMQVTLRHSYYAVAVTVSLVAVLNAPNFLNYKINEQPLNETCTDLDPMFW
      S. ratti DMSR-1     CHPRDWSYGWAIFLVSHAHLSLVGHSSSVWLSVMLALIRYITLRSRGKLSGTQVTLKHSYLAIGAVIIFVTTMNIPNFLTYKIIEQPLSASCTIKDERFQ
  B. xylophilus DMSR-1    CHPRHWSYGWALFLVSHAHLSLVGHTSSVWLSVMLALIRYLTLRNRGRMSGMQVGLKHSYISIAAVIFFVLIMNSPNFLTYKIIEMPLKYTCTITDPSVM
    G. pallida DMSR-1     CVPKNWSYGWCVFLISHAHLSLVGHSSSIWLSVMLALIRYITLRRRGKSGGVQIGLQHSYIAIASVVVFVSLMNGPNFLTYKISEQTLDMTCGDLVAEQF
      M. hapla DMSR-1     CIPKHWSYGWCVFLISHAHLSLVFHSSSIWLSVMLALIRYLTLRRRGKACVSQVFLKHSYSAIACVILFVSIMNAPNFLTYKITEQTLGETCGDLAVLQY
   M. incognita DMSR-1    CVPKHWSYGWCVFLISHAHLSLVFHSSSIWLSVMLALIRYLTLRRRG-------------------------NAPNFLTYKITEQTLGETCGDLAVLKY

             Consensus     CHPKHWSYGWAVFLISHAHLSLVGHSSSIWLSVMLALIRYMTLRSRGKV  LQI LRHSYMAIA VVLFVTLMNAPNFLTYKI EMRL ETC I D GN

     B. malayi DMSR-1      D-ASAYVPGVSDLALQAHCLIFR-MAFWISGTVFKMTPCLLLS-SFVWLLMKILTRVQQNR--LKLLYHSTRFYNG-KHESKKKHRGQS-----------
```

```
    W. bancrofti DMSR-1   D-ASAYVPGVSDLALEAHCLIFR-MAFWISGTVFKMTPCLLLS-SFVWLLMKILTRVQQNR--LKLLYHSTRFYNG-KYESKKKNRGQS----------
      D. immitis DMSR-1   D-ASAYIPGVSDLALEAHCLIFR-MAFWISGTIFKMIPCLLLS-SFLWLLMKILTKVQQNR--LKLFYHSTHFCNG-KYHDKKDYQGRNKNAID------
     O. volvulus DMSR-1   D-AFAYIPGVSDLALEAHCLVFFRMAFWISGTLFKMIPCLLLS-SFVWLLMKILTKVQQNR--LKLLYNSSRYCDGKKYDDRKAYQGRNK----------
      O. ochengi DMSR-1   D-AFAYIPGVSDLALEAHCLVFR-MAFWISGTIFKMIPCLLLS-SFVWLLMKILTKVQQNR--LKLLYNSSRYCDGKKYDDRKAYQGRNKNAT-------
          L. loa DMSR-1   D-AFAYIPGVSDLALEAHCLIFR-MAFWISGTIFKMIPCLLLS-SLVWLLMKILTRVQQNR--VKLLHHSTYYYNQ-KYVGKKKYCGKEKIPLIRIYQQL
         A. suum DMSR-1   Y-APAYIPGVSDMAVQAYCLVFR-MAFWISGTVFKVVPCLLLT-LFVWLLMRILNEVKQNR--LRLMEKSRPSNDGSSPKMQADD--------------
      A. caninum DMSR-1   G-AAAYLPGIAEIALAKHCLVFR-LVFWISGLVFKVIPCCLL--------------------------------------------------------
 N. brasiliensis DMSR-1   G-AAAYLPGIAEIALAQHCLVFR-LVFWISGLVFKVIPCCLLS-LFVWLLLGILKEVKANR--QRLLKNSRNSSVPATAPARNGK--VTLVASN------
     H. contortus DMSR-1   G-AAAYLPGIAEIALAKHCL-FR-LVFWISGLVFKVIPCCLLS-LFVWLLLGILKEVKVKANRERLLKNSHHAMPPANS---NGK--VVLVAH-------
       C. elegans DMSR-1   N-SPAYLPGIADIAKANSCLVFR-LSYWISGMVFKVLPCALLS-LFVWLLLRILREVRENR--QRLLKNSQHR-PPNQTTTRNGQRLSISVAGN------
         S. ratti DMSR-1   N-ASAYLTGISELAVSNSCMVFR-ITFWMSGIIFKVIPCLILT-LLVWLLTRILNEVQKNR--LKLFKGSSKYSPPSSESLTKHDNNLFISSSP------
    B. xylophilus DMSR-1   A-ALAYFPEVSDLALETNCLVFR-MAFWISGTIFKVLPCMLLT-LFVWLLTRILNEVKENR--ARLMK-----GNG-----------------------
       G. pallida DMSR-1   KNDTAYVPGVAD--VAADCLVFR-LAFWITGAVFQLFPCLLLTLLLVSLLMRILREVRDNR--QRLFLAGRGAAAASGGG-------------------
         M. hapla DMSR-1   RNHTAYVPGVAD--IASDCLVFR-LAFWITGAVFKLFPCLLLT-VLVSLLTQILKEVRTNR--QRLFFGGGCSSSTFEANV------------------
     M. incognita DMSR-1   KNHTAYIPGVAD--IASDCLVFR-LAFWITGAVFKLFPCLLLT-VLVSLLTQILKEVRANR--QRLFFGGGCSSSTFEAN-------------------

            Consensus       A AYIPGVSDLALAAHCLVFR MAFWISGTVFKVIPCLLLS LFVWLLMKIL EVK NR  LRLL  S     G     K

        B. malayi DMSR-1   ----KTDTAKTNTSIVYRADRTTRMLLTILCVFLITELPQGIMMVLSGILPEAFRRHIYNSLGDLLDLLSLCNSCTTFVIYCSMSEQFRNEFKQVFLPNS
     W. bancrofti DMSR-1   ----KIDTAKTNTSIVYRADRTTRMLLTILCVFLITELPQGIMMVLSGILPEAFRRHIYNSLGDLLDLLSLCNSCTTFVIYCSMSEQFRNEFKQVFLPHS
       D. immitis DMSR-1   -----SCYSKTNTDTIYRADRTTRMLLTILCVFLITELPQGIMMVLSGILPEAFRRHIYNSLGDLLDLLSLCNACTTFVIYCSMSEQFRNEFKQVFLPNS
      O. volvulus DMSR-1   ---------RTNASTVYRADRTTRMLLTILCVFLITELPQGIMMLLSGILPEAFRRHIYNSLGDLLDLLSLCNACTTFIIYCSMSEQFRNEFKQVFLPYS
       O. ochengi DMSR-1   ---------RTNASTVYRADRTTRMLLTILCVFLITELPQGIMMLLSGILPEAFRRHIYNSLGDLLDLLSLCNACTTFIIYCSMSEQFRN--KQVFLPYS
           L. loa DMSR-1   IVVPRMINFRINTSVVYRTDRTTRMLLTILCVFLITELPQGIMMVLSGILPEAFRRHIYNSLGDLLDLLSLCNACTTFVIYCSMSEQFRNEFKQVFLPNS
          A. suum DMSR-1   --NHSFKQASHVRC-GERTDRTTHMLLAIVFVFLVTELPQGVMAVLSGMFSEEFRRHIYNNLGDILDLLSLCGACTTFIIYCSMSGQFRNEFRRVFLP--
       A. caninum DMSR-1   ----------S--------DRTTYMLIIMLVVFLVTELPQGFLALLNG----------------------------------------------------
  N. brasiliensis DMSR-1   DQHKRTSSIRLAPPRGERADRTTNMLLAIVFVMLFTEMPQGIMALLSGMISQEFRRHIYNNLGDLLDLFSLCEACMSFIIYCSMSGQFRNEFHRVFVP--
      H. contortus DMSR-1   DKHHRTSSVRGR---GERADRTTNMLLAIVFVMLFTEMPQGIMALLSGMISQEFRRHIYNNLGDLLDLFSLCEACMSFIIYCSMSGQFRNEFRRVFIP--
       C. elegans DMSR-1   EKLGRNGSLRGR---GERVDRTTHMLLAIVAVMLVTELPQGIMAVLSGMCSEEFRIYIYNNLGDILDLFSLCGSCCSFIIYCSMSGQFRNEFHRVFVP--
         S. ratti DMSR-1   MPKMSSGKKISKGGKHAKTDRTTRMLLTIVCVFLITEIPQGVMAVLTGIFSDEFRIHIYNSFGDFLDLMALCNACTTFIIYCTMSSQFRTEFQKVFMPEK
    B. xylophilus DMSR-1   -----------------RADRTTRMLLAIVCVFLVTELPQGIMAVLIGLLSQEFRKNVYNNVGDILDLLSLCEACTSFIIYCSMSGQFRNEFRRVFIP--
       G. pallida DMSR-1   ----------------SRTDRTTRMLLAIVCVFLITELPQGVMAVLNGMFSEEFHYNIYNRVGDILDLLSLCNACTSFIIYCTMSAQFRNEFRRVFLPAY
         M. hapla DMSR-1   ----------------HRTDRTTRMLLAIVCVFLVTELPQGIMAVMSGMFSEEFRSHIYNNLGDILDLLSLCNALASFIIYCSMSAQFRNEFRRVFLPTT
     M. incognita DMSR-1   -----------------RTDRTTRMLLAIVCVFLVTELPQGIMAVMSGMFSEEFRSHIYNNLGDILDLLSLCNALASFIIYCSMSAQFRNEFRRVFLPTT

            Consensus                R       RADRTTRMLLAIVCVFLITELPQGIMAVLSGILSEEFRRHIYN LGDLLDLLSLCNACTTFIIYCSMSGQFRNEFKRVFLP S

        B. malayi DMSR-1   FFDHKLRWNLKRNYKES--------SS--HSTTDASSKNAFELPRNVLDRNIEGVCPAVTATSELPMESSMTWSQCAAENNLPEKLNTNIGNTENDEDAS
     W. bancrofti DMSR-1   FFDYKLRWKLKRNYKES--------SS--HSTTSASFKNAFELPRNALNRNIEGVCPAVTATSELPMESSMIWSQY-AENNVPEKSNRNIGNMVNDEGPS
       D. immitis DMSR-1   LFGFKLKWKSKRDYK-----------------------------------------------------------------------------------
      O. volvulus DMSR-1   LFGYKLKWKSKQGYKQP--------SSSAQSSTGTNSKTNFELHWYVL-RNTKHNGPLVSVTSELPMEGPWLNYVG----NDLEKLSTNIGNIENDEAYS
       O. ochengi DMSR-1   LFGYKLKWKSKQGYKQ---------------------------------RNTKHNGPLVSVTSELPMEGPWLNYVG----NDLEKLSTNIGNIENDEACS
```

```
           L. loa DMSR-1     LFGYKLTRKLQWNHKES--------SSSTHSSNGRNSKNIFELPANILNRNTKDVCPMVTATSELLMESSLT----------------------------
          A. suum DMSR-1     SSVLCFLSRSNRRSSEPLVKQCYLRPSDAANGNTSTAERSITMTLVGSVASIRKVTKSNNPPASVCTDSLQRMSSSDSVDASPTPKSPPSTAAFNFQSAS
       A. caninum DMSR-1     ----------------------------------------------------------------------------------------------------
  N. brasiliensis DMSR-1     KSVHCVKRP-VSVR----------RPSETY--TKMTYLKPTEVYEN---GSSTYSELDRSGAVKVIGNQSSSR----------LDISRGTPSPGFYGSST
      H. contortus DMSR-1     KSMHCLERP-PSIR----------RPSDAY--TKMTYLRPTDMYEN---GSSTYSELDKNAVVKVG-KLISNF----------IELSFGEIVN-SYGLSA
        C. elegans DMSR-1     AKVRCLRMSSPSIR----------RPSDAYSTTKMTFLKPNEKNGNGMNGNGTYSEDTRSASVKMVGIQVRRNSTEITRMTGCDSITPCSPMPTSFPSSP
          S. ratti DMSR-1     LNNACKIPWNKRSR----------RESVPFTENK------------------------------------------------------------------
     B. xylophilus DMSR-1     AQVSCWWSPQTAR-----------RYSDALFSKTGTQFLQ------------------------------------------------------------
        G. pallida DMSR-1     GRMKCWHRFV------------------------------------------------------------------------------------------
          M. hapla DMSR-1     QRVKCWHQLFVLTR----------RTRNDAKRNENKLERKQES--GCE----------------------------------------------------
      M. incognita DMSR-1     QRVKCWRQLLVLTR----------RRKRNDVEN-IKFEGRQESGGGCE----------------------------------------------------

               Consensus       V CLK                 R S              E                      L


         B. malayi DMSR-1     LLHSSA--------------------------------------------------
       W. bancrofti DMSR-1     LLHSSA--------------------------------------------------
        D. immitis DMSR-1     --------------------------------------------------------
        O. volvulus DMSR-1     V-------------------------------------------------------
         O. ochengi DMSR-1     V-------------------------------------------------------
            L. loa DMSR-1     --------------------------------------------------------
           A. suum DMSR-1     TARISSASHETLDDKSDRLDEKAELLSESCPTKRPCAGILDNKKKGRTLEARYHVV
        A. caninum DMSR-1     --------------------------------------------------------
   N. brasiliensis DMSR-1     LNEAT--AAGFDDERSRLID------------------------------------
      H. contortus DMSR-1     LEMVP--LTVTVIPNS----------------------------------------
        C. elegans DMSR-1     LPPIRSGEDESTDETSHLLNSSGPNSTASADGIRGHFQNI----------------
          S. ratti DMSR-1     --------------------------------------------------------
     B. xylophilus DMSR-1     --------------------------------------------------------
        G. pallida DMSR-1     --------------------------------------------------------
          M. hapla DMSR-1     --------------------------------------------------------
      M. incognita DMSR-1     --------------------------------------------------------

               Consensus
```

14. DMSR-2 homologues

```
      T. spiralis DMSR-2     MVAWIIDNETKGYAERADREAEDAAWVSSGLIAFTNSYGFIHAYLSPIICCIGILANIATIVVLTRPAMRTS-VNVILAAMAACHATTMLTYLIFTLRTN
         T. muris DMSR-2     -------------------------WIRNEIVPIANAYGYVHGYLSTLICVLGILANFATIAVLTRPAMRTS-VNIILAGIAACHAVTMFTYFIFTLRGM
        B. malayi DMSR-2     -------------------------------IPLASAYLPIHIFLYHFLCITGVFANLAIVVVLLRPAMRKNHFNAFLIAIALCDMTLMASYFIFKQVEI
     W. bancrofti DMSR-2     -------------------------------IPLTSAYLPVHIFLYHFLCITGVFANVAIVVVLVRPAMRKNHFNAFLIAIALCDMTLMASYFIFKQVEI
       D. immitis DMSR-2     --------------------------------PLASAYLPIHIFLYHFLCITGVFANLAIVVVLLRPAMRKNHFNTFLIAIALCDMTLMASYFIFKQVEI
      O. volvulus DMSR-2     --------------------------------PLASAYFPIHIFLYHVLCITGVFANLAIVVVLLRPAMRKNHFNTFLIAIALCDMTLMASYFIFKQVEI
       O. ochengi DMSR-2     --------------------------------PLASAYFPIHIFLYHVLCITGVFANLAIVVVLLRPAMRKNHFNTFLIAIALCDMTLMASYFIFKQVEI
           L. loa DMSR-2     -------------------------------IPLANAYLPIHIFLYHFLCITGVFANLAIVVVLLRPAMRRNHFNAFLIAIALCDMTLMASYFIFKQVEI
          A. suum DMSR-2     --------SCMG--LNESAIEEQKVYYAKLLSSAVQAYTPVHVFVYQFLCITGVFANISIIVVLLRPAMRKNPFNAFLIAIAICDMTLMAFYFIYKQVEI
     A. caninum DMSR-2.1     -------MSCMGLALNASERLQLRDYYRQILDPFTAVFSEIHAYLYVFLCVVGVFANIAIVVVLLRPAMRRSPFNLFLICIAICDATLMATYLLYKHVGL
     A. caninum DMSR-2.2     ----------------------------------------------------------------------------------------------------
 N. brasiliensis DMSR-2     -------MSCMGLALNVSELDELRDYYRQILAPFTNVFSQIHSYLYVFLCVVGVFANIAIVVVLLRPAMRRSPFNLFLICIAICDATLMATYLLFKHVEM
     H. contortus DMSR-2     -------MSCMGLALNASELDELREYYRQILAPFTNVFSQIHSYLYVFLCVVGVFANIAIVVVLLRPAMRRSPFNLFLICIAICDATLMATYLLFKHVEF
       C. elegans DMSR-2     -------MSCMG--LNTTELQEARDYFRQLLLPFTASFTMFHNYIYVFLCVIGVFANISIVVVLLRPAMRKSPFNLFLVVIAVCDASLMATYLTYKHVEL
         S. ratti DMSR-2     --------MSSCVEVNETERDAMIQYYLELIGEEIQMYDKIHVYIYQILCLLGVPLNVLVIVVLLRPQMRKNPFNLYLIVIAICDLILMANYYIFHFIER
    B. xylophilus DMSR-2     ------------------DIRHNQNTIFQLLQPVLSFYNQFHVLIYKFLCILGVLSNICIVVVLMRPAMRRNPFNVFLVAIAICDMTLMASYFVFKQVED
       G. pallida DMSR-2     ----------------KESEKACPFHHTQLLNGLSESYNTFHRFIYHFLCIIGVIANICFVVVLLRPTMRKNPFNLFLIAIAICDMTLMASYFMYKQVEM
         M. hapla DMSR-2     ------------------------------------------------------------------------------------------------QIEL
     M. incognita DMSR-2     ---------CTD--LDEESVKQSQIYYTELLSSLSERYDSFHKIIYQTICILGVIANICVVVVLLRPTMRKNPFNLFLIGIAICDMSLMASYFIYKQIEL
               Consensus                                   L PL  AY  IH FLY FLCILGVFANIAIVVVLLRPAMRKN FNLFLIAIAICDMTLMASYFIFKQVEI

      T. spiralis DMSR-2     LTNKCYFPVYDTYFWLVFVVMHADLSVTLHSVSLWLTVDLAIVRYLVLRRP----QAATVANAPRTGIWIMLVTVVAVCACCFLNFLRNSIGARPAPESL
         T. muris DMSR-2     LLNECNVHIYSSYMWQAIVVTHADLSVILQSISLWLTVALAVVRYLVLRRP----RAAAVANAARIGLLTIAGTSVAVCGLCFLNFLRNSIYER-SFRMP
        B. malayi DMSR-2     CH-----PWYFTYFWIVFTYFYAILSVFVHSSSLWLTVNMAVLRYLVLKRSATSTSKIPIVNTFRAAFIAIIAAIVISLLGSLPNMLRYQIRDSGYLNVP
     W. bancrofti DMSR-2     CH-----PWYFTYFWIVFTYFYAILSVFVHSSSLWLTVNMAVLRYLVLKRSATSTSKIPIVNTFRAAFIAIIAAIVISLLGSLPNMLRYQIRDSGYLNVP
       D. immitis DMSR-2     CH-----PWYFTYFWIVFTYFYAILSVFVHSSSLWLTVNMAVLRYLVLKRSATSTSKIPIVNTFKAAFIAIIAAIIISLLGSLPNMLRYQIRDSGYLNVP
      O. volvulus DMSR-2     CH-----PWYFTYCWIVFTYFYAILSVFVHSSSLWLTVNMAVLRYLVLKRSSTSTSKIPIVNTFKAAFIAIIAAIIISLLGSLPNMLRYQIRDSGYLNVP
       O. ochengi DMSR-2     CH-----PWYFTYCWIVFTYFYAILSVFVHSSSLWLTVNMAVLRYLVLKRSSTSTSKIPIVNTFKAAFIAIIAAIIISLLGSLPNMLRYQIRDSGYLNVP
           L. loa DMSR-2     CH-----PWYFTYFWIVFTYFYAILSVFVHSSSLWLTVNMAVLRYLVLKRSATSTSKIPIVNTFKAAFIAIIAAIIISLLGSLPNMLRYQIRDSGYLNVP
          A. suum DMSR-2     CH-----PTYFSYFWIVFTYMYAILSVFVHSVSLWFTVNMAILRYLVLKRSSISRSSLPNVNSFSAAFVAIVLAILISFLGSAPNMLRYQIRDNGFLDVP
     A. caninum DMSR-2.1     CH-----PWFFSHAWMIYTKFYAMFSVFVHSASLWFTVNMAVMRYLVLYRGSHSHTRLPPCNSYPAACIAIVIGAVIALIGSLPNMLRYKIELVREMPVP
     A. caninum DMSR-2.2     ----------------------------------------------------------------------------------------------------
 N. brasiliensis DMSR-2     CH-----PWFFTHAWMIYTRFYAMFSVFVHSASLWFTVNMAVMRYLVLYRGSHPHSRLPPCNGYPAACVAIAVGVVIALIGSLPNMLRYRIKFVEEMAVP
     H. contortus DMSR-2     CH-----PWFFSHVWMIYTRFYAMFSVFVHSASLWFTVNMAVMRYLVLYRGSHAHSRLPPCNGYPAACVAIIVGAVIALIGSLPNMLRYRIKFVKEMPVP
       C. elegans DMSR-2     CH-----PWYFSFPWAIYTKFYAIFSVFVHSCSLWLTVNMAILRFLVLYRGSRSETRIPQCNGFGAAFVAIVLAFAIACIGCLPIFIRYRIIEGEVGAVP
         S. ratti DMSR-2     CH-----PYYYTYFWIIYTKLYAITSVLLHSLSLWLTVIMAIHRYIVVKNSNGAQSN-ISWNTYKITLLGIGAATGISLIGSTPNTLRYEIQDNGLVGAE
    B. xylophilus DMSR-2     CN-----PIYYTYGWVVFTLVYALLSVFVHSASLWLTVNMAVLRFFVLRDSSNTGS--SRWNNYRSAIISVFLALLFSLVGAAPNMLRYEIEDKGPAEVP
       G. pallida DMSR-2     CH-----PWYFTFFWIVYTYGYAVLSVFVHSASLWLTVNMAVLRYLVLKSGSSSSRRWPRLNTYKAASIAVISAVLLAAVGSAPNMLRYQIRNNGQLGVP
         M. hapla DMSR-2     CH-----PLYFTFTWIVFTYSYALFSVLFHSASLWITVNMAVLRYLVLKSSSNSSRNWPRLNTYKAASFSIVAAILIAAVGSAPNMLRYQIFYKGEVPVP
     M. incognita DMSR-2     CH-----PLYFTFTWIVFTYSYALFSVLFHSASLWITVNMAVLRYLVLKSSSNSSRNWPRLNTYKAASFAIVAAILIAAVGSAPNMLRYQV---------
               Consensus     CH     PWYFTY WIVFTYFYAILSVFVHSASLWLTVNMAVLRYLVLKRSS S SKIP VNTFKAA IAIIAAIVISLLGSLPNMLRYQIRD G L VP
```

```
    T. spiralis DMSR-2     CPPAAVAHLAN---------QTVYEIKPPSWYTAEMEAINLWTVG-IVLKVLPCILLSIFIALLIHILIDTRRRQIRLSKKAN------IATGDRTTALL
       T. muris DMSR-2     DGCAGQARDRN---------STTYEIDPPRWWTPELDAINFWIVG-IMMKVLPCILLTIFIALLIHILVDTKRRQVRLAKTSN------VATSDRTTALL
      B. malayi DMSR-2     AYCTSNQSAYAHYYVD-NPKVHAYNIAQPAWWTCRWERFNFWAAG-LVLKLVPCLMLTIFMTLLVRMLIEARERRIRLCRGQTG----DKSQAERTTTML
   W. bancrofti DMSR-2     TYCTSNQSAYAHYYVD-NPKVHAYNIAQPAWWTCRWERFNFWAAG-LVLKLVPCLMLTIFMTLLVRMLIEARERRTRLCRGQTG----DKSQAERTTTML
     D. immitis DMSR-2     MHCTTNQSAYAHHYAD-NPKVHAYNIAQPSWWTCRWERFNFWAAG-LVLKMIPCLMLTIFMTLLVRMLIEARERRVRLCRGQLG----VKSQAERTTTML
    O. volvulus DMSR-2     PYCTTNQSAYAHHYVD-NPKVHAYNIAQPSWWTCRWERFNFWAAG-LVLKMIPCLLLTIFMTLLVRMLIEARERRMRLCRGQTG----VKSQAERTTTML
     O. ochengi DMSR-2     PYCTTNQSAYAHHYVD-NPKVHAYNIAQPSWWTCRWERFNFWAAG-LVLKMIPCLLLTIFMTLLVRMLIEARERRMRLCRGQTG----VKSQAERTTTML
         L. loa DMSR-2     AYCTSNQSAYAHYYVD-NPKVHAYNIAQPSWWTCRWERFNFWAAG-LVLKLVPCLMLTIFMTLLVRMLIEARERRTRLCHGQTG----GKSQAERTTTML
        A. suum DMSR-2     SYCTANGSTYAHYYTE-NSKVHAYNIGQPLWWNCRWERFNFWAAG-LVLKLIPCIMLTIFMTLLVRMLMEARERRTRLCGGTAS----GKSQAERTTSML
   A. caninum DMSR-2.1    KICL---SSKYHSSWTESSTIYKYDLLQPSWWNCHWERINFWMAA-CVLKLIPCVLLTVFMTLLVRMLIEARERRLRLCGGVAM----GNSQAERTTAML
   A. caninum DMSR-2.2    ---------YAHHYTD-NPKVHAYNIAQPSWWTCRWERFNFWAAG-LVLKMIPCLLLTIFMTLLVRMLIEARERRIRLCRGQTG----VKSQAERTATML
N. brasiliensis DMSR-2     KQCLR--SAKYHSSWLVTDTIYKYDLVQPTWWNCQWERINFWMAA-CVLKLVPCILLTTFMTLLVRMLIEARERRSRLCGGVPM----GNSQAERTTAML
   H. contortus DMSR-2     GSCL---ETKYHASWDESDTIYRYDLVQPTWWNCQWERINFWVA--CVLKLVPCVLLTVFMTLLVRMLIEARERRLRLCGGVPT----GNSQAERTTAML
     C. elegans DMSR-2     DLCL---EGKYSTDWQPNDMIQFYGLSQPLWWNCDWERINYWMAA-LILKLIPCLLLTIFMTLLVRMLIEARERRSRLCGGMGN----GNSQAERTTAML
       S. ratti DMSR-2     PRCLVKGGKYAHYYTETS-QIEAYQFVRPTFWNCNWERLTFWIAG-LLLKVIPCMLLTIFMTLLVRILIKARERRNRLCGSRTVLCT-AKTQAERTTAML
  B. xylophilus DMSR-2     HHCLKNGSTYAKFYK-EDELINSYTIGQPVFWNCSWERFSFWVAGGIMLKLIPCLLLTVFMTLLVRMLVEARERRSRLCANNSGSASTGKTQAERTTAML
     G. pallida DMSR-2     TFCVEAGAKYAHSYQ-PGQLVHAYTLGQPAYWGCGLERFSFWTAGTIFMTLL---------------------------------------------VRM
       M. hapla DMSR-2     DVCRANGSKFASFYDNPDIKVDAYVLVQPSFWNCTWERFSFWIAG-LLLKLVPCLLLTIFMTLLVRMLVEAR--------------------------ER
   M. incognita DMSR-2     ----------------------------------------------------------------------------------------------------

              Consensus      C  N S YAH Y D    VHAY IAQPSWW C WERFNFW AG LVLKLIPCLLLTIFMTLLVRMLIEARERR RLC G        KSQAERTT ML

    T. spiralis DMSR-2     LLIVFVFWLTQIPQGIMTIANAFDQAIFDIYLALGDFWDLLSLVNSCVSFPAYCIMSQMFRHEFMTVFGLKRCFKTNS----------------------
       T. muris DMSR-2     LLIVFVFWLTQLPQGIVIIANAFDKAIFHAYLTLGDIWDLLSLVNSCISFPAYCIMSQMFRDEFMIVFGMRRC---------------------------
      B. malayi DMSR-2     TAIVAVFLVTELPQGILVFAIGLKPGIRYAMQYIGDFIDVLSLINSSFNFILCALMSN--VREFLLTFGSCCPKLSENIGGIGSRALTMTHRITQSQRK-
   W. bancrofti DMSR-2     TAIVAVFLVTELPQGILVFAIGLKPGIRYAMQYLGDFIDVLSLINSSFNFILCALMSN--VREFLLTFGSCCPKLSENISGIGSRALTMTHRITQAQRKE
     D. immitis DMSR-2     TAIVAVFLVTELPQGILVFAIGLKPGIRYAMQYLGDFIDVLSLINSSFNFILCALMSN--VREFLLTFGSCCPKLSENINGIGPRTLTMAHRITLSQRKE
    O. volvulus DMSR-2     TAIVAVFLVTELPQGILVFAIGLKPGIRYAMQYLGDFIDVLSLINSSFNFILC--------REFLLTFGSCCPKLSENISGTGSRTLTMTSRIPQSQRRE
     O. ochengi DMSR-2     TAIVAVFLVTELPQGILVFAIGLKPGIRYAMQYLGDFIDVLSLINSSFNFILCALMS--NVREFLLTFGSCCPKLSENISGTGSRTLTMTSRIPQSQRRE
         L. loa DMSR-2     TAIVAVFLVTELPQGILVFAIGLKPGIRYAMQYLGDFIDVLSLINSSFNFLLCALMSN--VREFLLTFGSCCPKLSENISGVGSRPLTIANRITQSQQKE
        A. suum DMSR-2     TAIVAVFLITELPQGLLVFATGIKPGVRYAMQYLGDFIDLLSLINSSFNFILCALMSHVFRREFLLTFGSCCPKSSEDVSGTKS---AAIRAIPSKRNGF
   A. caninum DMSR-2.1    TGIVAVFLVTELPQGILGLAAGVNPRLMFITIPLGNFFDLLSLINSAVNFVLCALMSHVFRREFLQTFSMCCPQSSENHSGAPITKPNK---KSLFASLT
   A. caninum DMSR-2.2    TAIVAVFLVTELPQGILVFAIGLKPGIRYAMQYLGDFIDVLSLINSSFNFILRALMSNVFRREFLLTFGSCCPKLSENISGTGSRTLMMTSRIPQSQRRE
N. brasiliensis DMSR-2     TGIVAVFLITEVPQGVLGLAVGINPRLMFITNPLGNFFDLLSLINSAVNFVLCALMSHVFRREFLHTFSMCCPQSSENHSGAPITKSSNR-TFQFFSSLA
   H. contortus DMSR-2     TGIVAVFLITELPQGILGLAVGINPRMMFITVPLGNFFDLLSLINSAVNFVLCALMSHVFRREFLHTFSMCCPQSSENHSGAPITKPCNK-TFQFFSAFT
     C. elegans DMSR-2     TGIVAIFLITELPQGVLTFAAGANPRLTFLTLQMNNVFDLLSLINSAVNFVLCALMSHVFRREFLQTFGVCCPQSSENHSGAPINKTSNRSILSTFSNIA
       S. ratti DMSR-2     TIIVAVFLVTELPQGIFVFLQGVYAATEVIHLYLGQFLDFLALFNSSVNFILYTTMSYQFRSQFIETFATYLPKTFERISGSESINTNVLSSKISRDGTK
  B. xylophilus DMSR-2     TMIVALFLITELPQGILVFAIGIKPEMRFAMYYLGNILDLLSLLNSSVNFILYSTMSNLFRREFLETFGFCCPQPLRKWNSKRRLASGKASTKSRNPGLG
     G. pallida DMSR-2     LVIVAVFLITELPQGILVVAIGVKPQVRFAMHQLSN---------------IYATMSNLFRHEFLHTFGQCCP---------------------------
       M. hapla DMSR-2     RSLVAIMHITELPQGILVIWMGIRPQVRFAMAQLSNILDFLSLLNSSVNFLLYATMSNLFRHEFLQTFDQCCPK--------------------------
   M. incognita DMSR-2     --IVAVFLITELPQGILVIWMGIKPQVRFAMAQLSNILDFLSLLNSSVNFLLYATMSNLFRHEFLQT---------------------------------

              Consensus    TAIVAVFLVTELPQGILVFAIGLKPGIRFAM YLGDFIDLLSLINSSVNFILCALMSNVFRREFL TFG CCPK SEN SG
```

```
     T. spiralis DMSR-2     --------------------------------
        T. muris DMSR-2     --------------------------------
       B. malayi DMSR-2     ELMAVKCPDEMN--------------------
    W. bancrofti DMSR-2     LMAVVKCPDEMNDNNNT---------------
      D. immitis DMSR-2     LMTVVKCSDEMNDNNNT---------------
     O. volvulus DMSR-2     IMAVVKCNDEMNDNNNT---------------
      O. ochengi DMSR-2     IMAVVKCNDEMNDNNNT---------------
          L. loa DMSR-2     LMVVVKCPDEMNDNNNT---------------
         A. suum DMSR-2     MPLRSSCQDGTN--------------------
    A. caninum DMSR-2.1     PLRK--AKGFLPVPTNEREDRRTDD-------
    A. caninum DMSR-2.2     LLAVVKGPDEMN--------------------
N. brasiliensis DMSR-2     PKKAP-KDGFVPVPTN----------------
    H. contortus DMSR-2     PKKA--KDGFVAVPTN----------------
      C. elegans DMSR-2     KPKTSKKNGFLPVPTNCPDDKNTEQLAMN---
        S. ratti DMSR-2     NNNGNFNTSKVHLLTATTTFQRKDLKGKIDEV
   B. xylophilus DMSR-2     TPALPDPQNGARKAQ-----------------
      G. pallida DMSR-2     --------------------------------
        M. hapla DMSR-2     --------------------------------
    M. incognita DMSR-2     --------------------------------

            Consensus                 M
```

15. DMSR-3 homologues

```
         B. malayi DMSR-3    ---------------------------------------------------------------TQVALCANFFYSSLFKYLSDVCCISQLWSYAWVSTLL
      W. bancrofti DMSR-3    -----------TSEEDEALLAVHIKTAIILVLCTAGITGHVISIYILYSMIN-PNNLFMISMSTQVALCANFFYSSLFKYLSDVCCISQLWSHAWVSTLL
        D. immitis DMSR-3    ---------QMSSEDDEVLLAVHIKTIIILVLCAAGINGHIISIYLLYSMISPNNLFLIGMSCTQVALCANFFYSSLFKYLSDVLCISQLWSHAWVTTLL
       O. volvulus DMSR-3    ---------QMSSEDDELLLTIHIKTAIILALCTAGIAGHVISIYILYSMIN-SNNLFLISMSTQIALCGNFFYSSLFKYLSDVYCISQLWSHTWVSTLL
        O. ochengi DMSR-3    ---------QMSSEDDELLLTIHIKTAIILALCTAGIAGHVISIYILYSMIN-SNNLFLISMSTQIALCGNFFYSSLFKYLSDVYCISQLWSHTWVSTLL
            L. loa DMSR-3    ------------SSDDEALSAVHIKTAMILILCTAGIIGHVISIYVLYSIIN-PNNLFMI----------------------------------------
        C. elegans DMSR-3    MSSNLTDTTTTVFETFLLEYGRIIHPPMVLLLCISGALGHMLTITTLSSMLNPTNMFLISMSCSQLALCINFLYSTFFKFMSDQLCQPFFFSYYLATTMH
   N. brasiliensis DMSR-3    -------------------YGRVVHPPLVLILCISGSLGHLVSIATLSSMMNPTNAFLISMSSSQLALCGNFLYSTFFKWASDELCSPFFFSYYMAKTMH
       H. contortus DMSR-3    -------------------YGRVVHPPLVLILCISGSLGHLVSIATLSSMMNPTNAFLISMSSSQLALCANFLYSTFFKWASDELCYPFFFSYYMTKTMH
     B. xylophilus DMSR-3    ----------------------ILHEPLAMTLCLAGILGHSFSINVLSKMPNPTNLLLISMSSSQLLLCINFLYSTLYKYGSDELCLSSLWSYAWTGTLL

             Consensus                   SEDDE L AVHIKT IILILC AGILGHVISIYILYSMINPTN LLI MSSTQLALCANFFYSSLFKYLSDVLCISQLWSY WVSTLL


         B. malayi DMSR-3    V----------------------------------------------QKCLRIIMLINIGAVLLCIPFFFNSEVRKVASHWKCSVQSSSKSNLSVHEISF
      W. bancrofti DMSR-3    VSVNASVVLYMSGVFHIITLSIIRFISLNQL---------------------------------------------------------------------
        D. immitis DMSR-3    VSVNASVVVHMSGVFHVVTLSIVRFISLNQVLKIKSSEPWFSY----NKCLRTIVLINIGAILLCTPFFFNSEVRKVSSHDECSSRYPYKSNLTAHELSF
       O. volvulus DMSR-3    VSVNASIVVYMSGVFHVVTLSIVRFISLNQFLKIKSSEPWFSY----SKCLRTIVLINISAILLCTPFFFNSEVRKVPSHDECSARYPYMSNLSAHELSF
        O. ochengi DMSR-3    VSVNASIVVYMSGVFHVVTLSIVRFISLNQFLKIKSSEPWFSY----SKCLRTIVLINISAILLCTPFFFNSEVRKVPSHDECSARYPYKSNLSAHELSF
            L. loa DMSR-3    ----------------------------------------------------------------------------------------------------
        C. elegans DMSR-3    FSVTVSVLVHMSAVFHVVALSLIRFFSLAQLSSVNSNVQWFTW----QKSRVAIIMIYVSVIFLCIPLHFTSQVTEVSENEGCAERYPALRNKVAYQLSY
   N. brasiliensis DMSR-3    VSVTLSVLVHMAGVFHVVALSIIRYCSLSSLANINSSQQWFTYQFQKCRISLTIIFVSVAIIGIPL-HFTSEVVMVSE-NEGCAARHPALRNASAFQLQF
       H. contortus DMSR-3    VSVTLSVLVHMAGVFHVVALSIIRYFSLSALANIHSSQQWFT--FQKFRISLTIIFVSVAIIGIPL-HFTSEVVMVSE-NEGCARRHPALRNATAFQLQF
     B. xylophilus DMSR-3    ISVNVSVLVHMTGVFHVVALSIIRYMSLRQLSQTTSHVQWFNMKVSMIYYIFIDRKIFLGFLNFKMGHFTSHIAESNEPDENCLVKYPSLVNTSVHYLEF

             Consensus       VSVN SVLVHMSGVFHVV LSIIRFISL QL  I SS  WFSY     K L TIILI IS I LCIP FF S V  V   E CS RYPAL N SAH LSF


         B. malayi DMSR-3    TNLASSTALGRINFWLLGIICKFIPCIVMIVMTVLLVQKLRQIQLLSIRFTSPIREKRRRCITY------------VEVPQGIILVLSSMQYASFAGSEL
      W. bancrofti DMSR-3    ----------------------------------------------------------------------------------------------------
        D. immitis DMSR-3    TILASNTVFGRINFGLLGTMCKLIPCAIMIVMSVLLIQKLRQIQLLSVRFTSRIREKRRRCITYIILI-IMINFVIVELPQGIISVLSSVKGASFVVSES
       O. volvulus DMSR-3    TILANNTVLGRVNFGLLGTMCKLIPCAIMIVMTVLLIQKLRQIQLLSIRFTSRTREQRLRRITY------------VELPQGIISILSSIKGASFAISES
        O. ochengi DMSR-3    TILANNTVLGRVNFGLLGTMCKLIPCAIMIVMTVLLIQKLRQIQLLSIRFTSRTREQRLRRITY------------VELPQGIISILSSIKGASFAISES
            L. loa DMSR-3    ----------------------------------------------------------------------------------------------------
        C. elegans DMSR-3    TQSIW---LRNLNFWLFYLVAKVVPSIILCIMTCLILDQLKKIQVLSARFSSVERDKQHSRTTNMILA-IMILFIIVELPQGVLAVLSTVT--SVKLIYE
   N. brasiliensis DMSR-3    SSFPY---LQTLNFWLFNLCSKIIPSV-------------------------------------------------------------------------
       H. contortus DMSR-3    SSFPH---LQTLNFWLFNLCSKIIPSVILCLMTFMILAKLKRIQQLSSRFTSVERDKQYQRTTKMILVRVHILFIIVELPQGLLAVAATVT--ELQLIVA
     B. xylophilus DMSR-3    SSNEQ---LQQLNFWLFGFVCKLVPCSVLSMMTILLLRELKKLRELSSRFQNVERDRQYSRTTNMIIV-IMVVFIVVEFPQGVLTVAQSIT—QIPYQDT

             Consensus       T       L  LNFWL G MCKLIPC IM VMTVLLI KLR IQLLS RFTS  REK   R T             VELPQGIISVLSSI   S  I E
```

```
         B. malayi DMSR-3     LGDLFDILSLLNSCVTFGLFCSMSSRIRHAF-------------------------------------------------------
      W. bancrofti DMSR-3     --------------------------------------------------------------------------------------
        D. immitis DMSR-3     LG------------------------------------------------------------------------------------
       O. volvulus DMSR-3     IGDLFDIFSLLNSCITFALFCSMSSRIRNAF-------------------------------------------------------
        O. ochengi DMSR-3     IGDLFDIFSLLNSCITFALFCSMSSRIRNAF-------------------------------------------------------
            L. loa DMSR-3     --------------------------------------------------------------------------------------
        C. elegans DMSR-3     LGDLTELFTLLTSIIIFTLLCSMNGKIRSAFKELSCVRSIGRIFATMCPPPSPVAASISGHDALLEHNTIIITNCHIDKSENYAVL
 N. brasiliensis DMSR-3     --------------------------------------------------------------------------------------
      H. contortus DMSR-3     LGECLMNGKIRSAFKEAPCFRWLER-------------------------------------------------------------
    B. xylophilus DMSR-3     LGDLFESLTLLTSCIIFGLFFTMNSRLRDEFME-----------------------------------------------------

             Consensus        LGDL D  SLL S I F LF SM  RIR  F
```

16. DMSR-4 homologues

```
        A. suum DMSR-4     -MCDRSARLFDMEDPYTLSFIKGLQKFSKYYSSVHEGICMVICIFGIMSNLVHILVLTRAPLRRCAVNCVLTAVAVCDVLTMSSYVVYLLRFRFFAVSRG
     A. caninum DMSR-4     ----------------------------------------------------------------------------------------------------
N. brasiliensis DMSR-4     -MCNEGRSLLDLNDSLVLSFVNRLEQFNKVYTVFHRYACLVICAIGVISNAVHILVLSRPRMRRCAVNCVLTAVAFCDVFTMTSYIVYLFRFRIYQGENG
    H. contortus DMSR-4     ----------------------------RVYTVFHRYACLVICLIGIISNAVHILVLSRPRMRRCAVNCVLTAVAFCDVVTMTSYTVYLFRFRIYQGEYG
      C. elegans DMSR-4     MCQDEGGTLFDPQDPAVSGLIDALEQFQSFYTYFHRYACLFICIVGVFSNAIHIAVLSRPRMRRCAVNSVLTAVAFCDVITMTSYSIYLMRFRFYETDHG
   B. xylophilus DMSR-4     ------------------------------YNAIHPFLCLFICPLGILANLVHILVLTRRRMRRCAVNSVLIGIAICDVITMTSYFIYIVRFEFAKGLTK
        M. hapla DMSR-4     -------LFDRNSTTQLAVIQSLSKIAGIYNLFVHPFLCLLICPLGVLANFVHILVLTRRKMRRCAINNCLIGIAVCDVFTMTSYFIYIVRFEIWTRIS-
    M. incognita DMSR-4     ---------------------MALDNTGLYNLFVHPFLCLLICPLGVLANFVHILVLTRRKMRRCAINNCLIGIAICDICTMTSYFVYILRFEIWTRITS
      G. pallida DMSR-4     ----------------------------LYNNHVHAYLCLLICPLGILANLVHILVLTRRRMRRCAINNCLMGIAICDIFTMFTYLVYILYFEIYAKIYQ

              Consensus                                  YY  VH YLCLLIC LGILAN VHILVLTR RMRRCAVN VL AIAICDV TMTSY VYILRF IY

        A. suum DMSR-4     ---YPYIWIAYLLSHAVFTIALHAITLYLSVALAFIRWQALGNVHS-PWLLPSSAWKLFGVCTIVVSILCIPTMLVHRIVKMP--------LKILPLVSE
     A. caninum DMSR-4     ----------------------------------------------------------------------------------------------------
N. brasiliensis DMSR-4     ---YSYSWMMFLKIHVVVSIALHAITLYMGAVLAFIRWQALGNIHS-QWLQPRSAWKLFAFVTATISVMCVPTVMLHEIFVVS--------DSKEHEFSE
    H. contortus DMSR-4     ---YSYSWMLFLKIHVVVSIALHAITLYMGAALAFIRWQALGNIHS-RWLQPRSAWKLFGIVTASISMMCVPTVMLHEIYEIS--------NFAEQASST
      C. elegans DMSR-4     ---YSYIWLVFLKFHVWSSMTLHAITLYMGGALAFIRWQALGNIHS-KWLQPRNSWQLFGVVSVVLSIVCLPTLVLHKIYEIESPEIETSTVSEVLRLSG
   B. xylophilus DMSR-4     ---FSYFWAVVLEFHATLSICLHTINLYLCVTMAFIRWKAMRTSKS-AVMKPNTAWKIFCVIALVVSVLCVPTFLVHEAHEVNLTVENTTYSFYTVDISD
        M. hapla DMSR-4     -G-ISHFWATYLHFHATSSICLHTIALYMCVTMALIRWRVMSQPFSKVVVQPIISWKIFGLTTVVVSAFCIPTYLVHDVISF-------GANFFSVDISE
    M. incognita DMSR-4     IKTISYFWATYLHFHATSSICLHTIALYMSVTMALIRWRVMSQPFSKVVVQPILSWKIFGSVTTVVSMLCIPTYLVHDVINF-------GAHFFSVDISE
      G. pallida DMSR-4     TN-ISHFWATYLHFHATVSICLHTITLYTCVTMALIRWRVMSQPYS-LIVQPATVWKIFALVTVLVCFFCVPTYLVHDVVKFRY---ADGTTFFTVDISE

              Consensus        YSY W  YL FHA  SI LH ITLYM V LAFIRW AL N HS   LQP SAWKIFGVVTVVVSILCVPT LVHDI  I          F  VDISE

        A. suum DMSR-4     PATLIAKELYTLDMVEDACSLFQFNLWLTAIFLKAVPCVLLLWFTFALVSKLRDTDIKRNDLYSKS-FRKHMRKTVVPDKTTYMLVIMISVFLATELPQG
     A. caninum DMSR-4     ----------------------------WILHFQAIPCGLLLWFTLALVLKLRKTDEKRNYLYSKS-FRKHIKKTTVPDRTTYMLIIMLVVFLVTELPQG
N. brasiliensis DMSR-4     PS-------YTLDFANYSCGFYKFNLWMLAVVMKAIPCGLLLWFTVALVLKLRKTDEKRNYLYSKS-FRKHIKKTTVPDRTTYMLIIMLVVFLVTELPQG
    H. contortus DMSR-4     PS-------YTLDFSNYSCGFYKFNLWMLAVAMKAIPCALLLWFTVALVMKLRKTDEKRNYLYSKS-FRKHIKKTTVPDRTTYMLIIMLVVFLVTELPQG
      C. elegans DMSR-4     QSIP-KEVRYSLNFSTYSCAFFKFNLWMLAIVLKAIPCALLLWFTIALVVKLRQTDEKRNYLYSKS-FRKHVKKTTVPDRTTYMLIIMLVVFLVTELPQG
   B. xylophilus DMSR-4     WAR---------MN---RCRWFKANLWIIGIVLKAIPCFLLLWFTMALMIRLHKNNAKRAMLLYTNSVKGARKRRQNYDRTTFTLIVMLTAFLVTELPQG
        M. hapla DMSR-4     LAR---------SN---NCRYFKLNLFIIGIVLKAIPCFLLLWFTLALMRRLRENNAKRALLLKGH-----NKKLLNYDRTTFTLIVVLGVFLVTELPQG
    M. incognita DMSR-4     WAR---------SN---NCRYFKLNLFIIGIVLKAIPCFLLLWFTLALMRRLRENNAKRAQLLKDQ-----NKKSLNYDRTTFTLIVVLGVFLLTELPQG
      G. pallida DMSR-4     WAR---------TD---HCHYLKANLFIIGIVLKAIPCFLLLWFTMALMIRLRENNAKRALLLRDK-----CKKLQNYDRTTFTLIVMLGVFLMTELPQG

              Consensus     A                C FFK NLWIIAIVLKAIPC LLLWFTLALVIKLR TD KRN LYSKS FRKHIKKT VPDRTTYMLIIML VFLVTELPQG

        A. suum DMSR-4     FLALLNGVYTADVHRFIYVNVGDVLDLMSLINCSVDCVLYCMMSSRYRHTFNHILIRAESWLRNKREERQYEKHWRTSTDNNPAKLGI------------
     A. caninum DMSR-4     FLALLNGVYTTDVNNYIYQHVGELFDLLSLVNCR------------------------------------------------------------------
```

```
N. brasiliensis DMSR-4     FLALLNGVYTTDVNNYIYQHVGELFDLLSLVNCSVDFVLYCFMSSRYRQTFGHILIRVESWLRNQSSGWKYSKDLTKVAPPTV-----------------
   H. contortus DMSR-4     FLALLNGVYTTDVNNYIYQHVGELFDLLSLVNCSVDFVLYCFMSSRYRQTFGHILIRVESWLRNQSSGCKYSKEFSKTPPPTI-----------------
     C. elegans DMSR-4     FLALLNGLYTGDVNIYIYKNLSELLDFLSLINCSVDFLLYCVMSSRYRQTFGHMLIRVESWLRNHGERRRLAREIKKKLPAPVGV---------------
   B. xylophilus DMSR-4    MLTMLNGVYTNDVHTVIYMNLANVLDVLSLINCYVGFIAYCFLCSKYRQTFMMMIL--------------------------------------------
       M. hapla DMSR-4     VLTILNGVFTNDVHTVFYMNLANILDLLSLINCYVGFIAYCFLCTKYQQTFIMMIITWLE----------------------------------------
   M. incognita DMSR-4     VLTILNGIFTNDVHTVFYMNLANLLDLLSLINCYVGFLAYCFLCTKYQQTFVLMIFNCISTLFKCFGVKRNTVITNQERRKRASRKQSAHKRSLVRSSYN
     G. pallida DMSR-4     ILTILNGIYTNDVHSVFYMNLANLLDILSLINCYVGFMAYCFLCSKYRQTFGLMILTSLDSYRKLVCHEKYGQQGQKHRRSHRHHRNSQNE--LLRRPEN

          Consensus        FLALLNGVYT DVH YIYMNLAELLDLLSLINC V FV YCFL SKYRQTF  MII   S LR      K


        A. suum DMSR-4     ------------
     A. caninum DMSR-4     ------------
N. brasiliensis DMSR-4     ------------
   H. contortus DMSR-4     ------------
     C. elegans DMSR-4     ------------
   B. xylophilus DMSR-4    ------------
       M. hapla DMSR-4     ------------
   M. incognita DMSR-4     YSKTDCEMPQRH
     G. pallida DMSR-4     ------------

          Consensus
```

17. DMSR-5 homologues

```
   A. suum DMSR-5    DDFKNCILMMSMCDDDAYLFDIDN-TTQLFLYRLHLFSSVYTIFHRYFCALICFCGVTVNLVHIAVLTRKQMRCFAVNSILSVMAICDVIIMSFYFVYII
N. brasiliensis DMSR-5    ------------CQNDPPLFDLKDNATAEFFGTLIEFSRVYTHVHRYLCLVVCLVGIVLNTLHFFVLSRRPMRTYIINALLCAMAICDALTMLSYLIYIL
H. contortus DMSR-5    ------------CQNDPPLFDLEDNATIQFFHELQAFSRIYTGFHRFICLVVCIVGISLNILHFYVLSRRPMRAYIINAILCAMAVCDALTMSSYLIYII
C. elegans DMSR-5    -----------MCTQDLPLFDLENNSTIYFFDQLIAFNQVYTVLHRYLCLFVCFFGVLLNSLHFYVLTRKAMRVYIINALLCAMSICDIITMTSYFIYIL
G. pallida DMSR-5    -------MRLMICDEELSLFDLNSNTTVAFLEALWGISTLYNQVHCWLGLFICVFGSLANCVHIFVLSRPAMQKCAVNRLLAIIALCDIATMASYTVFVV
M. hapla DMSR-5    -------MKLMICDEELSLFDLNSSTTIAFLETLWNISSLYNQIHCYVGLFICVFGSLANVVHIFVLSRPAMQKCAVNRLVRIIAICDICTMFSYTVFIV
M. incognita DMSR-5    -------MKLMICDEELSLFDLNSSATITFLETLWNISNLYNQIHCYVGLFICVFGSLANVVHIFVLSRPAMQKCAVNRLLSIIAICDICTMFSYTVFIV

Consensus           M L ICDEDL LFDL  NTTI FLE L  FS LYT IHRYLCLFICVFGILLNVVHIFVLSRKAMR YAVNALL IMAICDIITM SY VYIV

   A. suum DMSR-5    RFRVIENED-DTLGYTYGWLVFLLIHVVLSIALHTIGLYLSVFMAYVRWVALDRLDSKWIRTRPVGRIFFAIAITIFALSIPTLLVHEIVPFKHFTTVNE
N. brasiliensis DMSR-5    RFRIFDNDV-GIIGYSYPWLVFLIAHVTSSIALHTCSLYLSVTMAYIRWTALDRLDAKWINNSAIKQIFFLTAFSVTFISIPTLMVHKIVSVNDILGTND
H. contortus DMSR-5    KFRIFDNN--GVIGYSYPWLVFLIIHVTSSIALHTCSLYLSVTMAYIRWTALDRLDAKWINNSAVRHIFFFTAASVAFISIPTLMVHKIVPVSEVLETND
C. elegans DMSR-5    RFRIFDTPS-TIIGYSYPWLIFLITHVTSSIALHTTSLYLSVIMAYIRWTALDRLDAKWINHGALKQILIFTALIVSVISIPTVMVHKIVPVTEVLGMNE
G. pallida DMSR-5    RFEFAVDKEEPPLGYDFFWITFLLAHVVCSIALHTITLYLTVVTAFIRYKTLKRVGSLWNHENIACPIFLTVAVLTVFLCIPTFLIHSIIPVTSTDPITQ
M. hapla DMSR-5    RFGFAVDPDEPPVGYNFIWIIFLLAHVVCSIALHTITLYLSVATAFIRYKTLKQIGSLWNHENIALPVFLLVAAFTVILCIPTFLIHSIIPVVAQNAKTG
M. incognita DMSR-5    RFGFAVNPEEPPVGYNFIWIIFLLAHVVCSIALHTITLYLSVATAFIRYKTLKQIGSLWNHENIALPVFLLVAAFTIILCIPT-----------------

Consensus    RFRI DN D   IGYSY WLIFLLAHVV SIALHTISLYLSV MAYIRW ALDRLDSKWI    V  IFL VA  V  LSIPTLLVH IVPV      ND

   A. suum DMSR-5    NYTINLIDNITYYTVGLIPQATDNSCRLFKANLWLTGIFFK-VIPCVLLFWFTVALMRKLNETSRKRIFLLGE--------------------------
N. brasiliensis DMSR-5    TQSNNLYT-------VTLDESDFTGCTLFRANLWITGIFFK-AVPCVLLLWFTIALIWKLCEMSEKRRQLRGDS-------------------------
H. contortus DMSR-5    TQYDNLYT-------VQLDEIDFNGCTLFRANLWLTGIFFK-ALPCALLLWFTIALIWKLCEMSEKRRQLRGDN-------------------------
C. elegans DMSR-5    TDLLEGAKFDGLYT-VQLDETKINGCALFRVNLWITGIMFK-ALPCLLILWFTIALIYKLVQMSEKRKILRGEKRENVEFQLLATSATPTTTTRNEASPR
G. pallida DMSR-5    HNITLFTV-------GLAEFEQFDTCLFFKLNLWLTGIIFK-VIPCVLLLLFTLALLYELEKNHRKRKLMCSGTT------------------------
M. hapla DMSR-5    ENTTLYTV-------GLAEFKRLDTCFFFKLNLWMTGIIFKVVIPCILLLLFTLALLYELEKNRRKRKGMCSGAT------------------------
M. incognita DMSR-5    ---------------LAEFKRLDTCFFFKLNLWMTGIIFK-VIPCILLLLFTLALLYELEKNRRKRKGMCSGAT------------------------

Consensus     N              LLD     TC LFKLNLWLTGIIFK VIPCVLLLWFTIALIYKL   SRKRK L GD

   A. suum DMSR-5    ------KKKKMAMDKTTLMLIIMLFVFLCTELPQGALAILNAMYTSDIHAYIYMNLGELLDLLSLINCNVGFVLYCCMSSRYRSTFRHTFWLP--ASAAA
N. brasiliensis DMSR-5    ----NNKCTKLSIDKTTLMLIIMLVVFLCTELPQGLLAILSALYPTHVHTMVYLNVGEMLDLLSLVNCLTSFVLYCCMSSTYRATVYHLLMPNKKTPAQK
H. contortus DMSR-5    ----FRKCTKLSMDKTTLMLIIMLVVFLCTELPQGLLAILSAMYPSHIHTMVYVNVGEMLDLLSLVNCLTSFVLYCCMSSTYRSTVRALIMPSRTTPARK
C. elegans DMSR-5    LRKVSQCSRNVSIDRTTLMLIIMLVVFLCTEMPQGLLSILSAIYPTHVHTMIYVNVGEVLDLMSLINCLTSFIVYCVMSTTYRATVKSVLCRSTSRRTVF
G. pallida DMSR-5    ---LAYRRHSAQSDRTTKILLILLIVFFCTEFPQGILAILNGLFPNDIHQFVYLSLGELLDLLSLINCNTCFIVMYCSNSSFFLYFK-------------
M. hapla DMSR-5    ---LAYRRHSAQSDRTTKILLILLTVFFSTEFPQGILAILNGLFPNDIHQFVYLSLGELLDLLSLINCNTCFIV-------------------------
M. incognita DMSR-5    ---LAYRRHSAQSDRTTKILLILLTVFFSTEFPQGILAILNGLFPNDIHQFVYLSLGELLDLLSLINCNTCFIV-------------------------
```

```
        Consensus        LA KR  LSIDRTTLMLIIMLVVFLCTELPQGILAILNALYPSDIH FVYLNLGELLDLLSLINCNT FIVYCCMSSTYRAT K

      A. suum DMSR-5     SAISANFRKFSFSV-
N. brasiliensis DMSR-5     TTLAAQSLKLLIS--
   H. contortus DMSR-5     NALAAQSLKLLMS--
     C. elegans DMSR-5     KNTHLLLDSYRIQVS
     G. pallida DMSR-5     ---------------
       M. hapla DMSR-5     ---------------
   M. incognita DMSR-5     ---------------

        Consensus
```

Comment [AM1]:

18. DMSR-6 homologues

```
      B. malayi DMSR-6     MECPNSPQLFDEKDAIALSILSIVNEFCL-SYRKLHKYVSMILCVFGLLANCTHIWILRRPSMXRSSVHTVLICIALADSGTMSSYAIYLLRYEFCASST
    W. bancrofti DMSR-6     MECPNSPQLFDEEDTIALSILSAVNEFCL-SYRKLHKYVSMILCVFGLLANCTHIWILRRPSMLRSSVHTVLICIALADSGTMGSYAIYLLRYEFCASST
     O. volvulus DMSR-6     MECPNYPQLFDENDITVLSILSTLSELCLRFYRTFHKYVSMLLCLFGLLANCTHIWILRRPSMLRSSVHTVLICIALADIGTMSSYAIYLLRYEFYTSST
      O. ochengi DMSR-6     MECPNYPQLFDENDITVLSILSTLSELCL-FYRTFHKYVSMLLCLFGLLANCTHIWILRRPSMLRSSVHTVLICIALADIGTMSSYAIYLLRYEFYTSST
         A. suum DMSR-6     ----------------------------FRFYRSFHRYISLLLCLFGLFFNFVHIWVLTRPRMLQSSVHTVLTCIAIADIGTMTSYLIYLLRYEFSRDEE
      A. caninum DMSR-6     ----------------------------------------------------------------------------------------------------
 N. brasiliensis DMSR-6     ---CKDPQLFDMNNNITHSVLQTLFLFQQ-TYSAVHRYVAVLLCLFGLLANSIHIWVLTRPRMQFSSVHTLLVCIAVSDMGTMTSYLIYMTRFEFMQDHR
     H. contortus DMSR-6     ---CKDPPLFDVDNNFTHNILQTLFLFQQ-TYSSFHRYISVLLCLFGLLANSIHIWVLTRPRMRFSSVHTVLVCIAVSDMGTMTSYLIYMTRFEFMQEST
      C. elegans DMSR-6     -MECNDSRLFDTNNNLTYFVIEQFYRFRY-LYSNVHPFLSFILCVLGLAANAIHITVLTRPRMRHSSVHTVLVCIAISDMGTMTSYLMY-ISRFEFLSDK
        S. ratti DMSR-6     -LNCTSLQLFDYNSDFSTKIILNLFSFKS-YYQPFHKYISVILCLLGIITNNIHIWVLSRKQMRFQSVHIVLIFIASADIGTMLSYLIYIIR-FEFFN-D
  B. xylophilus DMSR-6     -------------------------------YGTIHRYVALILCLGGLVINCIHICILTLPRMRNSSVHTTLTCIALADIGTMTSYLVWYLTRFEYFSST

             Consensus           PQLFD        IL  L  F    Y   HKYVSMILCLFGLLAN IHIWILTRP M  SSVHTVLICIALADIGTMTSYLIYLLRYEF  SST


      B. malayi DMSR-6     GGYSYGWVLLLKLHVVLSTALHSISLYLLVFITYIR--ICSVQTQRSQLLETRVAGLISLLISFAIFALCIPTLLAHDIVLQENSPHFIGLKEIHPTDLS
    W. bancrofti DMSR-6     GGYSYGWVLLLKLHVVLSTALHSISLYLLVFITYIR--ICSVQTQRSQLLETRVAGFISLLISFAIFALCIPTLLAHDIVLRENSPHFIELKEIHPTDLS
     O. volvulus DMSR-6     GGYSYGWVLLLKLHVVLSTALHSISLYLLVFISYIR--LCSMQTQRSRLLETRIAGLISLLIALVIFALCIPTLLAHDILPQENN-SYLIKSTTLPTNFS
      O. ochengi DMSR-6     GGYSYGWVLLLKLHVVLSTALHSISLYLLVFISYIR--LCSMQTQRSRLLETRIAGLISLLIALVIFALCIPTLLAHDILPQENN-SYLIKSTTLPTNFS
         A. suum DMSR-6     NGYPYGWVLFLKIHAVTSIALHAVSLYLVVFMAYIR--LCAIRTTNSRWLQTRLAGFTSLMIALVVFILCVPTLLAHEIYAQKRTLQYYTTVTENEHTSS
      A. caninum DMSR-6     ---------------LISIALHAITLYLVVLMAFIR--FSAMRLSSSEWLRSERSRIAAGGIAVSVFVLCVPTLLAHEIYSRRAISRQG-----------
 N. brasiliensis DMSR-6     G-YPYSWALFLKIHVVLSIALHAITLYLVVLMAFVR--YSAMRVATSGWLQPRRSILAAIGIAICVFVLCVPTLLAHEITARRAVSTQGEVSKRYSIGFS
     H. contortus DMSR-6     G-YPYGWAMFLKIHVVLSIALHAITLYLVVLMAYIR--YRAMRVVSSEWLKSSRSMFTAIVIAICVFVLCIPTLLSHDILARPPDSASGRQTALYSIGFS
      C. elegans DMSR-6     EGYSYFWALFLKCHAMLSIALHAITLYLVVLMAFIR--LSAMKLTTSRWLDHTRALTSAIFIALFVFIMCVPTLLAHQIDETTRGVTMN-----------
        S. ratti DMSR-6     EGYAYKWTVFLHFHASVSIALHGLSIYLVVFMAFIRCQVMKVKTGQSIWMLPHVALLSASVITLTLFILSIPTYLAHEIVERTTT---------------
  B. xylophilus DMSR-6     QGYAYAWALFLQVHAVLSIALHALSIYLVTLMAFLR--LMAMNAGQSKWMKPRLALTASLFVTITVFTICIPTFLAHEVKQNSAA---------------

             Consensus     GGYSYGW LFLKLHVVLSIALHAISLYLVVFMAYIR  L AMRT  S WL TRVA L SLLIAL VFILCIPTLLAHDIL                   S


      B. malayi DMSR-6     ----ESDSSSISNKYSIGLSKMFVNNDCFLLKLNLWLTGIMMKVIPCILLLFLTYCLLVKLAKNKKK---RVALLRERAKDRVYKDHTTCMLLLMVSSFL
    W. bancrofti DMSR-6     ----ESYPSSISNKYSIGLSKMFVSNDCFLLKLNLWLTGIIMKVIPCILLLCLTYCLLVKLAKNKKK---RVALLRERAKDRVYKDRTTCMLLLMVSSFL
     O. volvulus DMSR-6     ----IPDPNPVTNKYTIGLSKMFVSNDCFLLKLNLWLTGIMMKV----------------AK-NKKK---RMALLRDRAKEKSYNDRTTYMLLLMVSIFL
      O. ochengi DMSR-6     ----IPDPNPVTNKYTIGLSKMFVSNDCFLLKLNLWLTGIMMKVTPCMLLLCLTYCLLIKLAKNKKK---RMALLRDRAKEKSCNDRTTYMLLLMVSIFL
         A. suum DMSR-6     SEYNQEYYDSVDAKYSIGFSAMVLQNNCMLLKMNLWLTGIMLKAVPCFLLLWLTFALLVKLRANSKKRMLRLGMKKDKRKDGSLRDRTTSMLLLMLCVFL
      A. caninum DMSR-6     ----------------------------------------------------------------------------------------------------
 N. brasiliensis DMSR-6     ------------------D--MVLENSCLLLKGNLWLTGIFLKAIPCVLLFWFTIALIGRMKENKEK---RKKLLKSRD-DKGKRDVTTYMLVFMVAVFL
     H. contortus DMSR-6     ------------------E--MVLENGCFLMKGNLWLTGIFLKAIPCVLLFWFTIALIGRMKENKEK---RKKLLKSRD-DKGKRDVTTYMLVFMVTVFL
      C. elegans DMSR-6     ---------GMYYKYSVGFSTLMMQNGCSLMKGNLWLTGIFLKAIPCLLLLTFTIALINRLRENNEK----RKILIKEERAKKRGDFTTYMLLLMVTVFL
        S. ratti DMSR-6     -----K---DGIIKYTIIIARNYRANDCVAFKIYLWVTGILIKAVPCFLLMFFTLGLLKKLRENQKKRNKLLSRNRNSSKRQNTPDRTNKMLLLMVTIFL
```

```
    B. xylophilus DMSR-6     -------------YYNVGFSALFMENGCNLLKLNLWLTGIFLKAVPCFLLMCFTAALLRRLRANDKK----RQLLFHGESRRSNSDRVTYMLLLMVFVFL

            Consensus                  V  KYSIG S MFV N C LLKLNLWLTGIMLKAIPCILLL  T ALL KLR NKKK   RL LLR R KDK   DRTTYMLLLMVSVFL

        B. malayi DMSR-6     CTELPQGIIAIFNAIYTAQFHFYVYLTIADILDVLSLINCYVGFTVYFCXCSRYRRTFYSIIPYFGFV--------------------------------
      W. bancrofti DMSR-6    -----------FPAIYTAQFHFYVYLTIADILDVLSLINCHVGFTVYFCTCSRYRRTFYSILPYFGFV--------------------------------
      O. volvulus DMSR-6     CTELPQGIIAIFNAIYTAQFHLYIYLTIADVLDVLSLINCYVGFTVYFCTCSRYRRTFYSV--------------------------------------
       O. ochengi DMSR-6     CTELPQGIIAIFNAIYTAQFHLYIYLTIADVLDVLSLINCYVGFTVYFCTCSRYRRTFYSV--------------------------------------
          A. suum DMSR-6     STELPQGVMAVLNAIYTKQFHLFVYLPLADVLDLLSLINCYVGFTVFFCTCTRYRQTLLTLFPQQCGCVTRSQTPTHNSMLLQQGVPKRYYTPQIDEHLT
       A. caninum DMSR-6     -----------------------------------------VWFLVYVCTSSRYRQTLLSILPNMP----------------------------------
  N. brasiliensis DMSR-6     TTELPQGVMAVLNALYTSQFHAYVYLSLADVLDLLSLINCYVAFLVYICTCSRYRQTLLSILPERPLILSAATSHSTMK---------------------
      H. contortus DMSR-6    ITELPQGIMAVLNAIYTSQFHSYVYLSLADVLDLLSLINCYVAFLVYICTCSRYRQTLLSILPNMP----------------------------------
        C. elegans DMSR-6    FTELPQGIMAILNALFTTQFHQMVYLNLADVLDLLSLINCYVAFLVYSFTSSRYRQTLFSLLPLTKISYSGISTRQGTLKSHQHPGAKTLVQRANSVEVA
          S. ratti DMSR-6    ITELPQGICAILNALFTQQFHEIVYSNLADVLDLLSLINCFVAFIVYTSTCSRYRRLLLDVLKPL----------------------------------
     B. xylophilus DMSR-6    VTELPQGLFAILNAMFTYQFHRYVYVCFADLLDILSLINCYVCFSVYTATSSTYRQTLLMLVP-------------------------------------

            Consensus         TELPQGIMAILNAIYTAQFH YVYLTLADVLDLLSLINCYVGFTVY CTCSRYRQTLLSILP

        B. malayi DMSR-6     ---------------------------------
      W. bancrofti DMSR-6    ---------------------------------
      O. volvulus DMSR-6     ---------------------------------
       O. ochengi DMSR-6     ---------------------------------
          A. suum DMSR-6     SKNIAQVSIMSTMQTTVTNGVTTILATEADAFV
       A. caninum DMSR-6     ---------------------------------
  N. brasiliensis DMSR-6     ---------------------------------
      H. contortus DMSR-6    ---------------------------------
        C. elegans DMSR-6    SARSPLVDKAAITRPNSAQPTDF----------
          S. ratti DMSR-6    ---------------------------------
     B. xylophilus DMSR-6    ---------------------------------

            Consensus
```

19. DMSR-7 homologues

```
     T. spiralis DMSR-7    --------------------EIFLNVINQFHQ-QYQAVHGHLCIVLCLFGIVTNILNCIVLTRRQMR-SASNMFLAIIALIDMGLMVLYLVYVAHFVVVQ
        T. muris DMSR-7    ------CSFSRSDLLTGNASAVFLDTLNQFHL-RYQHVHGHLCIGLCLFGIVTNIVNFIVLTRSHMR-SASNMFLAIIALIDMSLMVLYLVYIARFVVIE
       B. malayi DMSR-7    ---MLCVNDTPLFDLSDNATRLFLAALEAFQR-SYSPVHGFICIVICTFSIGTNLIHILVLTRPNMRCSAVNCVLTMVAICDMGTMGSYFIYICHFVLFK
    W. bancrofti DMSR-7    ---MLCVNDTPLFDLSDNATRLFLAALEAFQR-TYSPVHGFICIVICTFSIGTNLIHILVLTRPNMRCSAVNCVLTMVAICDMGTMGSYFIYICHFVLFK
      D. immitis DMSR-7    ---MLCVNDTPLFDLSDNATLLFLATLEVFKR-SYSPVHGFICIVICSFSITTNLVHILVLTRPNMRCSGVNCVLTAVATCDIGTIASYFIYICHFVLFK
     O. volvulus DMSR-7    -------------------------------R-IYSPVQGFICIVICTFSIVTNLVHTLVLTRPNLRCSGVNCIL-------------------------
      O. ochengi DMSR-7    -------------------------------R-IYSPVQGFICIVICTFSIVTNLVHTLVLTRPNLRCSGVNCIL-------------------------
          L. loa DMSR-7    ---MLCVNDTPLFDLSNNTTRLLLAALEAFQR-AYSPIHGFICIVICTFSIGTNLIHVLVLTRPNMRCSAVNCVLTMVAICDMGTMASYFIYICHFVLFK
         A. suum DMSR-7    ---MLCTNDTPLFDFSSNSTKRFIQFLATFQQ-AYAPVHGFICVAICTFGIITNLVHVLVLTRPSMRCSAVNCVLTAVAICDMGTMGSYLVYICHFVLRK
      A. caninum DMSR-7    ----------------------------------------------------------------------------------------------------
 N. brasiliensis DMSR-7    ---MECPNDTPLFDFSQNATRNFISTLSNFRN-LYAPIHGYLCIGLCVFGILTNLVHVVVLSRPVMRNSAVNCVLTAVALCDIGTMVTYLVYILHFVFMR
     H. contortus DMSR-7    ---MECPNDTPLFDFTQNATLNFINTLINFQS-LYAPIHGYVCIGLCVFGILTNFVHVVVLTRPVMRNSAVNCVLTAVAICDIGTMATYLIYISHFVVLR
       C. elegans DMSR-7    ---MECPHDAQLFDPNSNSTQAFLRKLAHFQR-WYQPIHGYVCVLICVFGIFTNFVHVAVLSRPNMRNSAVNCILTAVAVCDIGTMASYLIYIIHFVLRR
         S. ratti DMSR-7    MARKLCPNDVSLFSGNDTFTQLSLHYLDLFYN-FYAKPHGYICLVICIFGLVTNFVHVIVLTRKNLRKSAVNCIMTAVAFCDMGTMFTYLIYNLHYVLGF
    B. xylophilus DMSR-7    ----------------SSQTCKILHFIPSYFS-FYAPLHGYICVFLCLFGFSSNFIHVIVLTRQSMRGSAVNSIMTAVALCDMGTMASYLIYICHFVLK-
       G. pallida DMSR-7    --MHCAN-DPALFDFDSNSTRAFIQSLVLFQK-AYAPIHGRICVFLCLFGVVTNLIHCVVLTRPQMRVSAVNVIMTAVALCDLGTMGSYLIYIVHFVFQN
         M. hapla DMSR-7    --MECGPDEPTLFNFENNSTRQFIHSLVLFQR-AYAPIHGRICVFLCLFGVITNLIHCVVLTRPQMRQSAVNSIMTAVALCDLGTMGSYLIYIWHFVKK-
     M. incognita DMSR-7    --MECGPNEATLFNFENNSTRQFIHSLVLFQKRAYAPIHGRICVFLCIFGVITNLIHCVVLTRPQMRQSAVNSIMTAVALCDLGTMGSYLIYIWHFVK–

            Consensus           C ND  LFD   NAT  FL  L  FQR  YAPVHGFICIVICLFGIVTNLVHVLVLTRPNMR SAVNCILTAVALCDMGTMGSYLIYI HFVL

     T. spiralis DMSR-7    Q----CSHFIYTYSWTWFLLLHALFSVVLHTISLWLAALFALVRLIVIRRTQDVSLFLTTRFIGKLFCYLAISVAILSIPHALTFTVVQETVPCKEH---
        T. muris DMSR-7    ED--RCQQTMHTYGWTWFLLLHALVSVVLHTISLWLAALFSLVRLIVIRRKQDVSLFLTTAFITRLFMYLAISVAILSIPHALTFRVVPEVYLCKDS---
       B. malayi DMSR-7    D---TT---IYSYLWMRYLLCHMVLSITLHTTSLWLIVAMAFIRQMTLRNAILNSNWQKPQMAWRVCTLIYFCVFILCIPTFLMYDVVEV--GDWHP-AP
    W. bancrofti DMSR-7    D---TTCVLIYSYLWMRYLLCHMVLSITLHTTSLWLIVAMAFIRQMTLRSAILNSNWQKPQMAWRVCILIYFCVFILCIPTFLVYDVTEV--GDWHP-AP
      D. immitis DMSR-7    N---CS-MPIYSYLWMRYLLYHMVASIALHTTSLWLIVAMAFIRQMTLRNTVLNSSWQKPKMAWKICTVIYLTVFLLCIPTLLMYDVVEF--GDWRP-AS
     O. volvulus DMSR-7    ----------------------------------------------------------------------------------------------------
      O. ochengi DMSR-7    ----------------------------------------------------------------------------------------------------
          L. loa DMSR-7    D---TTCMPIYSYLWMRYLSCHMVLSITLHTTSLWLIVAMAFIRQMTLRNAILNSNWQKPQMAWKVCLLIYFCVFLLCIPTLLVYDVVEI--GDWHP-AP
         A. suum DMSR-7    A---SSCSPTYSHLWMQFLLWHVVLSIALHTTSLWLAVAMAFIRRMTLKVARLNSEWQRPQFAWRVCMGVYVAVFVLCIPTLLVHDIIEYTQAEWHP-PP
      A. caninum DMSR-7    -----------------------------------------------------------PKFAWKLCILIYVCVFIMCIPSVFVHEISLYPEGVWQP-TP
 N. brasiliensis DMSR-7    Q---STCSPTFTHGWMQFLQWHVMISITLHTTSLWLAVAMAFIRRMTLRVARLNSTWQRPQFAWKLCIVIFICVTLMCTPNMFVHEIAVYEGAVWEP-ST
     H. contortus DMSR-7    Q---STWSPTFTHGWMQFLQWHVMLSITLHTTSLWLAVAMAFIRRMTLRVARLNSTWQRPQFAWKLCIAIYIIVAVLCVPSLFVHEIAVYEGIVWEPPHP
       C. elegans DMSR-7    N---NSCTPTFTHSWLQFLLWHVVLSITLHTTSLWLAVAMAFIRRMTLRVTALNSQWQRPKFAWKLCLGIYMVVFVLCIPNMLVHEIARVEGNDWRP--G
         S. ratti DMSR-7    SFSTKSCSNKNSYNWNIFLYIHVIGSILLHSTTLWLAVAMAFLRRITLQTNSFNSSWQKAKIARKICIVIFIFTFISIIPTILVHNIVKLNKWYPKDEGK
    B. xylophilus DMSR-7    --------GKSTYLSMSFLLFHMFLSITLHTTTLWLAVVMAFLRRMTLHTTTLHSQWQRSRLATKISLIVFFVVMLFSLPTLLVHRIVEYEADVWRP-KP
       G. pallida DMSR-7    TP--NSCSNPQTYWWMLFLILHIFFSILLHTATLWLAVVMALLRRMTLHRNTLYSHWQRAPLARFISMGVLGTVFLLSVPSLLVHEIIELPHNRWTP-SA
         M. hapla DMSR-7    ------SSNTQTHIWMYYLLLHIFISILLHTATLWLAVLMAFLRRMTLHRNTLYSKWQRVPLARRASLSVIFFILFLCIPSLAVHQVIEYPHANWQP-SL
     M. incognita DMSR-7    ------SSNTQTHIWMYYLLLHIFLSIVLHTATLWLAVLMAFLRRMTLHRNTLYSKWQRVALARRASLTVIFIILLLCIPSLAVHQVIKYPHANWQP-SL
```

```
          Consensus        S    YTY WM FLL HMVLSI LHTTSLWLAVAMAFIRRMTLR   LNS WQRP LAWKLCI IYI VFILCIPTLLVHDIV    G W P

   T. spiralis DMSR-7   -----------SENASIRYTLMPS-QLAIAGDCFLLKTTFWINGFPFKLVPCVILFVSILSLVKFLSEAQAKKQRIFGNNKRQQ---------------
      T. muris DMSR-7   -----------LHNTTLRYTLMPS-QLAIANHCFLLKMTFWINGFPYKLIPCVILFISILSLLKFLSEAQAKKRRTFGNNRRQ----------------
     B. malayi DMSR-7   HCSHSFP----PNYTAKYYTFHVS-PSAMANGCRFFKWNLWMSGIIFKVVPCILLLYFSSSLILTLHQTTRKRKLILKYNSTK----------------
  W. bancrofti DMSR-7   QCSHGFP----PNYTAKYYTFHLS-PSAMANGCRFFKWNLWMSGIIFKVVPCILLLYFSSSLMLTLHQTTKKRKLILKYNSTK----------------
    D. immitis DMSR-7   HCAHHFP----TNYTAKDYTFHIS-SSAMANGCRFFKWNLWMSGVIFKVIPCTLLFYFSSALILKLNRVTKKRKLLLKCNVMT----------------
   O. volvulus DMSR-7   ---------------------------------------------------------FSSRLIIKLHRTTKKRKLLLKCRAT-----------------
    O. ochengi DMSR-7   ---------------------------------------------------------FSSRLIIKLHRTTKKRKLLLKCRAT-----------------
        L. loa DMSR-7   HCAHDFP----PNYTAKYYTFHIS-SSAMENGCRFFKWNLWMSGIIFKVIPCILLLCFSSSLMLTLHRTTKKRMHLLKCNSTK----------------
       A. suum DMSR-7   DCEQYYP----ANYTAKLYTLHIP-ESATANGCRFFKLNLWMSGVLFKVIPCVLLFFLSIGLMMKLHNASRKRRALLKKNFDS----------------
    A. caninum DMSR-7   RCSQFYP----ANYSQPIYTFTVS-KEATANNCSLFKLNIWMIGIVFKVI-------------------------------------------------
N. brasiliensis DMSR-7  QCAMHFP----PNYTQLIYTFTQVSKEATANNCSIFKLNIWMIGLVFKVIPCILLMFFSVGLVNKIRQAENNRRKLTNVSLNNLNSTDAGAMS------
   H. contortus DMSR-7   HCALSYP----PNYTEPIYTFTQVSKAATANNCRIFKLNIWMIGLVFKVIPCILLMYFSVGLVNKIRQAEKHRRKLTNVSLNNVNSCDSSSMS------
    C. elegans DMSR-7   KTCQKFA----SNYSEPVYTFMVS-HAATINNCRLFKWNIWMIGILFKIIPCILLIFLSFGLVSKIRDAEKHRRKLTSVPSN----ASTDSKP------
      S. ratti DMSR-7   KCLNDYGG--NENISFQRYTIDFP-EFVLKDNCRIFKGNLWLSGILNKVIPSLLLFFLSFSLMSKLKIAQEKRRKLLENGHKLSHDN------------
  B. xylophilus DMSR-7  SCMEFYP----ANYTETIYTLAVS-PTAMHNGCRWFKANLWLTGTLFKVIPCILLIILISSLLLKLKNAEQKRRLLLLNGGVGNDTN------------
     G. pallida DMSR-7   QCSDIYP----ANYSERLFTVIIR-QSALANGCVLFKTNLWLTGLFFKVIPCVLLLTLSCSLMQKLRMAERKQGDLLGNVRRNSAVPCGSSGG-----S
      M. hapla DMSR-7   LCPKVISGILSKNHSEPIFTIIIR-NIAMANGCWLFKANLWLTGIIFKVIPCLLLLILSASLLQRLRQAEKKRCQLLKQLGDSPNGRNSLDNLKQRQTN
  M. incognita DMSR-7   LCPKIISGILSKNHSEPIFTIIIR-NTAMANGCWLFKANLWLTGICFKVIPNLLLLILSASLLQRLKQAEKKRCQLLKQLSDSP---------------

          Consensus      C   FP     NYT   IYT   IS    AMANGCRLFK NLWMSGIIFKVIPCILLL  SSSLM KL  A KKRR LL  N

   T. spiralis DMSR-7   ---------PVRYISRSTTKLLVTILSIFLITEIPQAIVATLSGIFPAEIYAKIYPKVGDLLDLLALINCNANFILLCIMSSQFRTTFASMFLPRRFQC-
      T. muris DMSR-7   ---------PTRYISRSTTKLLVTVLSIFLITEIPQAIVATMSGIYPSDIYAKIYPKLGDLLDLLSLINSNANFILLCIMSSQFRTTFSMMFLPRKF---
     B. malayi DMSR-7   -------IRRGVTKSDRTSALLLAIVLVFLIAEMPQGIIAIMNAIYTTHVHIYIYFNLGDILDLLSLLNSSITFVLYCLMSSRYRDTFWTVVLPKHFFRE
  W. bancrofti DMSR-7   -------IR-GATKSDRTSALLLAIVLVFLIAEMPQGIIAIMNAIYTTHVHVYIYFNLGDILDLLSLLNSSITFVLYCLMSSRYRDTFWTVVLPKHFF--
    D. immitis DMSR-7   -------TKRSVIKSDRTSHLLLAIVLVFLIAEMPQGIIAIMNAVYTTHVHQHIYFNLGDILDLLSLLNSSITFVLYCLMS-SYRETFWTVALPKCVFFS
   O. volvulus DMSR-7   -------------------------------KEMPQGIIAIMNAVYTTHVHEYIYFNFGDILDLLSLLNLSIIFILYCLMFSRYRKTFWTVMLPKYLSSE
    O. ochengi DMSR-7   -------------------------------KEMPQGIIAIMNAVYTTHVHEYIYFNFGDILDLLSLLNLSIIFILYC----RYRKTFWTVMLPKYLSS-
        L. loa DMSR-7   -------TRRDVIKSDRTSVLLLAIVLVFLIAEMPQGIIAIMNAIYTTHVHIYIYFNLGDILDLLSLLNSSITFVLYCLMSSRYRDTFWTVVLPKHLFSE
       A. suum DMSR-7   -------TKKHTNVSDRTTAMLICVLIVFLITELPQGILAILNAIYTTHVHMYIYFHLGDILDLLSLLNSSINFVLYCVMSSRYRNTFWTVVLPPHVYRY
    A. caninum DMSR-7   ----------------------------------------------------------------------------------------------------
N. brasiliensis DMSR-7  -------AKKKSHKSDRTTAMLVVILAVFLITELPQGVISILCAIFTADVHKYLYFHLGDILDLLSLLNSSVNFVLYCLMSSRYRQTFWLVFLPAGAHPL
   H. contortus DMSR-7   -------AKK-SHRSDRTTTMLVVILAVFLITELPQGIISILCAIFTTDVHKYLYFYLGDILDLLSLLNSSVNFVLYCVMSSRYRQTFWLVFLPSG--AL
    C. elegans DMSR-7   -------LKKKNGTSDRTTLMLVVILLVFLITEFPQGIISILCAIFTTDVHRYLYFYIGDVLDLLSLVNSSVNFVLYCVMSSRYRQTFWEVIIPSWACGL
      S. ratti DMSR-7   --------TMKRKYTDKTTPMLLAILIVFLMTEVPQGVVMIITAIYTNDGFNVLYKNLADILDLLSLINSSVNFIMYCVMSSRYRATFLQVVLNRKFQNI
  B. xylophilus DMSR-7  ----------KRITSDKTTVMLLAILAVFLFTEFPQGILSILSAVYTNDVHNMIYFMLGDVLDLLSLVNSSVNFVLYCLMSSR----------------
     G. pallida DMSR-7   PAALSGTSKLKKINSDRTTAILLAILTVFLLTELPQGLIAILNAIYTADVHRFIYLTLGDLLDLLSLVNSSCNFVLYCLMSSRYRQTFCSLLIPIAGCKR
      M. hapla DMSR-7   IGLKKFLKNNKRMNADRTTGLLLAILCVFLLTELPQGIIAILNAIYTADVHRFIYLTLGDVLDLLSLINSSVNFVLYCLMSSRYRQTFCSLFLPIQIC--
  M. incognita DMSR-7   -----------RMHADRSSGLLLAILCVFLLTELPQGLIAILNAIYTADVHRFIYLTLGDVLDLLSLINSSVNFVLYCLMSSRYRQTFCSLFLPIKIC—
```

```
              Consensus         K     SDRTT LLLAIL VFLITELPQGIIAILNAIYTTDVH YIYF LGDILDLLSLLNSSINFVLYCLMSSRYR TFWTVVLPK


      T. spiralis DMSR-7     ----------------------------------------------------------------------------------------------------
         T. muris DMSR-7     ----------------------------------------------------------------------------------------------------
        B. malayi DMSR-7     FTRKKTSEMLSS------------------EMHSTQKQINK-----------------------------------------------------------
     W. bancrofti DMSR-7     Q--KKTSEMLSS------------------EMHFTQKQIKQTIPCNNNCETRILK---------------------------------------------
       D. immitis DMSR-7     R--KKTSEILLS------------------EMHSMQKGMKYTVASSSNYSG-------------------------------------------------
      O. volvulus DMSR-7     V--KHTVASSSN------------------YNRTILK---------------------------------------------------------------
       O. ochengi DMSR-7     ----------------------------------------------------------------------------------------------------
           L. loa DMSR-7     FIRRKTSRMLPS------------------EMRFMQKQVKRTVSSSSNYEAKILK---------------------------------------------
          A. suum DMSR-7     FCIKTTSLEMISSMPAGTLKPQNRATKILINNNTTRKNSRKSGCNHDNYRAITEESDDYSMPLFPEAHSDHL----------------------------
       A. caninum DMSR-7      ----------------------------------------------------------------------------------------------------
  N. brasiliensis DMSR-7     CVNKQTTTVSNFNFTTIPIQRKNSNKVRSKEYAPLATEAEEQRDD-------------------------------------------------------
     H. contortus DMSR-7     CINKQTATASNLNFTQIQLQRKNSNKVRNKEYAPLATEAEEHRDE-------------------------------------------------------
       C. elegans DMSR-7     WTTRRGSPTELSQLQLNNSLRLG----RKQSYTPLATEPEPARNEYWRYEASVSDNQNSSREHQL-----------------------------------
         S. ratti DMSR-7     IINKNGITTLFFTKEFIPSTSNGNQLLKMKSTSLQNNINEELNFLNKKKDIINNKNDKQSVSIKKIGTSQYRGMKKLKNSIGEKNCFVYEDKNQDITNVC
     B. xylophilus DMSR-7    ----------------------------------------------------------------------------------------------------
        G. pallida DMSR-7    CVATAHQSLPMVPSFASQADRDNNRIARTVDTGRRR----------------------------------------------------------------
         M. hapla DMSR-7     ----------------------------------------------------------------------------------------------------
     M. incognita DMSR-7     ----------------------------------------------------------------------------------------------------

              Consensus       K

      T. spiralis DMSR-7     ------------------------
         T. muris DMSR-7     ------------------------
        B. malayi DMSR-7     ------------------------
     W. bancrofti DMSR-7     ------------------------
       D. immitis DMSR-7     ------------------------
      O. volvulus DMSR-7     ------------------------
       O. ochengi DMSR-7     ------------------------
           L. loa DMSR-7     ------------------------
          A. suum DMSR-7     ------------------------
       A. caninum DMSR-7     ------------------------
  N. brasiliensis DMSR-7     ------------------------
     H. contortus DMSR-7     ------------------------
       C. elegans DMSR-7     ------------------------
         S. ratti DMSR-7     DTNSIFEDNKEEYSIAKIEGDIQI
    B. xylophilus DMSR-7     ------------------------
       G. pallida DMSR-7     ------------------------
         M. hapla DMSR-7     ------------------------
     M. incognita DMSR-7     ------------------------
```

Consensus

20. DMSR-8 homologues

```
T. muris DMSR-8            ----------------------VDHFQG-AYQRIHPYLSSFVCSLGIVLNLVNIVILTRSRMKLCPTNVILTAISICDLCVMTTYLMFTQAEITGFHA--
T. spiralis DMSR-8         -------------------CNRVDFFSQSGYNRVHPYLSSFVCSLGMLFNLVNIVILTRTRMKLCPTNVILTAISICDFCVMTTYLMFSLSQITNFHQ--
B. malayi DMSR-8           -MNGGACEIGSVPFQNATWLFATLSNFHVTYGVIHPYIAVILCFAGTILNVMTIAVLTRP-SMISPVNVLLCSVAICDVLVMASYLIFVIHFLINAANRC
W. bancrofti DMSR-8        -MNGGACEIGSVPFQNATWLFATLSNFHVTYGVIHPYIAVILCFAGTILNVMTIAVLTRP-SMISPVNVLLCSVAICDVLVMASYLIFVTHFLINAANRC
D. immitis DMSR-8          -MNGGACEIGSVPFQNATWLFAALADFHVTYGLIHPYIAVILCFAGTILNVMTIAVLTRP-SMISPVNVLLCSVAICDVLVMASYLIFVTHFLISAANRC
O. volvulus DMSR-8         MNGGGACEIGSVPFQNATWLFATLSNFHVTYGLIHPYIAVILCFAGTILNVMTVAVLTRP-SMISPVNVLLCSVAICDILVMASYLIFVTHFLINAANRC
O. ochengi DMSR-8          --GGGACEIGSVPFQNATWLFATLSNFHVTYGLIHPYIAVILCFAGTILNVMTVAVLTRP-SMISPVNVLLCSVAICDILVMASYLIFVTHFLINAANRC
L. loa DMSR-8              -MNGGACEIGSVPFQNATWLFATLSHFHVTYGFIHPYIAVILCFAGTILNVMTIAVLTRP-SMISPVNVLLCSVAVCDVLVMASYLVFVTHFLINAANRC
A. suum DMSR-8             --------IGSVPLPNITWLISAIAQFDSSYGAVHPYIALFLCVSGTIMNLVTVIVLTRP-SMVSPVNVLLCSVAVCDVIVMASYLIFVSHFLIGAANRC
A. caninum DMSR-8          --------MGALLVRNAEWLADAVAVLHGAYAHIHPYMAVALCLAGTAMNAVTVVVLTRP-SMLSPINAILCAVAICDILVMTSVLVFVYHFLISASFRC
N. brasiliensis DMSR-8     ------------------------------------------------------------------------------------------------FRC
H. contortus DMSR-8        --------MGAIPLTDAEWLSDAVSTLHSAYAHLHPYMAVVLCLAGTVMNAVTVVVLTRP-SMLSPINAILCAVAICDILVMTSVLVFVYHFLISANFRC
C. elegans DMSR-8          --------MGSVNFTGAEWLETAVTNINTAYAHIHPYVSVILCLAGTAMNIVTVIVLTRP-SMRSAVNSLLCAIALCDILVMTSVLVFVTHFLLFAGYRC
S. ratti DMSR-8            -MSFDTCTSSSNVVFVENNFIQVWKYYLYLYSEYHSNVALGICIIGILLNIVTVAVLSRP-IMRNSINIILCFIAICDIIVMLSYAIFNFHYYVTAGMRC
B. xylophilus DMSR-8       --------IGSILLPNITWLTDGVNAFGQSYVEIHQYVATFLCIVGTLMNLTTVVVLTRP-SMISPVNVLLCAVAICDILVMTSYLVFIVHFHLIAPARC
G. pallida DMSR-8          --------IGSLEMPPSEWLFSGAASFQQSYGQVHPYLAAVLCVVGTLMNAVTVLVLSRP-SMRSPVNVLLSAVAVCDIVVMSSYLVFVVHFLLTAASRC
M. hapla DMSR-8            --------IGTLEMPPLEWLFTAVAAFQQNYGQIHPYLAVGLCIAGTIMNFVTVLVLTRP-SMRSPVNVLLCAVACCDILVEVSYLIFVLHFLLASAYRC
M. incognita DMSR-8        --------IGTLEMPPLEGLFTAVAAFQQNYGQIHPYLTVGLCIAGNIMNLVTVLVLTRP-SMRSPVNVLLCAVACCDILVEVSYLIFVLHFLLASADRC

Consensus                          IGSV   NA WLF ALA F  TYG IHPYIAVILCIAGTILNVVTVVVLTRP SMISPVNVLLCAVAICDILVM SYLIFV HFLI AA RC


T. muris DMSR-8            STPTGMFYWAVFVLIHSNASVVLHAG----------------------------------------------------------------------------
T. spiralis DMSR-8         SSSSCMFYWAIFVLFHSNASVVLHATSIWLT-VLLASIRVYSVRRALRRSLW-HCSPRSAARYSVAIFLIALLLDIPTMLSFGIEQRWATENATVANSTE
B. malayi DMSR-8           LHTDYSYPWTIFTLIHAHASVILHSTSIWLT-VLLAQIRVLTIRRSTLHPST-TVTIRFTVLLSLATCSIMSLFNLPNFLTFKIVRTSPELFLPCLVKFS
W. bancrofti DMSR-8        LHTDYSYPWTIFTLIHAHASVILHSTSIWLT-VLLAQIRVLTIRRSTLHPST-TVTIRFTVLLSLATCFIMSLFNLPNFLTFKIVRTSPELFLPCLVKFS
D. immitis DMSR-8          LHTDYSYLWTIFTLIHAHASVILHSTSIWLT-VLLAQIRVLTIRRSTLHPST-TVTIRFTVLLSIATCLIMSLFNLPNFLTFKIVRTSPELFLPCLVKYS
O. volvulus DMSR-8         LHTDYSYPWTIFTLIHAHASVILHSTSIWLT-VLLAQIRVLTIRRSTLHPST-TVTIRFTVLLSIATCIIMSLFNLPNFLTFKVVRTSPELFLPCLVKYS
O. ochengi DMSR-8          LHTDYSYPWTIFTLIHAHASVILHSTSIWLTVVLLAQIRVLTIRRSTLHPST-TVTIRFTVLLSIATCIIMSLFNLPNFLTFKVVRTSPELFLPCLVKYS
L. loa DMSR-8              LHTDYNYPWTIFTLIHAHASVILHSTSIWLT-VLLAQIRVLTIRRSTFHPST-TVTIRFTVLLSLATCFIMSLFNLPNFLTFKIVKTSPELFLPCLIKYS
A. suum DMSR-8             DYNDYSFAWAVFTLFHAHASVIFHSTSIWLT-VSLAQIRVLTISRATSGPTT-TITTRFTVALSFITCLIMASVNIPNFLTFEIAEMAASTFLS------
A. caninum DMSR-8          DPDDYDRMWAYFLFAHSQSTVILHATSIWLT--VLLAK----------------------------------------------------------------
N. brasiliensis DMSR-8     DPSDYDQIWAYFVFAHSQSTVIFHATSIWLT--VVQVA----------------------------------------------------------------
H. contortus DMSR-8        DPEDYDKKWAYFLFAHSQSTVILHATSIWLT--LLAQIRVYTIKRATAGPSE-AISARSTGGIALITLCVVALVNIPNFMTFEIVEIDAELWLPCL----
C. elegans DMSR-8          DPTDYNIYWAYFLYYHSQATVIFHATSIWLT-VLLAQIRVFSIRRATSVAGE-SVTNQMTCIIAVTTFIVVCLLNVPNMLTFEIIETPASLWLQCKANET
S. ratti DMSR-8            SISDWSYGWALFMMFHANSSVIVHSTSLWLT-VSLAQIRLLTIRKATVGPTG-MFSERATVYLSIATFFTMFIVNIPNILSMEIFSTDSIQFFKPFGCLT
B. xylophilus DMSR-8       QLQDYSYGWGIFTMIHAHVSVICHATSIWLTVVSLAQIRVLTIRRATAGPTT-QITERFSIILAIVTLIIMAAVNLPNFMTFEVL---------------
G. pallida DMSR-8          EPSDFSYAWALFMIFHAHASVIFHAASIWMT-VSLAQIRVLTIRRATAGPSTPLITERFTVLLAVLMLCAMTLVNLPNFLTFEIMEVSSNSMLPCL----
M. hapla DMSR-8            EPSDFSWWWAVFMMFHAHASVIFHAASIWML-VVMAQLRVLTIRRATKGPTTPLTTERFTALIALLTTLVS-----------------LN-----------
M. incognita DMSR-8        EPSDFSWGWAVFMMFHAHASVIFHAASIWML-VVMAQLRVLTIRRATKGPTTPLITERFTALIALLTTIVMTIVNIPNFLTFEVFNIKNN----------
```

```
            Consensus     TDYSY WAIFML HAHASVILHATSIWLT VLLAQIRVLTIRRAT  PST  VT RFTVLLSL T IIMSL NLPNFLTF IV      FL C

          T. muris DMSR-8  ------------------------------------------------------------ILFKVVPCILQSILIGMLLYLLKQARFRRKRLSSGALNRS
       T. spiralis DMSR-8  MSSFY--------------VTSI-----------------STFAQKNDRLLLKFAFWCNGVSFKVVPCIFQSLLIGMLLHLLRKARFRRKRMSSGAIHRS
         B. malayi DMSR-8  ENSSL-----HDKVDDDYTEQNS-----DYSQYNDEYPVYMLVASQGNCLKLKLAFWSNGILFKVVPCLLLTISIIVLLKVIADLSRQRR-HLAQLMKR-
      W. bancrofti DMSR-8  ENSSL-----YDKVDDDYTEQNS-----DYSQYNDEYPVYMLVASQGNCLKLKLAFWSNGILFKVVPCLLLTISIIVLLKVIADLSRQRR-HLAQLMKR-
        D. immitis DMSR-8  KNSSLLLSIDNSKLDDEYVQQNSDYSQYDDDNDDNEYPVYMLVASQGNCMKLKLAFWSNGILFKVVPCLLLTISIIVLVKVIADLSNQRR-TLAQLMKR-
       O. volvulus DMSR-8  KNSSLSLSIDNDKFDDDYSQQN---LEYVDDSDD-EYPVYMLVASQGNCMKLKLAFWSNGILFKVVPCFLLTVSIIVLVKVIADLSNQRR-TLAQLMKR-
        O. ochengi DMSR-8  KNSSLSLSIDNDKFDDDYSQQN---LEYVDDSDD-EYPVYMLVASQGNCMKL--AFWSNGILFKVVPCFLLTVSIIVLVKVIADLSNQRR-TLAQLMKR-
            L. loa DMSR-8  ENSSS-----YSKIDDDYSEQNP-----DYSQHDDEYPVYMLVASQGNCVKLKLAFWSNGILFKVVPCLLLTVSIIILLKVIADLSHQRR-TLAQLMKR-
           A. suum DMSR-8  --------------------------------------------------CLKLAFWSNGMIFKVVPCFLLTISIAALLKIIADVSHRRR-SLAQVMKKK
        A. caninum DMSR-8  -----------------------------------------------------VAFWENGILFKVPFVALLTVSIIALLKIIKDVSVRRK-SLAQVMNKK
   N. brasiliensis DMSR-8  -----------------------------------------------------FWTNG--ILFKVVPCTLLTISIIALLKIIRDVSHRRK-SLAQVMNKK
       H. contortus DMSR-8  --------------------------------------------TSAT--QEEVAFWTNGIFFKVVPCTLLTVSIIALLKIIRDVSHRRK-SLAQVMNKK
        C. elegans DMSR-8  -AE--------------------------------DDMLVYLVAPSDHCGLLNIAFWTNGVLFKVVPCLLLTFSIVALVSIIRDVGKRRK-QLAQVMNKK
          S. ratti DMSR-8  ITNYDDKVIYNFP-----SSENETIENIDKIESNIKYLAYSYVR-KNNCIMIKISYWINGAIYKIVPCVLLTISIVALLKMIKDVKKRRL-KLAECTKR-
     B. xylophilus DMSR-8  ---------------------------------------------------FQMAFWSNGMIFKVVPCLLLTVSIVALLKIIADVSNKRK-NLAQVMKK-
        G. pallida DMSR-8  ------------------------------QSSDPADMDYTVRAHDSDCMKLKLAFWSNGMIFKVIPCLLLTLSIWALLRIIADVAHKRR-NLAQVMRK-
          M. hapla DMSR-8  -----------------------------------EPIVYTVRAHESDCMNLKLAFWTNGILFKVIPCILLTLSIAALLRIIADVAHKRK-NLAQVMRK-
      M. incognita DMSR-8  -----------------------------------EPIVYTVRPHESDCMNLKLAFWTNGILFKVIPCILLTFSIAALLRIIADVAHKRK-NLAQVMRK-

            Consensus                                        E  VY L A    CM LKLAFWSNGILFKVVPCLLLTISIIALLKIIADVS RRK  LAQVMKK

          T. muris DMSR-8  LRDYDKSTPMLIALLAAFLIAELPQGLLLMGTALFKDFRTKV---YLKSGPFMDLLSLLN----------------------------------------
       T. spiralis DMSR-8  LRDYDRSTPMLIVLLAAFLIAELPQGLLLVGTAIFKDFRTKI---YLKSGAFMDLLSLMNSAVTFVIYTSMSAAFRRLFFAIFIPFFRTSSPYEPT----
         B. malayi DMSR-8  KVPKDHTTPMLVAVLSIFLITELPQGVMNVLTGIFTGDTFHQ-KVYLPLGDFMDLLSLINSAVNFLIYCIMNKRFRVTFLQLFCGFFTNRKLSRVVEDYS
      W. bancrofti DMSR-8  KVPKDHTTPMLVAVLSIFLITELPQGVMNVLTGIFTGDTFHQ-KVYLPLGDFMDLLSLINSAVNFLIYCIMNKRFRVTFLQLFCGFFINRKLSRVVEDYS
        D. immitis DMSR-8  KVPKDHTTPMLVAVLSIFLITELPQGIMNVLTGIFTGDTFHQ-KVYLPLGDFMDLLSLINSAVNFLIYCIMNKRFRITFLQLFCDFCTNQKLS-IAEDYS
       O. volvulus DMSR-8  KVPKDHTTPMLVAVLSIFLITELPQGVMNVLTGIFTGDTFHQ-KVYLPLGDFMDLLSLINSAVNFLIYCIMNKRFRITFLQLFCDFCPNRKLSRIVEEYS
        O. ochengi DMSR-8  KVPKDHTTPMLVAVLSIFLITELPQGVMNVLTGIFTGDTFHQ-KVYLPLGDFMDLLSLINSAVNFLIYCIMNKRFRITFLQLFCDFCPNRKLSRIVEDYS
            L. loa DMSR-8  KVPKDHTTPMLVAVLSIFLITELPQGVMNVLTGIFTGDTFHQ-KVYLPLGDFMDLLSLINSAVNFLIYCIMNKRFRITFLQLFCDFCANRKFSRVVEDYS
           A. suum DMSR-8  KMPKDHTTPMLVAVLSIFLIAELPQGVMSVLTGIYSRETFHQ-KIYLPLGNLMDLLSLINSAVNFLIYCAMSRKFRIVFLQLFIKSLPSAFFR-------
        A. caninum DMSR-8  RMPRDHTTPMLVAVLSIFLIAELPQGMLHVCNAIYSNETFYQ-KIYLPLGDLMDLLSLLNSAVNFLIYCAMSRKFRTVFIQTFLACLPDPLYRKFLQDWP
   N. brasiliensis DMSR-8  RMPRDHTTPMLVAVLSIFLIAELPQGMLHVCNAIYSNETFYQ-KV-------------------------------------------------------
       H. contortus DMSR-8  RMPRDHTTPMLVAVLSIFLVAELPQGMLHVCNAIYSNETFYQ-KIYIPLGDLMDLLSLLNSAVNFLIYCAMSRKFRTVFMQTFLSCLPDPLYRKFLKDWP
        C. elegans DMSR-8  RMPRDHTTPMLVAVLSIFLFAELPQGVLHVFNAIFTKETFYD-KIYIHLGDVMDVLSLLNSAVNFIIYCAMSRKFRAVFIQIFLTCLPQKIIRKYAMEAF
          S. ratti DMSR-8  KIPKDHTTPMLACVLIIFLITELPQGILHLLSGIYTHESFQD-HIIKPFGDFSDLLSLTNSATSFIIYCTMSRKFRAVFFAFFCDWLPKPIKKHIRETDR
     B. xylophilus DMSR-8  KVPKDHTTPMLVAVLSIFLIAELPQGVMLVMTGIFSSDTFKKTVIYQPLGDFMDFLSLLNSAIGFFIYVGMSRKFRSVFFQLFFSLIHCTPYR-------
        G. pallida DMSR-8  KVPKDHTTPMLAAVLLIFLLAELPQGLFLVLNGIFSAESFHK-RVYLPLGDLMDLLSLINSAVGFFIYVGMSRKFRTVFLQLLFS---------------
          M. hapla DMSR-8  KVPKDHTTPMLAAILTIFLLAELPQGLLLVLNGIFSSESFHK-RVYLPLGDLMDLLSLLNSAVGFLIYVGMSRKFRTVFLQILFSFL-------------
      M. incognita DMSR-8  KVPKDHTTPMLAAILTIFLLAELPQGLLHVLNGIFSSESFHK-R--------------VNSAVGFLIYVGMSRKFRTVFLQILFSVL-------------
```

```
                 Consensus     KVPKDHTTPMLVAVLSIFLIAELPQGVL VLTGIFS ETFHQ KVYLPLGDFMDLLSLINSAVNFLIYC MSRKFR VFLQLF  F           D
        T. muris DMSR-8        ----------------------------------------------------------------------------------------------------
     T. spiralis DMSR-8        ----------------------------------------------------------------------------------------------------
       B. malayi DMSR-8        RMDYAYESPGQRN--RSTDVSRTEQLIPTSSQHQPSATSVFTSLSVTREEPLQNYRKFTGNLLTVEGNHCRRMTIVTGQAIINDNLRCYIIMYTSTISHV
    W. bancrofti DMSR-8        RMDYAYESPGERN--RSTDVSRTEQLMPTSSQHQPSATSVFTSLSVTREEPLQNYRKFTGNLLTVEGNHCRRMAIVAGQEESKE----------------
      D. immitis DMSR-8        RVEYAYESQRQHN--RSIDISRTEQLM-LTSQHRPSAASVFTNSSSTRDEPLKNQKKFTGNLLTVDGNQYRRMTILAKQ---------------------
     O. volvulus DMSR-8        RVEYAYESSRQPN--RSADISRTEQLM-LTSQHRPSSVSVFTNLSSTREEQLKNQRKFTGNLLTVEDNHYRRMTILAKQ---------------------
      O. ochengi DMSR-8        RVEYAYESSRQPN--RSADISRTEQ---VTSQHRPSSVSVFTNLSSTREEQLKNQRKFTGNLLTVEDNHYRRMTILAKQ---------------------
          L. loa DMSR-8        RMDYAYELPGQRN--RSADISRTEQLMLTSSQHRPSATSVFTSLSVTREEPSKNHRKFTGNLLTVEGNQCRRMTTVTKQEESKE----------------
         A. suum DMSR-8        -------SAQR----LRRNYVNENASQLALTSHRPSATSVLVSIG--PRDDRLMYRKYGDNLT-------------------------------------
      A. caninum DMSR-8        ALAEMELSRHKPS--GFTDLTKTEQ--LALTSHRVSATSMLIHAAS--KE--STSKMYTGNLLSVDTPGPRVSFDARPGTDLMARNSLEIHEIRLVVPDT
 N. brasiliensis DMSR-8        ----------------------------------------------------------------------------------------------------
    H. contortus DMSR-8        ALAELEMSRHKPS--GLTDLTRSEQ--LALTSHRVSATSMLIHATA--KE--STSKMYTGNLLSMSK----IRHFWENGTNAIE--SSPQRRINLIKCET
      C. elegans DMSR-8        LDVEVSRMRPTAD------MTKSEQ--LALTSHRVSASSVLLPISRPDPPRMYSGNLLTADINGGYSTSPRVSFDIVRKESQISSVVDFQLVVPNMQQDS
        S. ratti DMSR-8        FETKTDATKIKSSRGNIIESSMYDYTQHRLSIASSFAGELFNTIRDDRKMSWLSRKISNIKVKKNDANNLESTIEHKKSNFLTVPSSYLPRRISLTIEVD
   B. xylophilus DMSR-8        ----------------------------------------------------------------------------------------------------
      G. pallida DMSR-8        ----------------------------------------------------------------------------------------------------
        M. hapla DMSR-8        ----------------------------------------------------------------------------------------------------
    M. incognita DMSR-8        ----------------------------------------------------------------------------------------------------

                 Consensus            S          DISRTEQ     S H  SA SV          E     R FTGNL
```

21. EGL-6 homologues

```
      A. suum EGL-6     -----------------------------FLQVYDQIHIPLSLTICVFGAISNVFNIIVLTRR--MRTPINVLLAGLSFSQW------------------
   C. elegans EGL-6     MENIIEHNDSFSAMNDTLICTYFECYIHMFAMYYDEIHIPLSISICIFGAASNVFNIIVLTRKR-MRTPINILLTGLSIAQWLLATNYFLYLLLEYYRYQ
   A. caninum EGL-6     ----------------------------------------------------------VYSRKT-MRNPINILLCGLSAAQWLLASN-------------
B. xylophilus EGL-6     ---------------------------------YDRIHIPLSTTICVFGSISNIVNILVLTRRKTMRTPINIFLTGLSMAQFRLLAL-------------

        Consensus                                    F   YD IHIPLSITICVFGAISNVFNIIVLTRKK MRTPINILLTGLSIAQWLLASNYFLYLLL


      A. suum EGL-6     ----------------------------------------------------------------------------------------------------
   C. elegans EGL-6     CVQLLWSEAFTRYRFFNVNLNTVFHTIAFTTTIVVAVFRYCALKFPIQANRFIYKCQPAIAANVIIWIIIPIISLPLFFISEVKIVARDHVAYDLQCEME
   A. caninum EGL-6     ----------------------------------------------------------------------------------------------------
B. xylophilus EGL-6     -K------------------------------------------WPLRANRFLFCNTWALACSLGIWIIVPFLCIPVFLTSYVALVD------DNRGCNF

        Consensus                                                   FPI ANRFIF    AIA  L IWIIIP I IPLF  S V IV       D


      A. suum EGL-6     --MYDLNYSQNAALVKFVFWMFGIVVKPHG----------------------------------------------------------------------
   C. elegans EGL-6     GPLYDLSYQESPLLVSAVFWAFGIVFKLLPSLILSILLIALIRSLKSVERRRKNWKRTQGANICTNSERKAKRKLTTRPRTTRMLVIILLLCVMVELPMG
   A. caninum EGL-6     ----------------------------------------------------------------------------------------------------
B. xylophilus EGL-6     KGMYDLTYSEDSFLVSAVFWMFGIVLKPHG----------------------------------------------------------------------

        Consensus         MYDLSYSE A LVSAVFWMFGIVLKPHG


      A. suum EGL-6     ALNLCTGIYGETFGIRIYDHLGSFMEMLTLLYSSISFVLYCSMSNDFLHTFQSLF-------------------------------------------
   C. elegans EGL-6     ILNLCVAIYGEEFGNRYYDPVGNLMEMLTLLYSSVSFVLYCTMSNEYLSTFRALFFPWTRKNSLRGTRRSWNHRHDDETKSPRTFLINRTAPSSYVGS
   A. caninum EGL-6     --------------------------------------------------------------------------------------------------
B. xylophilus EGL-6     ILNLFTAIYGDKFGVHIYDQLGSFMEMLTLLYSSISFILYCVTSNDFLRTFRQLFLNSKKR-------------------------------------

        Consensus       ILNLCTAIYGE FGIRIYD LGSFMEMLTLLYSSISFVLYCSMSNDFL TFRALF    KK
```

22. FRPR-3 homologues

```
      A. suum FRPR-3   ---------------------------------------------------STICVVVMIIMCMCGVVGNSLSLYVYWTPTFRKRSINLLLAALSASDLF
   A. caninum FRPR-3   ----------------------------------------------------------------------------------------------------
N. brasiliensis FRPR-3 ----------------------------------------------LLALLSQICGAVMIVLCLFGVVGNSLSLYIYTRPAFRKRSINILLSALSATDLS
  H. contortus FRPR-3  ----------------------------------------------------------------------------------------------------
    C. elegans FRPR-3  ----------------------------------------------MLLLLSRVLVNVMVLMCFGGILTNFISFYIYTRKTFRKKSINVLLAALSMSDLC
      S. ratti FRPR-3  -----------------------------------------------------VLSIICFVMCIIGVITNTFSIIIYTSVNFRKRSINILLTGMSASDLV
 B. xylophilus FRPR-3  MEGENSIINLTTIGVEDDVGTSEIIVVGGISLTESKCSLQEEDGLEFFSSLSLFFSCIMVCMCLAGAIMNTVSLFIFTRPSFRKRSINVLLSGLSASDLV
    G. pallida FRPR-3  ----------------MPPNASSPEAAEET--FLPSDHHVLLPSDQLLWALSLFFSLLMASLCVVGAVMNSISLFIYSRPAFRKRSINVLLCGLSASDLA
      M. hapla FRPR-3  ----------------MNSENTNNITGEQ-----QQFIVDNSIPKDLLPSLSMVFAVLMAFLCVAGTVTNTLSLIIFAKASSRRRSINVLLCGLSASDLC
  M. incognita FRPR-3  ----------------MNSDNSKNITEEQKSNEQQFDSIPLLDNQDFLPSLSLVFAVLMAFLCVAGTVTNTLSLIIFAKASSRRRSINVLLCGLSASDLS

           Consensus                                                 L  LS V SVLM  LCV G V NTLSLYIYTR SFRKRSINVLL GLSASDL

      A. suum FRPR-3   LCLLAMPVFSITQIQSFLPGLS--KHLTGHILLFAYPVTLMLQSISVWLLVSITIDRYLAVCYPFKVRAYCTRTRATITIILIVFFSIVYNFVRFWEFTL
   A. caninum FRPR-3   ----------------------------------------------------------------------------------------------------
N. brasiliensis FRPR-3 VCVLSIPVFS---SNHLHHLLPVSEKAIALLMFYAYPITVMFQSMSVWLLVSITVDRYLAVCHPFEVGSYSTRARALLTVFLIVVFSIAYNFVRFWEFQI
  H. contortus FRPR-3  ----------------------------------------MFQSMSVWLLVSITVDRYLAMCHPLXVGSYCTRSRALMTVLLIVVFSVAYNFVRFWEFQI
    C. elegans FRPR-3  VCVLAIPVFASTQLQQVIP-----PTITAMIMVYLYPVTIMFQSVSVWLLVSITIDRYLAVCHPFMVNTYCTRNRALITVGVVVIFSVAYNLIRIWEYTI
      S. ratti FRPR-3  VCLLAVPVFIGTELPTLFPKIELPPKILAYCIVYLYPITIMAGCMSVWMLVSITVDRYLAVCHPFMVRVYCTINRAKITVVVITIVTIAFNFVRFWEFDI
 B. xylophilus FRPR-3  LCILAIPVFCFSQLQQIIPGISF--TMLAQILVYAYPVTIMAQTMSVWMLVSITVDRYLAVCHPFLVRVYCTTTRALLTTAVIFVFSVGYNFVRFWEYTI
    G. pallida FRPR-3  LCVLALPVFSMSPLEYFMPGLP--PALTGHLLVFCYPVTLMAQTMSVWMLVGITVDRFIAVCYPFSVRIYCTVRRAQLTIGAILIFSVGYNLIRFWEYRI
      M. hapla FRPR-3  LCILALPVFSLAQLQQFIP-----PSLANHLLVFCYPLCLMAQTMSVWMLVGITIDRWLAVCYPFSIRIHCTVKRARLIVLGTFLFSLFYNLVRFWEYRI
  M. incognita FRPR-3  LCILALPVFSLAQLQHLIPGLP--PSLANHLLVFCYPLCLMAQTMSVWMLVGITIDRWLAVCYPFSIRIHCTVKRARLIVLSTFLFSLFYNLVRFWEYRI

           Consensus   LCILAIPVFS TQLQ LIP L   P L AHLLVF YPVTLMAQSMSVWMLVSITVDRYLAVCHPF VRIYCT  RA LTVLLIVIFSVAYNFVRFWEY I

      A. suum FRPR-3   DDSGG--TSSIEESIVPLLRGDPLFMLLYQNIATLITQFFLPLIVLCFLNLHV---ARTILEAGVQRR-----ELVASER--REYRTAKMMLYVVIV--F
   A. caninum FRPR-3   ----------LDSIVQPLLRDSPLFLLWYQNIATLLSQFVLPLLVLCVLNLQV---ARTILMAAEQRR-----ELVASEK--REHNTAKMMIFVVIV--F
N. brasiliensis FRPR-3 VESQS--EESLEAIVQPLLRDNQLFMLWYQNIATLLSQFVLPLLVLCVLNLQV---ARTILIAAEQRR-----ELVASEK--REHNTAKMMIFVVIV--F
  H. contortus FRPR-3  P-------ESLEAIVQPLLRDSALFMLWYQNIATLLSQFVLPLLVLCILNLQVSQVARTILMATEQRR-----ELVASEK--REHNTAKMMIFVVIITVF
    C. elegans FRPR-3  NFDVAPENRTIEDLVVPKLRANPHFLLWYQNVATLVSQFAFPLTVLCVLNIQV---ARTIIEASEQRR-----ELVASVK--REHSTAKMMIMVVLV--F
      S. ratti FRPR-3  AEP-S--EETGGFAFKKLLRADPIFMLVYQNIAYIISQYLLPLFVLCLLNLEV---AKTILKATEQRR-----ELVSSVK--TEHQTAKMMIFVVLV--F
 B. xylophilus FRPR-3  SDDPN---MPEEMLIVGLLRENQIYMVLYQNIAMLLTQFVLPLMVLCILNLQV---AKTILEAGETRR-----ELVASEK--REHSTAKMMLFVVIV--F
    G. pallida FRPR-3  NPSDVP-----SEPVVGLLREDYLYMLLYQNAATMLSQFLLPLCVLCALNFQV---ASAILAAVEQRREMWAEELSAAEQ--REHKTAKMMLMVVLV--F
      M. hapla FRPR-3  DSNGE---------IVGLLRDDYLFMLLYQNLATTLSQFLLPLCVLCPLNLQV---ARSILAARERRKNMLWTELSSVEASSREQSTANIFCFIFLV--F
  M. incognita FRPR-3  DSNGE---------IVGLLRDDYLFMLLYQNLATTLSQFLLPLCVLCPLNLQV---ARSILAARERRKNMLWTELSSVEASSREQSTAAMMLVVVLV--F

           Consensus            SLE IVVPLLRDD LFMLLYQNIATLLSQFLLPLLVLCVLNLQV   ARTIL A EQRR     ELVASEK  REH TAKMMIFVVIV  F
```

```
          A. suum FRPR-3     IFCYALSMILNLAEILDEDLFRQPIGYLLNDVNNILVVINSSSSFIFYTKYSTR--FRAQLR--------------------------------------
       A. caninum FRPR-3     LVCYTFSFILNVAEILFSSLFRHPVGYLLNDINNILIVVNTSSPFVFYVKYSTR--YRNQLRTMYGIRWFASKMKFVDSKPLRGSERLVPTPNGKALNSY
 N. brasiliensis FRPR-3     LVCYTFSFILNVAEILFSSLFRHPVGYLLNDINNILIVVNTSSPFMFYVKYSTR--YRNQLRTLYGIRWVAAWMKFD-----------------------
     H. contortus FRPR-3     LVCYTFSFILNVAEILFSALFRHPIGYLLNDINNILVVVNTSSPFVFYIKYSTRSRYRNQLRTIYGIRWAAARFKFYDRK--------------------
       C. elegans FRPR-3     LVCYIFSFILNIWEILDKETFGGDIGWFMNDINNVLIVVNSTSAIVFYYKYSTR--FRNQARTLPGIRWYASMSKFNVYDTEATSNRTMVTRYKESMISI
         S. ratti FRPR-3     IFCYTFGIFLNLLEHLYREIFDTTIGYMLNDINNILVVFNSSSNFIFYVKYSTR----------------------------------------------
    B. xylophilus FRPR-3     IFCYMLSFCLNVLEIFNPDLFKNHIGFLLNDVNNILVVVNSSSSFIFYVKYSSR--YRAQLRTMPVIAFFAARCCFENRNTNFSDKSGERSENYMTLTGY
       G. pallida FRPR-3     LFCYSLSFCLNVLEIVLPDLFRSQVGYLLNDVNNILIVLNSSSSFVFYAQFSSR--YRAQLMYLLRQFRCVDLLFRWIYGKKPVPYEGSPSGALSVGALL
         M. hapla FRPR-3     VFCYFLSLCLNVLEMFMPRLFRSPTGFLLNDVNNILVLINSASSALFYPSMNKD--CALE----------------------------------------
     M. incognita FRPR-3     VFCYFLSLCLNVLEMFIPRLFRSSTGFLLNDINNILVLINSASSALFYARFSSR–YRAHLISLLRNFKFLDCLLPPPKVSSFGGAETPSCVFSGIGLRQ

            Consensus        LFCYTLSFILNVLEIL  DLFR PIGYLLNDINNILVVVNSSSSFVFYVKYSTR  YR QLRTL  I W AA   F

          A. suum FRPR-3     ----------------------------------------------------
       A. caninum FRPR-3     TSIKTAFTDKQKNRR-------------------------------------
 N. brasiliensis FRPR-3     ----------------------------------------------------
     H. contortus FRPR-3     ----------------------------------------------------
       C. elegans FRPR-3     RGTSTRLSSSHNLLYKPSYSKPCDI---------------------------
         S. ratti FRPR-3     ----------------------------------------------------
    B. xylophilus FRPR-3     SSRQTVYTSATALDNSQVDTSLKPPVRKF-----------------------
       G. pallida FRPR-3     G------KQRLSTTVLSEWSHRTPSPFLL-----------------------
         M. hapla FRPR-3     ----------------------------------------------------
     M. incognita FRPR-3     QNSYNINRQKMSSTFLTVASCNNNNNDYGGGGVTKQQQQKRLRANSANPCPL

            Consensus
```

14. FRPR-18 homologues

```
     T. spiralis FRPR-18   ----------------------------------------------MDILLIGLAAADNALLLSGTPVYSVIALYTYR-PSEQLMKMMNHLTVTLYPISM
        T. muris FRPR-18   --------------CSVLMIIVAVVGLIGNTIFIQLFWQSSS-PTSMDILLTGLAAADSALLLSGTPVFSVIALYTYS-PSVQLMKAMNYLTVTLYPISM
         A. suum FRPR-18   SLRLVENVMDSQHLMASAILLIVCIGVLGNLVSLFMFSRPHMRTASVNVLLSALSAVDLGLLVLSIPVFVVPGLDPWQEDISAQYKYYAYMLKFVYPVNL
N. brasiliensis FRPR-18   --------MDSQQWMACAILSLVIVGCFGNSLSLLLFSRPHMRCFSVSLR----------------SSQQ------------SAVTYLAYVLKWVYPVNL
    H. contortus FRPR-18   --------MDSQQWMACAILSLVIIGCFGNSLSLLLFSRPHMRCSSVNVLLCALSFIDLSLLLLSIPTFVITNLDLW-----SAVTYLAYVLKWVYPVNL
      C. elegans FRPR-18   --------MESQQLMACAILVIVLVGIFGNSLSFILFSRPHMRSSSVNVLLCALSFFDFSLLTLSIPIFVIPNLDLWANDL-SLSTYMAYILKLIYPINL
        S. ratti FRPR-18   --------------------------------SFLVYTRYAT---SINILLTALSVIDICLLILATPVFVLPFMHIFY-FSFIEDTFQAYVLQYIYPLNC
   B. xylophilus FRPR-18   -----MEEDNLQQYLASATLMIVVIGCLGNFLSLILFLRHSS---SVNTLLAALSVVDLCLLLLAVPVFVMPSLDIWP-DQTSLYNFLSCVLKYVYPVNL
      G. pallida FRPR-18   -------MDTLRHILASSSLAMVIFGCFGNCLSAIIFLQRKT---SINILLSALAFIDIGLLLFAIPVFILPTLDRDG--FITLESVQAIVVKFLYPINL
        M. hapla FRPR-18   -------MELERHILASCTLSIIIFGCFGNILSALVFLQRTS---SINILLTALSLIDFGLLLFAIPVFVLPNLQSG--EHFYSEQFQAIVIKFLYPVNL
    M. incognita FRPR-18   -------MELERHLLASCTLAIIIFGCFGNILSALVFLQRTS---SINILLTALSLIDFGLLLFAIPVFVLPNLQSG--ELLYSEQFQAIVVKFLYPVNL

       Consensus                        LA A L IVIIGCFGN LS LLF R  S   SVNILLTALS ID GLLL AIPVFVLP L  W         FLAYVLKFLYPVNL

     T. spiralis FRPR-18   MAHCASVWTMVSISVERYIAVCHPLAARSLCSARRSV-AVLCVVTTTSVLYNFCRFWEYRID---------DHNRVVPLLRSNELYVQVFVHWMYFFTVF
        T. muris FRPR-18   MAHCASVWTMVSVSIERYIAVCHPLAIRSLCSPRKSV-TCLVVIAVLSVAYNFCRFWEYKLN---------HRNDVVPFLRSNELYVQVFVHWMYFFTVF
         A. suum FRPR-18   IMQTCSIYIMLLITVERWTAVCRPLQVRVWCSVRKSR-IALLVVLICAILYNLVRFFEYTIEVTPEGD--TVYARNLRDPDLYPYYMIGYFTASYLITHF
N. brasiliensis FRPR-18   IMQTCSVYTMVMITFERWTAVCRPLQVRVWCTPKKSR-IAVGCIFVSAVLYNFARFFEYRLIHTPDG---AIYERWLRDPANYRGYYIGYYTVLYVVTHF
    H. contortus FRPR-18   TMQTCSVYTMVMITFERWTAVCRPLHVSTWYAPRLSNNHDIIVLLLSCFLYLFTRFVYKLVYKPTVI---VTFSGKVYKITWVRGYYIGYYTILYVVTHF
      C. elegans FRPR-18   MMQTCSVYIMVMITLERWVAVCRPLQVRVWCTPRKSR-NAILVIIVSAFLYNFVRFFEYRFVVTESG---ALYEKWLRDPGKHRWYYVGYYTILYIVTHF
        S. ratti FRPR-18   ISQTCSVYVMVFITIERWTAIAKPLHVRFWCTTKSAK-MILTGVIISASIYNIPRFFEYEED-------HTTFKRILRDTVLYPNYMVYYYTLLYLLTHF
   B. xylophilus FRPR-18   MFQTCSIYIMVLITIERWTAVCKPLQVRIWCTARTSR-MALLCILVFAIAYNVIRFWEYQI---VETEKGLDYTRNLRDINRHPHYVIFYYTGLYLLTHF
      G. pallida FRPR-18   MFQTWSIYTIVLITAERWLAICRPLHVRSLCTPRTAK-WSLLFTALFSALYNIVRFWEYSLRSVWAPDGSLFFERNLRDPTEHPFYVLWYYSMLFLVTHF
        M. hapla FRPR-18   IFQTCSIYTIVLITAERWLAICRPLHVRSLCTPRTAK-RSLLCIFGFSIAYNIVRFWEYTL--IESSDGSLSFQRNLRNPNEHPWYMLGYYSLLFLLSHF
    M. incognita FRPR-18   IFQTCSIYTIVLITAERYLAICRPLHVRSLCTPRTAK-RSLFFILGFSIAYNIVRFWEYTL--IESSDGSLSFQRNLRNPNDHPWYMLGYYSLLFLLSHF

       Consensus           I QTCSVYTMVLITIERWLAVCRPL VRSWCTPR SK  ALL ILV AILYNIVRFWEY L           F R LRDP  HP YMIGYYTLLYLLTHF

     T. spiralis FRPR-18   GLPLFTLASVNCCIVHTIFRARRLRATMSRSQLRQHRLAVMMIVLVVIFFVSNTLAFVLNALEAAG-FFDNMKDDKYIFYCLMDVNNLLVEFNSSINLYL
        T. muris FRPR-18   GIPLLTLASVNCCIVNTIFRARRLRSTMSRSQLRQHRLAVMMVVLVLVFFISNALAFVLNALEAAG-FFDNVMYDRYIFYCLTDVNNLLIEFNSSINLFI
         A. suum FRPR-18   LIPFGIIIVMNGHVCCSMITLRRARLALTRQQQREYSTTFMLMMVTVMFALCNTLPFILNLVECALPDFFSNPETMYFAYQLNDISNLLVVLNSATTFII
N. brasiliensis FRPR-18   LVPFTIMAVLNGSVIVAMWRGRRMRQMMTRQQQREQSTTVMLLVVTIVFAVCNTLPFLLNVVESVFPDIFVDPRTTHIAYTLNDLSNLLVVLNSATTFLV
    H. contortus FRPR-18   LLPFTIMAVLNGSVIVTMWKGRQMRQMLTRQQQREQSTTVMLLVITILFAVCNTLPFILNVVESVFPDTFVDPRTTHIAYTVNDLSNLLVVLNSATTFLV
      C. elegans FRPR-18   LVPFSVMAFANGHVIVAMCKLSKTRQMLTRQQQREQSTTVMLLIVTFVFAICNTLPFLLNVSESIFPTLFQDESTRGLAYWLNDLSNLLVVLNSGTTFII
        S. ratti FRPR-18   CIPFAAIIFFNMHVIFLILKQKKERLNLTSQQRKEIKTTVMLIIVTVMFAICNVLTFIMNLWENINPTLFSDPKTLSMAYMFNDLANFLVILNSASTFII
   B. xylophilus FRPR-18   LIPFSVIIIMNGHVCKQIIQLRRARLMLTRQQQREQSTTLMLLVVTLVFAVCNTLPFLLNLAECIKRDLFEAESTAWIAYQLNDLSNLLVVLNSSTTWIV
      G. pallida FRPR-18   LLPFGTIFTLNAVVCHRILRLRSVREQLTMK---------MLLLVTLAFALCNALPFLLNLAECIKRDLFEAERTAQMAYLLNDLANFLVILNSSTTWIF
        M. hapla FRPR-18   LLPFGTIISLNGLLAHRILHLRSIREQLTNKQLTEHNTTAMLLLVTLSFALCNVLPFLLNLAECVQSDLFINERTASTAFILNDLANFLVVLNSSTTWIY
    M. incognita FRPR-18   LLPFGTIISLNGLLAHRILNLRSIREQLTNKQLREHNTTAMLLLVTLSFALCNVLPFLLNLAECVQSDLFVNESTAATAFILNDLANFLVVLNSSTTWIY
```

```
        Consensus      LLPF   I   LNG VI   ILRLRRIR   LTRQQ RE   TTVMLLVVTLVFALCNTLPFLLNL E I   DLF     T   IAY LNDLSNLLVVLNSSTTFII

     T. spiralis FRPR-18     YLIFCRRFRTRFCRMLRCL-------------NTASSLPLPFAFPTHADKEPYSKTGLNLSLQRRNLNNSSFDVTSAYYD--------------------
        T. muris FRPR-18     YLIFCRRLRARFWYLFRCS-------------KPEGKIPL---FPFRSVERMSSLRRSTSATYRR---RSS-----------------------------
         A. suum FRPR-18     YFIFSAKYRQTILLFLRHGCFWDHSRGQCYDNGRSHSLRYVSSTRKHSSANALRSENLLAPAAMQKRTERSASVQCATTAHPKSLFREASYRTPTPHRCV
N. brasiliensis FRPR-18     YFSFSEKYRQTLIFIIKNGCCASIS-----DYNNYTAMSRTASMRMNN------SVDLYRAGSKMSTSSR------------------------------
   H. contortus FRPR-18     YFTFSEKYRQTFIFIIKNGCCASIS-----DYNTYTAMSRTASMRVNNS-----TSDLQRAGSKKSTSR-------------------------------
      C. elegans FRPR-18     YFTFSEKYRQTLVFILKNGCCATVS-----DYNNYTAMSRTASMRISSET----GGQIQRQGSKMSNSSRSSDVLLKPIYMQKRSERFSSEYNERTCKHL
        S. ratti FRPR-18     YFLFSEKYRTLLFDIFR---------------YGCTKTKRENYYNNTFSRSLSIR---------------------------------------------
  B. xylophilus FRPR-18     YIIFSAKYRETAMRILWRCNTVYEKDVKYNSLSRTHSMRASSARKGPATSCTTESIRTLQSRTNRSNSECDERIRKHVSSIISDKFKRDSKNNKTSNGYR
      G. pallida FRPR-18     YTSFSAKFREGVAALFGLLSQD------------------------------------------------------------------------------
        M. hapla FRPR-18     YAVFSAKYREGVAYLFG---------------LRTLDRRRSGLVTNTIEERSNSACSNYNSATGGIQYFEPNKL--------------------------
    M. incognita FRPR-18     YAVFSAKYREGVAYLFG---------------LRTLDRRRSGLVTNTVEERSNSACSNYNSATGGMQNFEPNKL--------------------------

              Consensus      Y IFS KYR  L FILR                   AM RS S                 SAS          V

     T. spiralis FRPR-18     ------------------------------------------------------
        T. muris FRPR-18     ------------------------------------------------------
         A. suum FRPR-18     GKFREKLNGALPETSLRLTEQASKSDS---------------------------
N. brasiliensis FRPR-18     ------------------------------------------------------
   H. contortus FRPR-18     ------------------------------------------------------
      C. elegans FRPR-18     APFEEHKLPKLPSEKRKKKLHKMSAVEHRGMPEITITFSEDLPDGEPDSPCQPC
        S. ratti FRPR-18     ------------------------------------------------------
  B. xylophilus FRPR-18     VSTQASKKESSSSRLSLEVQPSTELLSPPPMTFRAPSPKWSEQPDRPEV-----
      G. pallida FRPR-18     ------------------------------------------------------
        M. hapla FRPR-18     ------------------------------------------------------
    M. incognita FRPR-18     ------------------------------------------------------

              Consensus
```
