# Supplementary material for: New insights into the FLPergic complements of parasitic nematodes: Informing deorphanisation approaches
Source: EuPA Open Proteom. 2014 Apr 19;3:262–72. doi: 10.1016/j.euprot.2014.04.002 (PMC4405611; doi:10.1016/j.euprot.2014.04.002)
Supplement: Supplementary Table 1 — Nematode Genome and Transcriptome BLAST databases/servers employed. BI, denotes Broad Institute; 959, denotes 959 Nematode Genomes; SI, denotes Sanger Institute; WB, denotes WormBase; INRA, denotes INRA Genomic Resources; EST, denotes Expressed Sequence Tags; NR/NT, denotes Nucleotide Collection; TSA, denotes Transcriptome Shotgun/RNA-seq Assembly; GSS, denotes Genome Survey Sequences; WS231-8, denotes Wormbase release WS231-8; PP, denotes Predicted Proteins; 454, denotes 454 reads; GS/C, denotes the most recent Genomic Scaffolds/Contigs available; UP, denotes unplaced reads; P, denotes published genome; UOG, denotes unpublished on-going; UIA, denotes unpublished in annotation; GOG, denotes GSS on-going; 454OG, denotes 454 on-going. NCBI: http://www.ncbi.nlm.nih.gov/; WormBase: http://www.wormbase.org/tools/blast_blat; 959 Nematode genomes: http://www.nematodes.org/nematodegenomes/index.php/959_Nematode_Genomes; The Sanger institute: http://www.sanger.ac.uk/resources/software/blast/; The Broad institute: http://www.broadinstitute.org/annotation/genome/filarial_worms/Blast.html; INRA Genomic resources: http://meloidogyne.toulouse.inra.fr/blast/blast.html. [file mmc4.docx]

**Supplementary Table 1. Nematode Genome and Transcriptome BLAST databases/servers employed.**

| **Clade** |  | **Species** | **Transcriptome BLAST server** | **Transcriptome database** | **Genome BLAST server** | **Genome database (version)** | **Genome status** |
| --- | --- | --- | --- | --- | --- | --- | --- |
|  | **2** | ***Trichinella***  ***spiralis*** | NCBI | EST; NR/NT | WB | Genome  (WS231-8) | P^3^ |
|  |  | ***Trichuris***  ***muris*** | NCBI | EST; NR/NT | SI | GS/C | UOG |
|  | **8** | ***Brugia***  ***malayi*** | NCBI | EST; NR/NT | BI | GS/C | P^4^ |
|  |  | ***Wuchereria***  ***bancrofti*** | NCBI; BI | EST; NR/NT; TSA | BI | GS/C | UIA |
|  |  | ***Dirofilaris***  ***immitis*** | NCBI; 959 | EST; NR/NT; TSA | 959 | GS/C  (version 1.3) | P^5^ |
|  |  | ***Onchocerca***  ***vovulus*** | NCBI | EST; NR/NT | SI | GS/C; PP  (version 3) | UOG |
|  |  | ***Onchocerca***  ***ochengi*** | NCBI | EST; NR/NT | 959 | GS/C  (version 0.3) | UIA |
|  |  | ***Loa***  ***loa*** | NCBI; BI | EST; NR/NT; TSA | BI | GS/C  (version 3) | UIA |
|  |  | ***Ascaris***  ***suum*** | NCBI | EST; NR/NT; TSA | 959 | GS/C | P^6^ |
|  | **9** | ***Ancylostoma caninum*** | NCBI | EST; NR/NT | NCBI | GSS | GOG^7^ |
|  |  | ***Nippostrongylus brasiliensis*** | NCBI; SI | EST; NR/NT; TSA | SI | 454 | 454OG |
|  |  | ***Haemonchus contortus*** | NCBI | EST; NR/NT | SI | GS/C (12/06/12); 454 (26/08/09) | UOG |
|  | **10** | ***Strongyloides***  ***ratti*** | NCBI | EST; NR/NT | WB | Genome  (WS231-8) | UIA |
|  |  | ***Bursaphelenchus xylophilus*** | NCBI | EST; NR/NT | 959 | GS/C | P^1^ |
|  | **12** | ***Globodera***  ***pallida*** | NCBI | EST; NR/NT | SI | GS/C, PP (05/12); 454 | UOG |
|  |  | ***Meloidogyne***  ***hapla*** | NCBI | EST; NR/NT | WB | Genome  (WS231-8) | P^8^ |
|  |  | ***Meloidogyne incognita*** | NCBI | EST; NR/NT | INRA | GS/C; UR; PP^2^  (CNS 2007-10) | P^2^ |

References:

[1] Kikuchi T, Cotton Ja, Dalzell JJ, Hasegawa K, Kanzaki N, McVeigh P, et al. Genomic insights into the origin of parasitism in the emerging plant pathogen Bursaphelenchus xylophilus. PLoS Pathog 2011;7:e1002219.

[2] Abad P, Gouzy J, Aury J-M, Castagnone-Sereno P, Danchin EGJ, Deleury E, et al. Genome sequence of the metazoan plant-parasitic nematode Meloidogyne incognita. Nat Biotechnol 2008;26:909–15.

[3] Mitreva M, Jasmer DP, Zarlenga DS, Wang Z, Abubucker S, Martin J, et al. The draft genome of the parasitic nematode Trichinella spiralis. Nat Genet 2011;43:228–35.

[4] Ghedin E, Wang S, Spiro D, Caler E, Zhao Q, Crabtree J, et al. Draft genome of the filarial nematode parasite Brugia malayi. Science 2007;317:1756–60.

[5] Godel C, Kumar S, Koutsovoulos G, Ludin P, Nilsson D, Comandatore F, et al. The genome of the heartworm, Dirofilaria immitis, reveals drug and vaccine targets. FASEB J 2012;26:4650–61.

[6] Jex AR, Liu S, Li B, Young ND, Hall RS, Li Y, et al. Ascaris suum draft genome. Nature 2011;479:529–33.

[7] Abubucker S, Martin J, Yin Y, Fulton L, Yang S-P, Hallsworth-Pepin K, et al. The canine hookworm genome: analysis and classification of Ancylostoma caninum survey sequences. Mol Biochem Parasitol 2008;157: 187–92.

[8] Opperman CH, Bird DM, Williamson VM, Rokhsar DS, Burke M, Cohn J, et al. Sequence and genetic map of Meloidogyne hapla: a compact nematode genome for plant parasitism. Proc Natl Acad Sci USA 2008;105:14802–7.
