# Supplementary material for: New insights into the FLPergic complements of parasitic nematodes: Informing deorphanisation approaches
Source: EuPA Open Proteom. 2014 Apr 19;3:262–72. doi: 10.1016/j.euprot.2014.04.002 (PMC4405611; doi:10.1016/j.euprot.2014.04.002)
Supplement: Supplementary Table 2 — flp gene BLAST query GenBank accession numbers. [file mmc5.pdf]

Table 2. *flp* gene BLAST query GenBank accession numbers.

flp	GenBank Accession Number
1	AAC46464.1
2	CAA90031.1
3	CAA90030.1
4	NP_496173.1
5	NP_509445.1
6	NP_505444.1
7	NP_508985.1
8	CAA93746.1
9	AAC08946.1
10	CCD63311.1
11	CCD68560.1
12	CCD63264.1
13	NP_501255.1
14	CAA21533.2
15	NP_499820.1
16	CAE17795.1
17	NP_503051.1
18	NP_508514.2
19	NP_509776.1
20	CCD65290.1
21	NP_505011.2
22	NP_492344.2
23	NP_498907.3
24	CCD65361.1
25	CAE54900.1
26	NP_741827.1
27	CCD65048.1
28	CAE17946.1
29	BQ837449
30	AW588622
31	BM882182
32	CAA93671.2
33	CAD82918.1
34	CCD62625.1
