# Supplementary material for: New insights into the FLPergic complements of parasitic nematodes: Informing deorphanisation approaches
Source: EuPA Open Proteom. 2014 Apr 19;3:262–72. doi: 10.1016/j.euprot.2014.04.002 (PMC4405611; doi:10.1016/j.euprot.2014.04.002)
Supplement: Supplementary Table 3 — flp / putative flp-GPCR BLAST query GenBank accession numbers. *denotes deorphanised FLP-GPCR-encoding gene. [file mmc6.pdf]

Table 3. *flp* / putative *flp*-GPCR BLAST query GenBank accession numbers.

flp- / putative-*flp*-GPCR	GenBank Accession Number
*npr-1**	CCD67081.1
npr-2	CAD59155.1
*npr-3**	CAB05681.1
*npr-4**	NP_510101.2
*npr-5**	CCD70460.1
npr-6	NP_509725.2
npr-7	NP_509570.3
*npr-10**	CAA15513.1
*npr-11**	NP_508234.2
npr-12	CCD62029.1
npr-13	NP_506659.2
*npr-22**	CCD64003.1
tkr-3	CCD61149.1
*frpr-3**	NP_505004.1
frpr-4	NP_494987.2
frpr-6	CCD61444.1
frpr-11	NP_505301.1
frpr-12	CCD72369.1
frpr-16	CCD72321.1
*frpr-18**	CCD68155.1
*egl-6**	ACG61341.1
dmsr-1	CAA98492.1
dmsr-2	CCD68134.1
dmsr-3	CAB55110.1
dmsr-4	NP_493716.1
dmsr-5	CAA19526.1
dmsr-6	NP_497004.1
dmsr-7	NP_504431.2
dmsr-8	NP_505697.2
dmsr-9	CCD72499.1
dmsr-10	NP_504724.1
dmsr-11	NP_504725.2
dmsr-12	NP_504726.1
dmsr-13	NP_504730.1
dmsr-14	NP_504729.1
dmsr-15	CCD68165.1
dmsr-16	CCD72487.1
